# Synthesis of esters of diaminotruxillic bis-amino acids by Pd-mediated photocycloaddition of analogs of the Kaede protein chromophore

**DOI:** 10.3762/bjoc.16.98

**Published:** 2020-05-25

**Authors:** Esteban P Urriolabeitia, Pablo Sánchez, Alexandra Pop, Cristian Silvestru, Eduardo Laga, Ana I Jiménez, Carlos Cativiela

**Affiliations:** 1 Instituto de Síntesis Química y Catálisis Homogénea, ISQCH (CSIC - Universidad de Zaragoza), Pedro Cerbuna 12, E-50009 Zaragoza, Spain; 2 Supramolecular Organic and Organometallic Chemistry Centre, Departament of Chemistry, Faculty of Chemistry and Chemical Engineering, Babeş-Bolyai University, Str. Arany Janos 11, RO-400028 Cluj−Napoca, Romania

**Keywords:** amino acids, C–H activation, Kaede protein, oxazolones, photocycloaddition

## Abstract

The stereoselective synthesis of truxillic bis-amino esters from polyfunctional oxazolones is reported. The reaction of 4-((*Z*)-arylidene)-2-(*E*)-styryl-5(4*H*)-oxazolones **2** with Pd(OAc)_2_ resulted in *ortho*-palladation and the formation of a dinuclear open-book complexes **3** with carboxylate bridges, where the Pd atom is C^N bonded to the oxazolone. In **3** the two exocyclic C=C bonds of the oxazolone are in a face-to-face arrangement, which is optimal for their [2 + 2] photocycloaddition. Irradiation of dimers **3** in CH_2_Cl_2_ solution with blue light (465 nm) promoted the chemo- and stereoselective [2 + 2] photocycloaddition of the exocyclic C=C bonds and the formation of cyclobutane-containing *ortho*-palladated complexes **4**. Treatment of **4** with CO in a MeOH/NCMe mixture promoted the methoxycarbonylation of the palladated carbon and the release of the corresponding *ortho*-functionalized 1,3-diaminotruxillic bis-amino esters **5** as single isomers.

## Introduction

**Figure 1:**
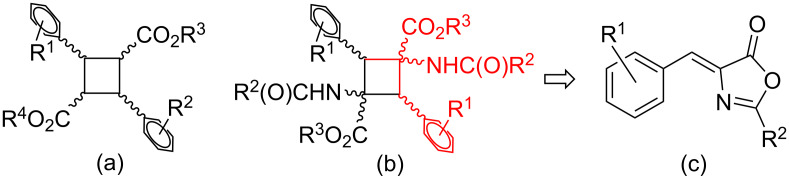
(a) General scheme for truxillic acid derivatives; (b) general scheme for symmetric 1,3-diaminotruxillic ester derivatives; (c) retrosynthesis: 4-arylidene-5(4*H*)-oxazolones are optimal precursors.

Truxillic acid derivatives (Figure 1a) are a special family of cyclobutanes that have been known since 1888 and show properties of high interest [1]. In this respect, their pharmacological activity has prompted extensive research that has focused on both their synthesis and the different pharmacological targets. Among them, probably the most important is their remarkable intrinsic anti-inflammatory and antinociceptive action, for which some mechanisms have been postulated to explain this activity [2–6]. The interest in this type of compound has gained even more relevance in the last few years due to the discovery that truxillic acid derivatives are inhibitors of FABP (fatty acid binding proteins), which are responsible for the cellular reuptake of anandamide, an endocannabinoid neurotransmitter, and that they can be involved in a very efficient treatment for chronic pain [7–10]. Moreover, alternative mechanisms to explain the antinociception of truxillic compounds have also been reported [11–12] and have generated intense debate. In addition, truxillic derivatives have shown remarkable activity as hepato-protective agents [13] and they also have applications as internal donors in Ziegler–Natta catalysts for polymerization [14] or as building blocks in polymer chemistry [15].

A very closely related group of cyclobutanes are the 1,3-diaminotruxillic ester derivatives (Figure 1b). This type of bis-amino esters also shows remarkable pharmacological activity, not only as antinociceptive drugs but also in the treatment of type 2 diabetes mellitus. In fact, recent results have shown that these compounds are the only non-peptidic agonists of the GLP-1R (glucagon-like peptide 1 receptor) and they have a higher stability than any other agonist prepared to date [16–18]. The properties outlined above highlight the importance of the wide scope of applications of this set of cyclobutanes.

Despite their importance, access to most of these compounds mainly relies in the well-known [2 + 2] photocycloaddition of olefins [19]. Unfortunately, the synthesis of truxillic or diaminotruxillic derivatives is far from being a highly efficient, selective and general process. Two different cases are usually found in the literature: (a) the direct [2 + 2] photocycloaddition of the corresponding olefins takes place, but shows poor efficiency (long reaction times, harsh irradiation conditions and low yields) [16,20], or (b) the direct [2 + 2] cycloaddition does not take place and it is necessary to synthesize a cyclobutane precursor and then functionalize it, thus increasing the number of reaction steps, the waste material, and decreasing the global yield [16,21]. Another drawback of these reactions is the selectivity, because good selectivity is achieved in photocycloadditions in solid state provided that the topochemical Schmidt's conditions are met [22–24], but the selectivity in solution is usually not as high [25–26].

**Figure 2:**
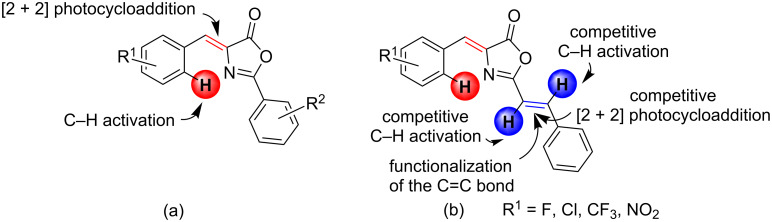
(a) (*Z*)-4-Arylidene-2-aryl-5(4*H*)-oxazolones used for the synthesis of 1,3-diaminotruxillic derivatives by direct [2 + 2] photocycloaddition of the exocyclic C=C bond or by incoporation of Pd to the oxazolone skeleton through C–H activation followed by [2 + 2] photocycloaddition; (b) (*Z*)-4-arylidene-2((*E*)-styryl)-5(4*H*)-oxazolones studied in this work with all synthetic possibilities shown.

Our group is interested in the synthesis of 1,3-diaminotruxillic derivatives and we have made some contributions to this area of research [27–30]. Firstly, we demonstrated that (*Z*)-4-arylidene-2-aryl-5(4*H*)-oxazolones (Figure 2a) are excellent precursors for the synthesis of this type of derivative, both by irradiation of the free species [27] and on using Pd complexes as templates [28–30]. The latter approach has shown good potential as a synthetic tool because it allows 1,3-diaminotruxillic derivatives to be obtained in a fully regio- and stereoselective way (only the ε-isomer is obtained). The process requires very mild reaction conditions in only three steps, with moderate to high yields in short reaction times under flow conditions [29], and it is compatible with substituents with different electronic properties (electron-releasing or electron-withdrawing) in the 4-arylidene ring. In addition, the *ortho*-position of the 2,4-aryl rings in the resulting bis-amino acids can be functionalized [30].

Substrates of the (*Z*)-4-arylidene-2((*E*)-styryl)-5(4*H*)-oxazolone type, shown in Figure 2b, are interesting starting materials for the synthesis of new 1,3-diaminotruxillic derivatives. These azlactones are precursors of the photoconvertible chromophore of the Kaede protein, an imidazolone with outstanding photophysical properties [31–38]. It is clear from Figure 2b that the molecular skeleton contains different reactive functional groups, which offer a variety of structural possibilities: two different competitive C=C bonds amenable to undergo photocycloaddition and at least three different C–H bonds that can be activated by a transition metal provide the potential for a rich organic and organometallic chemistry. Moreover, both the arylidene and the styryl groups can be tuned with different substituents to control the electronic and steric requirements of the ligand. We focused on precursors that have functional groups of interest (F, Cl, CF_3_, NO_2_) as substituents in the 4-arylidene ring. In this way the final products will contain either a functional group that can be reacted further (for instance C–Cl, C–NO_2_) or a functional group of interest from the medicinal or agrochemical point of view (for instance C–CF_3_, C–F) [39–41]. However, their reactivity is still totally unexplored in terms of both [2 + 2] photocycloaddition and metallations through C–H bond activations. In the present contribution we report the results obtained for these two different processes.

## Results and Discussion

### Synthesis and characterization of the (*Z*)-4-arylidene-2((*E*)-styryl)-5(4*H*)-oxazolones **2**

**Figure 3:**
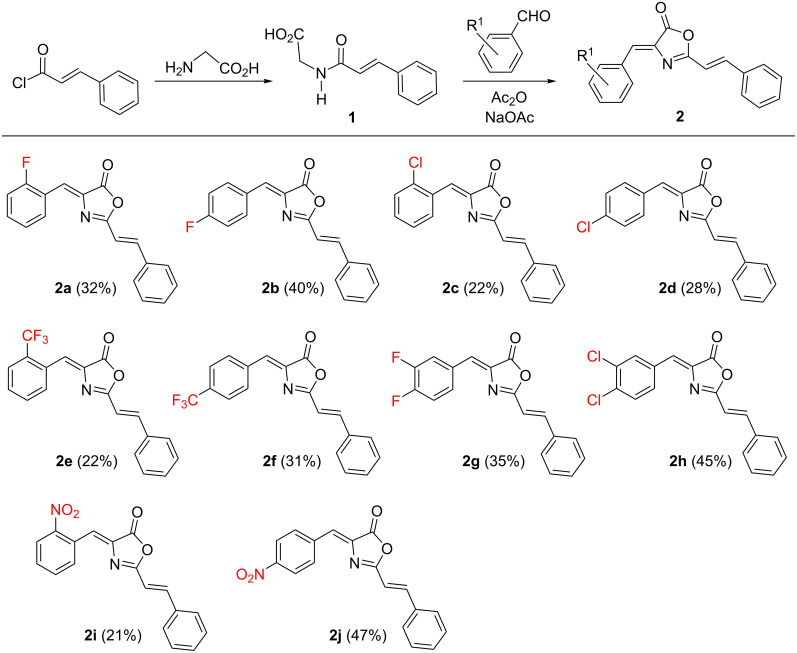
(*Z*)-4-Arylidene-2((*E*)-styryl)-5(4*H*)-oxazolones **2a**–**j** used in this work and overall reaction scheme.

The synthesis of the oxazolones **2** was carried out following the well-known Erlenmeyer–Plöchl method (Figure 3), by reaction of *N*-cinnamoylglycine (**1**) with the corresponding benzaldehyde ArCHO in acetic anhydride as solvent and in the presence of sodium acetate [42–48]. In turn, *N*-cinnamoylglycine (**1**) was prepared following the Schotten–Baumann method from glycine and cinnamoyl chloride [49]. All of the oxazolones used in this work are shown in Figure 3. The synthesis is quite general and tolerates substituents at different positions of the 4-arylidene ring, although the yields are only moderate to low. The lowest yields correspond to *ortho*-substituted substrates, which suggests that steric hindrance plays a role during the reaction. Oxazolones **2a**–**j** contain two exocyclic C=C bonds in their skeleton and these can, in principle, have different conformations. The NMR characterization of **2a**–**j** showed that they are obtained as single isomers. The oxazolone exocyclic C=C bond has the (*Z*)-configuration, because the signal assigned to the *C*=O group appears as a doublet in the proton-coupled ^13^C NMR spectrum, with a ^3^*J*_CH_ coupling constant of 5.6 Hz [50]. In contrast, the C=C bond of the styryl group has an (*E*)-configuration, as inferred from the observation of a ^3^*J*_HH_ coupling constant of 16 Hz between the two olefinic protons of the styryl fragment.

**Figure 4:**
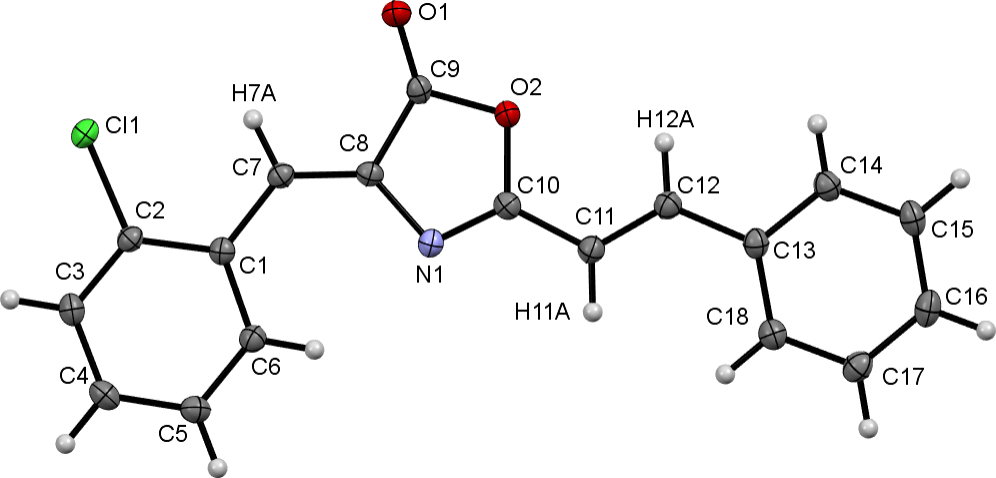
Molecular drawing of the oxazolone **2c**.

Further characterization of the new oxazolones was provided by the determination of the crystal structure of oxazolone **2c**. A molecular drawing of **2c** is shown in Figure 4, where the (*Z*)-configuration of the exocyclic C=C bond (C7–C8) and the (*E*)-configuration of the styryl moiety (C11–C12) are clearly seen, thus confirming the results obtained in solution. It is worth noting the orientation of the chlorine atom Cl1, which is *syn* with respect to the benzylidene proton. This orientation minimizes steric repulsions in the molecule and allows the establishment of an intramolecular contact between Cl1 and H7A. The non-bonding distance Cl1–H7A is 2.565(3) Å, which is much shorter than the sum of the van der Waals radii (2.95 Å) [51]. The same *syn* arrangement between the *ortho*-substituent and the benzylidene proton has also been observed in related *ortho*-substituted unsaturated 4-arylidene-oxazolones [52–59]. The internal bond distances and angles for the 4-arylidene fragment and the oxazolone ring are similar or identical, within experimental error, to those found in related oxazolones reported in the literature [52–59]. The styryl fragment bonded to an oxazolone ring has not been characterized previously by X-ray methods, but it shows internal bond distances and angles similar to those found in related 2-styrylimidazolone species [60].

### C–H Bond activation processes on (*Z*)-4-arylidene-2((*E*)-styryl)-5(4*H*)-oxazolones **2**

Once the starting oxazolones **2** had been prepared, their direct [2 + 2] photocycloaddition was attempted. However, irradiation of solutions of **2** in CD_2_Cl_2_ with blue LED light (465 nm), under the same conditions as reported previously by us for similar oxazolones [27], did not produce the expected cyclobutanes or gave only very low conversions (<5%) after long reaction times (96 h). As this reactivity was poor, it was decided to attempt the reaction using Pd complexes as templates [28–30]. The first step in this process was to study the C–H bond activation in oxazolones **2** promoted by Pd(OAc)_2_, which shows multiple possibilities (Figure 2b).

**Scheme 1:**
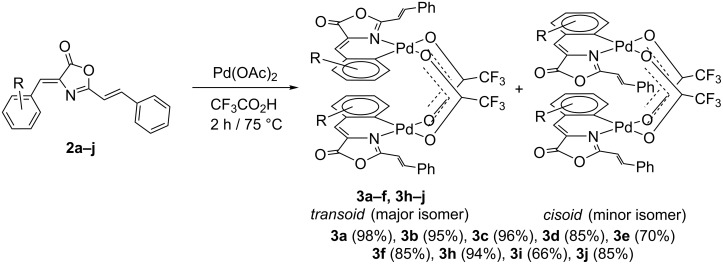
*Ortho*-palladation of oxazolones **2** by treatment with Pd(OAc)_2_ and different structures obtained for *ortho*-palladated complexes **3**.

Treatment of the oxazolones **2a**–**j** with Pd(OAc)_2_ (1:1 molar ratio) in CF_3_CO_2_H as solvent under reflux for 2 h resulted in the formation of the dinuclear complexes **3a**–**f** and **3h**–**j**, as shown in Scheme 1. In the case of oxazolone **2g** a very complex mixture was obtained and a pure compound could not be isolated.

Complexes **3** were obtained as air- and moisture-stable solids in good yields (see Experimental and Supporting Information File 1). In general, the yields were higher than 85%, which shows that the C–H activation is a general process and that the presence of strong electron-withdrawing groups is not an obstacle for the synthesis of the *ortho*-palladated derivatives. Only in the case of complexes **3e** and **3i** were the yields slightly lower than in the other cases (70% and 66%, respectively), probably due to two simultaneous factors, namely the presence of bulky CF_3_ and NO_2_ groups in *ortho* positions of the starting oxazolones **2e** and **2i**, and the strongly deactivating effect of the two groups on the C–H bond activation process. The reactions were usually carried out using 0.6 mmol of the starting material **2**. In this respect, it is remarkable that the C–H activation allows in this case scale-up of the reaction without a loss of yield. For oxazolone **2h** the reaction was carried out using 4.3 mmol of starting material and the final yield of analytically pure **3h** was 94% (see Experimental).

The characterization of complexes **3** shows that they are obtained as dinuclear derivatives, as inferred from the HRMS spectra. The oxazolone is bonded to the Pd(II) center as a C,N-chelate, as inferred by analysis of the ^1^H NMR spectra. The spectra show that the spin system of the styryl fragment –C(H)=C(H)Ph remains unaltered after the reaction, while the spin system of the 4-arylidene fragment (R–C_6_H_4_–C(H)=) changed to R–C_6_H_3_–C(H)=, thus showing the loss of one *ortho*-H. This strongly suggests that the C–H bond activation and incorporation of the Pd atom had occurred chemo- and regioselectively at the 4-arylidene ring. The same conclusions can be drawn from the analysis of the ^13^C NMR spectra, where the presence of the PdC_6_H_3_R–C(H)= group is clear. Therefore, the orientation of the C–H activation in these 4-aryliden-2-styryl-oxazolones is exactly the same as that observed for the 4-aryliden-2-aryloxazolones [28–30,56]. Signals corresponding to the activation of other C–H bonds present in oxazolones **2** (styryl, aryl) were not observed, so the reaction shows full selectivity towards the *ortho*-arylidene positions despite the presence of different C–H bonds that could be activated. Considering the dinuclear nature of compounds **3**, the C,N-chelate bonding mode of the *ortho*-palladated oxazolone and the ability of the carboxylate group to act as a bridging ligand in related complexes, we propose for complexes **3a**–**f** and **3h**–**j** the ‘open-book’ structure shown in Scheme 1.

The dimeric open-book structures of **3** can result in the formation of two isomers, the *transoid* (in which the two cyclopalladated oxazolones are in an *anti* arrangement) and the *cisoid* (*syn* arrangement). The presence of the two isomers in **3** is clear from the observation of two sets of signals due to the CF_3_CO_2_^–^ ligand in the ^19^F NMR spectra. The major isomer shows one singlet and this is assigned by symmetry to the *transoid* isomer, while the minor isomer shows two singlets and is assigned to the *cisoid* isomer. The *transoid* isomer is always the most abundant one, with *transoid*/*cisoid* ratios in the range from 70:30 (**3i**) to 96:4 (**3h**). Only the major *transoid* isomer in the mixture was fully characterized (see Experimental).

### Reactivity of *ortho*-palladated complexes **3** with light: intramolecular [2 + 2] photocycloaddition

**Scheme 2:**
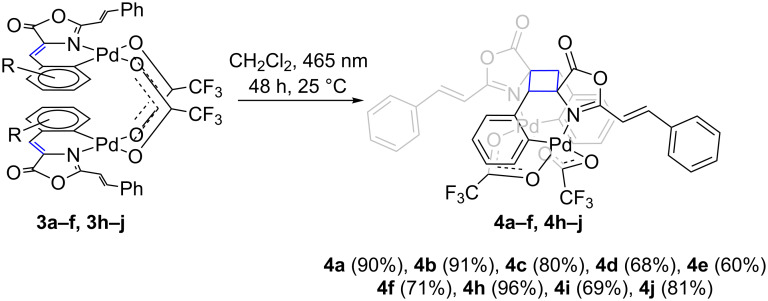
[2 + 2] Photocycloaddition of cyclopalladated complexes **3** in solution to give the dinuclear cyclobutane *ortho*-palladated complexes **4**.

Despite the lack of reactivity of free oxazolones **2** with blue light (465 nm), irradiation of the *ortho*-palladated complexes **3** with light of the same wavelength afforded better results. The irradiation of dinuclear derivatives **3a**–**f** and **3h**–**j** in CH_2_Cl_2_ solution with blue light (465 nm), provided by low-power LED lamps, took place with chemoselective [2 + 2] photocycloaddition of the oxazolone exocyclic C=C bonds and formation of the corresponding *ortho-*palladated cyclobutane derivatives **4a**–**f** and **4h**–**j**, as shown in Scheme 2.

Complexes **4a–f** and **4h–j** were obtained as air- and moisture-stable solids in good to very good yields. This also applied to the synthesis of **4h**, which was performed at scale of 2 mmol (instead of 0.2 mmol in most of the other cases) and was obtained with 96% isolated yield. In order to understand the different reactivities of oxazolones **2** and complexes **3** towards light, the absorption UV–vis spectra of the oxazolones **2** and *ortho*-palladated complexes **3** (CH_2_Cl_2_, 5 × 10^−4^ M) were measured and this provided some clues. The positions of the absorption maxima (λ_max_, nm) for both species are collected in Table 1.

**Table 1:** UV–vis spectra of **2** and **3**: absorption maxima (λ_max_, nm).

	oxazolones **2**	complexes **3**

**a**	379	457
**b**	376	454
**c**	377	459
**d**	381	459
**e**	375	467
**f**	380	461
**h**	381	466
**i**	383	464
**j**	385	457

According to the data in Table 1, the maximum absorption for oxazolones **2** is located in the near UV region, in the range of 375–385 nm, and this is quite far from the irradiation wavelength (465 nm). In fact, at 465 nm the UV–vis spectra of oxazolones **2** show that the absorption is zero or close to zero. Therefore, the lack of reactivity of species **2** can be related with the absence of the absorption of light. However, the *ortho*-palladation causes a clear bathochromic shift from compounds **2** to **3** and this results in absorption maxima for complexes **3** located in the range of 454–467 nm. In this case the use of blue light (465 nm) is optimal for their irradiation, because the irradiation wavelength matches the maximum absorption.

NMR characterization of complexes **4** showed that all compounds were obtained as a single stereoisomer, despite the presence of two isomers in the starting materials **3**. The ^1^H NMR and ^13^C NMR spectra clearly showed the chemical equivalence of the two *ortho*-palladated fragments, while ^19^F NMR spectra showed the equivalence of the bridging trifluoroacetate ligands. This means that only the *transoid* isomers of complexes **3** were transformed into cyclobutanes **4**, as shown in Scheme 2. The small amount of *cisoid* isomer in **3** probably decomposed under the reaction conditons, because in some cases the presence of small amounts of black Pd^0^ was observed. The formation of the cyclobutane ring is inferred in the ^1^H NMR spectra from the disappearance from the aromatic region of the signal due to the vinyl proton of the oxazolone exocyclic C=C bond and the appearance of a new singlet in the 4.8–5.7 ppm region. Further evidence can be found in the ^13^C NMR spectra, where the two peaks due to the exocyclic C(H)=C bond disappeared and two new signals appeared at around 68–69 ppm (quaternary C) and 51–60 ppm (CH). These facts are consistent with the expected hybridization change from Csp^2^ to Csp^3^ after formation of the cyclobutane ring.

**Figure 5:**
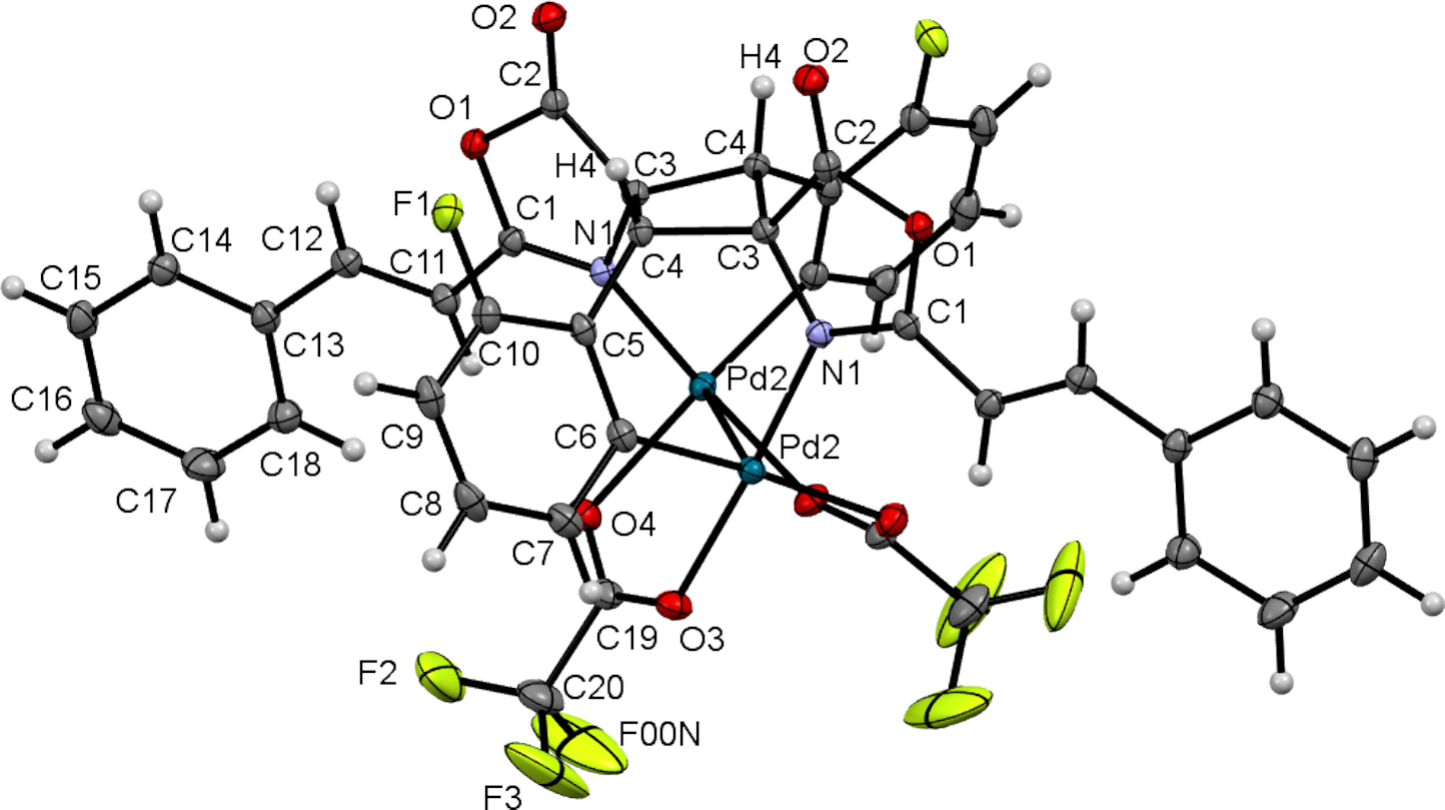
Molecular drawing of cyclobutane *ortho*-palladated **4a**. Ellipsoids are shown at the 50% probability level.

Determination of the crystal structure of complex **4a**, which is shown in Figure 5, provides additional information. Complex **4a** has a dinuclear structure in which each Pd atom is surrounded by one C,N-*ortho*-metallated oxazolone and two oxygen atoms of the trifluoroacetate ligands, which act as bridging ligands between the two Pd(C^N) fragments. In turn, the two Pd(C^N) fragments are linked by the cyclobutane ring formed by [2 + 2] photocycloaddition of the oxazolone exocyclic C=C bonds. The resulting configuration of the C3–C4–C3'–C4' cyclobutane ring, in which the carbonyl groups are on one side of the plane containing the cyclobutane and the nitrogen and the *ortho*-palladated ring on the other side of the plane, shows that the compound obtained is the epsilon (ε) isomer, according to the classification of Stoermer [61–62]. The non-bonding intramolecular distance between the *ortho*-fluorine F1 and the cyclobutane proton H4 is d(F1^...^H4) = 2.286(3) Å, which is much shorter than the sum of the van der Waals radii (2.67 Å) [51]. This suggest a close interaction between these two atoms, as observed in the crystal structure of **2c** (see above). The analysis of other internal bond distances and angles in the coordination sphere of the Pd atom shows that they are similar to those found in related examples reported in the literature and do not show additional noteworthy features [28–30].

From the observation of the molecular structure of **4a**, it is clear how the geometrical constraints of the ligands in complexes **3** establish unequivocally the stereoselective formation of the ε-isomer in **4**: (1) the initial (*Z*)-configuration of the oxazolones **2** is retained during the C–H bond activation step to give **3**; (2) the relative *transoid* arrangement in **3** establishes the 1,3-head-to-tail coupling of the exocyclic C=C bonds; (3) the template effect of the Pd_2_(O_2_CCF_3_)_2_ moiety establishes the *syn* approach of the C=C bonds. As a result, only the ε-isomer can be obtained and the stereoselectivity of the method is complete. As discussed previously, photocycloaddition products from the [2 + 2] reaction of the *cisoid* isomers of **3** were not observed, and we are unaware of the reasons for this lack of reactivity.

### Release of the 1,3-truxillic derivative by methoxycarbonylation

The last step to achieve the synthesis of the 1,3-diaminotruxillic targets was the release of the cyclobutane from the Pd_2_(O_2_CCF_3_)_2_ template. We previously reported that hydrogenation and halogenation were adequate tools to liberate 1,3-diaminotruxillics from the organopalladium template [29–30]. We employed similar reactions in this case with complexes **4**, but none of the attempts gave satisfactory results. Therefore, we discarded them and investigated other alternatives.

**Scheme 3:**
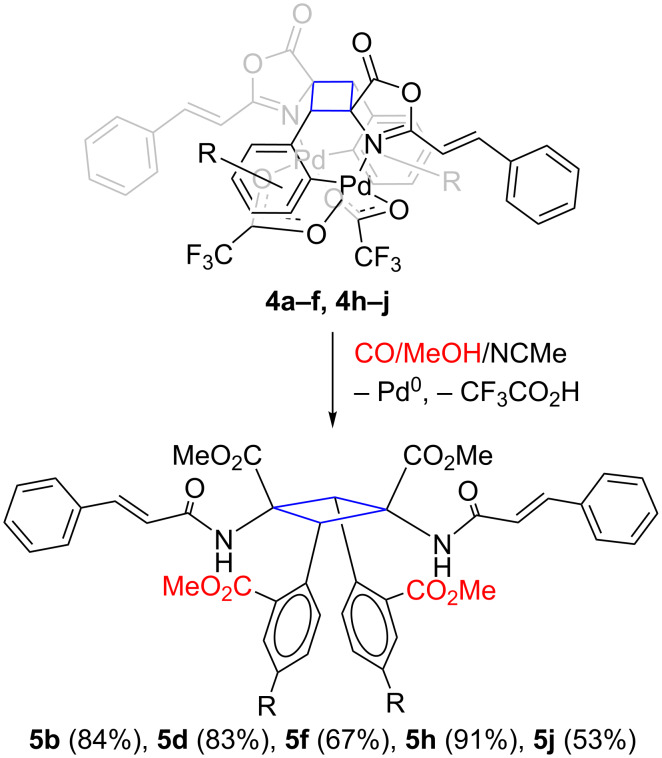
Release of the 1,3-diaminotruxillic bis-amino ester derivatives **5** by methoxycarbonylation of the Pd–C bond in cyclobutanes **4** and reductive elimination.

We found that the reaction of *ortho*-palladated complexes **4** with CO in methanol is a good option to promote the liberation of the organic cyclobutane, while retaining the functionalities already present (C–F, C–Cl, C–NO_2_, C–CF_3_ and –C(H)=C(H)– groups) and introducing a new functionality, i.e., the methoxycarbonyl group, selectively at the *ortho*-position of the aryl rings bonded to carbons C2 and C4 of the cyclobutane. Alkoxycarbonylation is a well-known reaction in Pd(II) chemistry [63–66]. Therefore, treatment of solutions of cyclobutanes **4** in a mixture of MeOH/NCMe (1:3) with CO (1 atm) at room temperature proceeded with decomposition of the organometallic core, precipitation of black Pd^0^ and concomitant formation of the cyclobutanes **5**, as represented in Scheme 3.

The methoxycarbonylation reaction to give **5** was not as general as the previous steps to give oxazolones **2** or cyclopalladated **3** and **4**. In the case of *para*- or *meta*-aryl-substituted derivatives **4** (**4b**, **4d**, **4f**, **4h** and **4j**) the reaction gave the corresponding cyclobutanes (**5b**, **5d**, **5f**, **5h** and **5j**) in moderate to good yields, but in the cases of the *ortho*-substituted compounds (**4a**, **4c**, **4e**, **4i**) intractable mixtures were obtained. The presence of two *ortho*-substituents in both aryl rings of the cyclobutanes (at C2 and C4) probably resulted in a strong steric hindrance around the already crowded cyclobutane ring, which gave rise to instability and decomposition. However, it is remarkable that all functional groups present in the starting materials **4** (C–F, C–Cl, C–NO_2_, C–CF_3_ and –C(H)=C(H)–) remained intact after the reaction, even in the presence of stoichiometric amounts of Pd^0^.

The presence of NCMe in large amounts in the reaction medium is critical. The reaction in methanol takes place at a very low rate but this increases markedly when NCMe is present. After a short screening of MeOH/NCMe ratios it was found that 1:3 was optimal. The ^1^H NMR spectrum of complex **4h** in NCMe/NCMe-*d*_3_ shows the presence of Pd-coordinated NCMe, which suggests that the role of the NCMe is the decoordination of the bridging carboxylate ligands by ligand exchange. The substitution of the bridging CF_3_CO_2_ by monodentate NCMe probably destabilizes the compact dinuclear scaffold, thus helping the coordination and further reactivity of CO. Nevertheless, the reaction is slow because it took about 16 h to reach completion. The reaction temperature is also critical, since room temperature is necessary in order to obtain a clean release of the truxillic esters. In addition to the methoxycarbonylation of the Pd–C, the reaction also involves ring opening of the oxazolone moiety to afford the corresponding ester and amido groups. This ring opening was also observed in the case of the hydrogenation and probably minimizes the steric strain around the cyclobutane ring.

The characterization of cyclobutanes **5** was performed by the usual methods. The HRMS spectra confirmed the loss of the Pd_2_(O_2_CCF_3_)_2_ unit and showed isotopic patterns in perfect agreement with the proposed stoichiometries. The ^1^H NMR spectra show in all cases the presence of two different CO_2_Me units and one N(H)C(O)C(H)=C(H)Ph moiety, thus demonstrating the incorporation of the methoxycarbonyl group and the ring opening of the oxazolone by methanol. The expected signals for the aryl and cyclobutane rings are also observed. These findings were also confirmed by analysis of the ^13^C NMR spectra.

## Conclusion

The synthesis of cyclobutane 1,3-diaminotruxillic bis-amino acids has been achieved with full stereoselectivity (ε-isomer) starting from polyfunctional oxazolones **2** derived from the chromophore of the Kaede protein in three steps. The new (*Z*)-4-arylidene-2-(*E*)-styryl-5(4*H*)-oxazolones **2** react with Pd(OAc)_2_ through a C–H bond activation reaction to give the dinuclear ‘open-book’ *ortho*-palladated complexes [Pd_2_(μ-O_2_CCF_3_)_2_(C^N-oxa))_2_] **3** with bridging carboxylate ligands. Despite the presence in **2** of different functional groups and different types of C–H bonds that could be activated, the C–H activation takes place regioselectively at the *ortho*-position of the 4-arylidene ring and leaves the remaining functional groups (C–F, C–Cl, C–NO_2_, C–CF_3_, C(H)=C(H)) intact. The ‘open-book’ complexes **3** show a face-to-face arrangement of the exocyclic C=C bonds of the oxazolone, which is optimal for their [2 + 2] photocycloaddition. Accordingly, irradiation of **3** with blue light (465 nm) results in the regioselective formation of the corresponding dinuclear *ortho*-metallated cyclobutane complexes **4**, which contain the skeleton of the 1,3-diaminotruxillic bis-amino acids. The reaction of the cyclobutanes **4** with CO (1 atm) in MeOH/NCMe results in the ring opening of the oxazolone group, methoxycarbonylation of the Pd–C bonds, reductive elimination, and finally release of the 1,3-diaminotruxillic bis-amino esters **5** as single isomers (ε-isomer).

The method shows great synthetic potential due to the selective formation of a single isomer, versatility and efficiency (high yields), general applicability and tolerance to the presence of different functional groups, although it is partially limited by steric hindrance in the final step. In addition, this approach allows the scale-up of the reaction without loss of yield of the final products.

## Experimental

### General methods

Solvents were obtained from commercial sources and used without further purification. All reactions were performed without special precautions against water and moisture. Thin-layer chromatography (TLC) was performed on Macherey-Nagel Polygram^®^ SIL G/UV254 silica gel on polyester sheets, with manganese-activated zinc silicate with green fluorescence for short-wave UV (254 nm) and special inorganic fluorescent pigment with blue fluorescence for long-wave UV (366 nm) as indicators. Fluka silica gel (pore size 60 Å, 70–230 mesh, 63–200 μm) was used for gravity column chromatography. C, H, N and S elemental microanalyses were carried out on a Perkin-Elmer 2400-B Series II Analyzer. High-resolution mass spectra-ESI (HRMS-ESI) were recorded using a Bruker MicroToF-Q™ equipped with an API-ESI source and a Q-ToF mass analyzer. Acetonitrile and methanol were used as solvents. Samples were introduced at a continuous flow of 0.2 mL/min, and nitrogen served both as the nebulizer gas and the dry gas. Infrared spectra were recorded on a Spectrum 100 Perkin-Elmer FTIR spectrophotometer, with a universal attenuated total reflectance (UATR) accessory made of thallium bromide-iodide crystals (KRS-5). The ^1^H, ^13^C{^1^H} and ^19^F NMR spectra were recorded on Bruker Avance-300 and -400 spectrometers (δ in ppm; *J* in Hz). All spectra were recorded at room temperature in solution, using CDCl_3_ as deuterated solvent (different conditions will be indicated). The ^1^H and ^13^C{^1^H} spectra are referenced using the residual solvent signal as internal standard, while ^19^F spectra are referenced to CFCl_3_. The ^1^H NMR peaks were assigned through standard 2D ^1^H−COSY (2K points in *t*_2_ using a spectral width of 10 ppm; 128 *t*_1_ experiments were recorded and zero-filled to 1K; for each *t*_1_ value four scans were signal-averaged using a recycle delay of 1 s) and selective 1D ^1^H-NOESY experiments. Typical mixing times in the case of selective 1D-NOESY experiments were in the range 1.2–1.8 s as a function of the irradiated signal. These optimized mixing times were set to be equal to the longitudinal relaxation time *t*_1_, determined using the inversion-recovery sequence. The ^13^C NMR peaks were identified using standard ^1^H−^13^C edited-HSQC and ^1^H−^13^C HMBC 2D-experiments. In both cases 4K points in *t*_2_ using spectral widths of 10 ppm (^1^H) and 200 ppm (^13^C) were used, with averaged values of the coupling constants ^1^*J*_CH_ = 145 Hz and long-range *^n^J*_CH_ = 10 Hz. Typically, 256 *t*_1_ experiments were recorded and zero-filled to 2K. For each *t*_1_ value 8 (HSQC) or 32 (HMBC) scans were signal-averaged using a recycle delay of 1 s. Absorption spectra were measured on a Thermo Scientific Evolution 600BB spectrophotometer. Cinnamoylglycine (**1**) was prepared by the Schotten–Baumann method [49].

### Irradiation setup for batch synthesis

The irradiation setup consisted of a flask (100 mL) irradiated by a printed circuit board (PCB) formed by 24 LEDs of 10 mm diameter each LED. The LEDs were serially connected in blocks of 6, with the minimum voltage possible (3 V) and a resistance of 60 Ω (according to Ohm's law). The output power per LED unit (blue, 465 nm) was 250 kmcd; the optical output power of the PCB of LEDs measured with a photometer (PM100D, Thorlabs) was 1 W. The PCB (dimensions: 7 × 6 cm) and the flask were placed inside a custom-built set-up for fixing the light source and the sample container, and dissipating the excess heat. A concave mirror was placed in front of the PCB to maximize the light that irradiated the balloon. Light emitting diodes (LEDs) were purchased from Topbright.

### X-ray crystallography

Single crystals of **2c** and **4a** of suitable quality for X-ray diffraction measurements were grown by slow diffusion of *n*-pentane into CH_2_Cl_2_ solutions of the crude product at –18 °C for several weeks. One selected single crystal was mounted at the end of a quartz fiber in a random orientation, covered with perfluorinated oil (*magic oil*) (**2c**) or paratone oil (**4a**) on MiTeGen microMounts cryoloop and placed under a cold stream of nitrogen gas. Crystallographic measurements were carried out at 100 K on Bruker Smart APEX CCD (**2c**) or Bruker D8 Venture (**4a**) diffractometers, using graphite monochromated Mo Kα radiation (λ = 0.71073 Å). A hemisphere of data was collected in each case based on ω-scan or φ-scan runs. The diffraction frames were integrated using the program SAINT [67] and the integrated intensities were corrected for absorption with SADABS [68]. The structures were solved and developed by Patterson and Fourier methods [69]. All non-hydrogen atoms were refined with anisotropic displacement parameters. The hydrogen atoms were placed at idealized positions and treated as riding atoms. Each hydrogen atom was assigned an isotropic displacement parameter equal to 1.2–1.5 times the equivalent isotropic displacement parameter of its parent atom. For structure solving and refinement the SHELX-97 [70], and Bruker APEX3 software package [71] were used. The structures were refined to F_o_^2^, and all reflections were used in the least-squares calculations [70] CCDC-1991019 (**2c**) and -1991018 (**4a**) contain the supplementary crystallographic data for this paper. These data can be obtained free of charge from the Cambridge Crystallographic Data Centre via http://www.ccdc.cam.ac.uk/data_request/cif.

### General synthesis of the 4-((*Z*)-arylidene)-2-((*E*)-styryl)-5(4*H*)-oxazolones **2a–j**

The oxazolones **2a**–**j** were prepared following the general Erlenmeyer–Plöchl method reported in the literature [42–48]. To a solution of *N*-cinnamoylglycine (**1**, 1.000 mg, 4.87 mmol) in acetic anhydride (5 mL), sodium acetate (400 mg, 4.87 mmol) and the corresponding benzaldehyde (4.87 mmol) were added. The resulting suspension was heated to 110 °C for 2 h and then allowed to cool to room temperature. The resulting deep yellow precipitate was stirred with ethanol (25 mL), filtered off, washed with water (20 mL), additional ethanol (25 mL) and diethyl ether (20 mL), and dried by suction. The resulting yellow solid was characterized as the corresponding oxazolone **2a**–**j**.

**Characterization of 4-((*Z*)-4-fluorobenzylidene)-2-((*E*)-styryl)oxazol-5(4*H*)-one (2b):** Following the general procedure, **1** (1000 mg, 4.87 mmol) was reacted with 4-fluorobenzaldehyde (0.513 mL, 4.87 mmol) and sodium acetate (400 mg, 4.87 mmol) in acetic anhydride (5 mL) to give **2b** as a yellow solid. Obtained: 577 mg, 1.969 mmol, 40% yield. ^1^H NMR (300.13 MHz, CDCl_3_) δ 8.16 (m, 2H, H_2'_, H_6'_, C_6_H_4_F), 7.70 (d, *J* = 16.2 Hz, 1H, H_β_), 7.59 (m, 2H, H_o_, C_6_H_5_), 7.49–7.38 (m, 3H, H_m_, H_p_, C_6_H_5_), 7.22–7.08 (m, 3H, H_3'_, H_5'_, C_6_H_4_F, H_vin_), 6.81 (d, *J* = 16.2 Hz, 1H, H_α_); ^13^C{^1^H} NMR (75.47 MHz, CDCl_3_) δ 167.3 (s, C=O), 164.3 (d, ^1^*J*_CF_ = 255.1 Hz, C-F, C_6_H_4_F), 163.6 (s, C=N), 144.2 (s, CH, =C_β_), 134.7 (d, ^3^*J*_CF_ = 8.5 Hz, CH, C_6_H_4_F), 133.3 (s, =C), 133.3 (s, C, C_ipso_, C_6_H_5_), 131.0 (s, CH, C_p_, C_6_H_5_), 130.1 (d, ^4^*J*_CF_ = 3.4 Hz, C, C_6_H_4_F), 129.9 (d, ^5^*J*_CF_ = 1.6 Hz, =CH, C_vin_), 129.3 (s, CH, C_m_, C_6_H_5_), 128.3 (s, CH, C_o_, C_6_H_5_), 116.3 (d, ^2^*J*_CF_ = 22.3 Hz, CH, C_6_H_4_F), 113.4 (s, CH, =C_α_); ^19^F NMR (282.40 MHz, CDCl_3_) δ −106.71 (tt, *J* = 8.5, 5.6 Hz); HRMS (ESI^+^) *m*/*z*: [M + Na]^+^: calcd for C_18_H_12_FNNaO_2_, 316.0750; found, 316.0719; IR (ν, cm^−1^): 1782 (νC=O), 1655 (νC=N).

### General synthesis of the *ortho*-palladated derivatives **3a–f**, **3h–j**

In a similar manner as described in [30], to a solution of the oxazolones **2a**–**j** in CF_3_CO_2_H (8 mL), the stoichiometric amount of Pd(OAc)_2_ (1:1 molar ratio) was added. The resulting mixture was heated at 75 °C for 2 h with stirring. During this time, the color of the suspension changed from yellow to reddish, and an increase in the amount of precipitated red solid was evident. After the reaction time the mixture was cooled to room temperature and distilled water (10 mL) was added. The resulting reddish solid was filtered off, washed with additional portions of distilled water until the smell of the trifluoroacetic acid disappeared (in general 3 × 10 mL was sufficient, but a larger amount could be necessary), dried in vacuo and characterized as the orthopalladated dimers **3a**–**f** and **3h**–**j**.

**Characterization of *ortho*-palladated complex 3b:** Following the general method, oxazolone **2b** (200 mg, 0.683 mmol) was reacted with Pd(OAc)_2_ (153 mg, 0.683 mmol) in CF_3_CO_2_H (8 mL) to give **3b** as a reddish solid. Obtained: 332 mg, 0.324 mmol, 95% yield. ^1^H NMR (300.13 MHz, CDCl_3_) δ 7.57 (m, 2H, H_o_, C_6_H_5_), 7.49 (d, *J* = 16.1 Hz, 1H, H_β_), 7.49–7.39 (m, 3H, H_m_, H_p_, C_6_H_5_), 7.30 (s, 1H, H_vin_), 7.21 (dd, *J* = 8.4, 6.1 Hz, 1H, H_2',_ C_6_H_3_F), 7.04 (d, *J* = 15.9 Hz, 1H, H_α_), 6.87 (td, *J* = 7.9, 2.4 Hz, 1H, H_3'_, C_6_H_3_F), 6.81 (dd, *J* = 10.1, 2.4 Hz, 1H, H_5'_, C_6_H_3_F); ^13^C{^1^H} NMR (75.47 MHz, CDCl_3_) δ 166.2 (q, ^2^*J*_CF_ = 39.1 Hz, C, *C*O_2_CF_3_), 165.7 (s, C=N), 161.8 (d, ^1^*J*_CF_ = 247.1 Hz, C–F, C_6_H_3_F), 160.0 (s, C=O), 149.2 (s, CH, =C_β_), 137.0 (d, ^3^*J*_CF_ = 6.8 Hz, C, C_6_H_3_F), 135.4 (s, CH, =C_vin_), 134.1 (d, ^3^*J*_CF_ = 8.7 Hz, CH, C_6_H_3_F), 133.5 (s, C, C_ipso_, C_6_H_5_), 132.5 (s, CH, C_p_, C_6_H_5_), 129.3 (s, CH, C_o_, C_6_H_5_), 129.2 (s, CH, C_m,_ C_6_H_5_), 126.2 (d, ^4^*J*_CF_ = 2.8 Hz, C, C_6_H_3_F), 122.0 (d, ^6^*J*_CF_ = 2.6 Hz, =C), 120.5 (d, ^2^*J*_CF_ = 22.3 Hz, CH, C_6_H_3_F), 115.0 (q, ^1^*J*_CF_ = 288.4 Hz, C, CF_3_), 113.5 (d, ^2^*J*_CF_ = 23.1 Hz, CH, C_6_H_3_F), 109.8 (s, CH, =C_α_); ^19^F NMR (282.40 MHz, CDCl_3_) δ −74.77 (s, CF_3_), −102.95 (ddd, *J* = 10.2, 7.5, 6.0 Hz, F_4'_, C_6_H_3_F). HRMS (ESI+) *m*/*z*: [M − COOCF_3_ + CH_3_O + Na]^+^ calcd. for, C_38_H_28_F_2_N_2_NaO_6_Pd_2_ 882.9887; found, 882.9908; IR (ν, cm^−1^): 1791 (νC=O), 1653 (νC=N), 1190 (νCF_3_).

### General synthesis of the *ortho*-palladated cyclobutanes **4a–f**, **4h–j**

The *ortho*-palladated dimers **3** (amount specified in each case) were suspended in CH_2_Cl_2_ (20–40 mL). The suspension was stirred at room temperature while irradiating with blue light (465 nm, see ‘irradiation setup’ section) for 48 h. As a rule of thumb, the initial suspension dissolved as the reaction progressed, and a clear solution was obtained after the reaction time. In a few cases some evidence of decomposition (presence of black Pd^0^) was observed. This was removed by filtration through a bed of Celite^®^. The resulting clear solution was evaporated to dryness to afford the corresponding *ortho*-palladated cyclobutanes **4** as deep yellow solids.

**Characterization of *ortho*-palladated cyclobutane 4b:** Following the general method, *ortho*-palladated **3b** (250 mg, 0.244 mmol) in CH_2_Cl_2_ (20 mL) was irradiated with blue light (465 nm) for 48 h to give *ortho*-palladated cyclobutane **4b** as a yellow solid. Obtained: 228 mg, 0.223 mmol, 91% yield. ^1^H NMR (300.13 MHz, CDCl_3_) δ 7.73 (d, *J* = 16.0 Hz, 1H, H_β_), 7.67 (m, 2H, H_o_, C_6_H_5_), 7.57 (d, *J* = 16.0 Hz, 1H, H_α_), 7.53–7.43 (m, 3H, H_m_, H_p_, C_6_H_5_), 6.86 (dd, *J* = 9.3, 2.4 Hz, 1H, H_5'_, C_6_H_3_F), 6.78 (dd, *J* = 8.3, 5.6 Hz, 1H, H_2'_, C_6_H_3_F), 6.71 (td, *J* = 8.0, 2.4 Hz, 1H, H_3'_, C_6_H_3_F), 4.95 (s, 1H, CH cyclobutane); ^13^C{^1^H} NMR (75.47 MHz, CDCl_3_) δ 172.2 (s, C=O), 168.9 (s, C, C_2_, C=N), 167.2 (q, ^2^*J*_CF_ = 36.4 Hz, C, *C*O_2_CF_3_), 160.1 (d, ^1^*J*_CF_ = 253.7 Hz, C-F, C_6_H_3_F), 151.2 (s, CH, =C_β_), 137.4 (d, ^3^*J*_CF_ = 6.2 Hz, C, C_6_H_3_F), 133.3 (s, C, C_ipso_, C_6_H_5_), 133.0 (s, CH, C_p_, C_6_H_5_), 130.0 (d, ^3^*J*_CF_ = 8.1 Hz, CH, C_6_H_3_F), 129.6 (s, CH, C_m_, C_6_H_5_), 129.6 (s, CH, C_o_, C_6_H_5_), 123.5 (d, ^4^*J*_CF_ = 2.9 Hz, C, C_6_H_3_F), 120.5 (d, ^2^*J*_CF_ = 22.1 Hz, CH, C_6_H_3_F), 115.3 (q, ^1^*J*_CF_ = 287.5 Hz, C, CF_3_) 113.2 (d, ^2^*J*_CF_ = 22.3 Hz, CH, C_6_H_3_F), 110.9 (s, CH, =C_α_), 69.0 (s, C, cyclobutane), 59.5 (s, CH, cyclobutane); ^19^F NMR (282.40 MHz, CDCl_3_) δ −74.60 (s, 3F, CF_3_), −110.53 (ddd, *J* = 9.0, 8.0, 5.7 Hz, 1F, F_ar_); anal. calcd for C_40_H_22_F_8_N_2_O_8_Pd_2_: C, 46.94; H, 2.17; N, 2.74; found: C, 46.81; H, 2.28; N, 2.94; IR (ν, cm^−1^): 1836 (νC=O), 1652 (νC=N), 1191 (νCF_3_).

### General synthesis of the *ortho*-methoxycarbonylated 1,3-diaminotruxillics **5**

The *ortho*-palladated cyclobutanes **4** (**4b**, **4d**, **4f**, **4h**, **4j**) were dissolved in a mixture of methanol and acetonitrile (1:3 ratio MeOH/NCMe). The solution was stirred under a CO atmosphere (1 atm; balloon) for 16 h. During the reaction time the decomposition of the organopalladium complex was evident as black Pd^0^ was formed. After the reaction time the resulting black suspension was filtered through a Celite^®^ pad to give a yellow solution. The Celite^®^ was washed with additional NCMe (10 mL) and the combined solutions were evaporated to dryness to afford the corresponding *ortho*-methoxycarbonylated 1,3-diaminotruxillic derivatives (**5b**, **5d**, **5f**, **5h**, **5j**) as yellow solids.

**Characterization of dimethyl 1,3-dicinnamamido-2,4-bis(4-fluoro-2-(methoxycarbonyl)phenyl)cyclobutane-1,3-dicarboxylate (5b):** Following the general method, a solution of the *ortho*-palladated cyclobutane **4b** (150 mg, 0.146 mmol) in a mixture of methanol (5 mL) and NCMe (15 mL) was stirred under a CO atmosphere (1 atm) for 16 h to give the truxillic cyclobutane derivative **5b** as a pale yellow solid. Obtained: 95 mg, 0.124 mmol, 84% yield. ^1^H NMR (400.13 MHz, CDCl_3_) δ 7.70 (dd, *J* = 8.9, 5.5 Hz, 1H, H_6_, C_6_H_3_F), 7.52–7.47 (m, 2H, H_o_, C_6_H_5_), 7.42–7.36 (m, 6H, NH, H_3_, C_6_H_3_F, H_m_, H_p_, C_6_H_5_, H_β_), 7.09 (m, 1H, H_5_, C_6_H_3_F), 6.40 (d, *J* = 15.7 Hz, 1H, H_α_), 6.01 (s, 1H, cyclobutane), 4.00 (s, broad, 3H, OMe, CO_2_Me cyclobutane), 3.88 (s, 3H, OMe, Ar–CO_2_Me); ^13^C{^1^H} NMR (75.47 MHz, CDCl_3_) δ 172.3 (s, *C*O_2_Me cyclobutane), 167.9 (s, Ar-*C*O_2_Me), 166.6 (s, NH*C*=O), 161.3 (d, ^1^*J*_CF_ = 248.0 Hz, C–F, C_6_H_3_F), 142.8 (s, CH, =C_β_), 134.4 (s, C, C_ipso_, Ph), 133.6 (d, ^3^*J*_CF_ = 7.0 Hz, C, C_6_H_3_F), 131.3 (d, ^3^*J*_CF_ = 7.8 Hz, CH, C_6_H_3_F), 130.4 (s, CH, C_p_, Ph), 129.2 (s, C, C_6_H_3_F), 129.0 (s, CH, C_m_, Ph), 128.2 (s, CH, C_o_, Ph), 119.7 (s, CH, =C_α_), 118.4 (d, ^2^*J*_CF_ = 22.0 Hz, CH, C_6_H_3_F), 117.2 (d, ^2^*J*_CF_ = 23.0 Hz, CH, C_6_H_3_F), 65.8 (s, C, cyclobutane), 53.9 (s, CH, OMe, CO_2_*Me* cyclobutane), 52.7 (s, CH, OMe, Ar–CO_2_*Me*), 48.2 (s, CH, cyclobutane); ^19^F NMR (282.40 MHz, CDCl_3_) δ −113.89 (pseudo dt, *J* = 8.6, 5.8 Hz). HRMS (ESI^+^) *m*/*z*: [M + Na]^+^ calcd. for C_42_H_36_F_2_N_2_NaO_10_, 789.2236; found, 789.2245; IR (ν, cm^−1^): 1723 (νCO_2_Me).

## Supporting Information

File 1 Complete experimental section; copies of NMR spectra of **2** and **3**.

File 2 Copies on NMR spectra of compounds **4** and **5**, crystallographic tables of compounds **2c** and **4a**.
