# Supplementary material for: Synthesis of esters of diaminotruxillic bis-amino acids by Pd-mediated photocycloaddition of analogs of the Kaede protein chromophore
Source: Beilstein J Org Chem. 2020 May 25;16:1111–23. doi: 10.3762/bjoc.16.98 (PMC7277947; doi:10.3762/bjoc.16.98)
Supplement: File 1 — Complete experimental section; copies of NMR spectra of 2 and 3. [file Beilstein_J_Org_Chem-16-1111-s001.pdf]

BEILSTEIN JOURNAL OF ORGANIC CHEMISTRY

Supporting Information

for

Synthesis of esters of diaminotruxillic bis-amino acids by Pd-mediated photocycloaddition of analogs of the Kaede protein chromophore

Esteban P. Urriolabeitia, Pablo Sánchez, Alexandra Pop, Cristian Silvestru, Eduardo Laga, Ana I. Jiménez and Carlos Cativiela

Beilstein J. Org. Chem. **2020,** *16,* 1111–1123. doi:10.3762/bjoc.16.98

Complete experimental section; copies of NMR spectra of 2 and 3

License and Terms: This is a supporting information file under the terms of the Creative Commons Attribution License (http://creativecommons.org/licenses/by/4.0). Please note that the reuse, redistribution and reproduction in particular requires that the authors and source are credited.
The license is subject to the *Beilstein Journal of Organic Chemistry* terms and conditions: (https://www.beilstein-journals.org/bjoc)

Contains:

- Experimental section
- Copies on NMR spectra of compounds 2 and 3

Experimental section

Synthesis and characterization of oxazolones 2a–j

Characterization of 4-((*Z*)-2-fluorobenzylidene)-2-((*E*)-styryl)oxazol-5(4*H*)-one (2a)

Following the general procedure, **1** (1000 mg, 4.87 mmol) was reacted with 2-fluorobenzaldehyde (0.513 mL, 4.87 mmol) and sodium acetate (400 mg, 4.87 mmol) in acetic anhydride (5 mL) to give **2a** as a yellow solid. Obtained: 451 mg, 1.539 mmol, 32% yield. ^{1}H NMR (300.13 MHz, $CDCl_3$): δ = 8.76 (td, *J* = 7.7, 1.8 Hz, 1H, $H_{6'}$, C_6H_4F), 7.73 (d, *J* = 16.2 Hz, 1H, H_β), 7.61 (m, 2H, H_o, C_6H_5), 7.52 (s, 1H, H_{vin}), 7.47 – 7.40 (m, 4H, H_m, H_p, C_6H_5, $H_{4'}$, C_6H_4F), 7.27 (dd, *J* = 7.8, 1.3 Hz, 1H, $H_{5'}$), 7.13 (ddd, *J* = 9.8, 8.3, 1.3 Hz, 1H, $H_{3'}$, C_6H_4F), 6.83 (d, *J* = 16.2 Hz, 1H, H_α). ^{13}C{^{1}H} NMR (75.47 MHz, $CDCl_3$): δ = 167.0 (s, C=O), 164.1 (s, C=N), 162.0 (d, $^1J_{CF}$ = 256.4 Hz, C-F, C_6H_4F), 144.6 (s, CH, =C_β), 134.6 (d, $^4J_{CF}$ = 3 Hz, =C), 134.6 (s, C, C_{ipso}, C_6H_5), 133.0 (d, $^3J_{CF}$ = 8.4 Hz, CH, C_6H_4F), 132.7 (d, $^3J_{CF}$ = 1 Hz, CH, C_6H_4F), 131.1 (s, CH, C_p, C_6H_5), 129.3 (s, CH, C_m, C_6H_5), 128.4 (s, CH, C_o, C_6H_5), 124.8 (d, $^4J_{CF}$ = 3.7 Hz, CH, C_6H_4F), 122.0 (d, $^3J_{CF}$ = 7.5 Hz, =CH, C_{vin}), 119.9 (d, $^2J_{CF}$ = 19.7 Hz, C, C_6H_4F), 115.7 (d, $^2J_{CF}$ = 21.8 Hz, CH, C_6H_4F), 113.3 (s, CH, =C_α). ^{19}F NMR (282.40 MHz, $CDCl_3$): δ = -114.01 (ddd, *J* = 10.2, 7.4, 5.3 Hz). HRMS (ESI+) [*m/z*]: Calc. for $C_{18}H_{12}FNNaO_2$ $[M+Na]^+$: 316.0750. Exp.: 316.0683. IR (ν, cm^{-1}): 1784 (νC=O), 1658 (νC=N).

Characterization of 4-((*Z*)-2-chlorobenzylidene)-2-((*E*)-styryl)oxazol-5(4*H*)-one (2c)

Following the general procedure, **1** (1000 mg, 4.87 mmol) was reacted with 2-chlorobenzaldehyde (0.546 mL, 4.87 mmol) and sodium acetate (400 mg, 4.87 mmol) in acetic anhydride (5 mL) to give **2c** as a yellow solid. Obtained: 340 mg, 1.10 mmol, 23% yield. ^{1}H NMR (400.13 MHz, $CDCl_3$): δ = 8.77 (dd, *J* = 7.7, 2.0 Hz, 1H, $H_{3'}$, C_6H_4Cl), 7.70 (d, *J* = 16.2 Hz, 1H, H_β), 7.66 (s, 1H, H_{vin}), 7.57 (m, 2H, H_o, C_6H_5), 7.47 – 7.40 (m, 4H, H_m, H_p, C_6H_5, $H_{6'}$, C_6H_4Cl), 7.39 – 7.32 (m, 2H, $H_{4'}$, $H_{5'}$), 6.79 (d, *J* = 16.2 Hz, 1H, H_α). ^{13}C{^{1}H} NMR (75.47 MHz, $CDCl_3$): δ = 166.9 (s, C=O), 164.4 (s, C=N), 144.7 (s, =CH, C_β), 136.6 (s, C-Cl, C_6H_4Cl), 134.9 (s, =C), 134.5 (s, C, C_{ipso}, C_6H_5), 133.2 (s, CH, C_6H_4Cl), 131.9 (s, CH, C_6H_4Cl), 131.6 (s, C, C_6H_4Cl), 131.1 (s, CH, C_6H_4Cl), 130.1 (s, CH, C_p, C_6H_5), 129.2 (s, CH, C_m, C_6H_5), 128.4 (s, CH, C_o, C_6H_5), 127.3 (s, CH, C_6H_4Cl), 126.0 (s, =CH, C_{vin}), 113.2 (s, =CH, C_α). HRMS (ESI+) [*m/z*]: $C_{18}H_{12}ClNNaO_2$ $[M+Na]^+$: 332.0454. Exp.: 332.0455. IR (ν, cm^{-1}): 1779 (νC=O), 1652 (νC=N).

Characterization of 4-((*Z*)-4-chlorobenzylidene)-2-((*E*)-styryl)oxazol-5(4*H*)-one (2d)

Following the general procedure, **1** (910 mg, 4.43 mmol) was reacted with 4-chlorobenzaldehyde (620 mg, 4.43 mmol) and sodium acetate (364 mg, 4.43 mmol) in acetic anhydride (5 mL) to give **2d** as a yellow solid. Obtained: 385 mg, 1.25 mmol, 28% yield. ^{1}H NMR (300.13 MHz, $CDCl_3$): δ = 8.08 (d, *J* = 8.5 Hz, 2H, $H_{2'}$, $H_{6'}$, C_6H_4Cl), 7.72 (d, *J* = 16.2 Hz, 1H, H_β), 7.60 (m, 2H, H_o, C_6H_5), 7.50 – 7.37 (m, 5H, $H_{3'}$,$H_{5'}$, C_6H_4Cl, H_m, H_p, C_6H_5), 7.13 (s, 1H, H_{vin}), 6.82 (d, *J* = 16.2 Hz, 1H, H_α). ^{13}C{^{1}H} NMR (75.47 MHz, $CDCl_3$): δ = 167.2 (s, C=O), 163.8 (s, C=N), 144.5 (s, CH, =C_β), 137.3 (s, C-Cl, C_6H_4Cl), 134.6 (s, C, C_{ipso}, C_6H_5), 134.1 (s, =C), 133.5 (s, CH, C_6H_4Cl), 132.2 (s, C, C_6H_4Cl), 131.1 (s, CH, C_p, C_6H_5), 129.6 (s, CH, =C_{vin}), 129.4 (s, CH, C_m, C_6H_5), 129.3 (s, CH, C_6H_4Cl), 128.4 (s, CH, C_o, C_6H_5), 113.3 (s, CH, =C_α). HRMS (ESI+) [*m/z*]: $C_{18}H_{12}ClNNaO_2$ $[M+Na]^+$: 332.0454. Exp.: 332.0452. IR (ν, cm^{-1}): 1785 (νC=O), 1654 (νC=N).

Characterization of 4-((*Z*)-2-trifluoromethylbenzylidene)-2-((*E*)-styryl)oxazol-5(4*H*)-one (2e)

Following the general procedure, **1** (1000 mg, 4.87 mmol) was reacted with 2-trifluoromethyl-benzaldehyde (0.643 mL, 4.87 mmol) and sodium acetate (400 mg, 4.87 mmol) in acetic anhydride (5 mL) to give **2e** as a yellow solid. Obtained: 366 mg, 1.07 mmol, 22% yield. ^{1}H NMR (400.13 MHz, $CDCl_3$): δ = 8.80 (d, *J* = 7.9 Hz, 1H, $H_{6'}$, $C_6H_4CF_3$), 7.80 – 7.73 (m, 2H, $H_{3'}$, $C_6H_4CF_3$, H_β), 7.68 (t, *J* = 7.7 Hz, 1H, $H_{4'}$, $C_6H_4CF_3$), 7.60 (m, 2H, H_o, C_6H_5), 7.57 – 7.49 (m, 2H, $H_{5'}$, $C_6H_4CF_3$, H_{vin}), 7.47 – 7.41 (m, 3H, H_m, H_p, C_6H_5), 6.83 (d, *J* = 16.2 Hz, 1H, H_α). ^{13}C{^{1}H} NMR (75.47 MHz, $CDCl_3$): δ = 166.6 (s, C=O), 165.1 (s, C=N), 145.2 (s, CH, =C_β), 135.8 (s, =C), 134.5 (s, C, C_{ipso}, C_6H_5), 133.4 (s, CH, $C_6H_4CF_3$), 132.1 (d, $^4J_{CF}$ = 0.9 Hz, CH, $C_6H_4CF_3$), 131.4 (q, $^3J_{CF}$ = 1.8 Hz, C, $C_6H_4CF_3$), 131.3 (s, CH, C_p, C_6H_5), 130.2 (s, CH, $C_6H_4CF_3$), 129.7 (q, $^2J_{CF}$ = 31.9 Hz, C, $C_6H_4CF_3$), 129.3 (s, CH, C_m, C_6H_5), 128.5 (s, CH, C_o, C_6H_5), 126.3 (q, $^3J_{CF}$ = 5.8 Hz, CH, $C_6H_4CF_3$), 125.1 (q, $^4J_{CF}$ = 2.4 Hz, CH, =C_{vin}), 124.0 (q, $^1J_{CF}$ = 277.7 Hz, CF_3), 113.1 (s, CH, =C_α). ^{19}F NMR (282.40 MHz, $CDCl_3$): δ = -58.44 (s). HRMS (ESI+) [*m/z*]: $C_{20}H_{16}F_3NNaO_3$ $[M+Na+CH_3OH]^+$: 398.0980. Exp.: 398.0973. IR (ν, cm^{-1}): 1785 (νC=O), 1654 (νC=N), 1117 (νCF_3).

Characterization of 4-((*Z*)-4-trifluoromethylbenzylidene)-2-((*E*)-styryl)oxazol-5(4*H*)-one (2f)

Following the general procedure, **1** (1000 mg, 4.87 mmol) was reacted with 4-trifluoromethyl-benzaldehyde (0.665 mL, 4.87 mmol) and sodium acetate (400 mg, 4.87 mmol) in acetic anhydride (5 mL) to give **2f** as a yellow solid. Obtained: 515 mg, 1.50 mmol, 31% yield. ^{1}H NMR (300.13 MHz, $CDCl_3$): δ = 8.22 (d, *J* = 8.2 Hz, 2H, $H_{2'}$, $H_{6'}$, $C_6H_4CF_3$), 7.73 (d, *J* = 16.2

Hz, 1H, $H_β$), 7.69 (d, *J* = 8.4 Hz, 2H, $H_{3'}$, $H_{5'}$, $C_6H_4CF_3$), 7.59 (m, 2H, H_o, C_6H_5), 7.49 – 7.39 (m, 3H, H_m, H_p, C_6H_5), 7.15 (s, 1H, H_{vin}), 6.82 (d, *J* = 16.2 Hz, 1H, $H_α$). $^{13}C\{^1H\}$ NMR (75.47 MHz, $CDCl_3$): δ = 166.9 (s, C=O), 164.7 (s, C=N), 145.1 (s, C, =$C_β$), 136.9 (s, C, $C_6H_4CF_3$), 135.6 (s, =C), 134.5 (s, C, C_{ipso}, C_6H_5), 132.3 (s, CH, $C_6H_4CF_3$), 132.0 (q, $^2J_{CF}$ = 31.9 Hz, C, $C_6H_4CF_3$), 131.2 (s, CH, C_p, C_6H_5), 129.3 (s, CH, C_m, C_6H_5), 128.6 (s, CH, =C_{vin}), 128.5 (s, CH, C_o, C_6H_5), 125.8 (q, $^3J_{CF}$ = 4.1 Hz, CH, $C_6H_4CF_3$), 124.0 (q, $^1J_{CF}$ = 273.1 Hz, C, CF_3), 113.1 (s, CH, =$C_α$). ^{19}F NMR (282.40 MHz, $CDCl_3$): δ = -62.95 (s). HRMS (ESI+) [*m/z*]: $C_{20}H_{16}F_3NNaO_3$ [M+Na+CH_3OH]$^+$: 398.0980. Exp.: 398.0996. IR (ν, cm^{-1}): 1782 (νC=O), 1657 (νC=N), 1110 (νCF_3).

Characterization of 4-((*Z*)-3,4-difluorobenzylidene)-2-((*E*)-styryl)oxazol-5(4*H*)-one (2g)

Following the general procedure, **1** (1000 mg, 4.87 mmol) was reacted with 3,4-difluoro-benzaldehyde (0.537 mL, 4.87 mmol) and sodium acetate (400 mg, 4.87 mmol) in acetic anhydride (5 mL) to give **2g** as a yellow solid. Obtained: 532 mg, 1.71 mmol, 35% yield. ^1H NMR (400.13 MHz, $CDCl_3$): δ = 8.24 (ddd, *J* = 10.0, 7.9, 1.9 Hz, 1H, $H_{2'}$, $C_6H_3F_2$), 7.73 (d, *J* = 16.2 Hz, 1H, $H_β$), 7.71 (m, 1H, $H_{6'}$, $C_6H_3F_2$), 7.60 (m, 2H, H_o, C_6H_5), 7.52 – 7.38 (m, 3H, H_m, H_p, C_6H_5), 7.23 (m, 1H, $H_{5'}$, $C_6H_3F_2$), 7.07 (s, 1H, H_{vin}), 6.82 (d, *J* = 16.2 Hz, 1H, $H_α$). $^{13}C\{^1H\}$ NMR (75.47 MHz, $CDCl_3$): δ = 167.0 (s, C=O), 164.2 (s, C=N), 152.1 (dd, $^1J_{CF}$ = 256.1 Hz, $^2J_{CF}$ = 13.2 Hz, C-F, $C_6H_3F_2$), 150.7 (dd, $^1J_{CF}$ = 250.4 Hz, $^2J_{CF}$ = 13.8 Hz, C-F, $C_6H_3F_2$), 144.8 (s, CH, =$C_β$), 134.6 (s, C, C_{ipso}, C_6H_5), 134.5 (d, $^5J_{CF}$ = 3.0 Hz, =C), 131.2 (s, CH, C_p, C_6H_5), 130.9 (q, $^3J_{CF}$ = 6.8 Hz, $^4J_{CF}$ = 4.2 Hz, C, $C_6H_3F_2$), 129.4 (dd, $^3J_{CF}$ = 6.7 Hz, $^4J_{CF}$ = 3.5 Hz, CH, $C_6H_3F_2$), 129.3 (s, CH, C_m, C_6H_5), 128.5 (s, CH, C_o, C_6H_5), 128.4 (m, CH, =C_{vin}), 120.6 (d, $^2J_{CF}$ = 18.5 Hz, CH, $C_6H_3F_2$), 117.8 (d, $^2J_{CF}$ = 18.1 Hz, CH, $C_6H_3F_2$), 113.2 (s, CH, =$C_α$). ^{19}F NMR (282.40 MHz, $CDCl_3$): δ = -131.62 (dddd, *J* = 21.1, 9.9, 8.0, 4.5 Hz, $F_{3'}$, $C_6H_3F_2$), -136.03 (ddd, *J* = 20.8, 11.6, 8.1 Hz, $F_{4'}$, $C_6H_3F_2$). HRMS (ESI+) [*m/z*]: $C_{19}H_{15}F_2NNaO_3$ [M+Na+CH_3OH]$^+$: 366.0918. Exp.: 366.0944. IR (ν, cm^{-1}): 1780 (νC=O), 1652 (νC=N).

Characterization of 4-((*Z*)-3,4-dichlorobenzylidene)-2-((*E*)-styryl)oxazol-5(4*H*)-one (2h)

Following the general procedure, **1** (2000 mg, 9.75 mmol) was reacted with 3,4-dichloro-benzaldehyde (1706 mg, 9.75 mmol) and sodium acetate (800 mg, 9.75 mmol) in acetic anhydride (10 mL) to give **2h** as a yellow solid. Obtained: 1523 mg, 4.42 mmol, 45% yield. ^1H NMR (400.13 MHz, $CDCl_3$): δ = 8.32 (d, *J* = 2.0 Hz, 1H, $H_{2'}$, $C_6H_3Cl_2$), 7.91 (dd, *J* = 8.4, 2.0 Hz, 1H, $H_{6'}$, $C_6H_3Cl_2$), 7.74 (d, *J* = 16.2 Hz, 1H, $H_β$), 7.61 (m, 2H, H_o, C_6H_5), 7.52 (d, *J* = 8.4 Hz, 1H, $H_{5'}$, $C_6H_3Cl_2$), 7.49 – 7.41 (m, 3H, H_m, H_p, C_6H_5), 7.05 (s, 1H, H_{vin}), 6.84 (d, *J* = 16.2 Hz, 1H, $H_α$). $^{13}C\{^1H\}$ NMR (100.61 MHz, $CDCl_3$): δ = 166.7 (s, C=O), 164.3 (s, C=N),

144.9 (s, CH, =$C_β$), 135.1 (s, C-Cl, $C_6H_3Cl_2$), 134.9 (s, C-Cl, $C_6H_3Cl_2$), 134.4 (s, C, C_{ipso}, C_6H_5), 133.5 (s, C, $C_6H_3Cl_2$), 133.3 (s, CH, $C_6H_3Cl_2$), 133.2 (s, =C), 131.1 (s, CH, $C_6H_3Cl_2$), 131.1 (s, CH, C_p, C_6H_5), 130.8 (s, CH, $C_6H_3Cl_2$), 129.2 (s, CH, C_m, C_6H_5), 128.4 (s, CH, C_o, C_6H_5), 127.7 (s, CH, =C_{vin}), 113.1 (s, CH, =$C_α$). HRMS (ESI+) [*m/z*]: $C_{18}H_{11}Cl_2NNaO_2$ $[M+Na]^+$: 366.0065. Exp.: 365.9946. IR (ν, cm^{-1}): 1786 (νC=O), 1658 (νC=N).

Characterization of 4-((*Z*)-2-nitrobenzylidene)-2-((*E*)-styryl)oxazol-5(4*H*)-one (2i)

Following the general procedure, **1** (1000 mg, 4.87 mmol) was reacted with 2-nitrobenzaldehyde (736 mg, 4.87 mmol) and sodium acetate (400 mg, 4.87 mmol) in acetic anhydride (5 mL) to give **2i** as an orange solid. Obtained: 328 mg, 1.02 mmol, 21% yield. ^1H NMR (300.13 MHz, $CDCl_3$): δ = 8.55 (d, *J* = 7.9 Hz, 1H, $H_{3'}$, $C_6H_4NO_2$), 8.05 (d, *J* = 8.1 Hz, 1H, $H_{6'}$, $C_6H_4NO_2$), 7.76 (d, *J* = 16.2 Hz, 1H, $H_β$), 7.73 (t, *J* = 8.0 Hz, 1H, $H_{5'}$, $C_6H_4NO_2$), 7.65 (s, 1H, H_{vin}), 7.63 – 7.51 (m, 3H, $H_{4'}$, $C_6H_4NO_2$, H_o, C_6H_5), 7.52 – 7.36 (m, 3H, H_m, H_p, C_6H_5), 6.80 (d, *J* = 16.2 Hz, 1H, $H_α$). $^{13}C\{^1H\}$ NMR (75.47 MHz, $CDCl_3$): δ = 166.0 (s, C=O), 165.3 (s, C=N), 149.2 (s, C-N, $C_6H_4NO_2$), 145.5 (s, CH, =$C_β$), 136.5 (s, =C), 134.3 (s, C, C_{ipso}, C_6H_5), 133.3 (s, CH, $C_6H_4NO_2$), 133.0 (s, CH, $C_6H_4NO_2$), 131.2 (s, CH, C_p, C_6H_5), 130.6 (s, CH, $C_6H_4NO_2$), 129.2 (s, CH, C_m, C_6H_5), 128.4 (s, CH, C_o, C_6H_5), 128.0 (s, C, $C_6H_4NO_2$), 124.9 (s, CH, $C_6H_4NO_2$), 124.4 (s, CH, =C_{vin}), 112.9 (s, CH, =$C_α$). HRMS (ESI+) [*m/z*]: $C_{18}H_{12}N_2NaO_4$ $[M+Na]^+$: 343.0695. Exp.: 343.0666. IR (ν, cm^{-1}): 1791 (νC=O), 1653 (νC=N).

Characterization of 4-((*Z*)-4-nitrobenzylidene)-2-((*E*)-styryl)oxazol-5(4*H*)-one (2j)

Following the general procedure, **1** (1000 mg, 4.87 mmol) was reacted with 4-nitrobenzaldehyde (736 mg, 4.87 mmol) and sodium acetate (400 mg, 4.87 mmol) in acetic anhydride (5 mL) to give **2j** as an orange solid. Obtained: 736 mg, 2.3 mmol, 47% yield. ^1H NMR (300.13 MHz, $CDCl_3$): δ = 8.30 (s, 4H, $H_{2'}$, $H_{3'}$, $H_{5'}$, $H_{6'}$, $C_6H_4NO_2$), 7.80 (d, *J* = 16.2 Hz, 1H, $H_β$), 7.63 (m, 2H, H_o, C_6H_5), 7.53 – 7.41 (m, 3H, H_m, H_p, C_6H_5), 7.17 (s, 1H, H_{vin}), 6.85 (d, *J* = 16.2 Hz, 1H, $H_α$). $^{13}C\{^1H\}$ NMR (75.47 MHz, $CDCl_3$): δ = 166.6 (s, C=O), 165.5 (s, C=N), 148.4 (s, C-N, $C_6H_4NO_2$), 146.0 (s, CH, =$C_β$), 139.6 (s, C, $C_6H_4NO_2$), 136.8 (s, =C), 134.4 (s, C, C_{ipso}, C_6H_5), 132.7 (s, CH, $C_6H_4NO_2$), 131.5 (s, CH, C_p, C_6H_5), 129.4 (s, CH, C_m, C_6H_5), 128.6 (s, CH, C_o, C_6H_5), 127.1 (s, CH, =C_{vin}), 124.1 (s, CH, $C_6H_4NO_2$), 113.0 (s, CH, =$C_α$). HRMS (ESI+) [*m/z*]: $C_{19}H_{16}N_2NaO_5$ $[M+Na+CH_3OH]^+$: 375.0957. Exp.: 375.0976. IR (ν, cm^{-1}): 1783 (νC=O), 1655 (νC=N).

Synthesis and characterization of complexes 3[a]–f and 3h–j

Characterization of *ortho*-palladated complex 3a

Following the general method, oxazolone **2a** (200 mg, 0.683 mmol) was reacted with $Pd(OAc)_2$ (153 mg, 0.683 mmol) in CF_3CO_2H (8 mL) to give **3a** as a reddish solid. Obtained: 342 mg, 0.334 mmol, 98% yield. ^1H NMR (300.13 MHz, $CDCl_3$): δ = 7.68 (s, 1H, H_{vin}), 7.58 (m, 2H, H_o, C_6H_5), 7.49 (d, J = 16.0 Hz, 1H, $H_β$), 7.53 – 7.43 (m, 3H, H_m, H_p, C_6H_5), 7.09 (d, J = 15.9 Hz, 1H, $H_α$), 7.00 – 6.79 (m, 3H, $H_{3'}$, $H_{4'}$, $H_{5'}$, C_6H_3F). $^{13}C\{^1H\}$ NMR (75.47 MHz, $CDCl_3$): δ = 166.2 (s, C=N), 166.2 (q, $^2J_{CF}$ = 39.1 Hz, C, <u>C</u>O_2CF_3), 159.5 (d, $^1J_{CF}$ = 247.1 Hz, C-F, C_6H_3F), 159.3 (s, C=O), 149.5 (s, CH, =$C_β$), 135.0 (d, $^3J_{CF}$ = 3.3 Hz, C, C_6H_3F), 133.5 (s, C, C_{ipso}, C_6H_5), 132.6 (s, CH, C_p, C_6H_5), 131.4 (d, $^3J_{CF}$ = 8.6 Hz, CH, C_6H_3F), 129.4 (s, CH, C_o, C_6H_5), 129.2 (s, CH, C_m, C_6H_5), 129.1 (d, $^4J_{CF}$ = 3.3 Hz, CH, C_6H_3F), 127.0 (d, $^3J_{CF}$ = 8.9 Hz, CH, =C_{vin}), 122.8 (d, $^4J_{CF}$ = 3.7 Hz, =C), 118.5 (d, $^2J_{CF}$ = 8.9 Hz, C, C_6H_3F), 115.1 (q, $^1J_{CF}$ = 288.4 Hz, C, CF_3), 112.5 (d, $^2J_{CF}$ = 21.5 Hz, CH, C_6H_3F), 109.6 (s, CH, =$C_α$). ^{19}F NMR (282.40 MHz, $CDCl_3$): δ = -74.69 (s, CF_3), -111.80 (dd, J = 9.3, 5.8 Hz, $F_{2'}$, C_6H_3F). HRMS (ESI+) [*m/z*]: $C_{38}H_{28}F_2N_2NaO_6Pd_2$ [M-2COOCF$_3$+2CH$_3$O+Na]$^+$: 882.9887. Exp: 882.9872. IR (ν, cm^{-1}): 1796 (νC=O), 1651 (νC=N), 1193 (νCF_3).

Characterization of *ortho*-palladated complex 3c

Following the general method, oxazolone **2c** (200 mg, 0.646 mmol) was reacted with $Pd(OAc)_2$ (145 mg, 0.646 mmol) in CF_3CO_2H (8 mL) to give **3c** as a reddish solid. Obtained: 327 mg, 0.309 mmol, 96% yield. ^1H NMR (300.13 MHz, $CDCl_3$): δ = 7.93 (s, 1H, H_{vin}), 7.58 (d, J = 15.9 Hz, 1H, $H_β$), 7.57 (m, 2H, H_o, C_6H_5), 7.51 – 7.40 (m, 3H, H_m, H_p, C_6H_5), 7.18 (d, J = 7.8 Hz, 1H, $H_{5'}$, C_6H_3Cl), 7.09 (d, J = 15.9 Hz, 1H, $H_α$), 7.03 (d, J = 8.1 Hz, 1H, $H_{3'}$, C_6H_3Cl), 6.83 (t, J = 8.0 Hz, 1H, $H_{4'}$, C_6H_3Cl). $^{13}C\{^1H\}$ NMR (75.47 MHz, $CDCl_3$): δ = 166.2 (s, C=N), 166.1 (q, $^2J_{CF}$ = 39.0 Hz, C, <u>C</u>O_2CF_3), 159.2 (s, C=O), 149.7 (s, CH, =$C_β$), 136.1 (s, C, C_6H_3Cl), 135.3 (s, C, C_6H_3Cl), 133.5 (s, C, C_{ipso}, C_6H_5), 132.6 (s, CH, C_p, C_6H_5), 132.2 (s, CH, C_6H_3Cl), 130.9 (s, CH, =C_{vin}), 130.3 (s, CH, C_6H_3Cl), 129.5 (s, CH, C_o, C_6H_5), 129.2 (s, CH, C_m, C_6H_5), 127.7 (s, CH, C_6H_3Cl), 127.1 (s, C, C_6H_3Cl), 123.5 (s, =C), 115.1 (q, $^1J_{CF}$ = 287.6 Hz, C, CF_3), 109.9 (s, CH, =$C_α$). ^{19}F NMR (282.40 MHz, $CDCl_3$): δ = -74.69 (s, CF_3). HRMS (ESI+) [*m/z*]: $C_{38}H_{28}Cl_2N_2NaO_6Pd_2$ [M-2COOCF$_3$+ 2CH$_3$O+Na]$^+$: 914.9296. Exp: 914.9289. IR (ν, cm^{-1}): 1803 (νC=O), 1645 (νC=N), 1192 (νCF_3).

Characterization of *ortho*-palladated complex 3d

Following the general method, oxazolone **2d** (200 mg, 0.646 mmol) was reacted with $Pd(OAc)_2$ (145 mg, 0.646 mmol) in CF_3CO_2H (8 mL) to give **3d** as a reddish solid. Obtained: 291 mg, 0.275 mmol, 85% yield. ^1H NMR (300.13 MHz, $CDCl_3$): δ = 7.57 (m, 2H, H_o, C_6H_5),

7.52 (d, J = 16.1 Hz, 1H, H_β), 7.49 – 7.39 (m, 3H, H_m, H_p, C_6H_5), 7.23 (s, 1H, H_{vin}), 7.12 (dd, J = 8.1, 1.8 Hz, 1H, $H_{3'}$, C_6H_3Cl), 7.07 (d, J = 8.1 Hz, 1H, $H_{2'}$, C_6H_3Cl), 7.04 (d, J = 15.8 Hz, 1H, H_α), 7.03 (d, J = 1.7 Hz, 1H, $H_{5'}$, C_6H_3Cl). $^{13}C\{^1H\}$ NMR (75.47 MHz, $CDCl_3$): δ = 166.2 (s, C=N), 166.2 (q, $^2J_{CF}$ = 39.2 Hz, C, $\underline{C}O_2CF_3$), 160.1 (s, C=O), 149.7 (s, CH, =C_β), 136.6 (s, C, C_6H_3Cl), 134.9 (s, CH, =C_{vin}), 134.8 (s, C, C_6H_3Cl), 133.6 (s, C, C_{ipso}, C_6H_5), 133.0 (s, CH, C_6H_3Cl), 133.0 (s, CH, C_6H_3Cl), 132.5 (s, CH, C_p, C_6H_5), 129.4 (s, CH, C_o, C_6H_5), 129.2 (s, CH, C_m, C_6H_5), 127.9 (s, C, C_6H_3Cl), 126.6 (s, CH, C_6H_3Cl), 122.7 (s, =C), 115.1 (q, $^1J_{CF}$ = 287.8 Hz, C, CF_3), 109.3 (s, CH, =C_α). ^{19}F NMR (282.40 MHz, $CDCl_3$): δ = -74.82 (s, CF_3). HRMS (ESI+) [m/z]: $C_{38}H_{28}Cl_2N_2NaO_6Pd_2$ $[M\text{-}2COOCF_3\text{+}2CH_3O\text{+}Na]^+$: 914.9296. Exp: 914.9282. IR (v, cm^{-1}): 1799 (vC=O), 1647 (vC=N), 1191 (vCF_3).

Characterization of *ortho*-palladated complex 3e

Following the general method, oxazolone **2e** (109 mg, 0.318 mmol) was reacted with $Pd(OAc)_2$ (71.4 mg, 0.318 mmol) in CF_3CO_2H (6 mL) to give **3e** as a reddish solid. Obtained: 125 mg, 0.112 mmol, 70% yield. ^1H NMR (300.13 MHz, $CDCl_3$): δ = 7.66 – 7.55 (m, 4H, H_{vin}, H_β, H_o), 7.52 – 7.40 (m, 5H, $H_{3'}$, $H_{5'}$, $C_6H_3CF_3$, H_m, H_p, C_6H_5), 7.05 (d, J = 15.9 Hz, 1H, H_α), 7.04 (t, J = 7.9 Hz, 1H, $H_{4'}$, $C_6H_3CF_3$). $^{13}C\{^1H\}$ NMR (75.47 MHz, $CDCl_3$): δ = 167.1 (s, C=N), 158.9 (s, C=O), 150.6 (s, CH, =C_β), 137.4 (s, CH, $C_6H_3CF_3$), 136.2 (s, C, $C_6H_3CF_3$), 133.5 (s, C, C_{ipso}, C_6H_5), 133.0 (s, CH, C_p, C_6H_5), 129.8 (s, CH, C_o, C_6H_5), 129.4 (s, CH, C_m, C_6H_5), 129.3 (s, CH, =C_{vin}), 129.0 (s, CH, $C_6H_3CF_3$), 126.6 (s, C, $C_6H_3CF_3$), 124.6 (q, $^3J_{CF}$ = 5.7 Hz, CH, $C_6H_3CF_3$), 124.1 (s, =C), 124.0 (q, $^1J_{CF}$ = 274.7 Hz, aryl-CF_3), 109.9 (s, CH, =C_α). Peaks due to the quaternary C nuclei of the $\underline{C}O_2\underline{C}F_3$ ligand and $C_6H_3CF_3$ fragment were not observed, probably due to low solubility, despite the use of long accumulation times. ^{19}F NMR (282.40 MHz, $CDCl_3$): δ = -57.06 (s, aryl-CF_3), -74.63 (s, bridging CO_2CF_3). HRMS (ESI+) [m/z]: $C_{40}H_{28}F_6N_2O_6Pd_2$ $[M\text{-}2COOCF_3\text{+}2CH_3O]^+$: 959.9925. Exp: 959.9935. IR (v, cm^{-1}): 1806 (vC=O), 1649 (vC=N), 1200 (vCF_3), 1111 (vCF_3).

Characterization of *ortho*-palladated complex 3f

Following the general method, oxazolone **2f** (200 mg, 0.583 mmol) was reacted with $Pd(OAc)_2$ (131 mg, 0.583 mmol) in CF_3CO_2H (8mL) to give **3f** as a reddish solid. Obtained: 277 mg, 0.247 mmol, 85% yield. ^1H NMR (300.13 MHz, $CDCl_3$): δ = 7.56 (m, 2H, H_o, C_6H_5), 7.52 (d, J = 16.2 Hz, 1H, H_β), 7.47 (m, 3H, H_m, H_p, C_6H_5), 7.40 – 7.35 (m, 2H, $H_{3'}$, $H_{5'}$, $C_6H_3CF_3$), 7.32 – 7.27 (m, 2H, $H_{2'}$, $C_6H_3CF_3$, H_{vin}), 7.05 (d, J = 15.8 Hz, 1H, H_α). $^{13}C\{^1H\}$ NMR (75.47 MHz, $CDCl_3$): δ = 167.2 (s, C=N), 166.4 (q, $^2J_{CF}$ = 39.1 Hz, C, $\underline{C}O_2CF_3$), 160.0 (s, C=O), 151.1 (s, CH, =C_β), 134.1 (s, CH, =C_{vin}), 133.7 (s, C, $C_6H_3CF_3$), 133.5 (s, C, C_{ipso}, C_6H_5), 133.1 (s, CH, C_p, C_6H_5), 132.4 (s, CH, $C_6H_3CF_3$), 132.3 (q, $^4J_{CF}$ = 1.4 Hz, C, $C_6H_3CF_3$),

131.2 (q, $^2J_{CF}$ = 32.1 Hz, C, $C_6H_3CF_3$) 130.1 (q, $^3J_{CF}$ = 3.6 Hz, CH, $C_6H_3CF_3$), 129.7 (s, CH, C_o, C_6H_5), 129.4 (s, CH, C_m, C_6H_5), 124.3 (s, =C), 123.1 (q, $^3J_{CF}$ = 3.6 Hz, CH, $C_6H_3CF_3$), 123.1 (q, $^1J_{CF}$ = 273.5 Hz, C, aryl-$\underline{C}F_3$), 115.1 (q, $^1J_{CF}$ = 284.6 Hz, C, bridging $CO_2\underline{C}F_3$), 109.5 (s, CH, =C_α). ^{19}F NMR (282.40 MHz, $CDCl_3$): δ = -63.49 (s, aryl-CF_3), -74.92 (s, bridging CO_2CF_3). Anal. Calc for $C_{42}H_{22}F_{12}N_2O_8Pd_2$: C, 44.90; H, 1.97; N, 2.49; found: C, 44.78; H, 1.80; N, 2.31. IR (ν, cm^{-1}): 1798 (νC=O), 1650 (νC=N), 1197 (νCF_3), 1128 (νCF_3).

Characterization of *ortho*-palladated complex 3h (large scale synthesis)

Following the general method, oxazolone **2h** (1490 mg, 4.344 mmol) was reacted with $Pd(OAc)_2$ (975 mg, 0.683 mmol) in CF_3CO_2H (25 mL) to give **3h** as a reddish solid. Obtained: 2307 mg, 2.05 mmol, 94% yield. ^{1}H NMR (300.13 MHz, $CDCl_3$): δ = 7.59 (m, 2H, H_o, C_6H_5), 7.52 – 7.42 (m, 4H, H_β, H_m, H_p, C_6H_5), 7.24 (s, 1H, $H_{2'}$, $C_6H_2Cl_2$), 7.18 (s, 1H, H_{vin}), 7.10 (s, 1H, $H_{5'}$, $C_6H_2Cl_2$), 7.01 (d, J = 15.9 Hz, 1H, H_α). $^{13}C\{^1H\}$ NMR (75.47 MHz, $CDCl_3$): δ = 166.9 (s, C=N), 166.5 (q, $^2J_{CF}$ = 38.9 Hz, C, $\underline{C}O_2CF_3$), 159.7 (s, C=O), 150.8 (s, CH, =C_β), 134.5 (s, CH, $C_6H_2Cl_2$), 134.1 (s, C, $C_6H_2Cl_2$), 133.4 (s, C, C_{ipso}, C_6H_5), 133.1 (s, CH, =C_{vin}), 132.9 (s, CH, C_p, C_6H_5), 131.9 (s, CH, $C_6H_2Cl_2$), 131.0 (s, C, $C_6H_2Cl_2$), 130.7 (s, C, $C_6H_2Cl_2$), 129.6 (s, CH, C_o, C_6H_5), 129.5 (s, C, $C_6H_2Cl_2$), 129.2 (s, CH, C_m, C_6H_5), 123.9 (s, =C), 114.9 (q, $^1J_{CF}$ = 288.0 Hz, C, $CO_2\underline{C}F_3$), 109.2 (s, CH, =C_α). ^{19}F NMR (282.40 MHz, $CDCl_3$): δ = -74.78 (s, CF_3). Anal. Calc for $C_{40}H_{20}Cl_4F_6N_2O_8Pd_2$: C, 42.70; H, 1.79; N, 2.49; found: C, 42.58; H, 1.67; N, 2.31. IR (ν, cm^{-1}): 1801 (νC=O), 1650 (νC=N), 1192 (νCF_3).

Characterization of *ortho*-palladated complex 3i

Following the general method, oxazolone **2i** (120 mg, 0.375 mmol) was reacted with $Pd(OAc)_2$ (84 mg, 0.375 mmol) in CF_3CO_2H (6 mL) to give **3i** as a red solid. Obtained: 134 mg, 0.124 mmol, 66% yield. ^{1}H NMR (300.13 MHz, $CDCl_3$): δ = 7.90 (s, 1H, H_{vin}), 7.68 (d, J = 15.9 Hz, 1H, H_β), 7.62 – 7.57 (m, 3H, $H_{3'}$, $C_6H_3NO_2$, H_o, C_6H_5), 7.53 – 7.41 (m, 4H, $H_{5'}$, $C_6H_3NO_2$, H_m, H_p, C_6H_5), 7.09 (t, J = 8.0 Hz, 1H, $H_{4'}$, $C_6H_3NO_2$), 7.06 (d, J = 15.9 Hz, 1H, H_α). $^{13}C\{^1H\}$ NMR (75.47 MHz, $CDCl_3$): δ = 167.3 (s, C=N), 166.4 (q, $^2J_{CF}$ = 38.1 Hz, C, $\underline{C}O_2CF_3$), 158.4 (s, C=O), 151.3 (s, CH, =C_β), 149.1 (s, C-N, $C_6H_3NO_2$), 137.5 (s, CH, $C_6H_3NO_2$), 135.5 (s, C, $C_6H_3NO_2$), 133.3 (s, C, C_{ipso}, C_6H_5), 133.2 (s, CH, C_p, C_6H_5), 129.7 (s, CH, C_o, C_6H_5), 129.3 (s, CH, C_m, C_6H_5), 129.1 (s, CH, $C_6H_3NO_2$), 127.6 (s, CH, =C_{vin}), 124.9 (s, =C), 122.5 (s, CH, $C_6H_3NO_2$), 122.1 (s, C, $C_6H_3NO_2$), 115.0 (q, $^1J_{CF}$ = 287.6 Hz, C, CF_3), 109.4 (s, CH, =C_α). ^{19}F NMR (282.40 MHz, $CDCl_3$): δ = -74.57 (s, CF_3). Anal. Calc for $C_{40}H_{22}F_6N_4O_{12}Pd_2$: C, 44.59; H, 2.06; N, 5.20; found: C, 44.27; H, 1.76; N, 4.99. IR (ν, cm^{-1}): 1807 (νC=O), 1650 (νC=N), 1199 (νCF_3).

Characterization of *ortho*-palladated complex 3j

Following the general method, oxazolone **2j** (200 mg, 0.625 mmol) was reacted with $Pd(OAc)_2$ (140 mg, 0.625 mmol) in CF_3CO_2H (6 mL) to give **3j** as a reddish solid. Obtained: 287 mg, 0.266 mmol, 85% yield. ^1H NMR (300.13 MHz, $CDCl_3$): δ = 8.00 – 7.93 (m, 2H, $H_{3'}$, $H_{5'}$, $C_6H_3NO_2$), 7.60 – 7.50 (m, 4H, $H_β$, H_m, H_p, C_6H_5), 7.49 – 7.43 (m, 2H, H_o, C_6H_5), 7.34 (d, J = 7.6 Hz, 1H, $H_{2'}$, $C_6H_3NO_2$), 7.33 (s, 1H, H_{vin}), 7.03 (d, J = 15.9 Hz, 1H, $H_α$). $^{13}C\{^1H\}$ NMR (75.47 MHz, $CDCl_3$): δ = 167.6 (s, C=N), 159.4 (s, C=O), 152.0 (s, CH, =$C_β$), 146.5 (s, C-N, $C_6H_3NO_2$), 134.5 (s, C, $C_6H_3NO_2$), 133.9 (s, C, $C_6H_3NO_2$), 133.5 (s, CH, C_p, C_6H_5), 133.1 (s, C, C_{ipso}, C_6H_5), 132.6 (s, CH, =C_{vin}), 132.0 (s, CH, $C_6H_3NO_2$), 129.8 (CH, C_m, C_6H_5), 129.4 (s, CH, C_o, C_6H_5), 127.8 (s, CH, $C_6H_3NO_2$), 125.3 (s, =C), 121.0 (s, CH, $C_6H_3NO_2$), 109.0 (s, CH, =$C_α$). Peaks due to the quaternary C nuclei of the $\underline{C}O_2\underline{C}F_3$ ligand were not observed, probably due to low solubility, despite the use of long accumulation times. ^{19}F NMR (282.40 MHz, $CDCl_3$): δ = -74.57 (s, CF_3).Anal. Calc for $C_{40}H_{22}F_6N_4O_{12}Pd_2$: C, 44.59; H, 2.06; N, 5.20; found: C, 44.67; H, 1.88; N, 5.03. IR (v, cm^{-1}): 1822 (vC=O), 1641 (vC=N), 1195 (vCF_3).

Synthesis and characterization of complexes 4a–f and 4h–j

Characterization of cyclobutane *ortho*-palladated complex 4a

Following the general method, orthopalladated **3a** (250 mg, 0.244 mmol) in CH_2Cl_2 (20 mL) was irradiated with blue light (465 nm) for 48 h to give orthopalladated cyclobutane **4a** as a yellow solid. Obtained: 225 mg, 0.220 mmol, 90% yield. ^1H NMR (300.13 MHz, $CDCl_3$): δ = 7.76 (d, J = 16.1 Hz, 1H, $H_β$), 7.67 (m, 2H, H_o, C_6H_5), 7.55 (d, J = 16.1 Hz, 1H, $H_α$), 7.54 – 7.45 (m, 3H, H_m, H_p, C_6H_5), 6.97 – 6.92 (m, 2H, $H_{4'}$, $H_{5'}$, C_6H_3F), 6.76 (ddd, J = 9.2, 5.7, 3.4 Hz, 1H, $H_{3'}$, C_6H_3F), 5.51 (s, 1H, CH cyclobutane). $^{13}C\{^1H\}$ NMR (75.47 MHz, $CDCl_3$): δ = 171.4 (s, C=O), 169.1 (s, C=N), 167.1 (q, $^2J_{CF}$ = 39.1 Hz, C, $\underline{C}O_2CF_3$), 158.2 (d, $^1J_{CF}$ = 250.5 Hz, C-F, C_6H_3F), 151.2 (s, CH, =$C_β$), 138.0 (d, $^3J_{CF}$ = 1,6 Hz, C, C_6H_3F), 133.3 (s, C, C_{ipso}, C_6H_5), 132.9 (s, CH, C_p, C_6H_5), 129.6 (s, CH, C_m, C_6H_5), 129.5 (s, CH, C_o, C_6H_5), 129.4 (d, $^4J_{CF}$ = 3.5 Hz, CH, C_6H_3F), 129.3 (d, $^3J_{CF}$ = 8.1 Hz, CH, C_6H_3F), 115.3 (q, $^1J_{CF}$ = 287.5 Hz, C, CF_3), 115.2 (d, $^2J_{CF}$ = 11.8 Hz, C, C_6H_3F), 112.1 (d, $^2J_{CF}$ = 22.9 Hz, CH, C_6H_3F), 111.0 (s, CH, =$C_α$), 67.3 (s, Cq, cyclobutane), 51.2 (d, $^3J_{CF}$ = 3.7 Hz, CH, cyclobutane). ^{19}F NMR (282.40 MHz, $CDCl_3$): δ = -74.65 (s, 3F, CF_3), -113.38 (m, 1F, F_{ar}). Anal. Calc for $C_{40}H_{22}F_8N_2O_8Pd_2$: C, 46.94; H, 2.17; N, 2.74; found: C, 47.19; H, 2.02; N, 2.55. IR (v, cm^{-1}): 1845 (vC=O), 1651 (vC=N), 1192 (vCF_3).

Characterization of cyclobutane *ortho*-palladated complex 4c

Following the general method, orthopalladated **3c** (250 mg, 0.237 mmol) in CH_2Cl_2 (20 mL) was irradiated with blue light (465 nm) for 48 h to give orthopalladated cyclobutane **4c** as a yellow solid. Obtained: 200 mg, 0.189 mmol, 80% yield. ^1H NMR (300.13 MHz, $CDCl_3$): δ = 7.78 (d, *J* = 16.1 Hz, 1H, H_β), 7.68 (m, 2H, H_o, C_6H_5), 7.51 (d, *J* = 16.1 Hz, 1H, H_α), 7.52 – 7.46 (m, 3H, H_m, H_p, C_6H_5), 7.08 (dd, *J* = 7.9, 1.5 Hz, 1H, $H_{3'}$, C_6H_3Cl), 7.07 (dd, *J* = 7.9, 1.5 Hz, 1H, $H_{5'}$, C_6H_3Cl), 6.87 (t, 1H, $H_{4'}$, C_6H_3Cl), 5.70 (s, 1H, cyclobutane). $^{13}C\{^1H\}$ NMR (75.47 MHz, $CDCl_3$): δ = 171.3 (s, C=O), 169.1 (s, C=N), 151.1 (s, CH, $=C_\beta$), 138.5 (s, C, C_6H_3Cl), 133.3 (s, C, C_{ipso}, C_6H_5), 132.9 (s, CH, C_p, C_6H_5), 132.6 (s, CH, C_6H_3Cl), 132.4 (s, C, C_6H_3Cl), 129.6 (s, CH, C_m, C_6H_5), 129.5 (s, CH, C_o, C_6H_5), 127.8 (s, CH, C_6H_3Cl), 126.7 (s, CH, C_6H_3Cl), 125.6 (s, C, C_6H_3Cl), 110.9 (s, CH, $=C_\alpha$), 67.7 (s, C, cyclobutane), 55.2 (s, CH, cyclobutane). Peaks due to the quaternary C nuclei of the $\underline{C}O_2\underline{C}F_3$ ligand were not observed, probably due to low solubility, despite the use of long accumulation times. ^{19}F NMR (282.40 MHz, $CDCl_3$): δ = -74.69 (s, CF_3). Anal. Calc for $C_{40}H_{22}Cl_2F_6N_2O_8Pd_2$: C, 45.48; H, 2.10; N, 2.65; found: C, 45.22; H, 1.89; N, 2.46. IR (ν, cm^{-1}): 1844 (νC=O), 1654 (νC=N), 1197 (νCF_3).

Characterization of cyclobutane *ortho*-palladated complex 4d

Following the general method, orthopalladated **3d** (250 mg, 0.237 mmol) in CH_2Cl_2 (20 mL) was irradiated with blue light (465 nm) for 48 h to give orthopalladated cyclobutane **4d** as a yellow solid. Obtained: 170 mg, 0.161 mmol, 68% yield. ^1H NMR (300.13 MHz, $CDCl_3$): δ = 7.74 (d, *J* = 16.1 Hz, 1H, H_β), 7.67 (m, 2H, H_o, C_6H_5), 7.57 (d, *J* = 16.1 Hz, 1H, H_α), 7.56 – 7.44 (m, 3H, H_m, H_p, C_6H_5), 7.15 (d, *J* = 2.0 Hz, 1H, $H_{5'}$, C_6H_3Cl), 6.99 (dd, *J* = 8.0, 2.1 Hz, 1H, $H_{3'}$, C_6H_3Cl), 6.73 (d, *J* = 8.1 Hz, 1H, $H_{2'}$, C_6H_3Cl), 4.94 (s, 1H, H_6). $^{13}C\{^1H\}$ NMR (75.47 MHz, $CDCl_3$): δ = 172.1 (s, C=O), 169.0 (s, C=N), 167.1 (q, $^2J_{CF}$ = 38.6 Hz, C, $\underline{C}O_2CF_3$), 151.4 (s, CH, $=C_\beta$), 136.6 (s, C, C_6H_3Cl), 133.4 (s, CH, C_6H_3Cl), 133.3 (s, C, C_6H_3Cl), 133.2 (s, C, C_{ipso}, C_6H_5), 133.0 (s, CH, C_p, C_6H_5), 129.8 (s, CH, C_6H_3Cl), 129.6 (s, CH, C_m, C_6H_5), 129.5 (s, CH, C_o, C_6H_5), 126.3 (s, CH, C_6H_3Cl), 126.0 (s, C, C_6H_3Cl), 115.1 (q, $^1J_{CF}$ = 290.7 Hz, C, CF_3), 110.8 (s, CH, C_α), 68.5 (s, C, cyclobutane), 59.5 (s, CH, cyclobutane). ^{19}F NMR (282.40 MHz, $CDCl_3$): δ = -74.66 (s, CF_3). Anal. Calc for $C_{40}H_{22}Cl_2F_6N_2O_8Pd_2$: C, 45.48; H, 2.10; N, 2.65; found: C, 45.73; H, 1.94; N, 2.33. IR (ν, cm^{-1}): 1837 (νC=O), 1652 (νC=N), 1194 (νCF_3).

Characterization of cyclobutane *ortho*-palladated complex 4e

Following the general method, orthopalladated **3e** (100 mg, 0.089 mmol) in CH_2Cl_2 (20 mL) was irradiated with blue light (465 nm) for 48 h to give orthopalladated cyclobutane **4e** as a yellow solid. Obtained: 60 mg, 0.053 mmol, 60% yield. ^1H NMR (400.13 MHz, $CDCl_3$): δ =

7.78 (d, J = 16.0 Hz, 1H, H_β), 7.67 (m, 2H, H_o, C_6H_5), 7.51 (d, J = 15.9 Hz, 1H, H_α), 7.54 – 7.44 (m, 4H, $H_{5'}$, $C_6H_3CF_3$, H_m, H_p, C_6H_5), 7.39 (d, J = 7.5 Hz, 1H, $H_{3'}$, $C_6H_3CF_3$), 7.07 (t, J = 7.8 Hz, 1H, $H_{4'}$, $C_6H_3CF_3$), 5.46 (s, 1H, cyclobutane). $^{13}C\{^1H\}$ NMR (75.47 MHz, $CDCl_3$): δ = 171.0 (s, C=O), 169.7 (s, C=N), 167.1 (q, $^2J_{CF}$ = 39.3 Hz, C, $\underline{C}O_2CF_3$), 151.5 (s, CH, =C_β), 140.0 (s, C, $C_6H_3CF_3$), 138.1 (s, CH, $C_6H_3CF_3$), 133.2 (s, C, C_{ipso}, C_6H_5), 133.0 (s, CH, C_p, C_6H_5), 129.6 (s, CH, C_m, C_6H_5), 129.6 (s, CH, C_o, C_6H_5), 126.8 (q, $^1J_{CF}$ = 290.1 Hz, C, aryl-CF_3) 126.8 (s, CH, $C_6H_3CF_3$), 125.1 (q, $^2J_{CF}$ = 34.3 Hz, C, $C_6H_3CF_3$), 124.0 (q, $^3J_{CF}$ = 5.8 Hz, CH, $C_6H_3CF_3$), 121.7 (q, $^3J_{CF}$ = 2.9 Hz, C, $C_6H_3CF_3$), 115.2 (q, $^1J_{CF}$ = 289.3 Hz, C, bridging $CO_2\underline{C}F_3$), 109.5 (s, CH, =C_α), 68.6 (s, C, cyclobutane), 53.5 (s, CH, cyclobutane). ^{19}F NMR (376.50 MHz, $CDCl_3$): δ = -58.2 (s, aryl-CF_3), -74.6 (s, bridging CF_3). Anal. Calc for $C_{42}H_{22}F_{12}N_2O_8Pd_2$: C, 44.90; H, 1.97; N, 2.49; found: C, 45.18; H, 2.01; N, 2.70. IR (ν, cm^{-1}): 1851 (νC=O), 1653 (νC=N), 1198 (νCF_3), 1110 (CF_3).

Characterization of cyclobutane *ortho*-palladated complex 4f

Following the general method, orthopalladated **3f** (200 mg, 0.178 mmol) in CH_2Cl_2 (20 mL) was irradiated with blue light (465 nm) for 48 h to give orthopalladated cyclobutane **4f** as a yellow solid. Obtained: 142 mg, 0.126 mmol, 71% yield. ^1H NMR (400.13 MHz, $CDCl_3$): δ = 7.76 (d, J = 16.0 Hz, 1H, H_β), 7.67 (m, 2H, H_o, C_6H_5), 7.56 (d, J = 16.1 Hz, 1H, H_α), 7.55 – 7.44 (m, 4H, $H_{5'}$, $C_6H_3CF_3$, H_m, H_p, C_6H_5), 7.26 (dd, J = 7.8, 1.3 Hz, 1H, $H_{3'}$, $C_6H_3CF_3$), 6.93 (d, J = 7.8 Hz, 1H, $H_{2'}$, $C_6H_3CF_3$), 5.04 (s, 1H, cyclobutane). $^{13}C\{^1H\}$ NMR (75.47 MHz, $CDCl_3$): δ = 171.9 (s, C=O), 169.3 (s, C=N), 167.3 (q, $^2J_{CF}$ = 39.1 Hz, C, $\underline{C}O_2CF_3$), 151.8 (s, CH, =C_β), 136.1 (s, C, $C_6H_3CF_3$), 133.2 (s, CH, C_p, C_6H_5), 133.1 (s, C, C_{ipso}, C_6H_5), 131.3 (q, $^4J_{CF}$ = 1.2 Hz, C, $C_6H_3CF_3$), 130.8 (q, $^3J_{CF}$ = 3.8 Hz, CH, $C_6H_3CF_3$), 129.9 (q, $^2J_{CF}$ = 32.3 Hz, C, $C_6H_3CF_3$), 129.7 (s, CH, C_m, C_6H_5), 129.7 (s, CH, C_o, C_6H_5), 129.2 (s, CH, $C_6H_3CF_3$), 123.4 (q, $^1J_{CF}$ = 274.8 Hz, C, aryl-CF_3), 122.9 (q, $^3J_{CF}$ = 3.8 Hz, CH, $C_6H_3CF_3$), 115.1 (q, $^1J_{CF}$ = 289.3 Hz, C, $CO_2\underline{C}F_3$), 110.7 (s, CH, =C_α), 68.0 (s, C, cyclobutane), 59.7 (s, CH, cyclobutane). ^{19}F NMR (282.40 MHz, $CDCl_3$): δ = -63.2 (s, aryl-CF_3), -74.8 (s, bridging CF_3). Anal. Calc for $C_{42}H_{22}F_{12}N_2O_8Pd_2$: C, 44.90; H, 1.97; N, 2.49; found: C, 44.73; H, 1.79; N, 2.35. IR (ν, cm^{-1}): 1839 (νC=O), 1651 (νC=N), 1196 (νCF_3), 1114 (νCF_3).

Characterization of cyclobutane *ortho*-palladated complex 4h (large scale synthesis)

Following the general method, orthopalladated **3h** (2295 mg, 2.039 mmol) in CH_2Cl_2 (120 mL) was irradiated with blue light (465 nm) for 48 h to give orthopalladated cyclobutane **4h** as a yellow solid. Obtained: 2196 mg, 1.951 mmol, 96% yield. ^1H NMR (400.13 MHz, $CDCl_3$): δ = 7.79 (d, J = 16.0 Hz, 1H, H_β), 7.68 (m, 2H, H_o, C_6H_5), 7.55 (d, J = 16.0 Hz, 1H, H_α), 7.59 – 7.45 (m, 3H, H_m, H_p, C_6H_5), 7.23 (s, 1H, $H_{5'}$, $C_6H_2Cl_2$), 6.90 (s, 1H, $H_{2'}$, $C_6H_2Cl_2$), 4.87 (s,

1H, cyclobutane). $^{13}C\{^1H\}$ NMR (75.47 MHz, $CDCl_3$): δ = 171.6 (s, C=O), 169.4 (s, C=N), 167.3 (q, $^2J_{CF}$ = 38.6 Hz, C, $\underline{C}O_2CF_3$), 152.2 (s, CH, =$C_β$), 134.9 (s, CH, $C_6H_2Cl_2$), 133.8 (s, C, $C_6H_2Cl_2$), 133.3 (s, CH, C_p, C_6H_5), 133.2 (s, C, $C_6H_2Cl_2$), 131.6 (s, C, C_{ipso}, C_6H_5), 130.2 (s, C, $C_6H_2Cl_2$), 129.8 (s, CH, $C_6H_2Cl_2$), 129.7 (s, CH, C_m, C_6H_5), 129.6 (s, CH, C_o, C_6H_5), 127.3 (s, C, $C_6H_2Cl_2$), 115.0 (q, $^1J_{CF}$ = 285.2 Hz, C, $CO_2\underline{C}F_3$), 110.6 (s, CH, =$C_α$), 68.3 (s, C, cyclobutane), 58.9 (s, CH, cyclobutane). ^{19}F NMR (282.40 MHz, $CDCl_3$): δ = -74.62 (s, CF_3). Anal. Calc for $C_{40}H_{20}Cl_4F_6N_2O_8Pd_2$: C, 42.70; H, 1.79; N, 2.49; found: C, 42.66; H, 1.71; N, 2.28. IR (ν, cm^{-1}): 1833 (νC=O), 1650 (νC=N), 1196 ($νCF_3$).

Characterization of cyclobutane *ortho*-palladated complex 4i

Following the general method, orthopalladated **3i** (100 mg, 0.093 mmol) in CH_2Cl_2 (20 mL) was irradiated with blue light (465 nm) for 48 h to give orthopalladated cyclobutane **4i** as a yellow solid. Obtained: 69.5 mg, 0.0645 mmol, 69 % yield. ^1H NMR (300.13 MHz, $CDCl_3$): δ = 7.87 (d, J = 16.0 Hz, 1H, $H_β$), 7.69 (m, 2H, H_o, C_6H_5), 7.64 (dd, J = 7.9, 1.1 Hz, 1H, $H_{3'}$, $C_6H_3NO_2$), 7.56 – 7.43 (m, 5H, $H_α$, $H_{5'}$ $C_6H_3NO_2$, H_m, H_p, C_6H_5), 7.12 (t, J = 8.0 Hz, 1H, $H_{4'}$, $C_6H_3NO_2$), 5.10 (s, 1H, cyclobutane). $^{13}C\{^1H\}$ NMR (75.47 MHz, $CDCl_3$): δ = 170.6 (s, C=O), 170.1 (s, C=N), 152.0 (s, CH, =$C_β$), 150.0 (s, C-N, $C_6H_3NO_2$), 140.9 (s, C, $C_6H_3NO_2$), 139.2 (s, CH, $C_6H_3NO_2$), 134.4 (s, C, C_{ipso}, C_6H_5), 133.2 (s, CH, C_p, C_6H_5), 129.7 (s, CH, C_m, C_6H_5), 129.7 (s, CH, C_o, C_6H_5), 127.0 (s, CH, $C_6H_3NO_2$), 122.7 (s, CH, $C_6H_3NO_2$), 122.3 (s, C, $C_6H_3NO_2$), 110.6 (s, CH, =$C_α$), 68.1 (s, C, cyclobutane), 54.4 (s, CH, cyclobutane). Peaks due to the quaternary C nuclei of the $\underline{C}O_2\underline{C}F_3$ ligand were not observed, probably due to low solubility, despite the use of long accumulation times. ^{19}F NMR (282.40 MHz, $CDCl_3$): δ = -74.66 (s, CF_3). Anal. Calc for $C_{40}H_{22}F_6N_4O_{12}Pd_2$: C, 44.59; H, 2.06; N, 5.20; found: C, 44.43; H, 1.84; N, 5.53. IR (ν, cm^{-1}): 1851 (νC=O), 1656 (νC=N), 1199 ($νCF_3$).

Characterization of cyclobutane *ortho*-palladated complex 4j

Following the general method, orthopalladated **3j** (300 mg, 0.278 mmol) in CH_2Cl_2 (40 mL) was irradiated with blue light (465 nm) for 48 h to give orthopalladated cyclobutane **4j** as a yellow solid. Obtained: 244 mg, 0.227 mmol, 81% yield. ^1H NMR (400.13 MHz, $CDCl_3$): δ = 8.04 (d, J = 2.2 Hz, 1H, $H_{5'}$, $C_6H_3NO_2$), 7.84 (dd, J = 8.3, 2.3 Hz, 1H, $H_{3'}$, $C_6H_3NO_2$), 7.78 (d, J = 16.0 Hz, 1H, $H_β$), 7.67 (m, 2H, H_o, C_6H_5), 7.54 (d, J = 15.9 Hz, 1H, $H_α$), 7.57 – 7.45 (m, 3H, H_m, H_p, C_6H_5), 6.99 (d, J = 8.3 Hz, 1H, $H_{2'}$, $C_6H_3NO_2$), 5.08 (s, 1H, cyclobutane). $^{13}C\{^1H\}$ NMR (75.47 MHz, $CDCl_3$): δ = 171.5 (s, C=O), 169.8 (s, C=N), 152.6 (s, CH, =$C_β$), 146.2 (s, C, $C_6H_3NO_2$), 136.8 (s, C, $C_6H_3NO_2$), 134.0 (s, C, $C_6H_3NO_2$), 133.5 (s, CH, C_p, C_6H_5), 133.0 (s, C, C_{ipso}, C_6H_5), 129.7 (s, CH, C_m, C_6H_5), 129.7 (s, CH, C_o, C_6H_5), 129.4 (s, CH, $C_6H_3NO_2$), 128.6 (s, CH, $C_6H_3NO_2$), 121.1 (s, CH, $C_6H_3NO_2$), 110.3 (s, CH, =$C_α$), 67.8

(s, C, cyclobutane), 59.5 (s, CH, cyclobutane). Peaks due to the quaternary C nuclei of the $\underline{C}O_2\underline{C}F_3$ ligand were not observed, probably due to low solubility, despite the use of long accumulation times. ^{19}F NMR (376.50 MHz, $CDCl_3$): δ = -74.51 (s, CF_3). Anal. Calc for $C_{40}H_{22}F_6N_4O_{12}Pd_2$: C, 44.59; H, 2.06; N, 5.20; found: C, 44.31; H, 2.24; N, 5.02. IR (v, cm^{-1}): 1824 (vC=O), 1642 (vC=N), 1195 (vCF_3).

Synthesis and characterization of diaminotruxillic ester derivatives 5

Characterization of dimethyl 2,4-bis(4-chloro-2-(methoxycarbonyl)phenyl)-1,3-dicinnamamidocyclobutane-1,3-dicarboxylate (5d)

Following the general method, a solution of the *ortho*-palladated cyclobutane **4d** (80 mg, 0.076 mmol) in a mixture of 3 mL of methanol and 9 mL of NCMe was stirred under a CO atmosphere (1 atm) for 16 h to give the truxillic cyclobutane derivative **5d** as a pale yellow solid. Obtained: 50 mg, 0.063 mmol, 83% yield. ^{1}H NMR (300.13 MHz, $CDCl_3$): δ = 7.70 (d, *J* = 8.7 Hz, 1H, H_6, C_6H_3Cl), 7.64 (d, *J* = 2.3 Hz, 1H, H_3, C_6H_3Cl), 7.50 (m, 2H, H_o, Ph), 7.42 – 7.34 (m, 6H, NH, H_β, C_6H_3Cl H_5, H_m, H_p Ph), 6.39 (d, *J* = 15.7 Hz, 1H, H_α), 5.98 (s, 1H, cyclobutane), 4.00 (s, 3H, OMe, CO_2Me cyclobutane), 3.87 (s, 3H, OMe, Ar-CO_2Me). ^{13}C{^{1}H} NMR (75.47 MHz, $CDCl_3$): δ = 172.3 (s, C, $\underline{C}O_2Me$ cyclobutane), 167.8 (s, C, Ar-$\underline{C}O_2Me$), 166.2 (s, C, NH$\underline{C}$=O), 142.5 (s, CH, =C_β), 134.5 (s, C, C_{ipso}, Ph), 133.3 (s, C, C_6H_3Cl), 133.2 (s, C, C_6H_3Cl), 132.0 (s, C, C_6H_3Cl), 131.3 (s, CH, C_6H_3Cl), 130.9 (s, CH, C_6H_3Cl), 130.3 (s, CH, C_p, Ph), 130.0 (s, CH, C_6H_3Cl), 129.0 (s, CH, C_m, Ph), 128.2 (s, CH, C_o, Ph), 119.9 (s, CH, =C_α), 65.7 (s, C, cyclobutane), 53.8 (s, CH, OMe, $CO_2\underline{Me}$ cyclobutane), 52.7 (s, CH, OMe, Ar-$CO_2\underline{Me}$), 48.4 (s, CH, cyclobutane). HRMS (ESI+) [*m/z*]: Calc. for $C_{42}H_{36}Cl_2N_2NaO_{10}$ $[M+Na]^+$: 821.1645. Exp.: 821.1658. IR (v, cm^{-1}): 1726 (vCO_2Me).

Characterization of dimethyl 1,3-dicinnamamido-2,4-bis(2-(methoxycarbonyl)-4-(trifluoromethyl)phenyl)cyclobutane-1,3-dicarboxylate (5f)

Following the general method, a solution of the *ortho*-palladated cyclobutane **4f** (100 mg, 0.089 mmol) in a mixture of 4 mL of methanol and 12 mL of NCMe was stirred under a CO atmosphere (1 atm) for 16 h to give the truxillic cyclobutane derivative **5f** as a yellow solid. Obtained: 52 mg, 0.060 mmol, 67% yield. ^{1}H NMR (400.13 MHz, $CDCl_3$): δ = 7.92 (d, *J* = 1.6 Hz, 1H, H_6, $C_6H_3CF_3$), 7.87 (d, *J* = 8.4 Hz, 1H, H_3, $C_6H_3CF_3$), 7.63 (dd, *J* = 8.3, 1.6 Hz, 1H, H_5, $C_6H_3CF_3$), 7.51 – 7.46 (m, 2H, H_o, Ph), 7.41 – 7.36 (m, 4H, NH, H_m, H_p, Ph), 7.35 (d, *J* = 15.9 Hz, 1H, H_β), 6.40 (d, *J* = 15.6 Hz, 1H, H_α), 6.09 (s, 1H, cyclobutane), 4.04 (s, 3H, OMe, CO_2Me cyclobutane), 3.91 (s, 3H, OMe, Ar-CO_2Me). ^{13}C{^{1}H} NMR (75.47 MHz,

$CDCl_3$): δ = 172.1 (s, C, <u>C</u>O_2Me cyclobutane), 167.8 (s, C, Ar-<u>C</u>O_2Me), 166.4 (s, C, NH<u>C</u>=O), 142.9 (s, CH, =$C_β$), 137.4 (s, C, $C_6H_3CF_3$), 134.4 (s, C, C_{ipso}, Ph), 132.3 (s, C, $C_6H_3CF_3$), 130.4 (s, CH, C_p, Ph), 130.2 (s, CH, $C_6H_3CF_3$), 129.4 (q, $^2J_{CF}$ = 33.0 Hz, C, $C_6H_3CF_3$), 129.0 (s, CH, C_m, Ph), 128.2 (s, CH, C_o, Ph), 127.7 (q, $^3J_{CF}$ = 3.9 Hz, CH, $C_6H_3CF_3$), 126.9 (q, $^3J_{CF}$ = 3.9 Hz, CH, $C_6H_3CF_3$), 123.7 (q, $^1J_{CF}$ = 274.1 Hz, C, CF_3), 119.7 (s, CH, =$C_α$), 65.8 (s, C, cyclobutane), 54.0 (s, CH, OMe, CO_2<u>Me</u> cyclobutane), 52.8 (s, CH, OMe, Ar-CO_2<u>Me</u>), 49.0 (s, CH, cyclobutane). ^{19}F NMR (282.40 MHz, $CDCl_3$): δ = -62.78 (s). HRMS (ESI+) [*m/z*]: Calc. for $C_{44}H_{36}F_6N_2NaO_{10}$ $[M+Na]^+$: 889.2172. Exp.: 889.2174. IR (ν, cm^{-1}): 1726 (νCO_2Me), 1125 (νCF_3).

Characterization of dimethyl 1,3-dicinnamamido-2,4-bis(4,5-dichloro-2-(methoxycarbonyl)phenyl)cyclobutane-1,3-dicarboxylate (5h) (large scale synthesis)

Following the general method, a solution of the *ortho*-palladated cyclobutane **4h** (750 mg, 0.665 mmol) in a mixture of 25 mL of methanol and 75 mL of NCMe was stirred under a CO atmosphere (1 atm) for 16 h to give the truxillic cyclobutane derivative **5h** as a pale yellow solid. Compound **5h** was purified by column chromatography using silica and a mixture of hexane/ethyl acetate (7:3) as eluant. Obtained: 528 mg, 0.608 mmol, 91% yield. ^{1}H NMR (400.13 MHz, $CDCl_3$): 7.91 (s, 1H, H_6, $C_6H_2Cl_2$), 7.76 (s, 1H, H_3, $C_6H_2Cl_2$), 7.50 (m, 2H, H_o, Ph), 7.44 (d, J = 15.7 Hz, 1H, $H_β$), 7.39 – 7.36 (m, 4H, NH, H_m, H_p, Ph), 6.41 (d, J = 15.8 Hz, 1H, $H_α$), 5.98 (s, 1H, cyclobutane), 4.01 (s, 3H, OMe, CO_2Me cyclobutane), 3.86 (m, 3H, OMe, Ar-CO_2Me). ^{13}C{^{1}H} NMR (75.47 MHz, $CDCl_3$): δ = 171.9 (s, C, <u>C</u>O_2Me cyclobutane), 167.1 (s, C, NH<u>C</u>=O), 167.0 (s, C, Ar-<u>C</u>O_2Me), 143.4 (s, CH, =$C_β$), 136.1 (s, C, $C_6H_2Cl_2$), 134.4 (s, C, C_{ipso}, Ph), 133.3 (s, C, $C_6H_2Cl_2$), 131.8 (s, CH, $C_6H_2Cl_2$), 131.8 (s, CH, $C_6H_2Cl_2$), 131.7 (s, C, $C_6H_2Cl_2$), 130.9 (s, C, $C_6H_2Cl_2$), 130.4 (s, CH, C_p, Ph), 129.0 (s, CH, C_m, Ph), 128.3 (s, CH, C_o, Ph), 119.4 (s, CH, =$C_α$), 65.7 (s, C, cyclobutane), 54.1 (s, CH, OMe, CO_2<u>Me</u> cyclobutane), 52.9 (s, CH, OMe, Ar-CO_2<u>Me</u>), 48.3 (s, CH, cyclobutane). HRMS (ESI+) [*m/z*]: Calc. for $C_{42}H_{34}Cl_4N_2NaO_{14}$ $[M+Na]^+$:889.0865. Exp.: 889.0856. IR (ν, cm^{-1}): 1723 (νCO_2Me).

Characterization of dimethyl 1,3-dicinnamamido-2,4-bis(2-(methoxycarbonyl)-4-nitrophenyl)-cyclobutane-1,3-dicarboxylate (5j)

Following the general method, a solution of the *ortho*-palladated cyclobutane **4j** (150 mg, 0.139 mmol) in a mixture of 5 mL of methanol and 15 mL of NCMe was stirred under a CO atmosphere (1 atm) for 16 h to give the truxillic cyclobutane derivative **5j** as an orange solid. Compound **5j** was purified by column chromatography using silica and a mixture of

hexane/ethyl acetate (7:3) as eluant. Obtained: 60 mg, 0.074 mmol, 53% yield. ^{1}H NMR (400.13 MHz, $CDCl_3$): δ = 8.51 (d, *J* = 2.5 Hz, 1H, H_3, $C_6H_3NO_2$), 8.22 (dd, *J* = 8.8, 2.6 Hz, 1H, H_5, $C_6H_3NO_2$), 7.94 (d, *J* = 8.8 Hz, 1H, H_6, $C_6H_3NO_2$), 7.48 (m, 2H, H_o, Ph), 7.43 – 7.35 (m, 5H, NH, H_β, H_m, H_p, Ph), 6.35 (d, *J* = 15.6, 1H, H_α), 6.12 (s, 1H, cyclobutane), 4.05 (s, 3H, OMe, CO_2Me cyclobutane), 3.94 (s, 3H, OMe, Ar-CO_2Me). $^{13}C\{^{1}H\}$ NMR (100.61 MHz, $CDCl_3$): δ = 171.8 (s, C, $\underline{C}O_2Me$ cyclobutane), 167.1 (s, C, Ar-$\underline{C}O_2Me$), 166.2 (s, C, NH$\underline{C}$=O), 146.4 (s, C-N, $C_6H_3NO_2$), 143.1 (s, CH, =C_β), 140.6 (s, C, $C_6H_3NO_2$), 134.2 (s, C, C_{ipso}, Ph), 132.9 (s, C, $C_6H_3NO_2$), 130.8 (s, CH, $C_6H_3NO_2$), 130.6 (s, CH, C_p, Ph), 129.1 (s, CH, C_m, Ph), 128.3 (s, CH, C_o, Ph), 125.7 (s, CH, $C_6H_3NO_2$), 124.9 (s, CH, $C_6H_3NO_2$), 119.3 (s, CH, =C_α), 65.7 (s, C, cyclobutane), 53.9 (s, CH, OMe, $CO_2\underline{Me}$ cyclobutane), 53.1 (s, CH, OMe, Ar-$CO_2\underline{Me}$), 49.1 (s, CH, cyclobutane). HRMS (ESI+) [*m/z*]: Calc. for $C_{42}H_{36}N_4NaO_{14}$ $[M+Na]^+$: 843.2126. Exp.: 843.2120. IR (ν, cm^{-1}): 1727 (νCO_2Me), 1187 (νNO_2).

NMR SPECTRA OF ALL NEW COMPOUNDS

1. NMR spectra of (*Z*)-4-arylidene-2-((*E*)-styryl)-5(4*H*)-oxazolones 2a–j

4-((*Z*)-2-Fluorobenzylidene)-2-((*E*)-styryl)oxazol-5(4*H*)-one (2a)

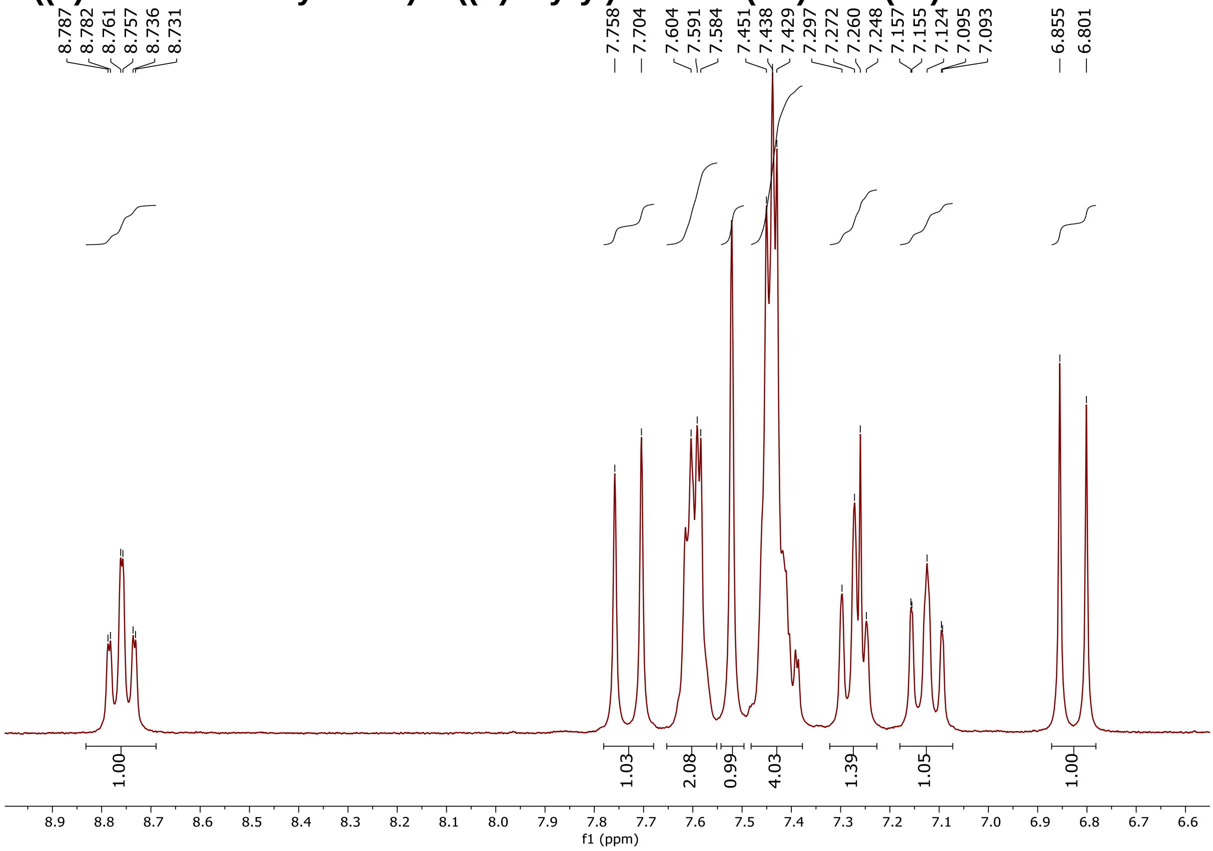

^{1}H NMR ($CDCl_3$, 300.13 MHz) of **2a**

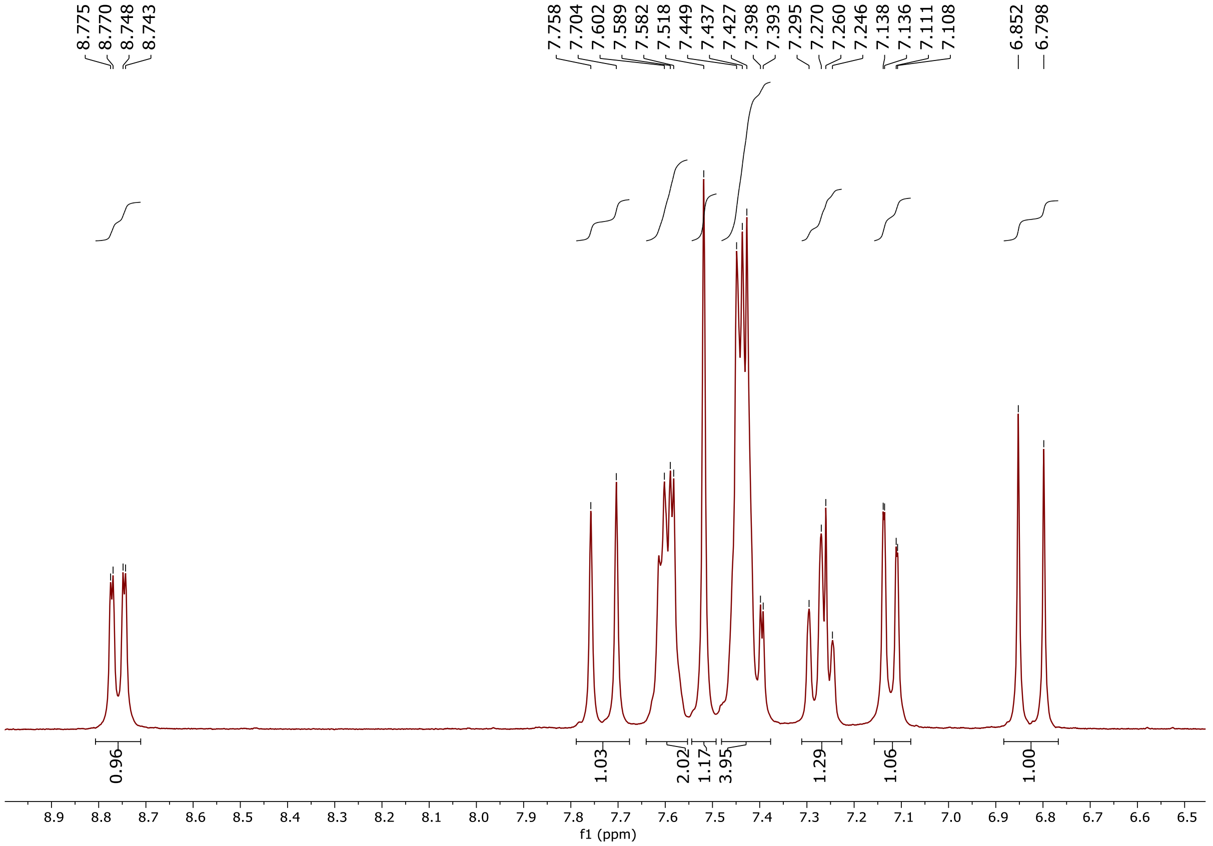

^{1}H{^{19}F}-NMR spectrum ($CDCl_3$, 300.13 MHz) of **2a**

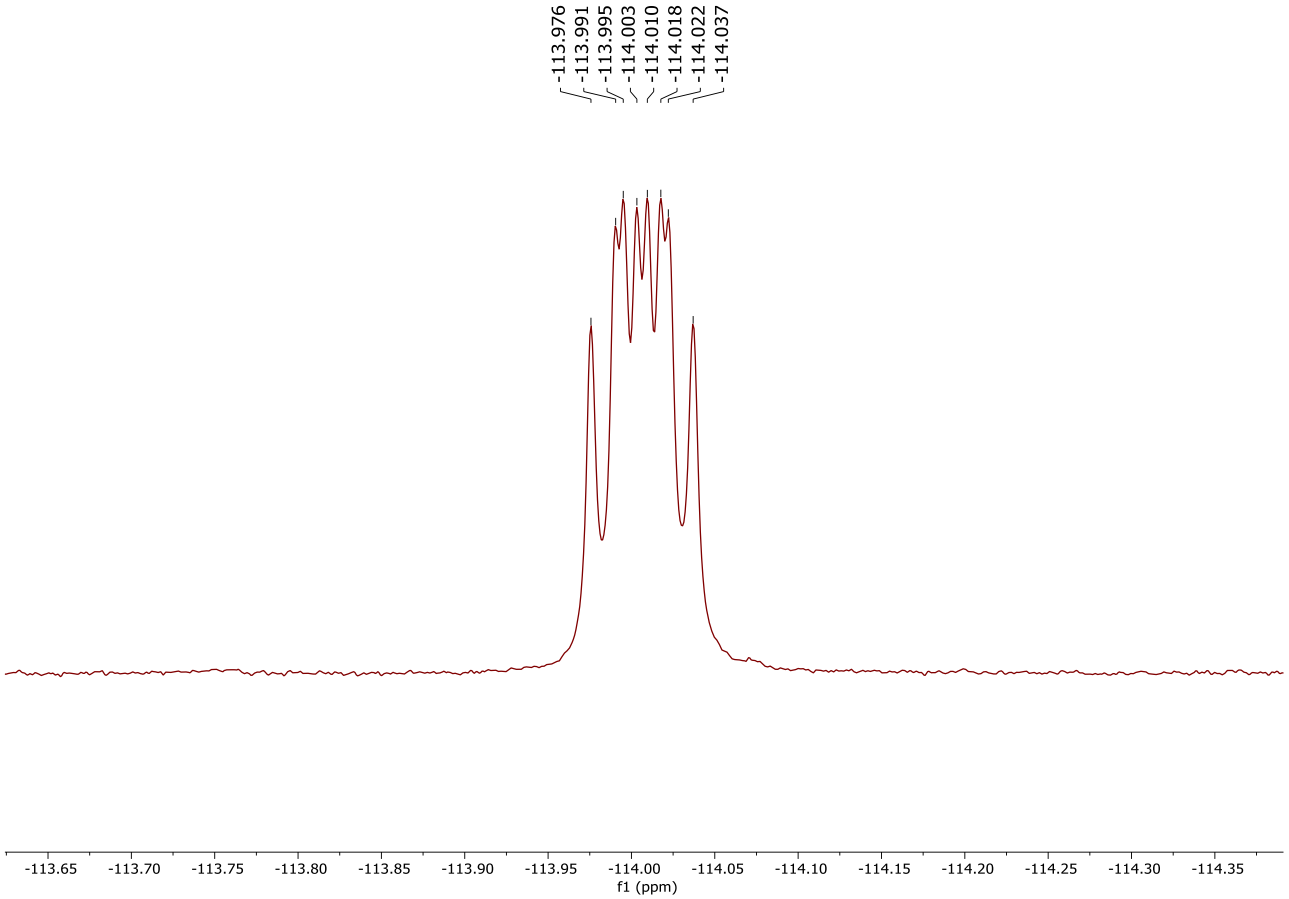

^{19}F-NMR spectrum ($CDCl_3$, 282.40 MHz) of **2a**

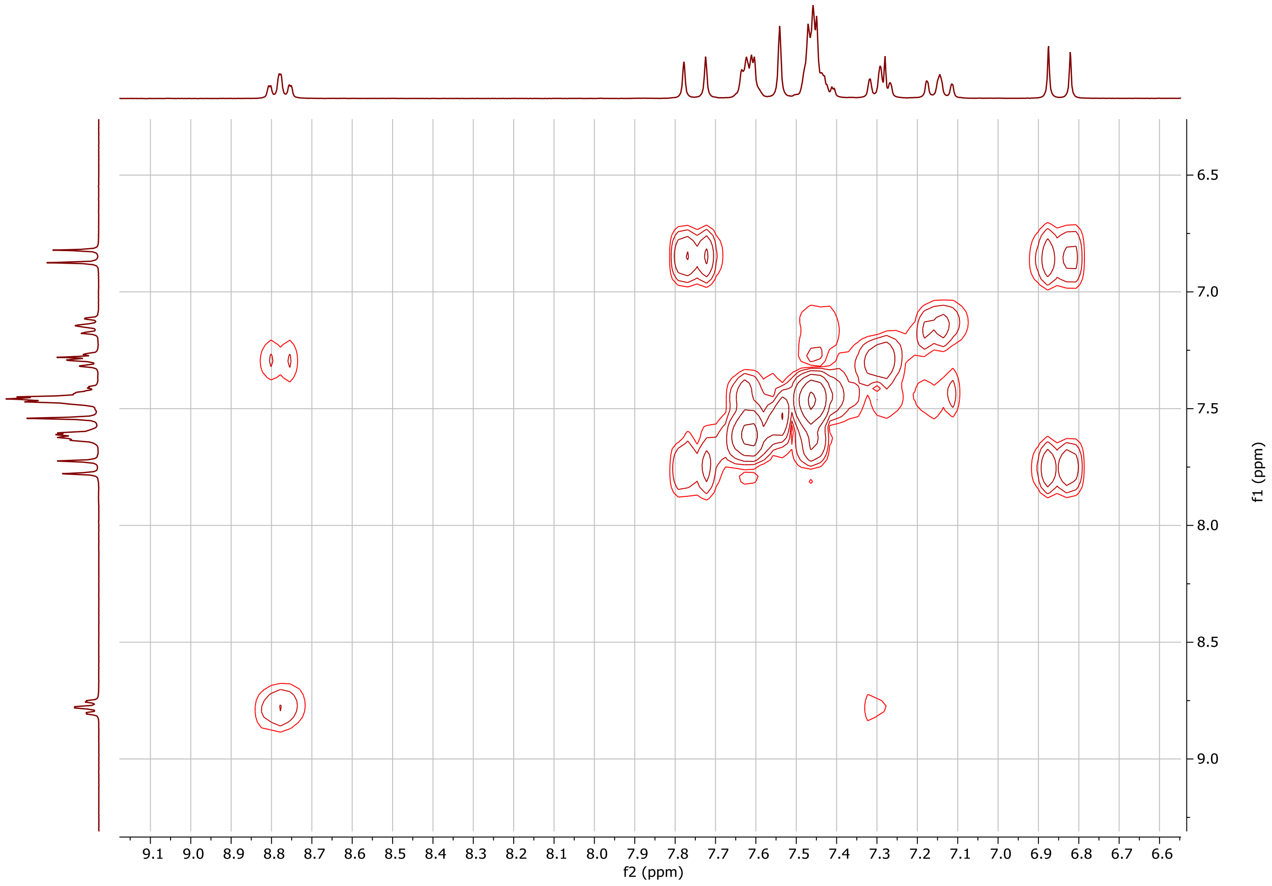

^{1}H-^{1}H COSY NMR spectrum of **2a**

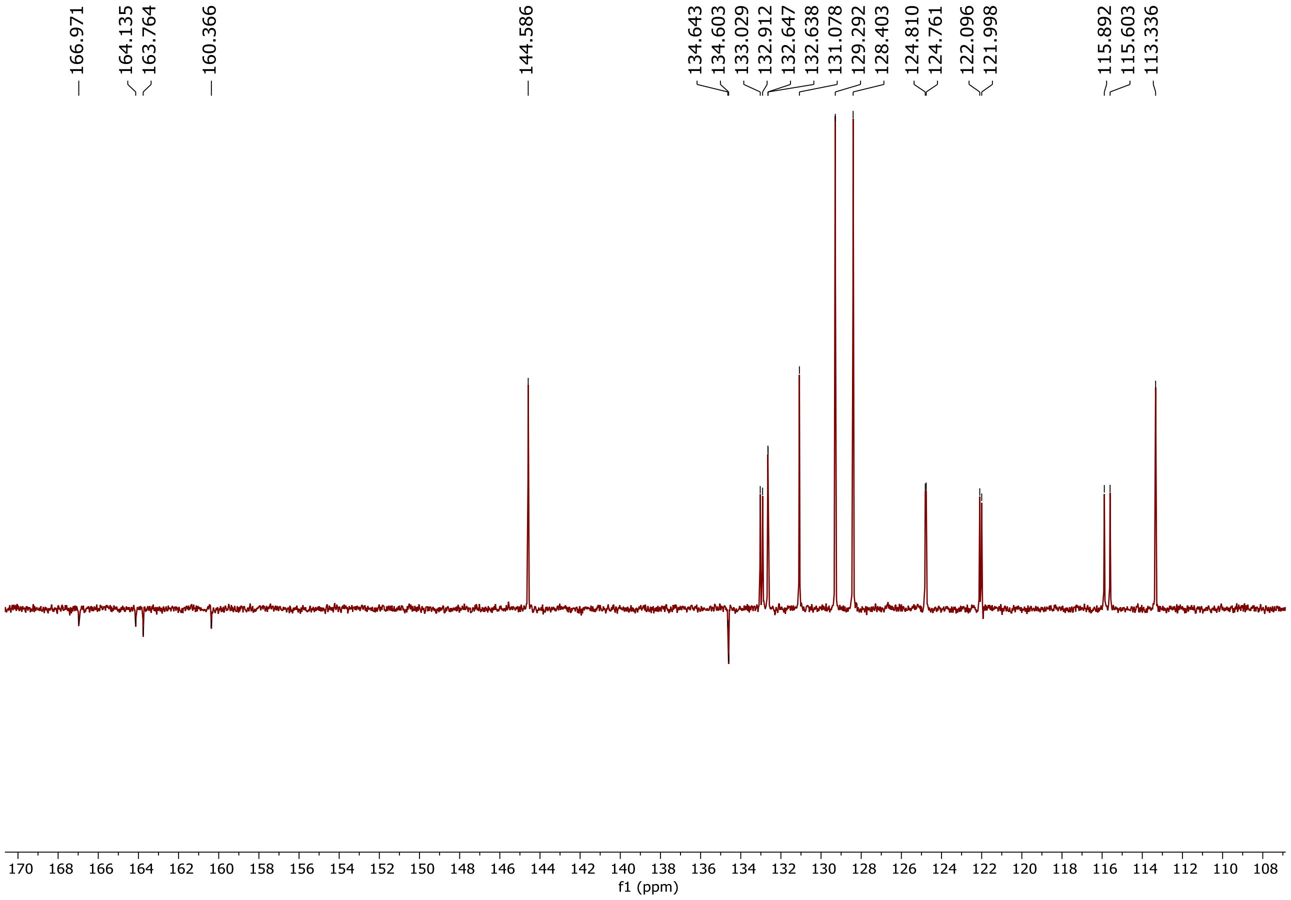

$^{13}C\{^1H\}$-(APT) NMR spectrum ($CDCl_3$, 75.47 MHz) of **2a**

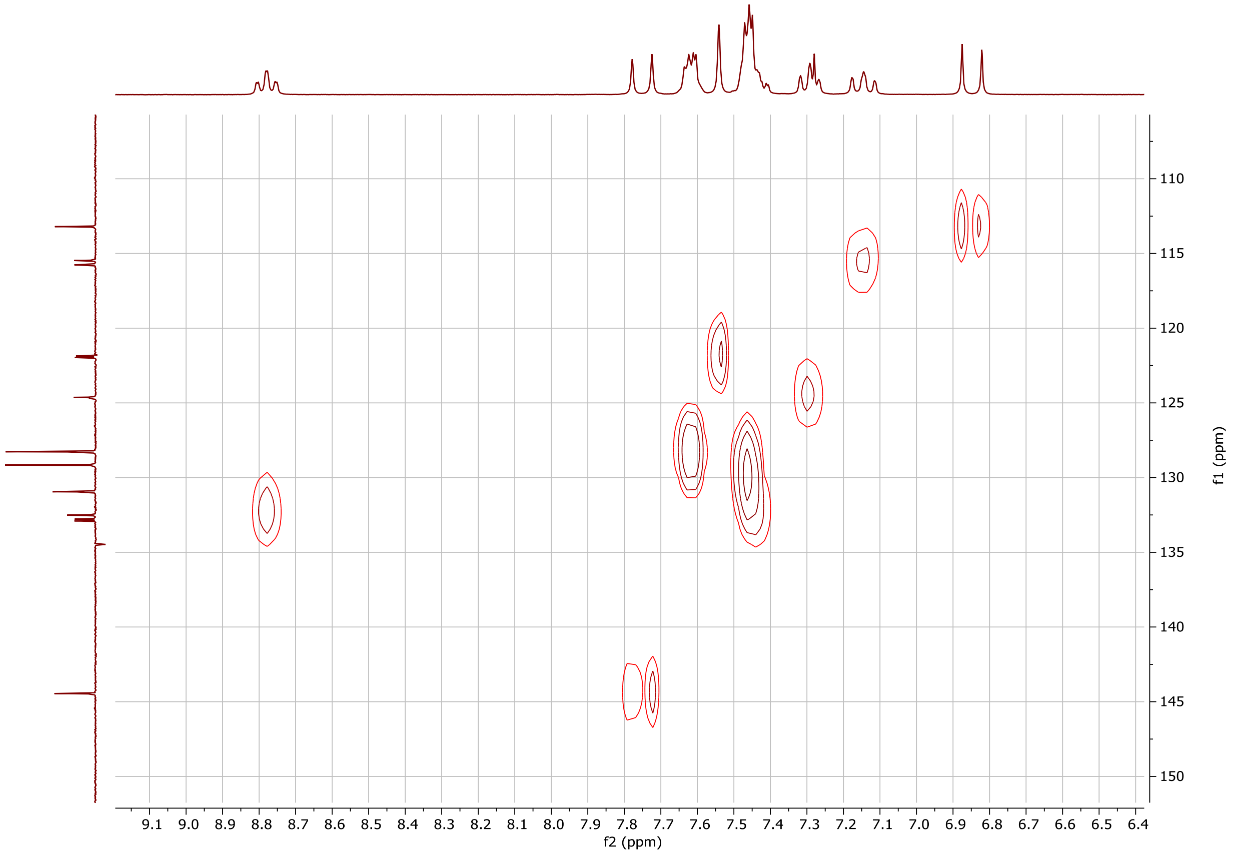

^1H-^{13}C HSQC NMR spectrum of **2a**

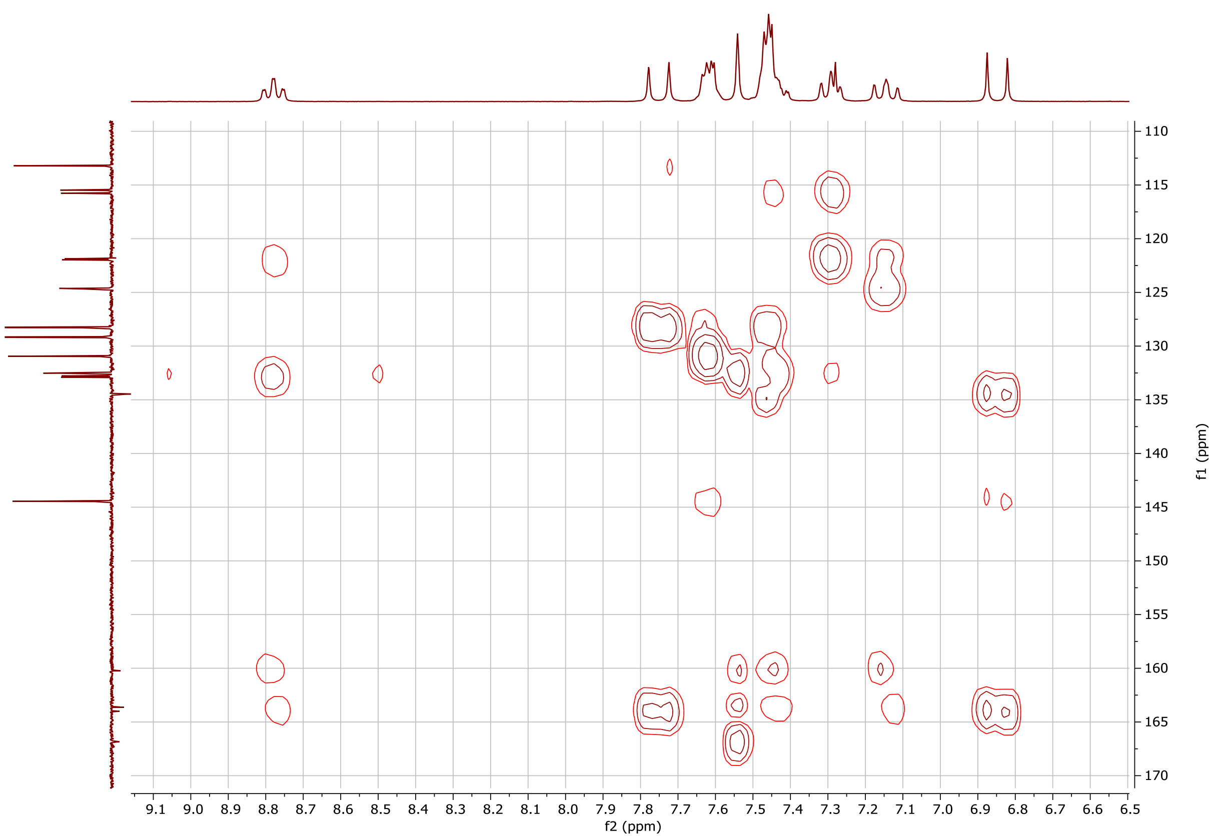

^1H-^{13}C HMBC NMR spectrum of **2a**

4-((*Z*)-4-Fluorobenzylidene)-2-((*E*)-styryl)oxazol-5(4*H*)-one (2b)

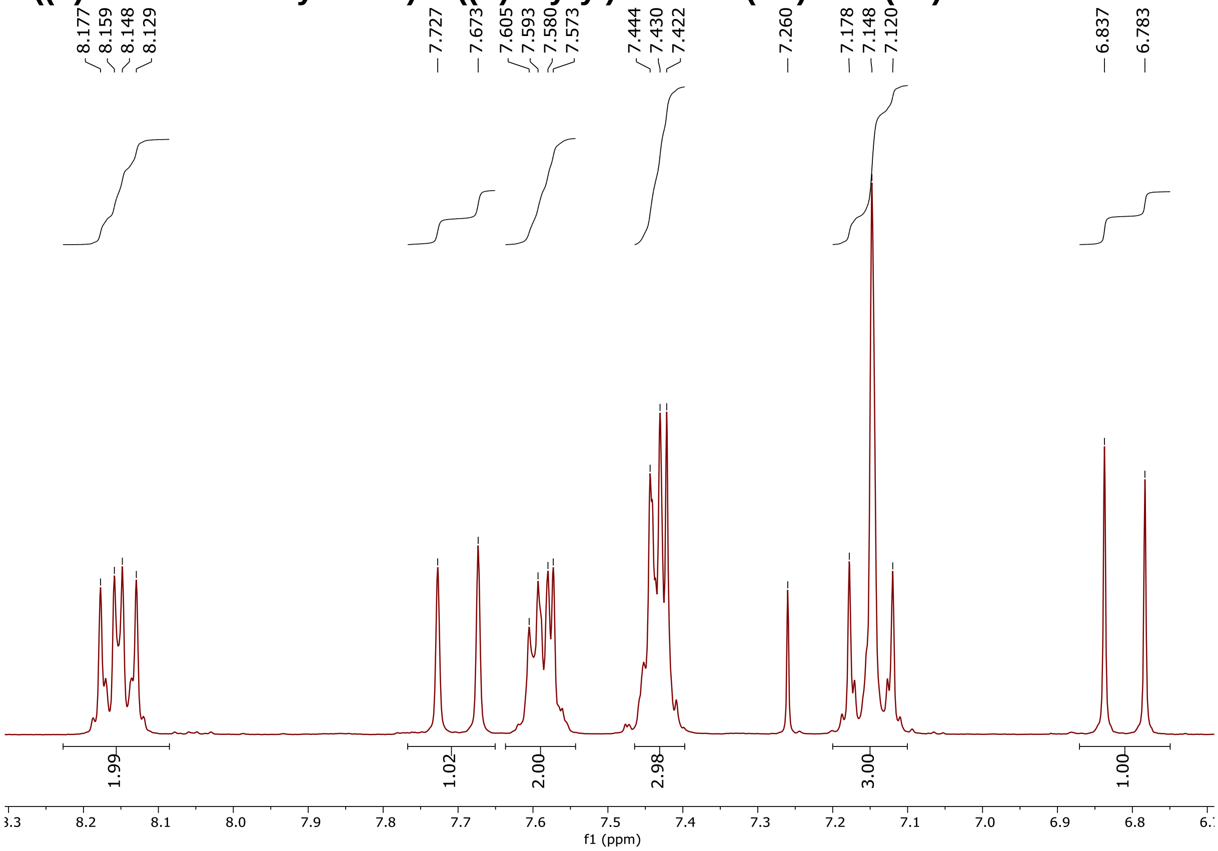

^1H NMR ($CDCl_3$, 300.13 MHz) of **2b**

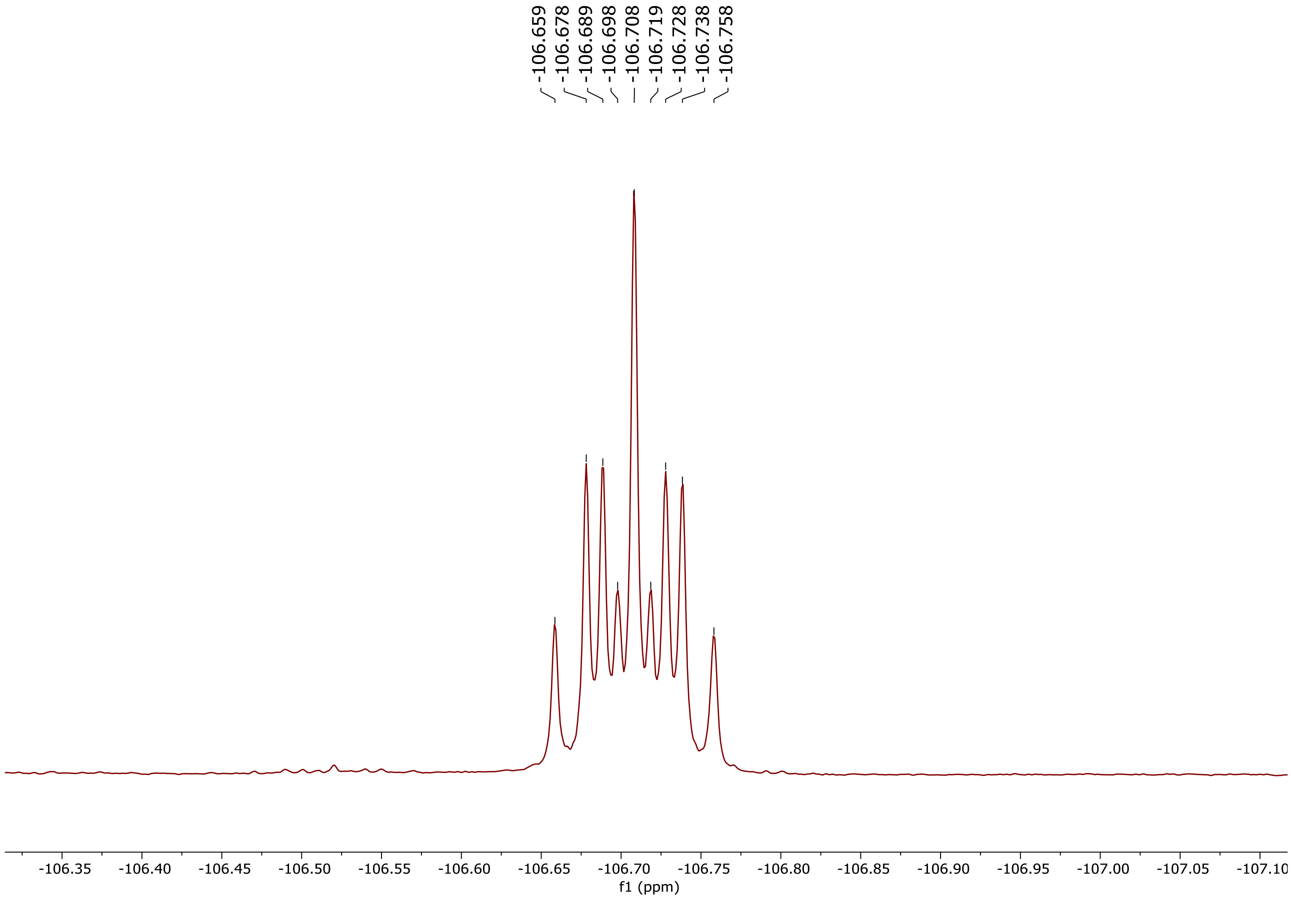

^{19}F-NMR spectrum ($CDCl_3$, 282.40 MHz) of **2b**

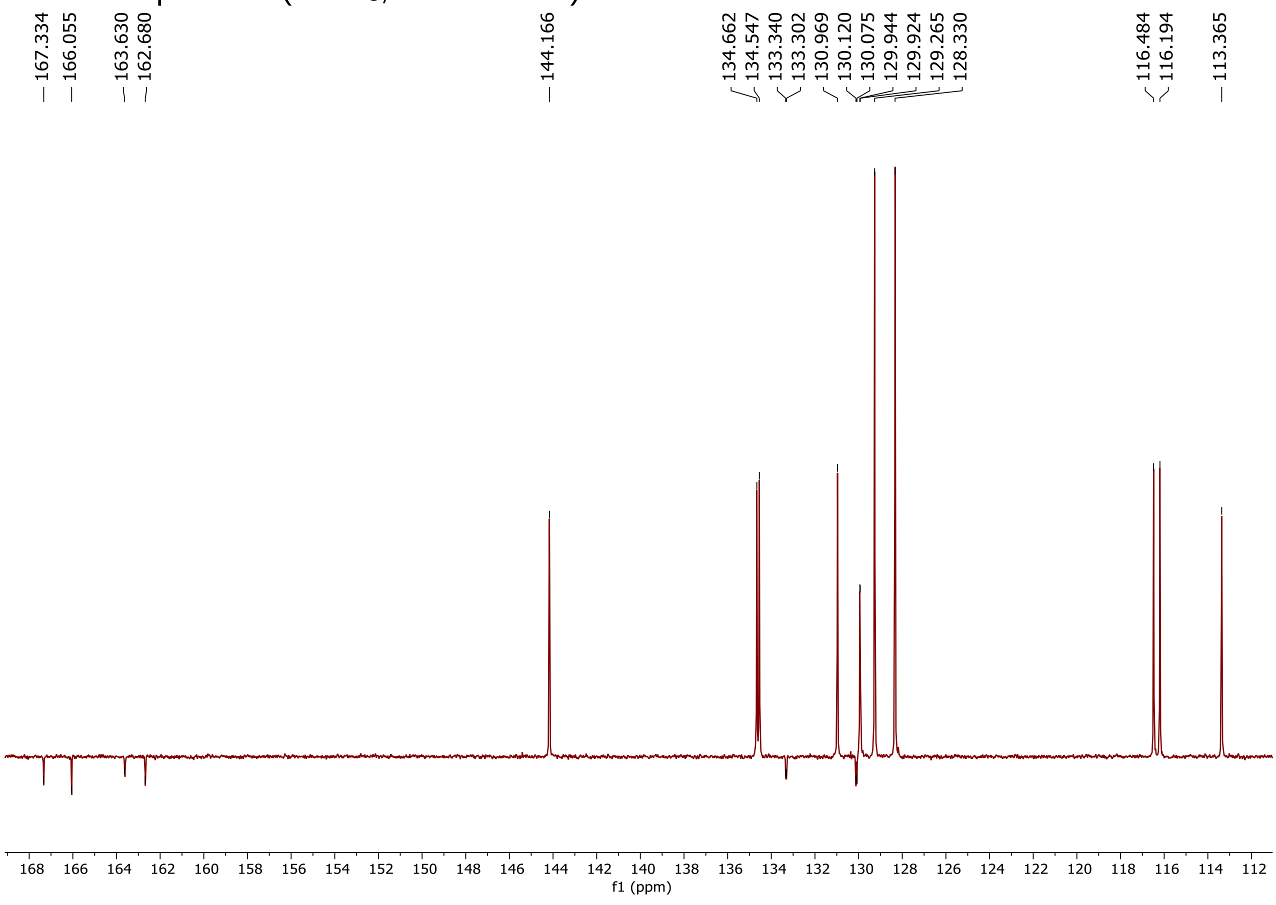

^{13}C{^{1}H}-(APT) NMR spectrum ($CDCl_3$, 75.47 MHz) of **2b**

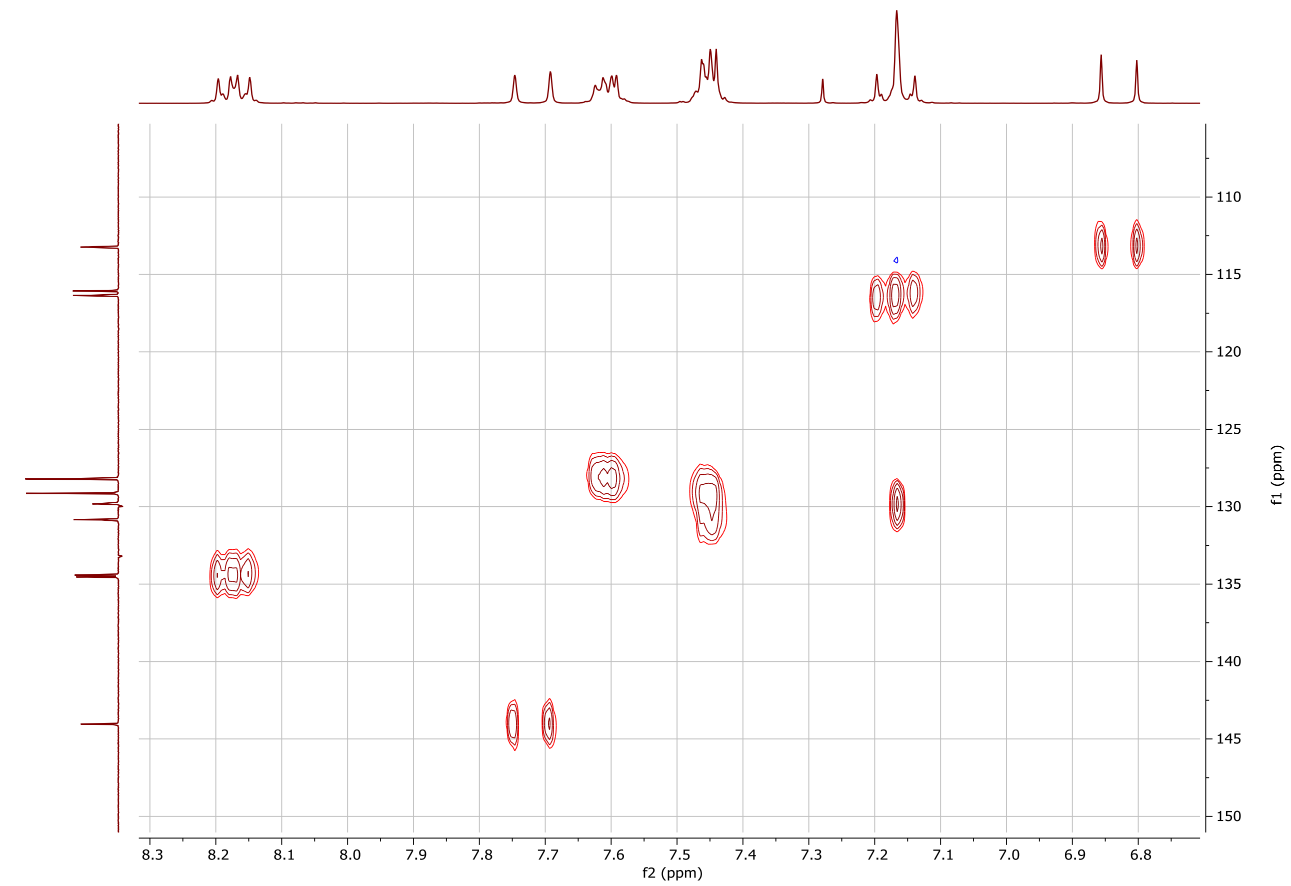

^{1}H-^{13}C HSQC NMR spectrum of **2b**

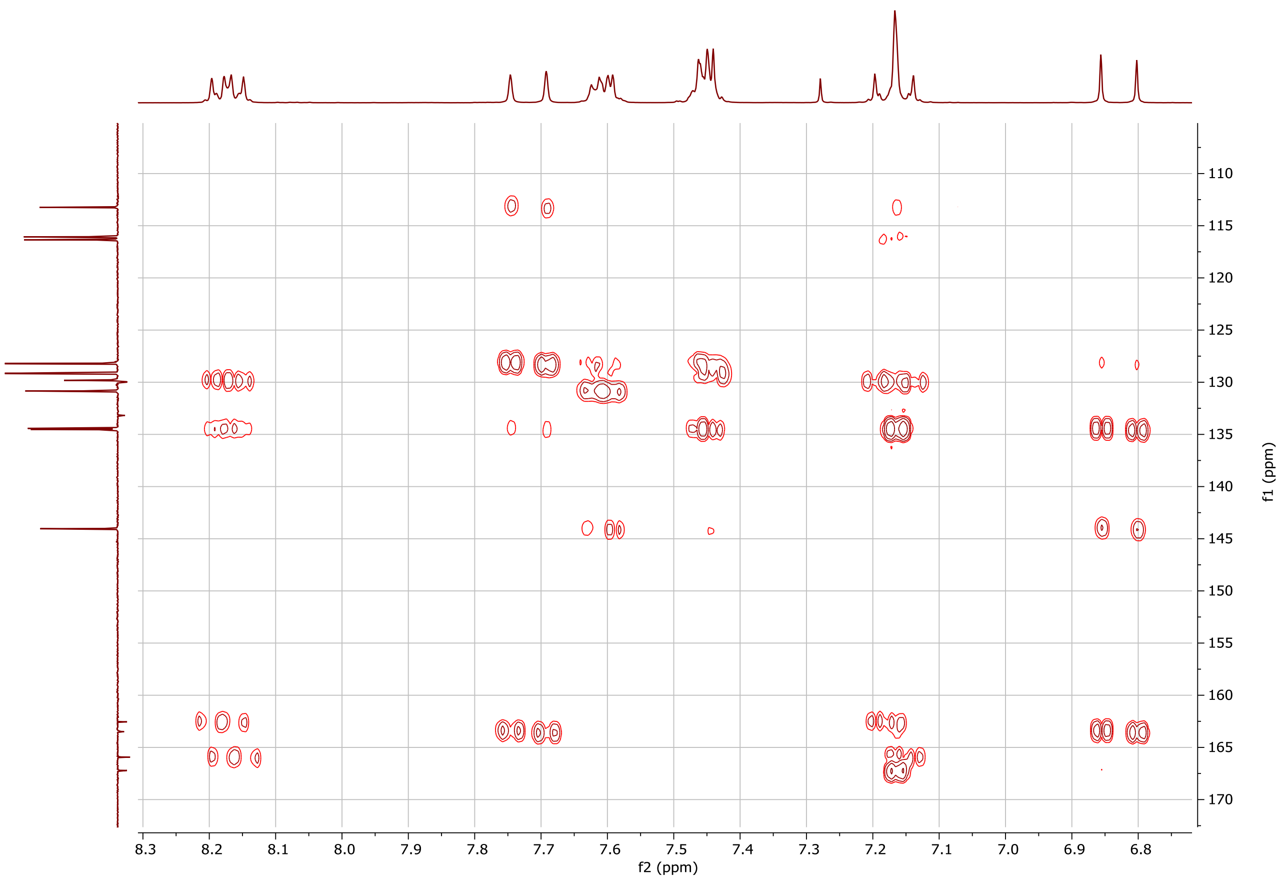

^{1}H-^{13}C HMBC NMR spectrum of **2b**

4-((*Z*)-2-Chlorobenzylidene)-2-((*E*)-styryl)oxazol-5(4*H*)-one (2c)

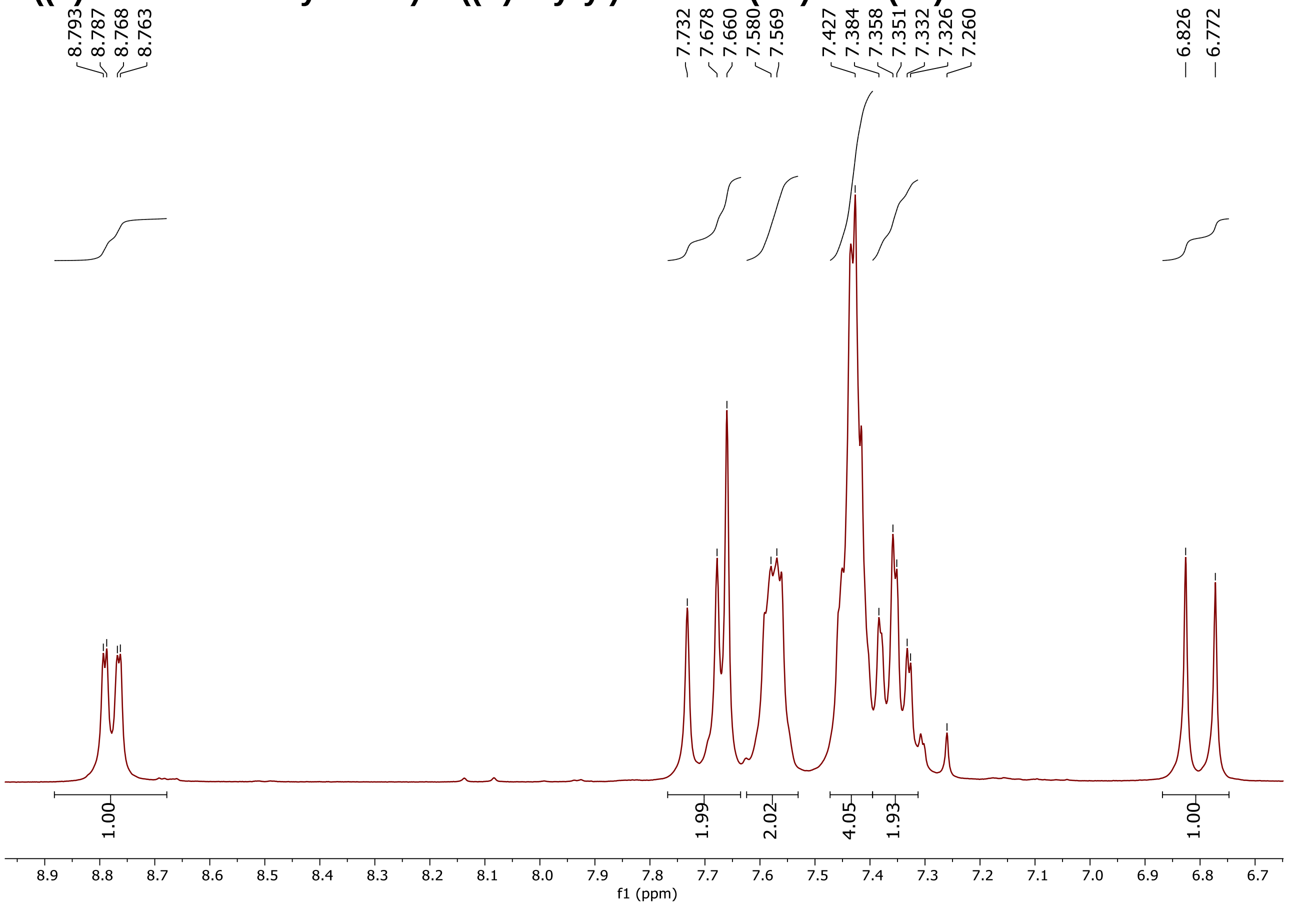

^{1}H NMR ($CDCl_3$, 300.13 MHz) of **2c**

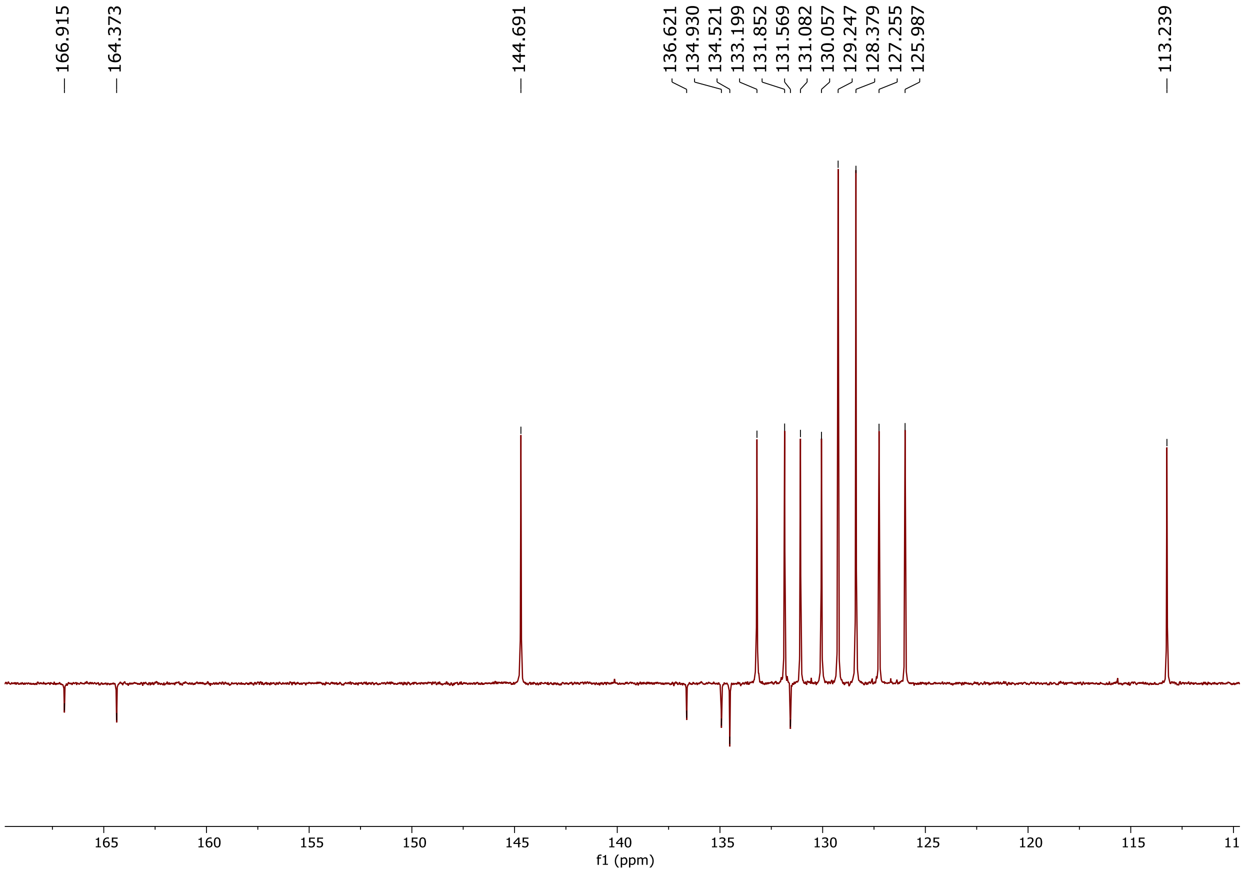

$^{13}C\{^{1}H\}$-(APT) NMR spectrum ($CDCl_3$, 75.47 MHz) of **2c**

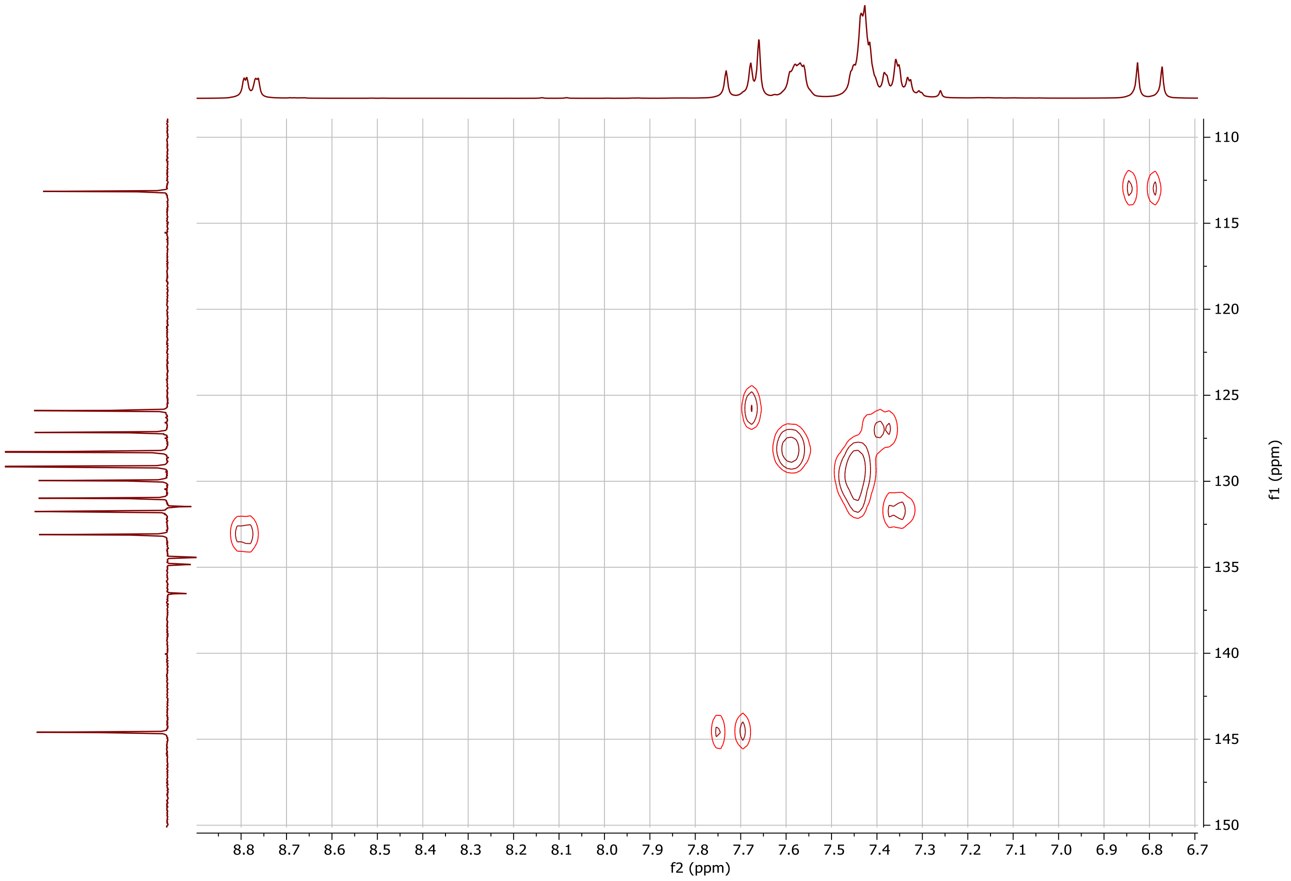

^{1}H-^{13}C HSQC NMR spectrum of **2c**

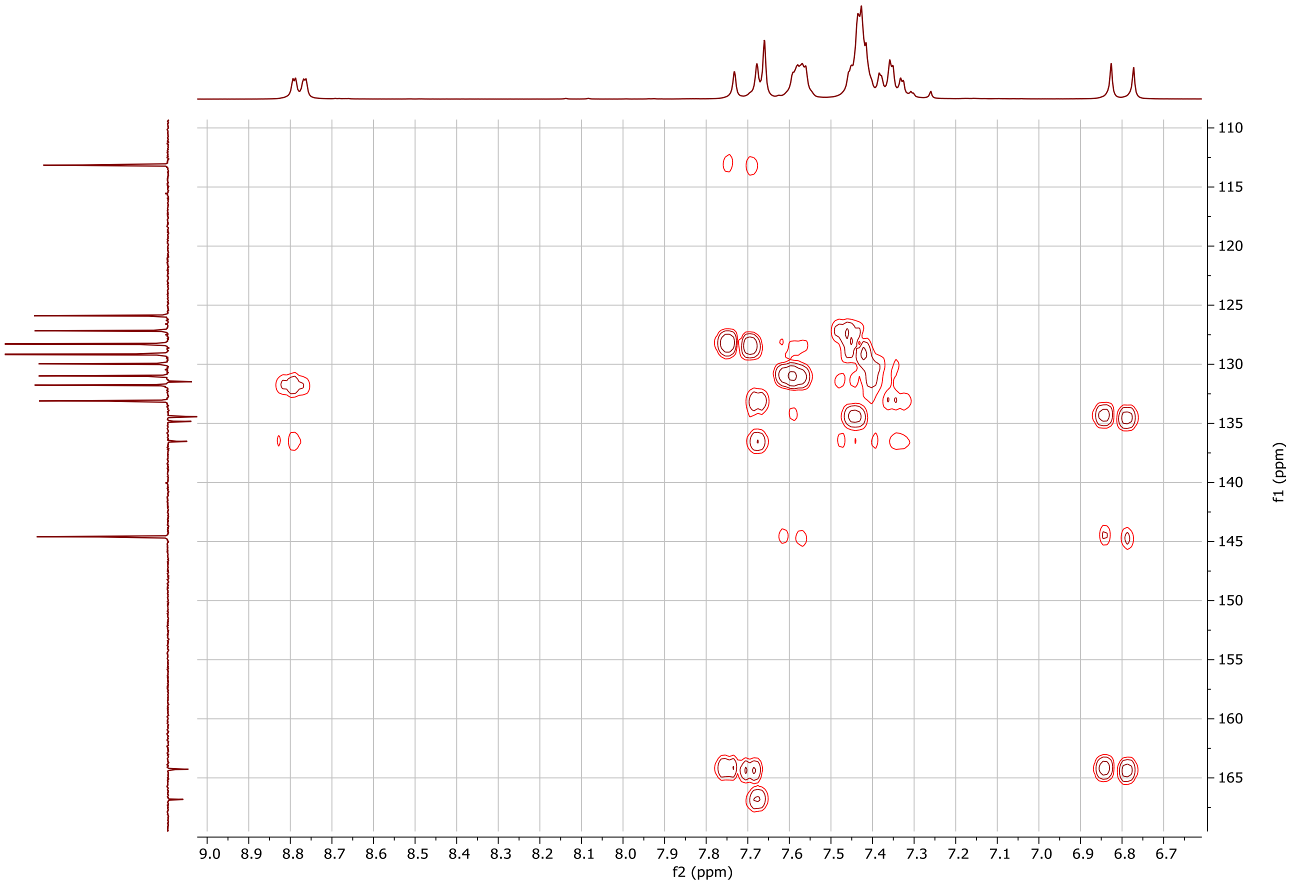

^{1}H-^{13}C HMBC NMR spectrum of **2c**

4-((*Z*)-4-Chlorobenzylidene)-2-((*E*)-styryl)oxazol-5(4*H*)-one (2d)

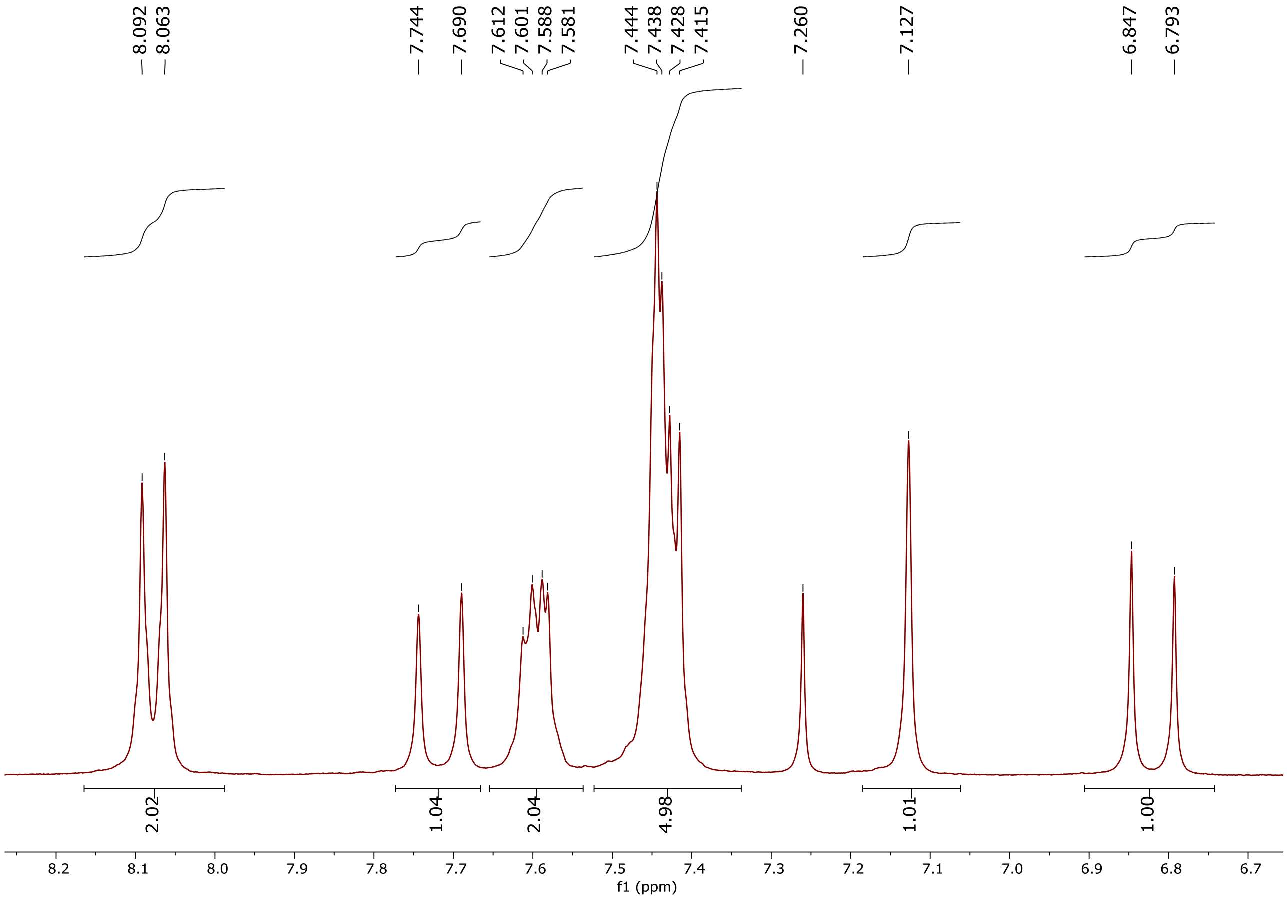

^{1}H NMR ($CDCl_3$, 300.13 MHz) of **2d**

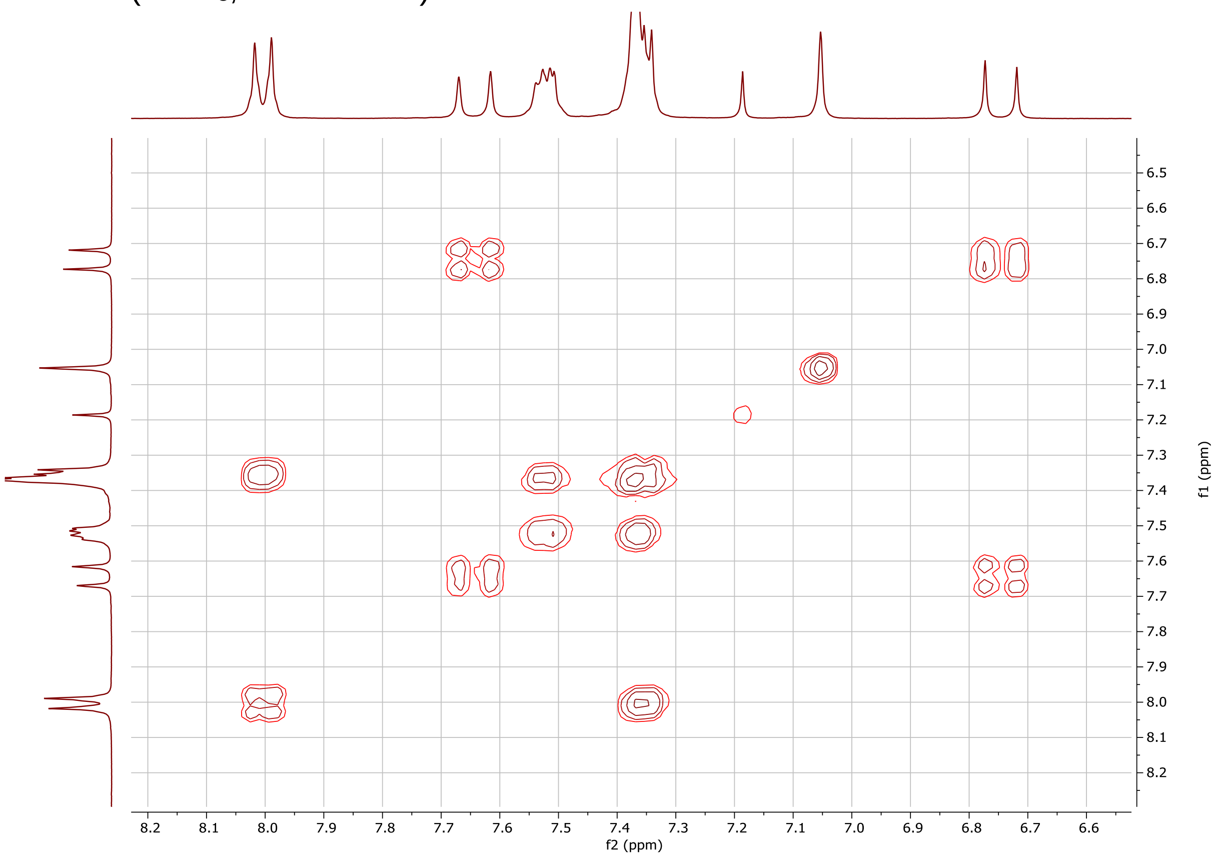

^{1}H-^{1}H COSY NMR spectrum of **2d**

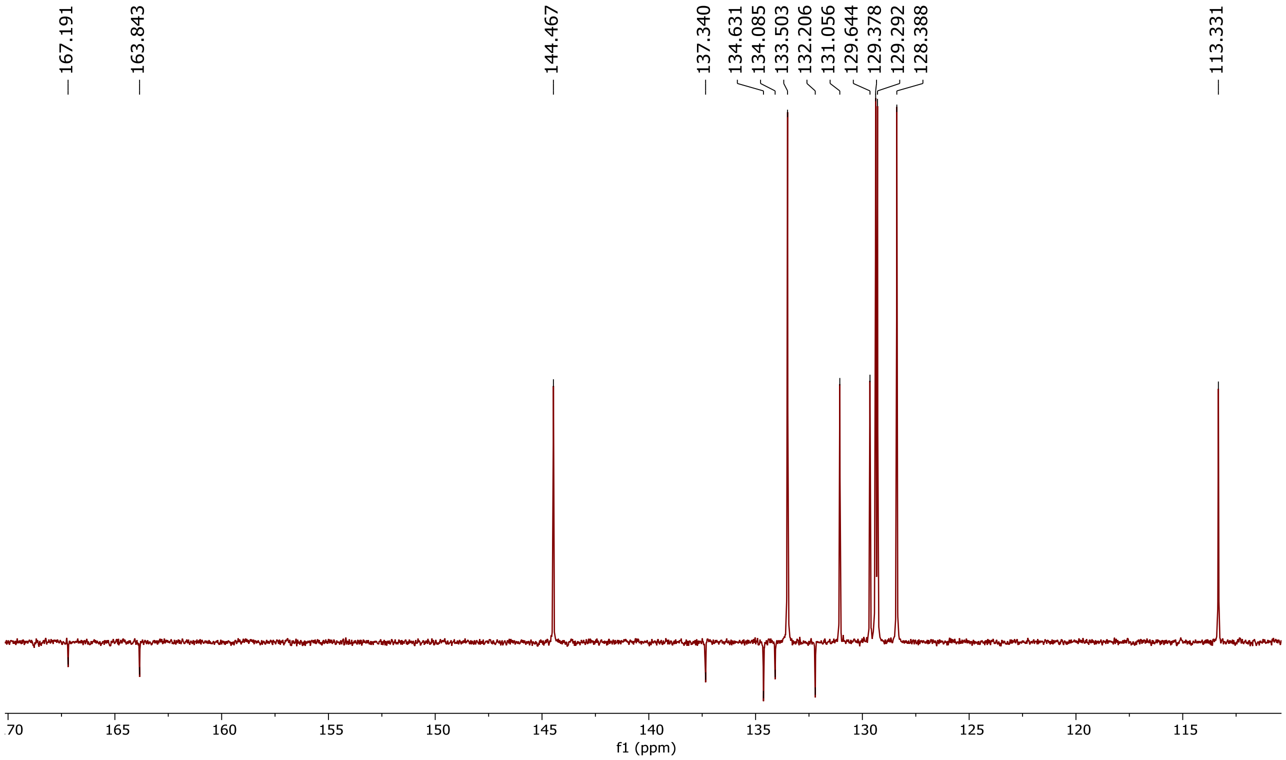

$^{13}C\{^{1}H\}$-(APT) NMR spectrum ($CDCl_3$, 75.47 MHz) of **2d**

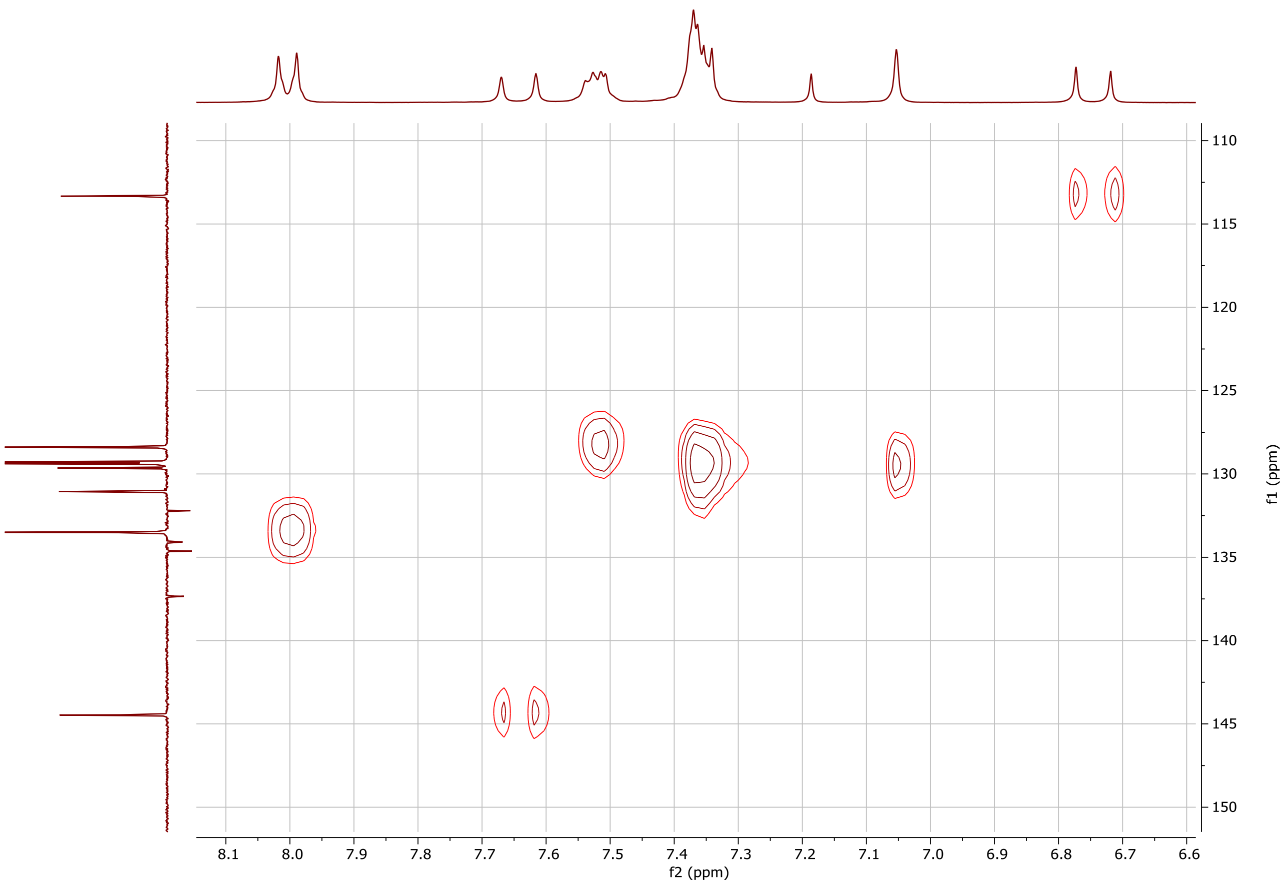

^{1}H-^{13}C HSQC NMR spectrum of **2d**

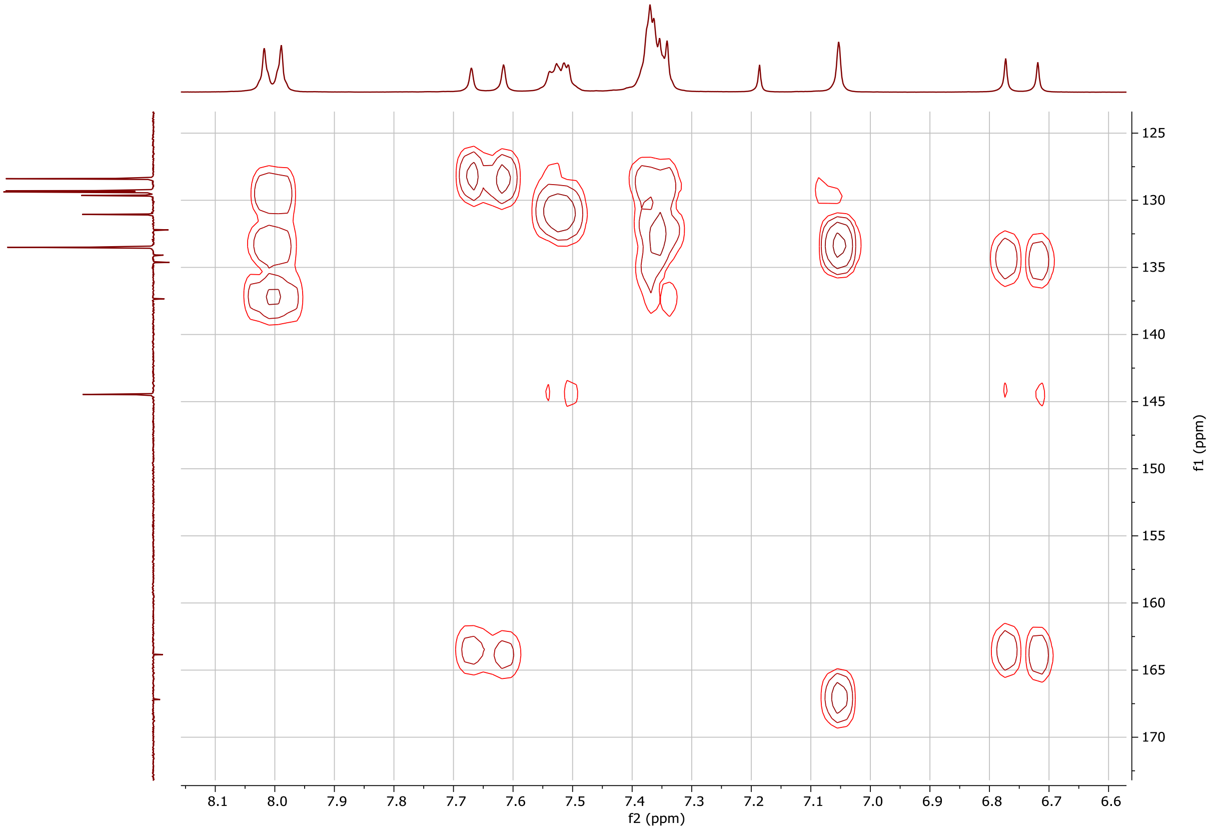

^1H-^{13}C HMBC NMR spectrum of **2d**

4-((*Z*)-2-Trifluoromethylbenzylidene)-2-((*E*)-styryl)oxazol-5(4*H*)-one (2e)

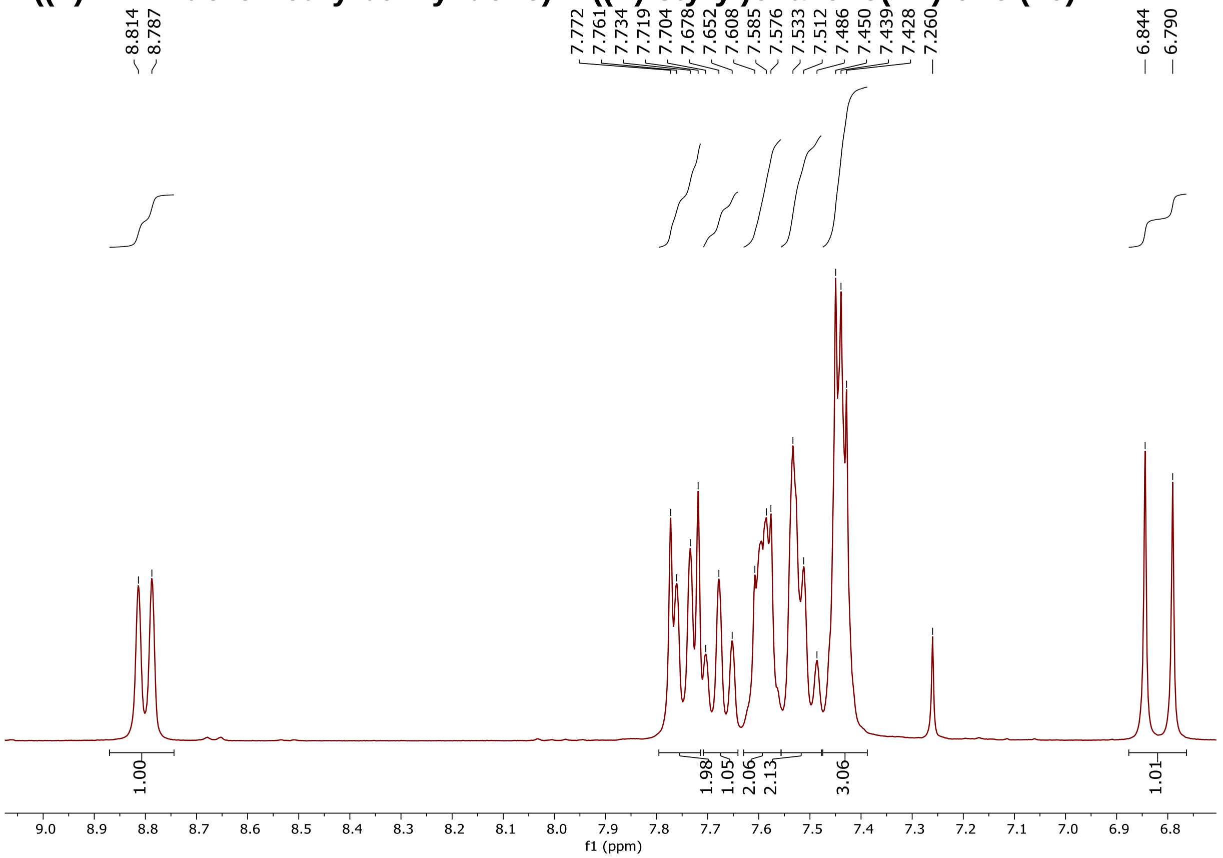

^1H NMR ($CDCl_3$, 300.13 MHz) of **2e**

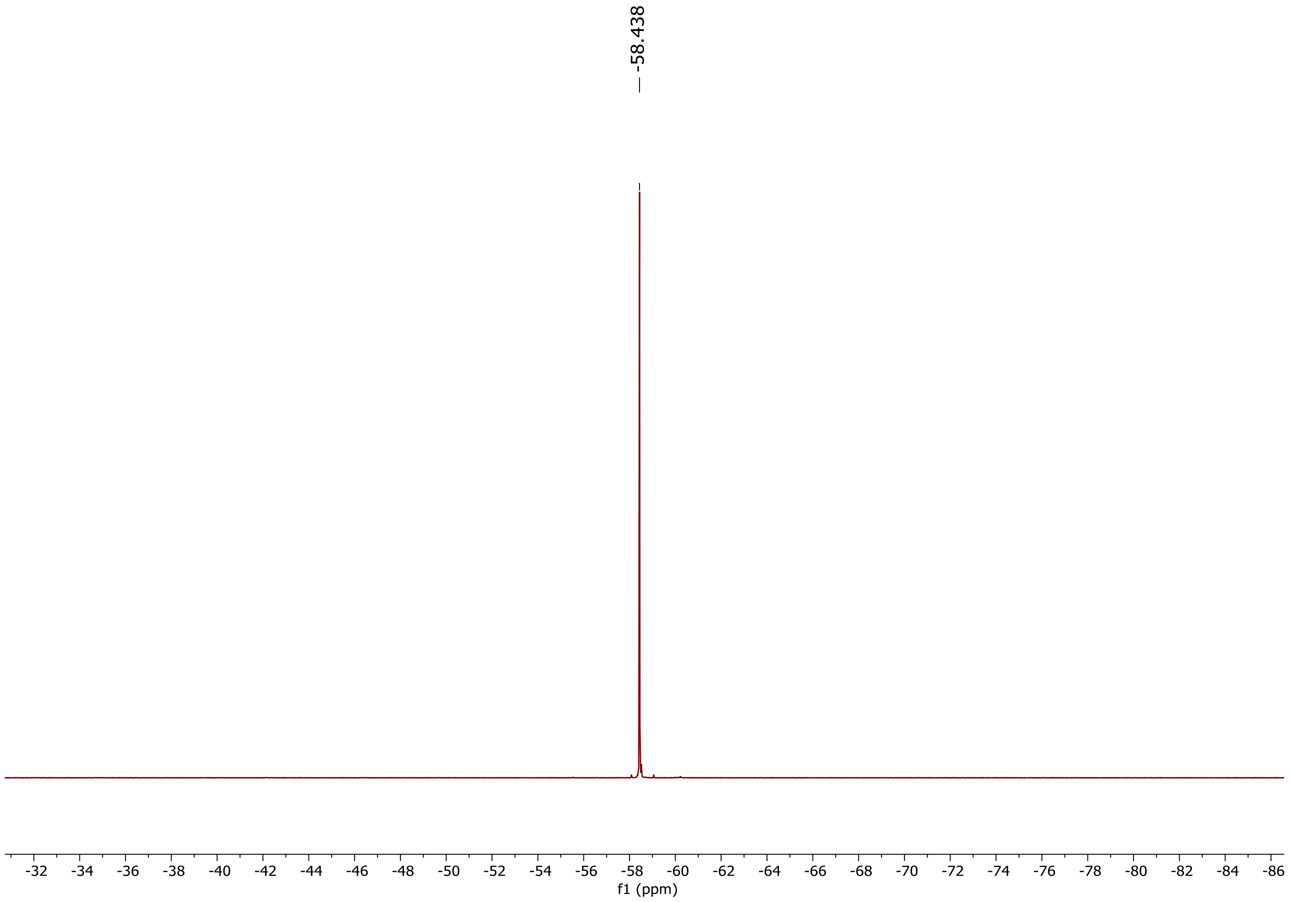

^{19}F-NMR spectrum ($CDCl_3$, 282.40 MHz) of **2e**

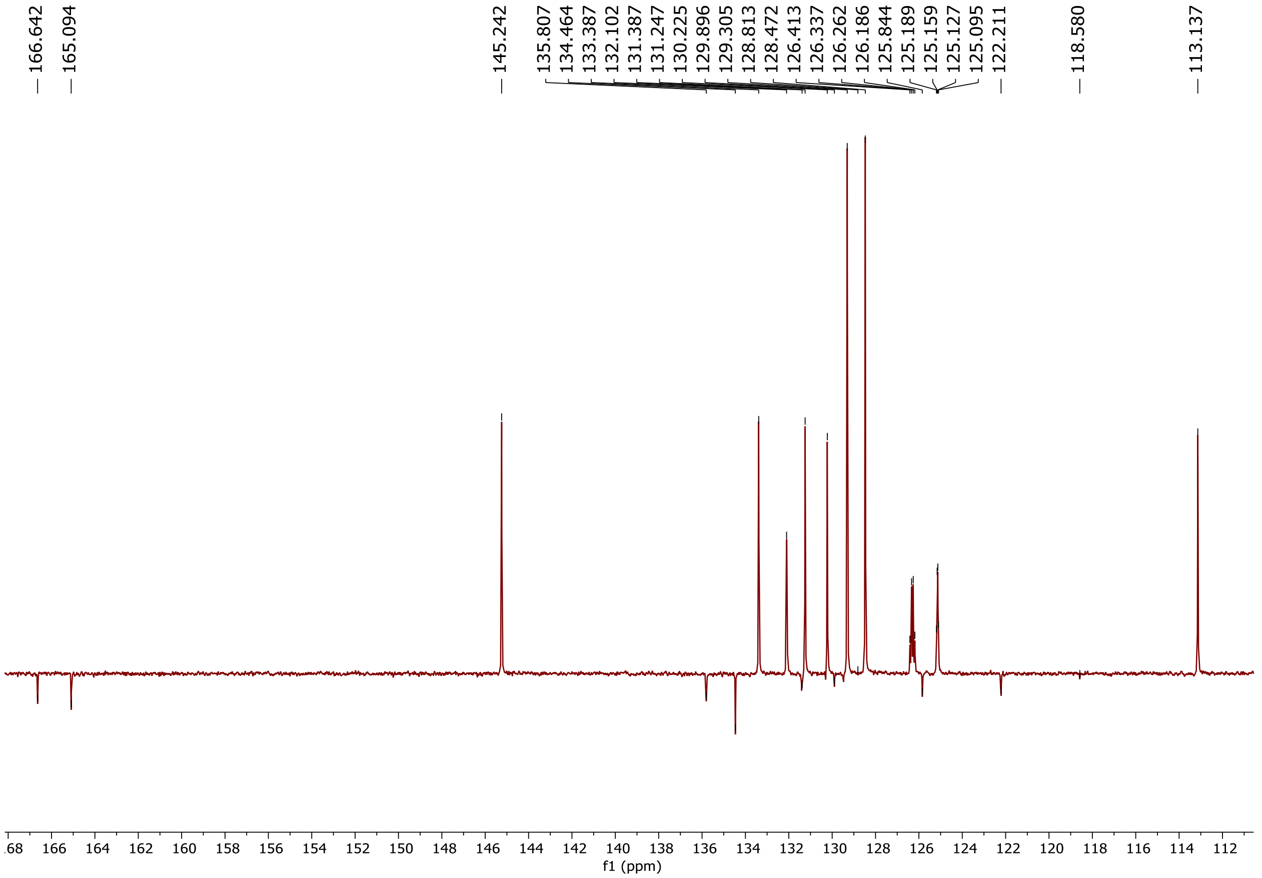

$^{13}C\{^{1}H\}$-(APT) NMR spectrum ($CDCl_3$, 75.47 MHz) of **2e**

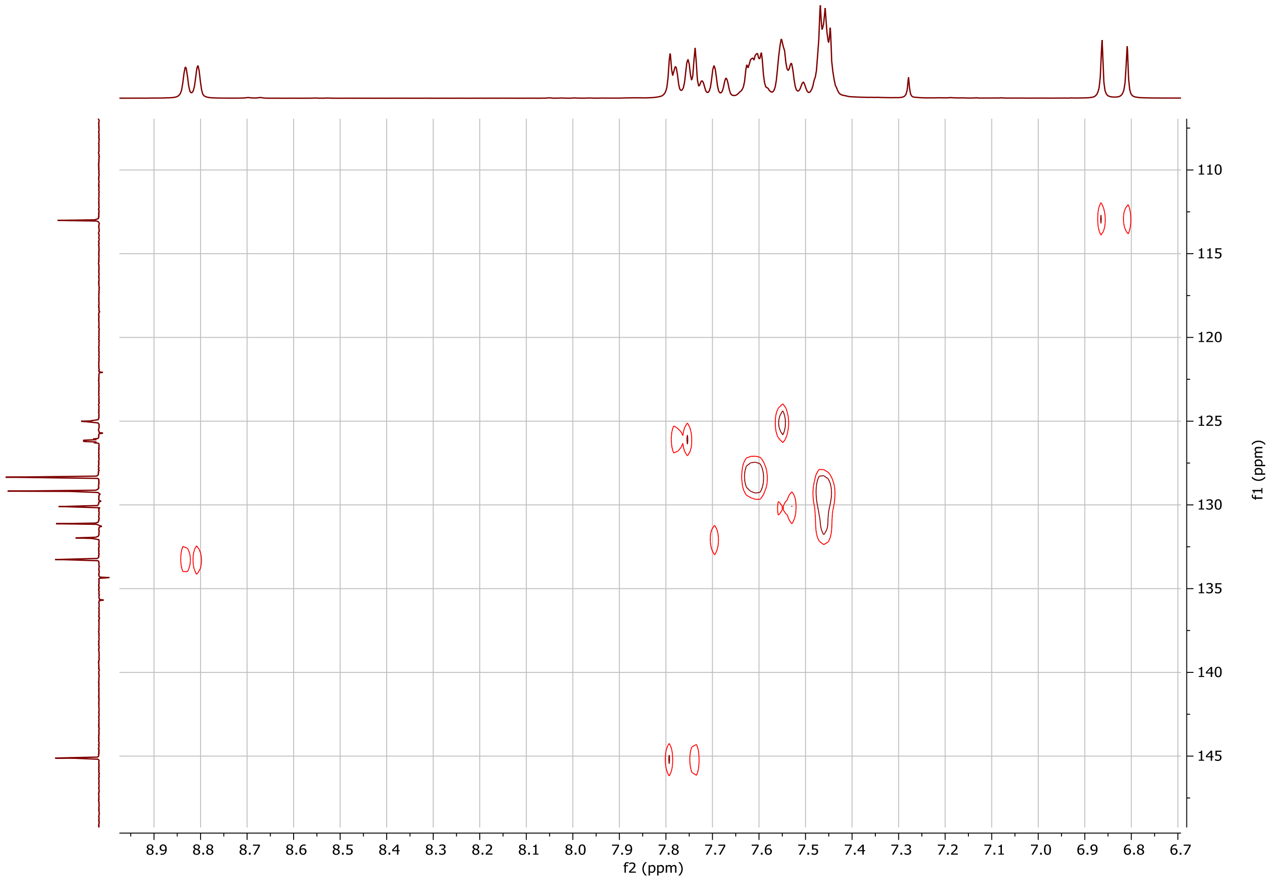

^{1}H-^{13}C HSQC NMR spectrum of **2e**

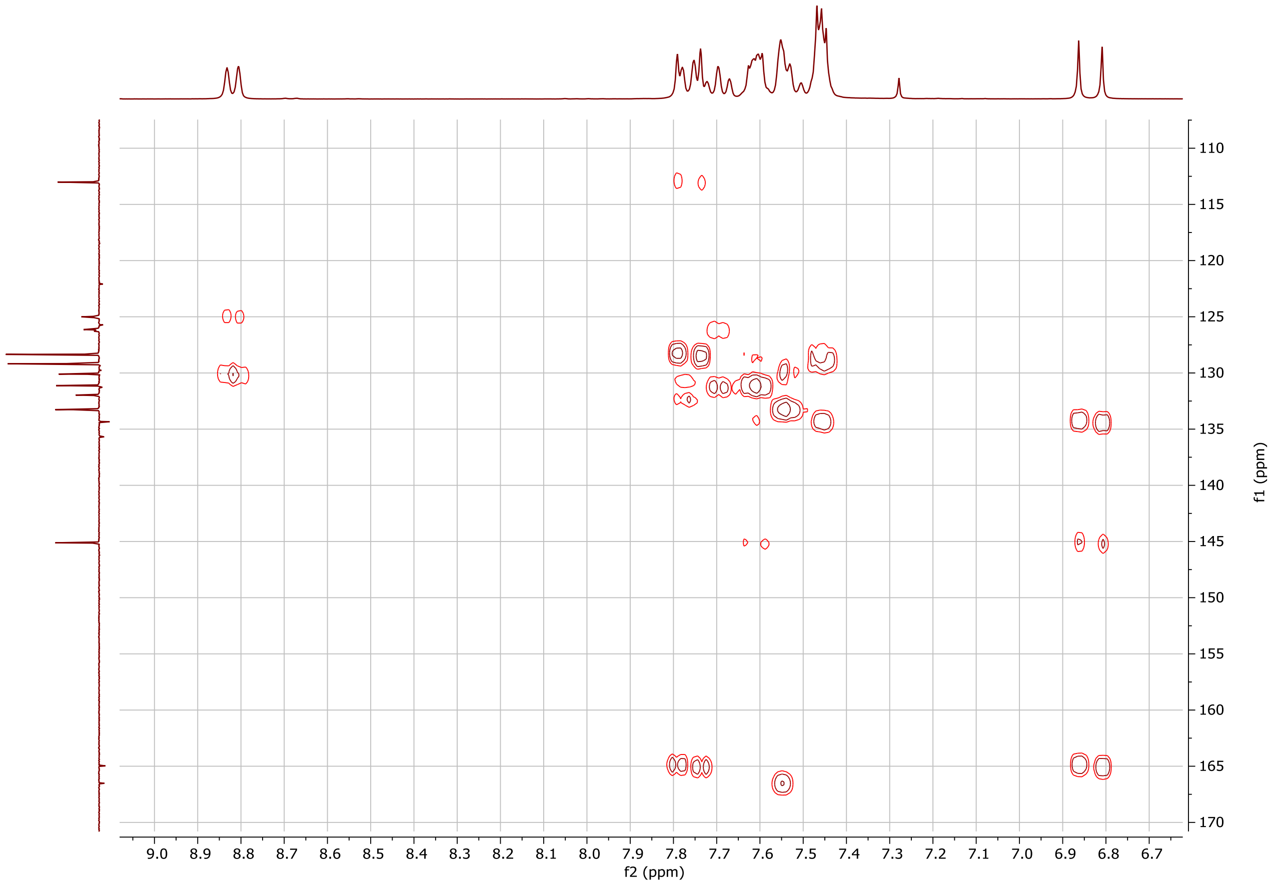

^{1}H-^{13}C HMBC NMR spectrum of **2e**

4-((*Z*)-4-Trifluoromethylbenzylidene)-2-((*E*)-styryl)oxazol-5(4*H*)-one (2f)

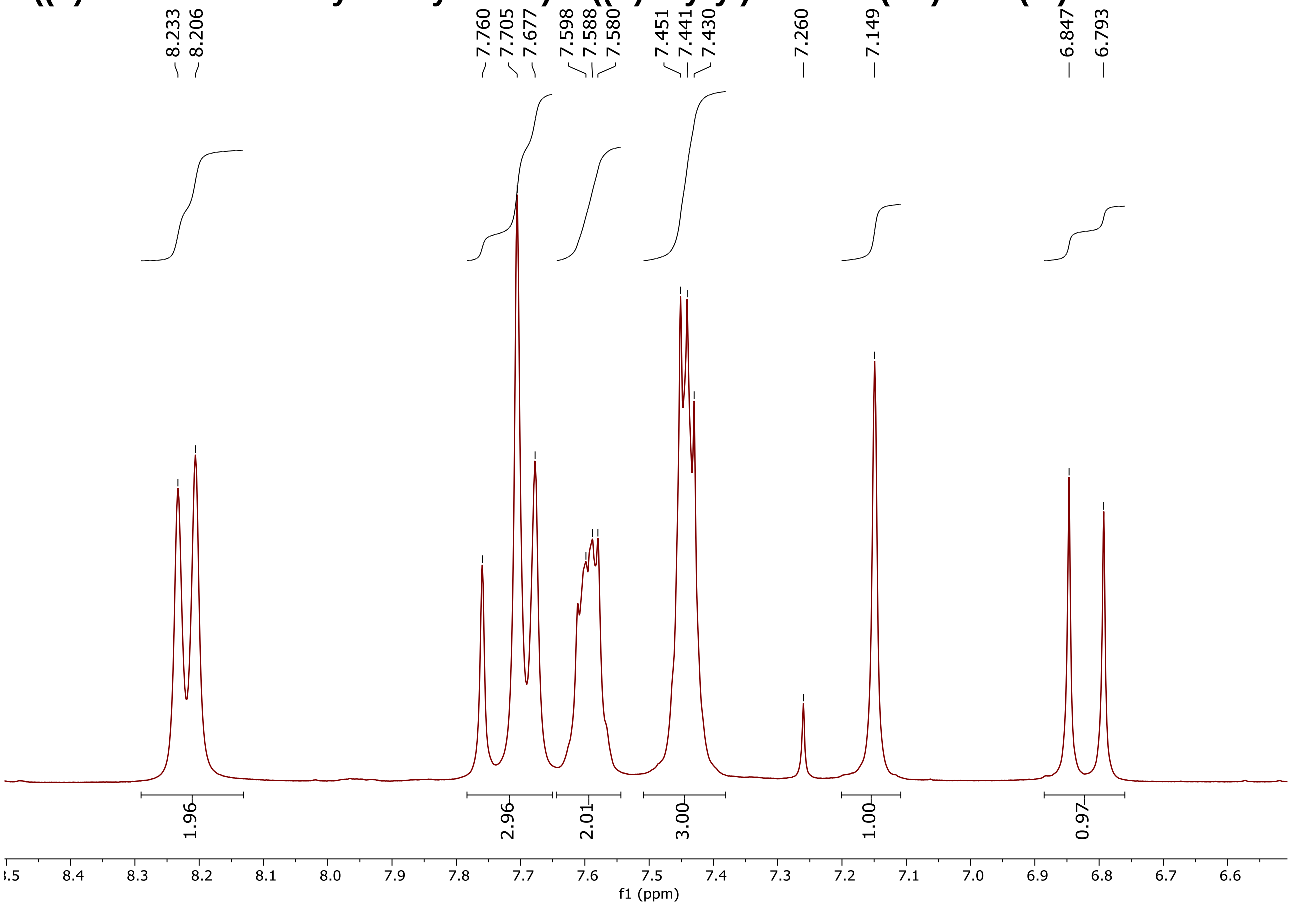

^{1}H NMR ($CDCl_3$, 300.13 MHz) of **2f**

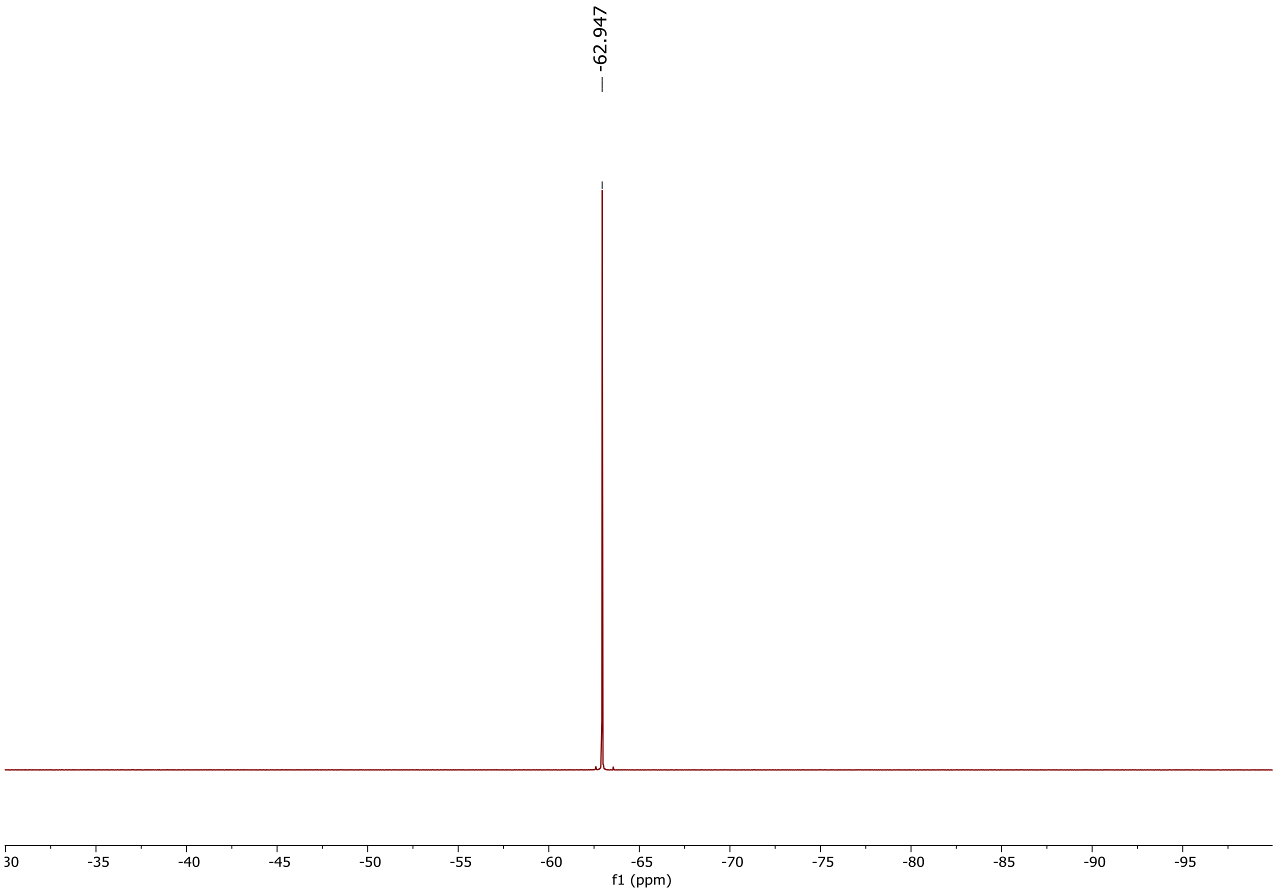

^{19}F-NMR spectrum ($CDCl_3$, 282.40 MHz) of **2f**

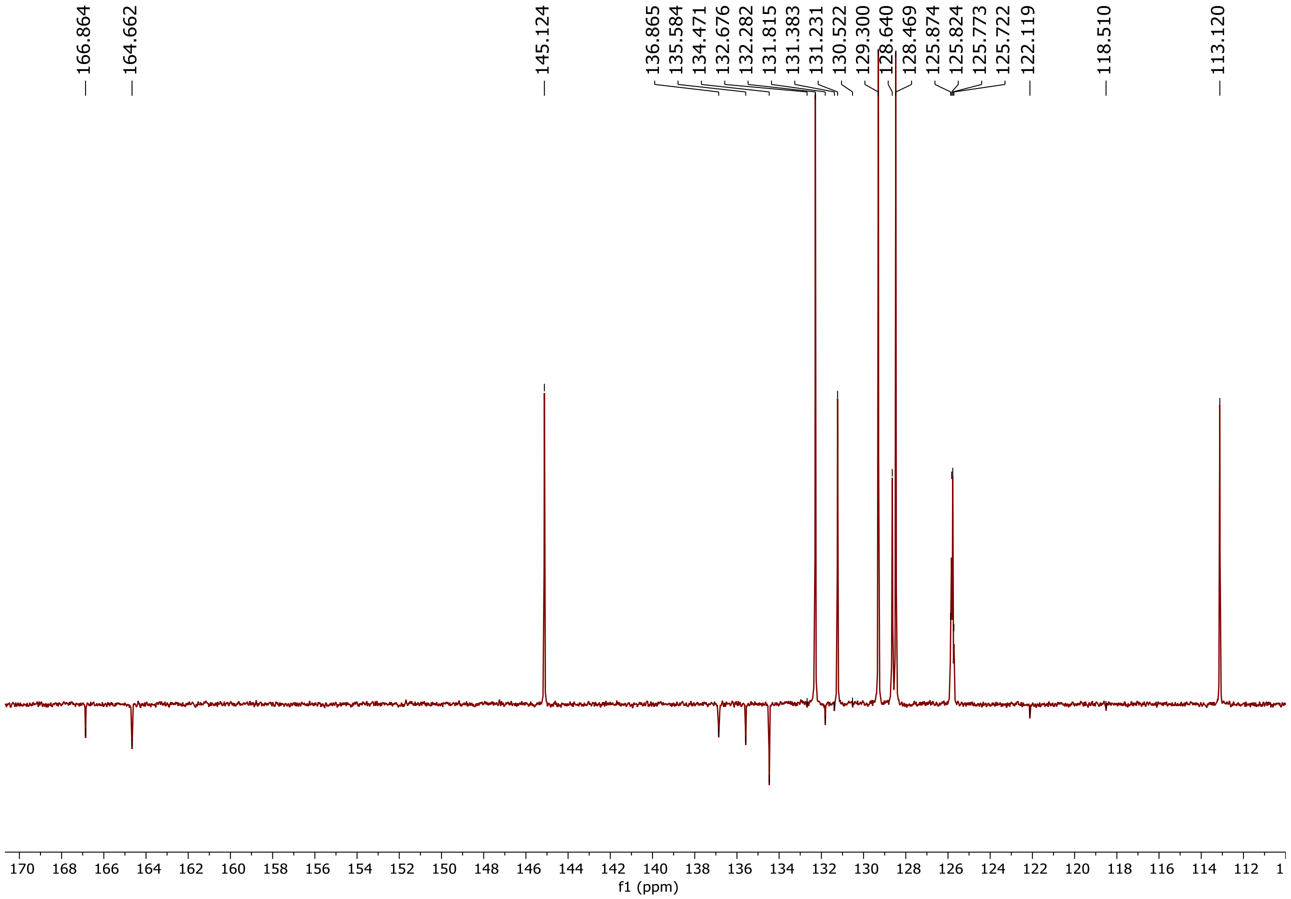

$^{13}C\{^{1}H\}$-(APT) NMR spectrum ($CDCl_3$, 75.47 MHz) of **2f**

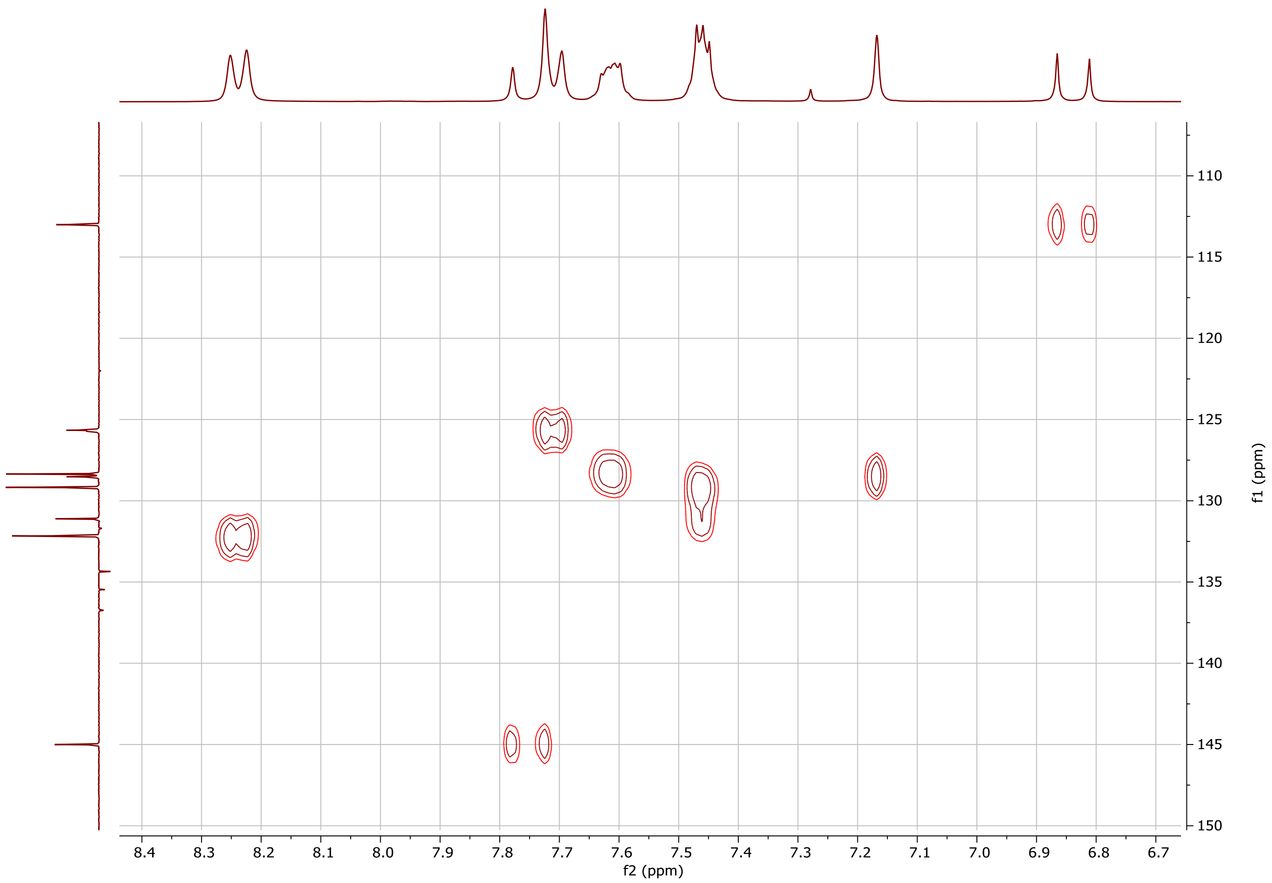

^{1}H-^{13}C HSQC NMR spectrum of **2f**

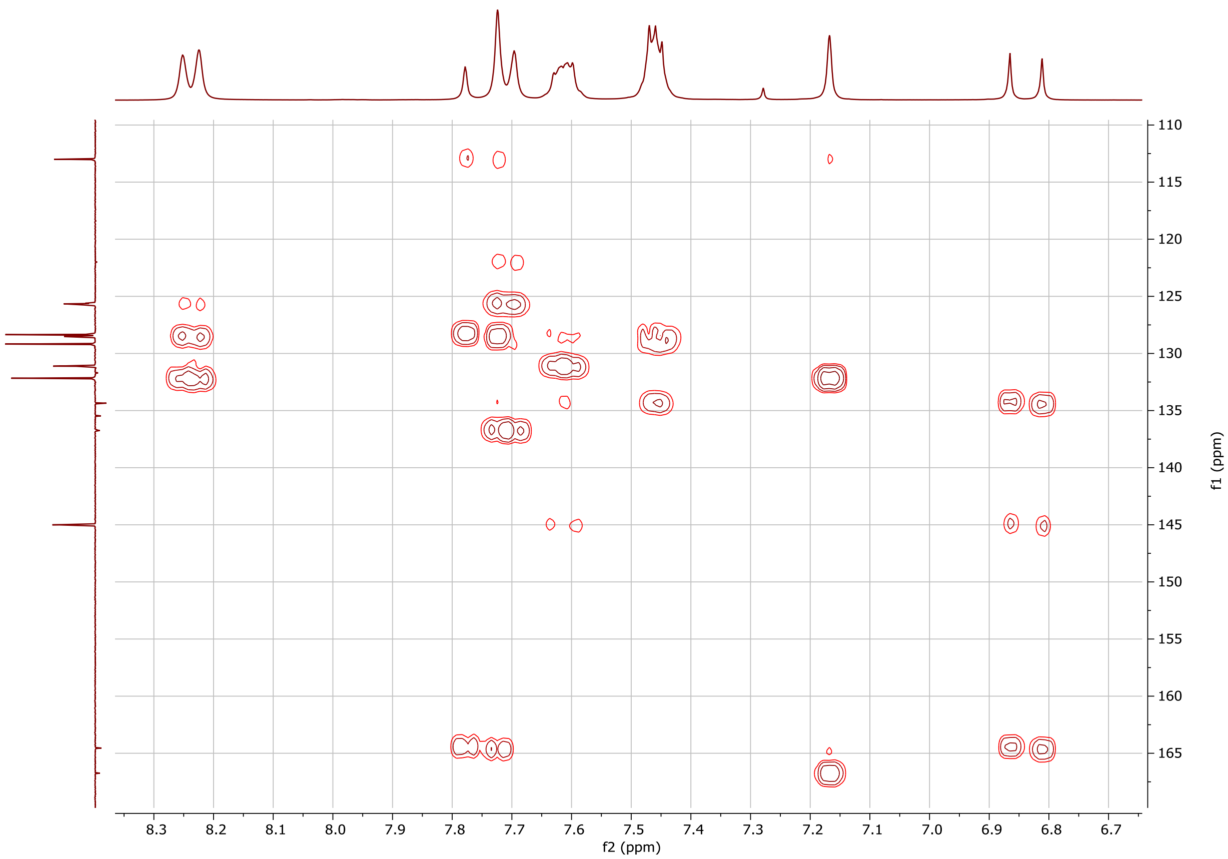

^1H-^{13}C HMBC NMR spectrum of **2f**

4-((*Z*)-3,4-Difluorobenzylidene)-2-((*E*)-styryl)oxazol-5(4*H*)-one (2g)

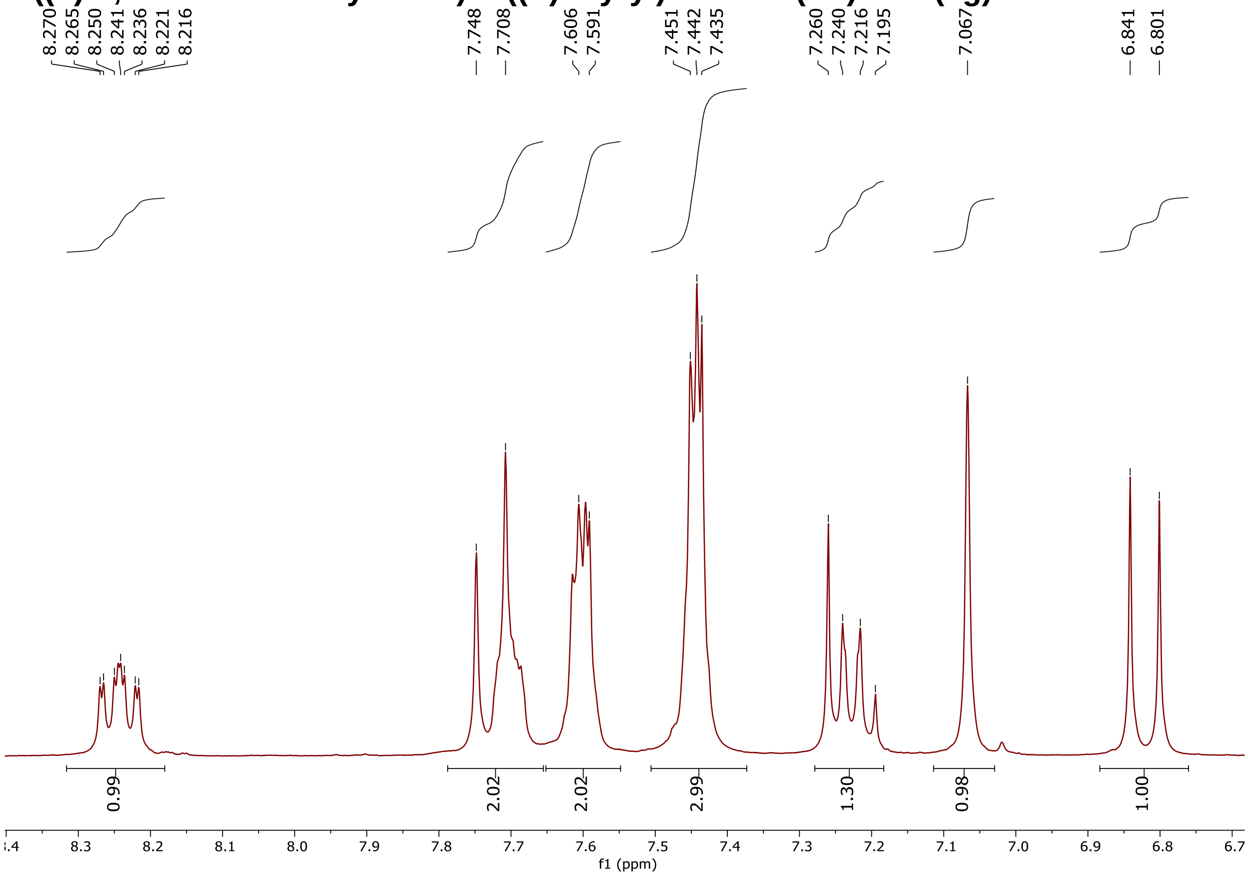

^1H NMR ($CDCl_3$, 400.13 MHz) of **2g**

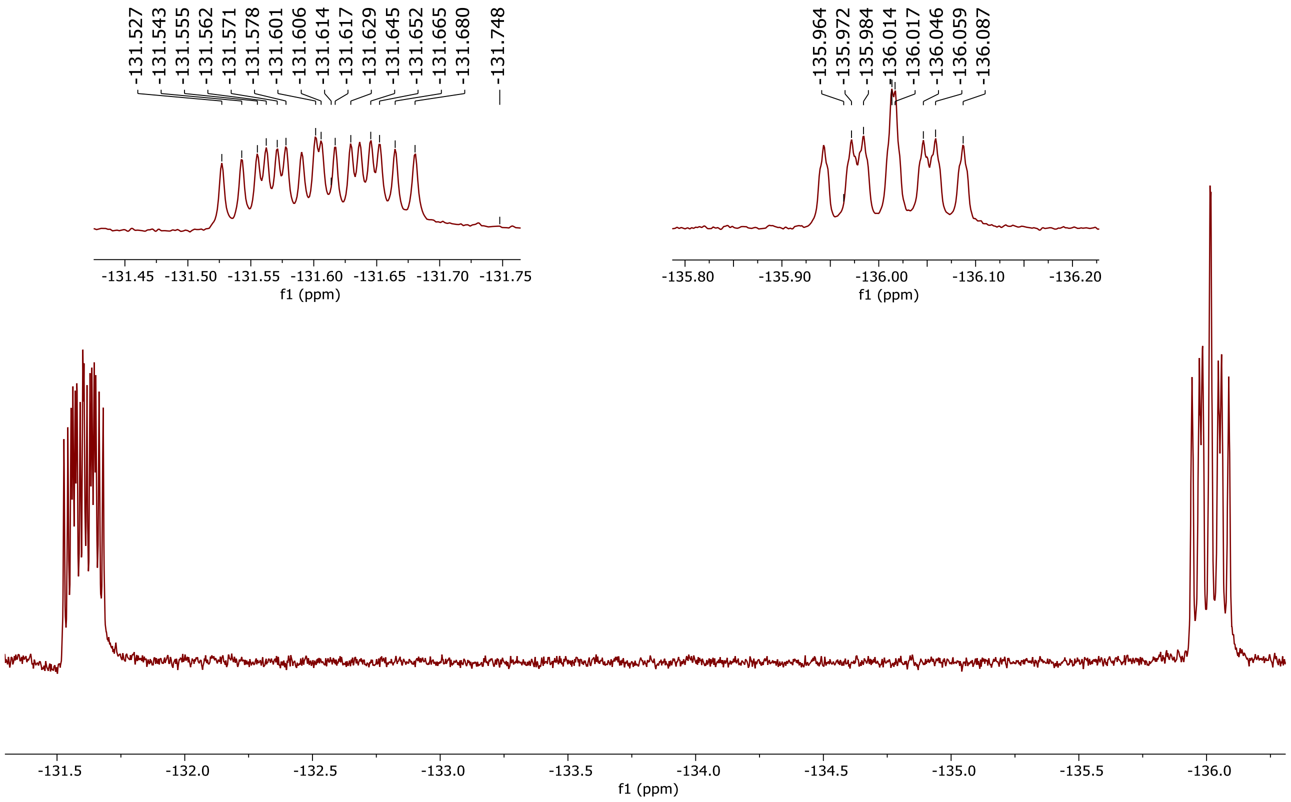

^{19}F-NMR spectrum (CDCl$_3$, 282.40 MHz) of **2g**

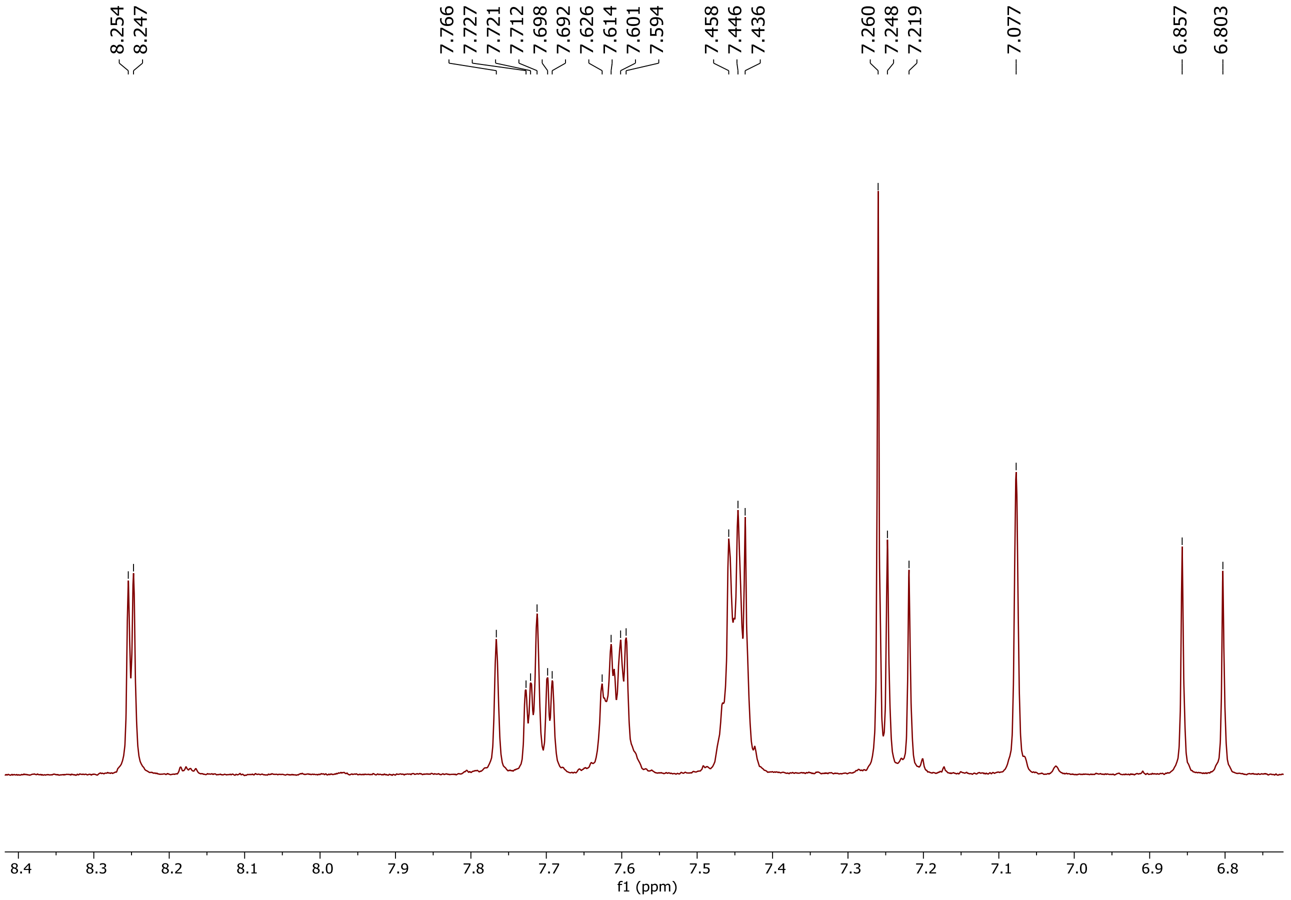

^{1}H{^{19}F}-NMR spectrum (CDCl$_3$, 300.13 MHz) of **2g**

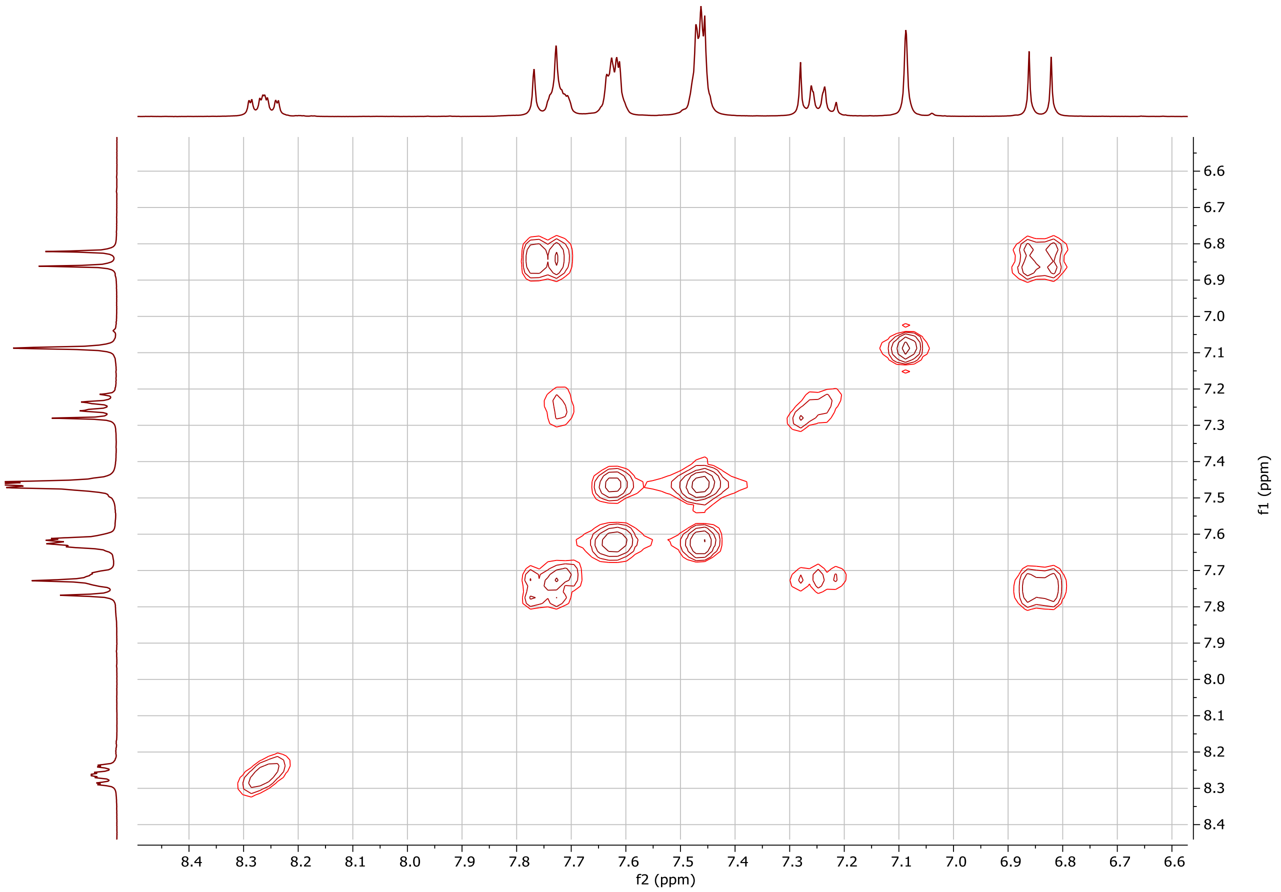

^1H-^1H COSY NMR spectrum of **2g**

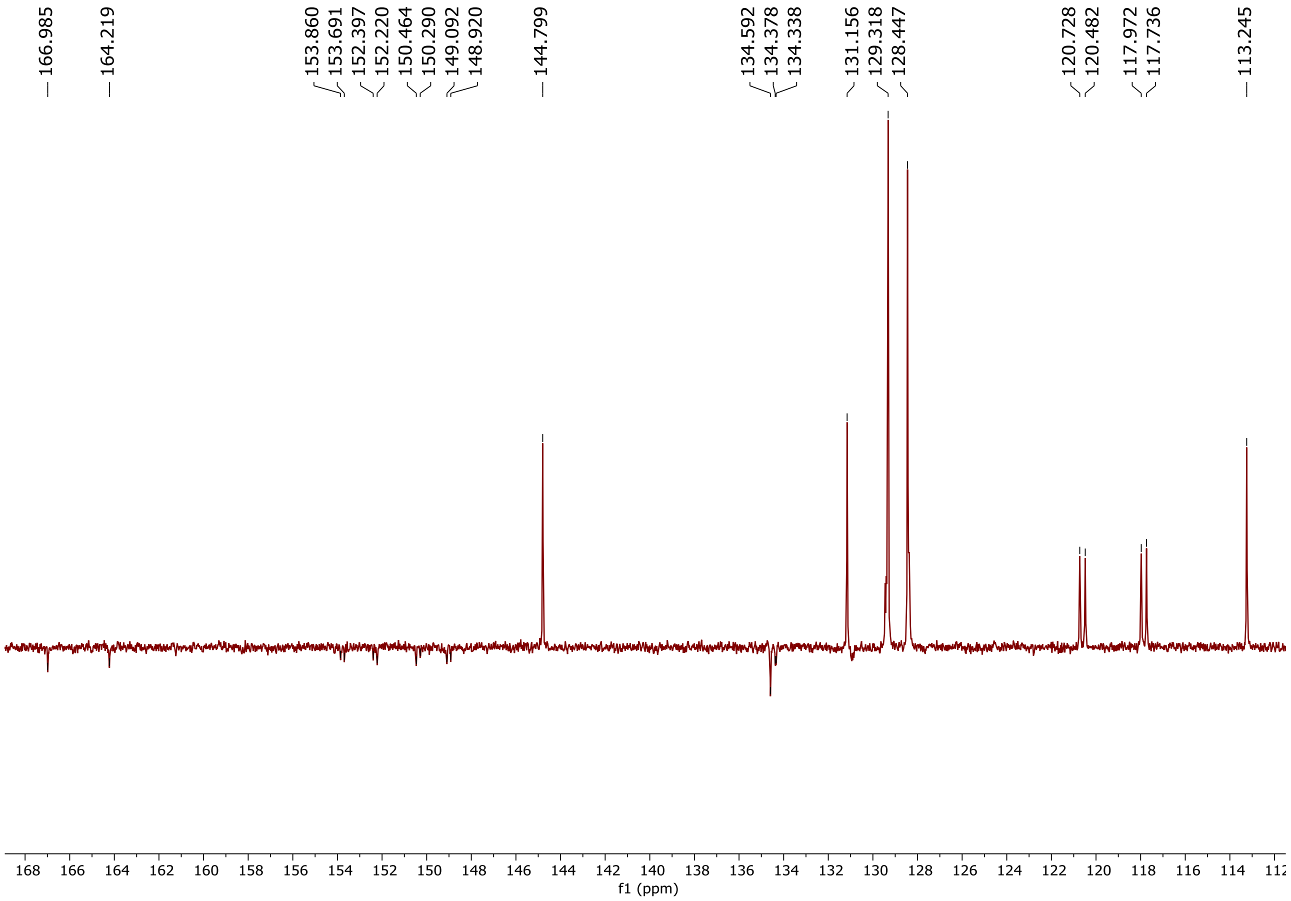

$^{13}C\{^1H\}$-(APT) NMR spectrum ($CDCl_3$, 75.47 MHz) of **2g**

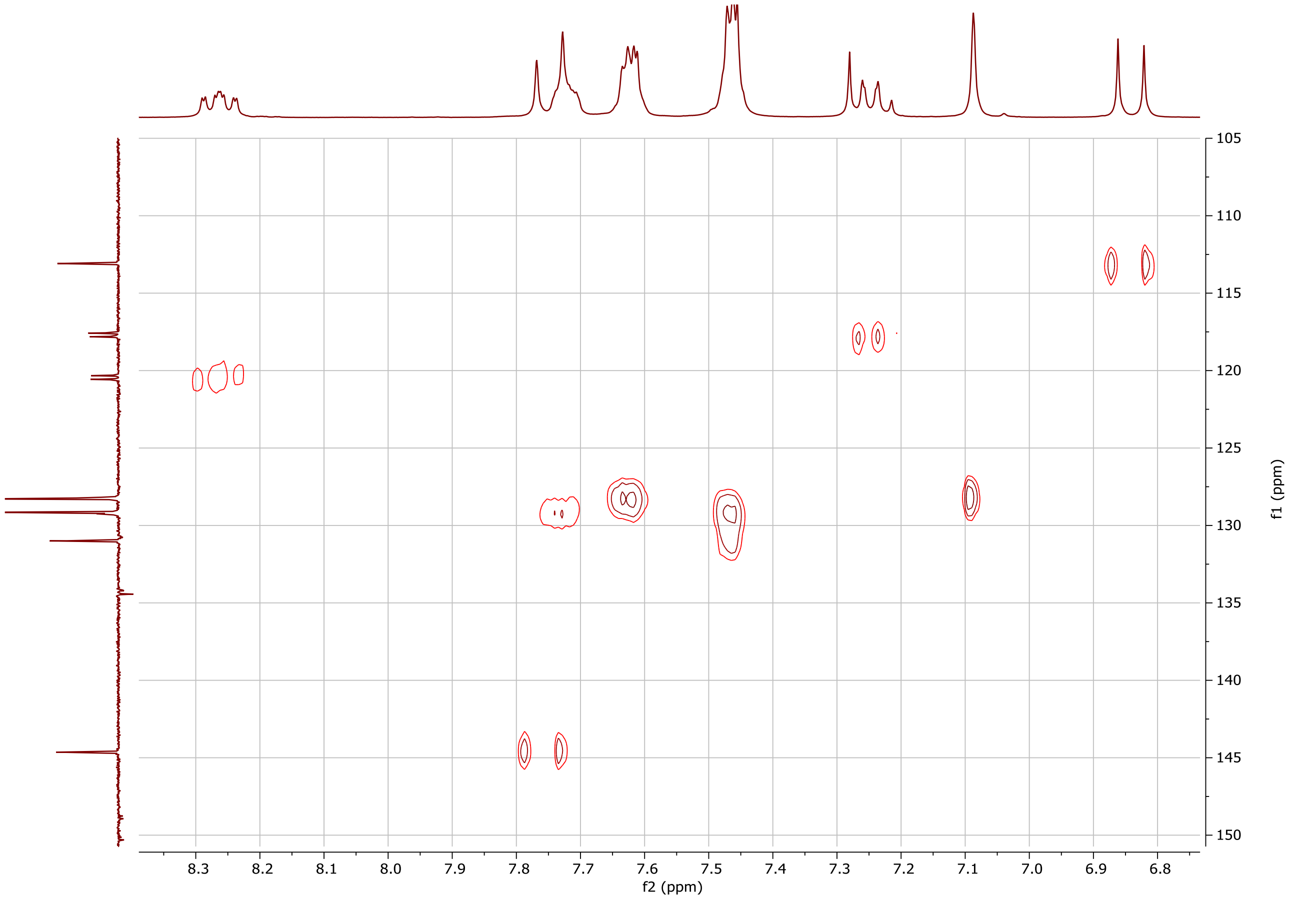

^{1}H-^{13}C HSQC NMR spectrum of **2g**

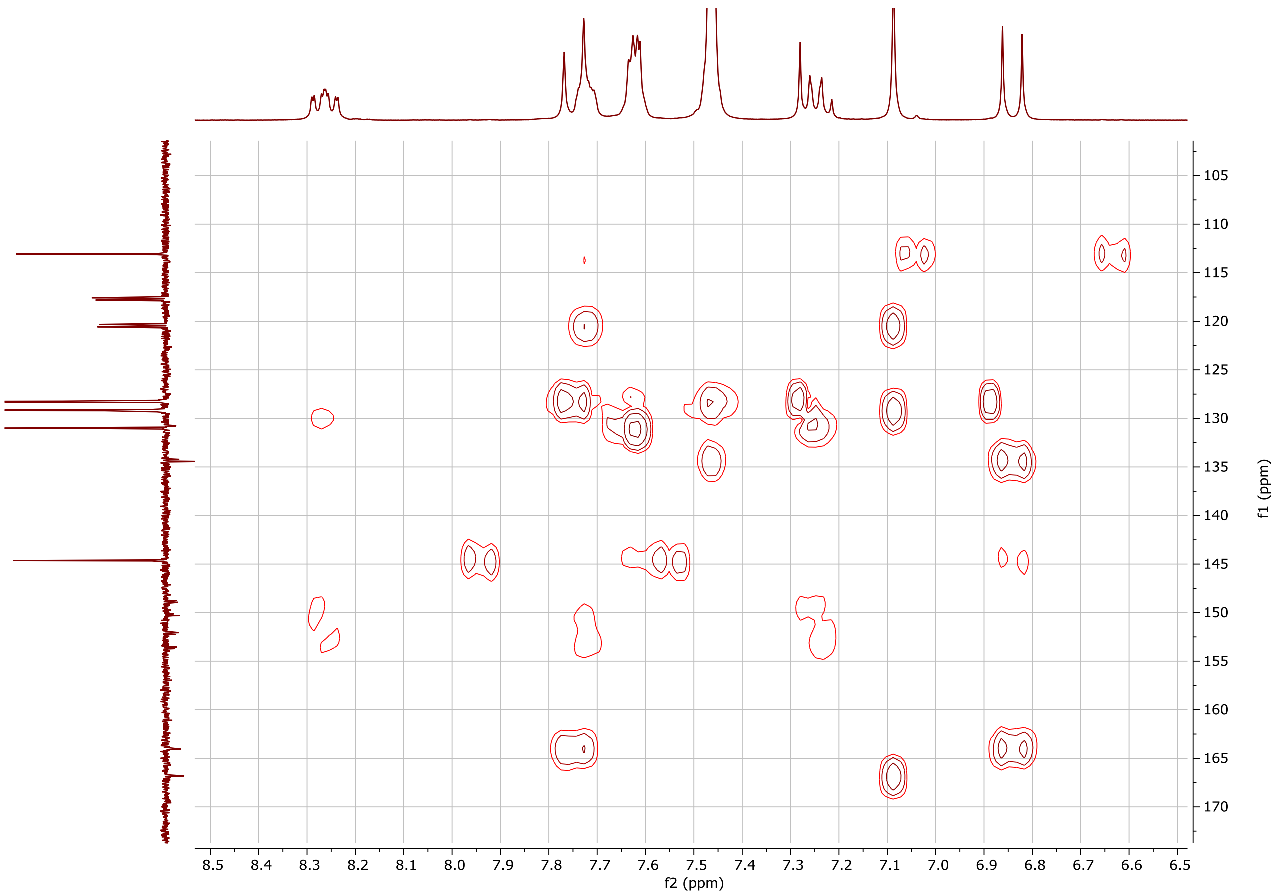

^{1}H-^{13}C HMBC NMR spectrum of **2g**

4-((*Z*)-3,4-Dichlorobenzylidene)-2-((*E*)-styryl)oxazol-5(4*H*)-one (2h)

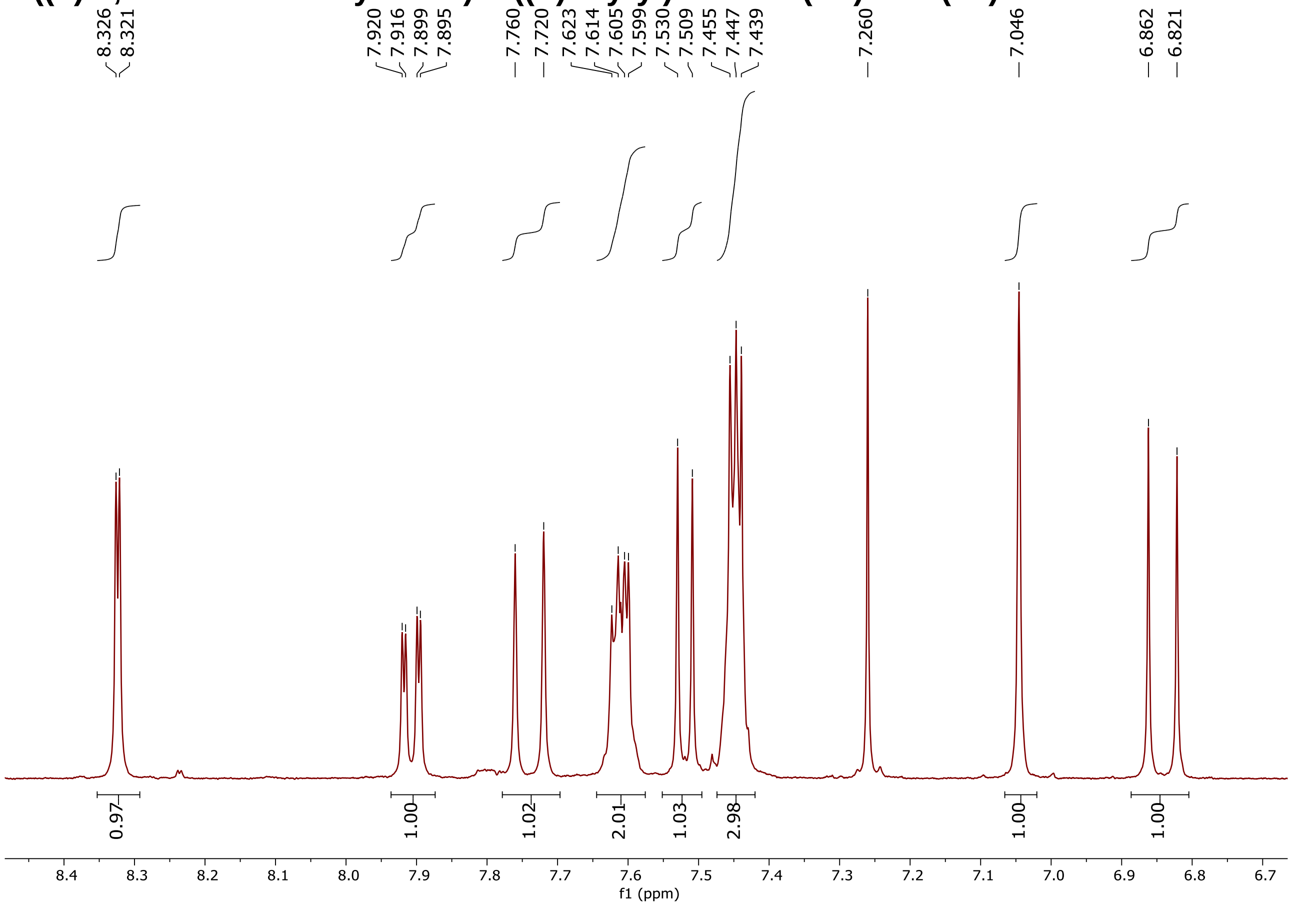

^{1}H NMR ($CDCl_3$, 400.13 MHz) of **2h**

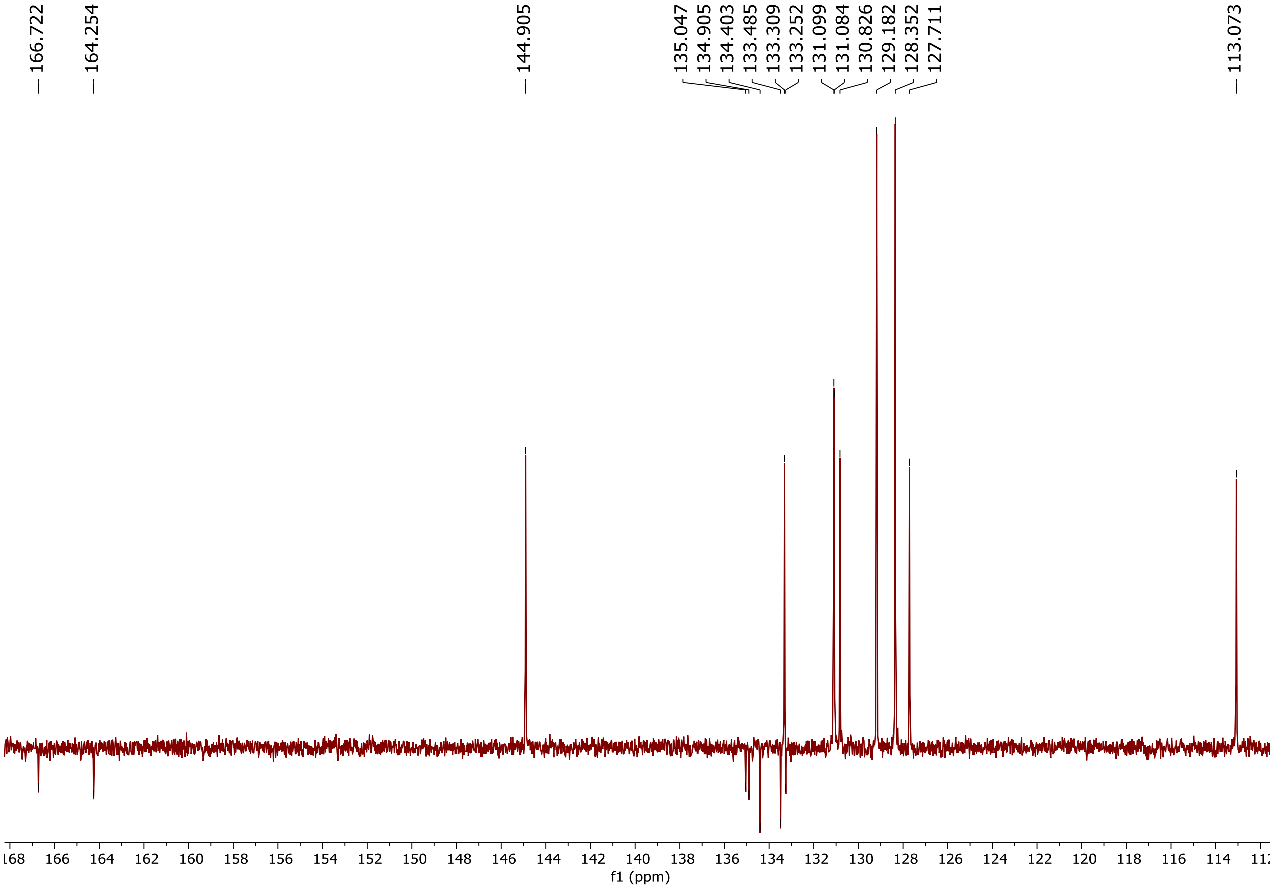

$^{13}C\{^{1}H\}$-(APT) NMR spectrum ($CDCl_3$, 75.47 MHz) of **2h**

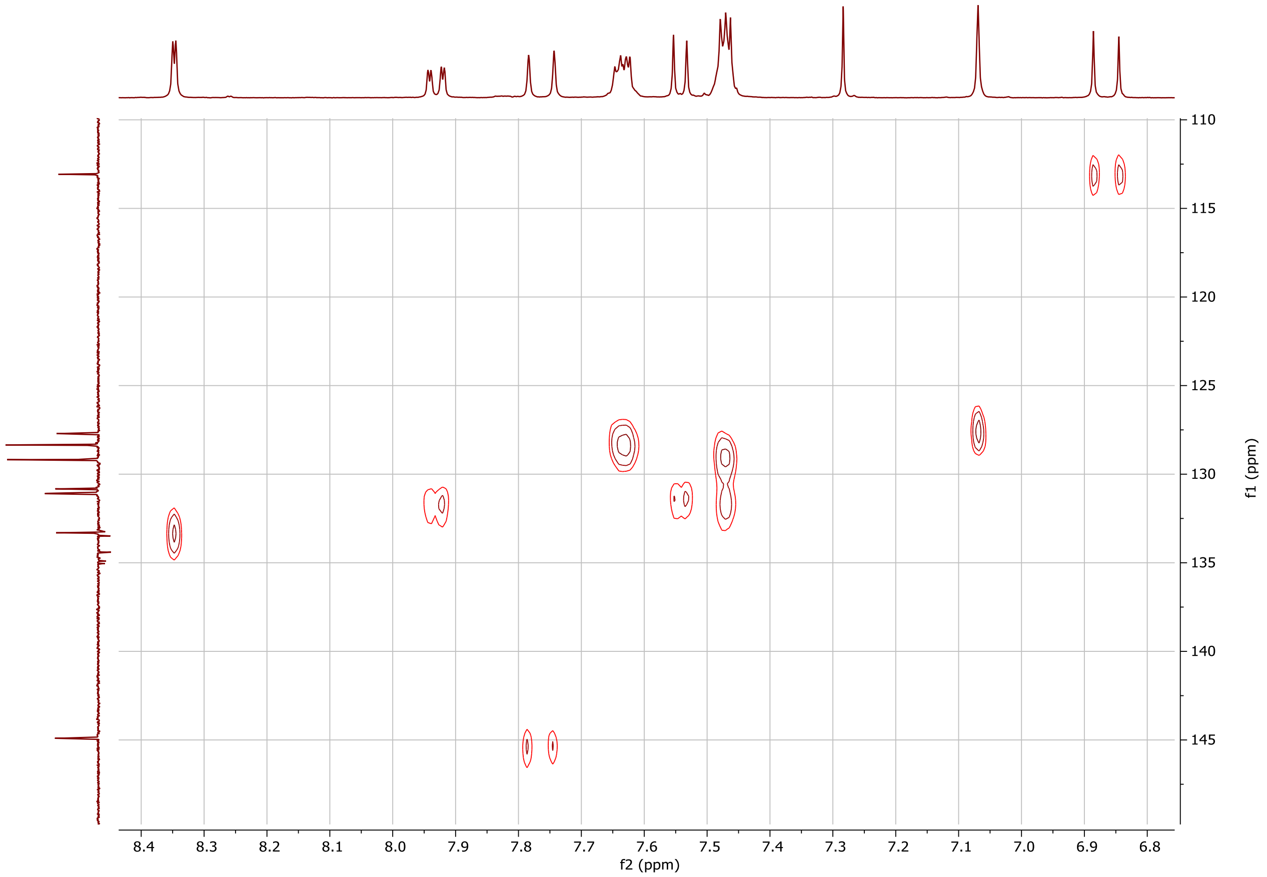

^{1}H-^{13}C HSQC NMR spectrum of **2h**

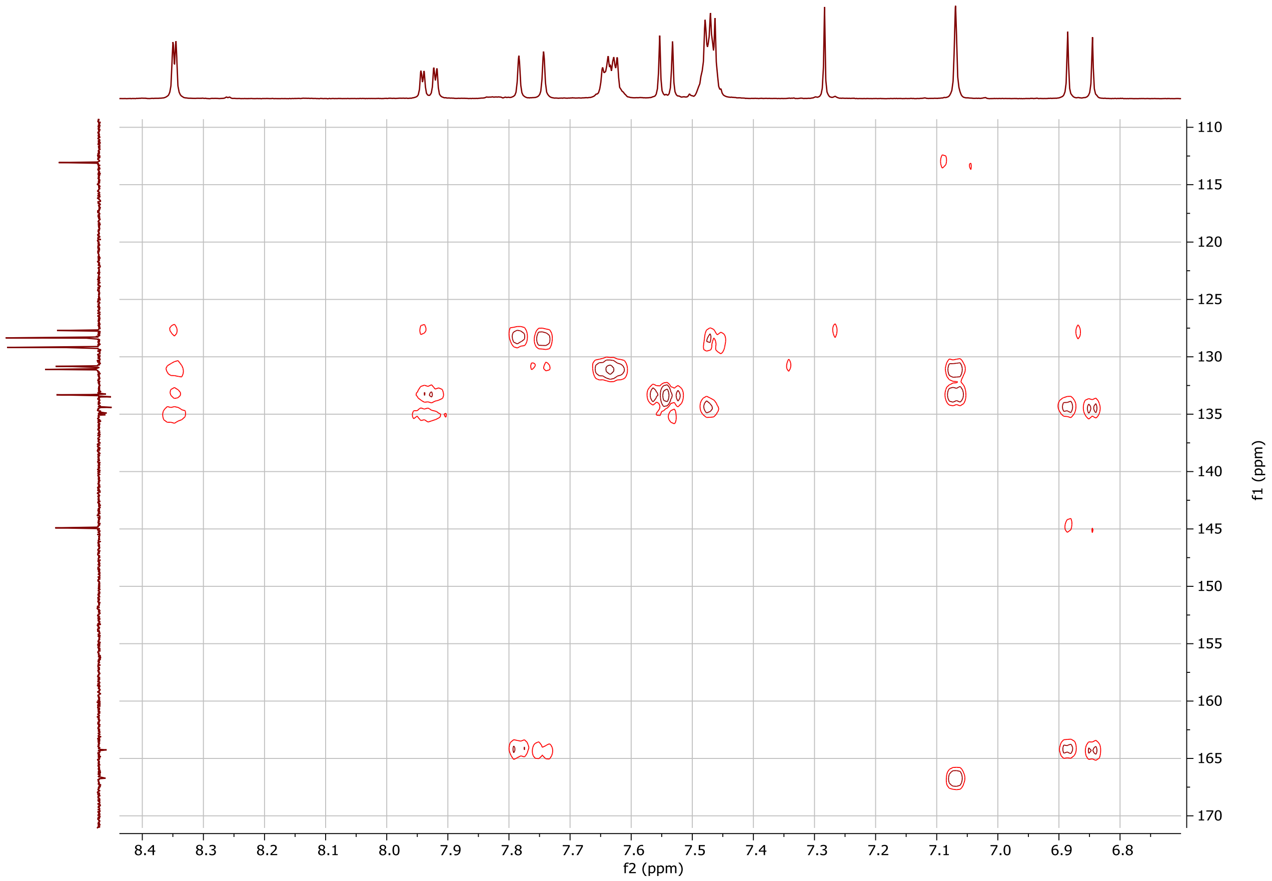

^{1}H-^{13}C HSQC NMR spectrum of **2h**

4-((*Z*)-2-Nitrobenzylidene)-2-((*E*)-styryl)oxazol-5(4*H*)-one (2i)

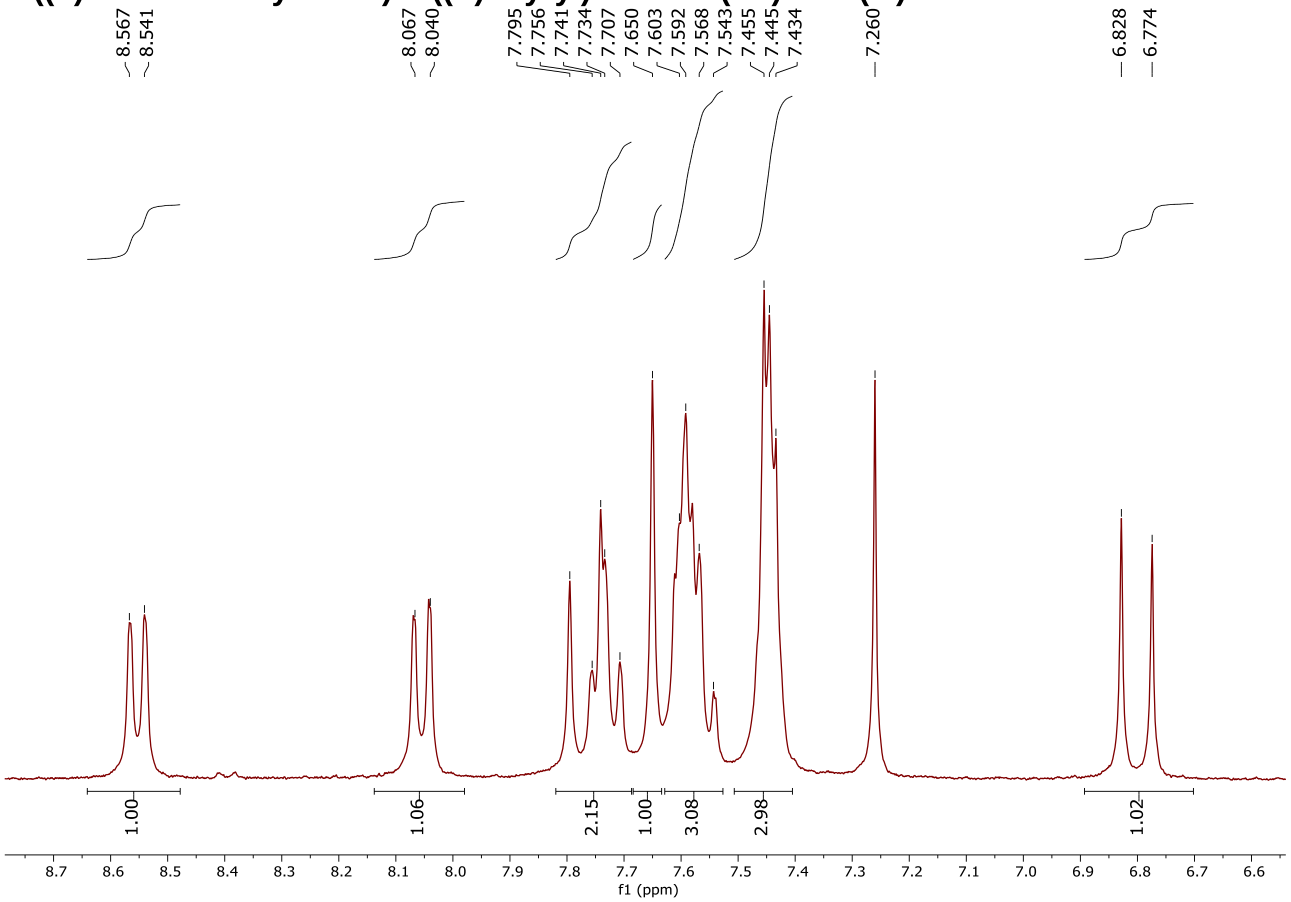

^{1}H NMR (CDCl$_3$, 300.13 MHz) of **2i**

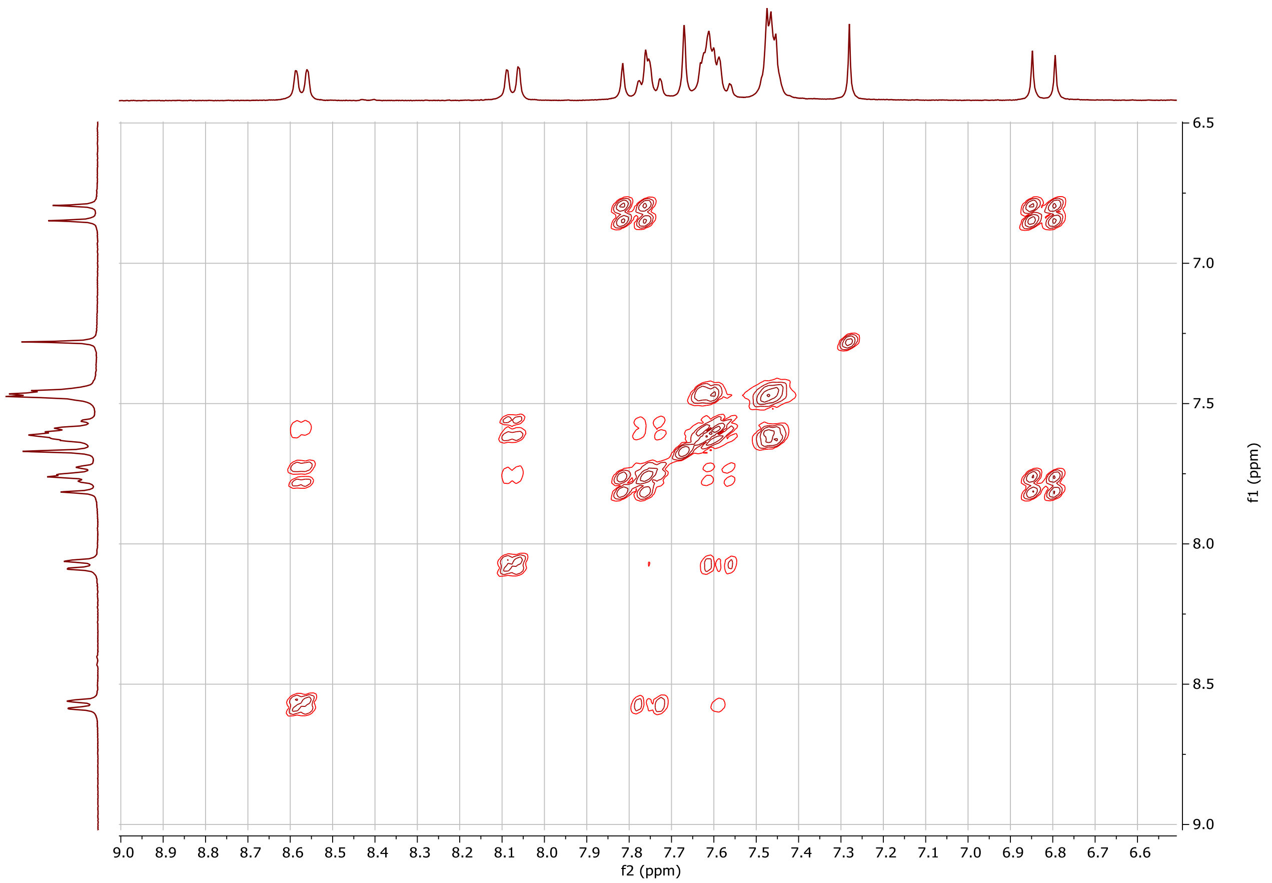

^{1}H-^{1}H COSY NMR spectrum of **2i**

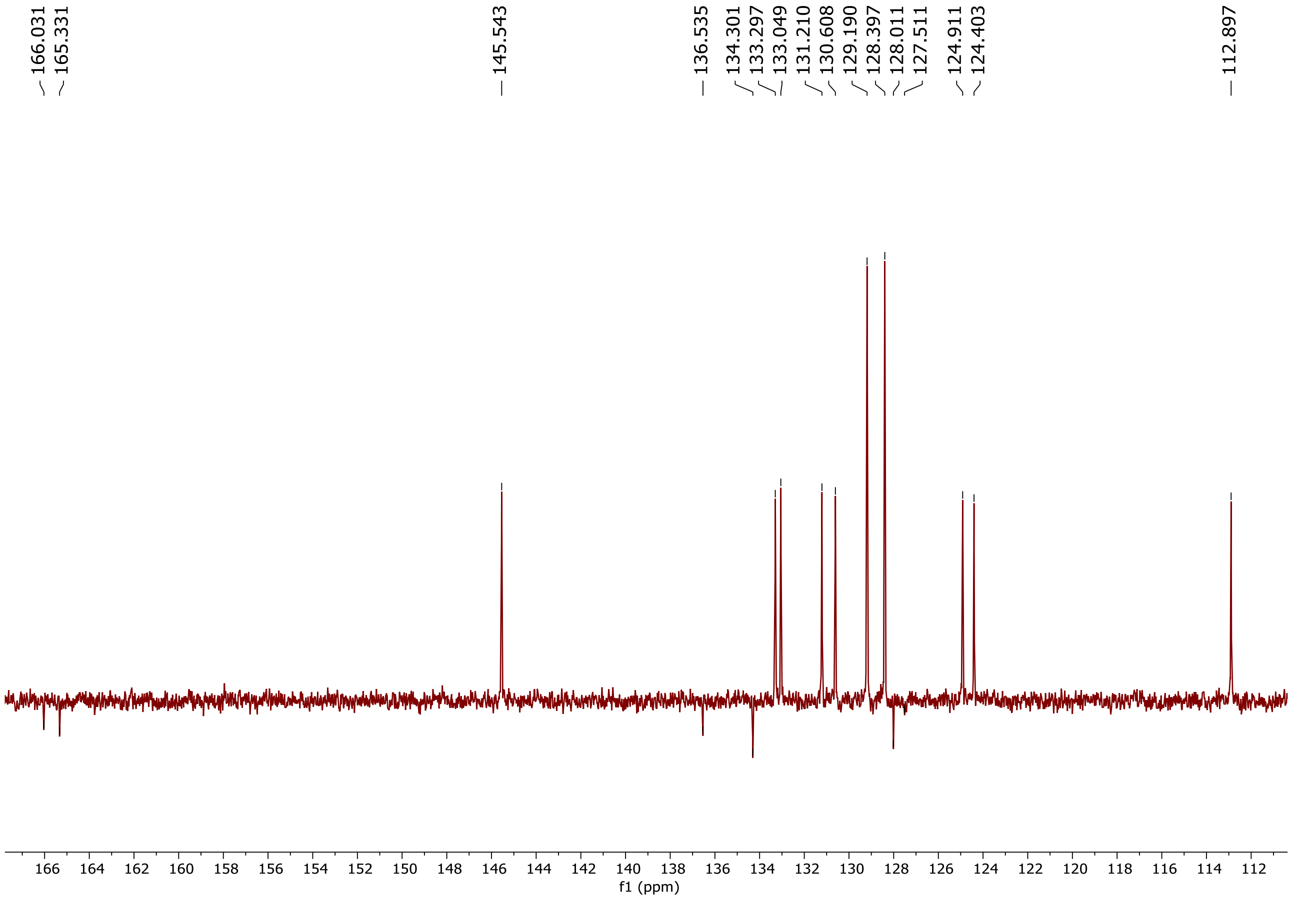

$^{13}C\{^{1}H\}$-(APT) NMR spectrum ($CDCl_3$, 75.47 MHz) of **2i**

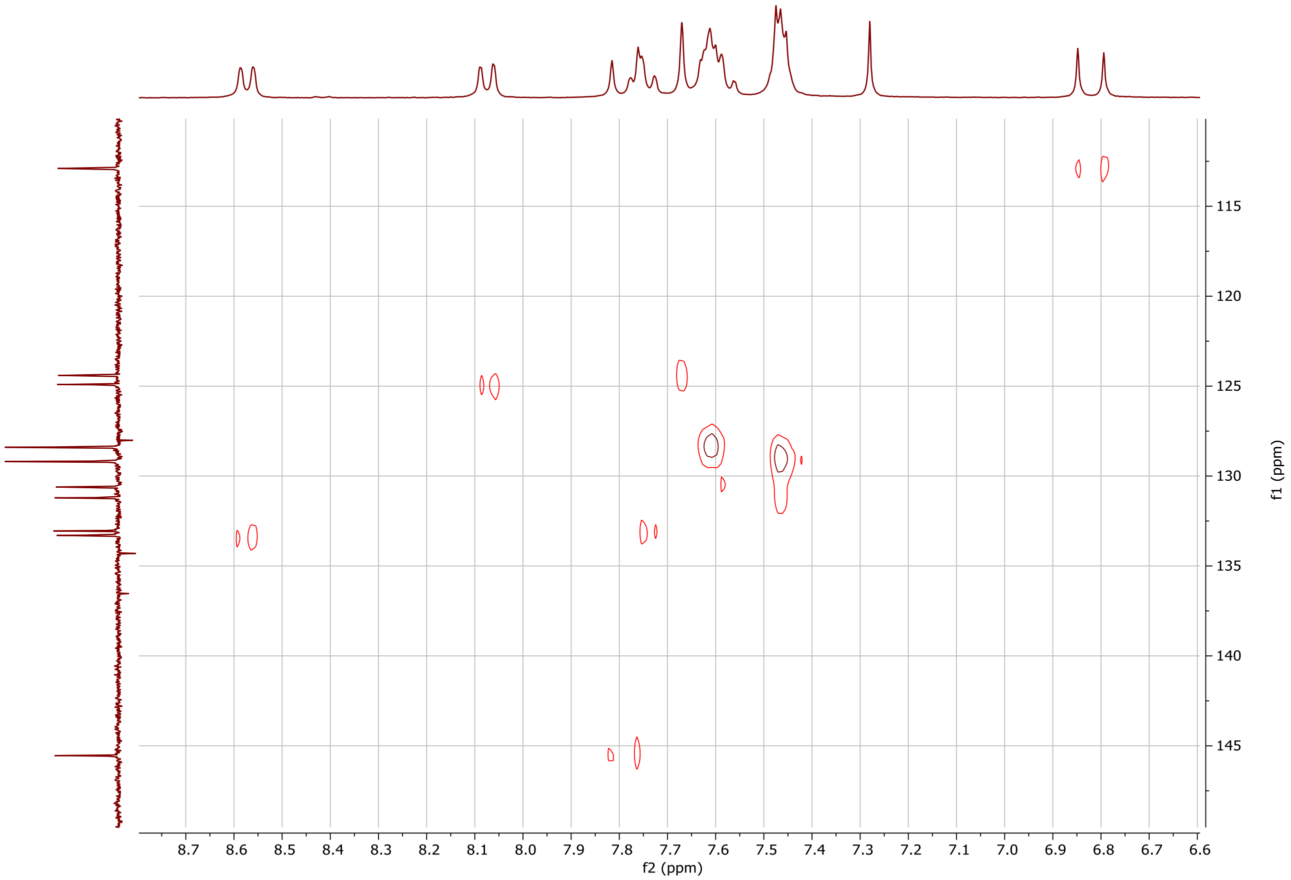

^{1}H-^{13}C HSQC NMR spectrum of **2i**

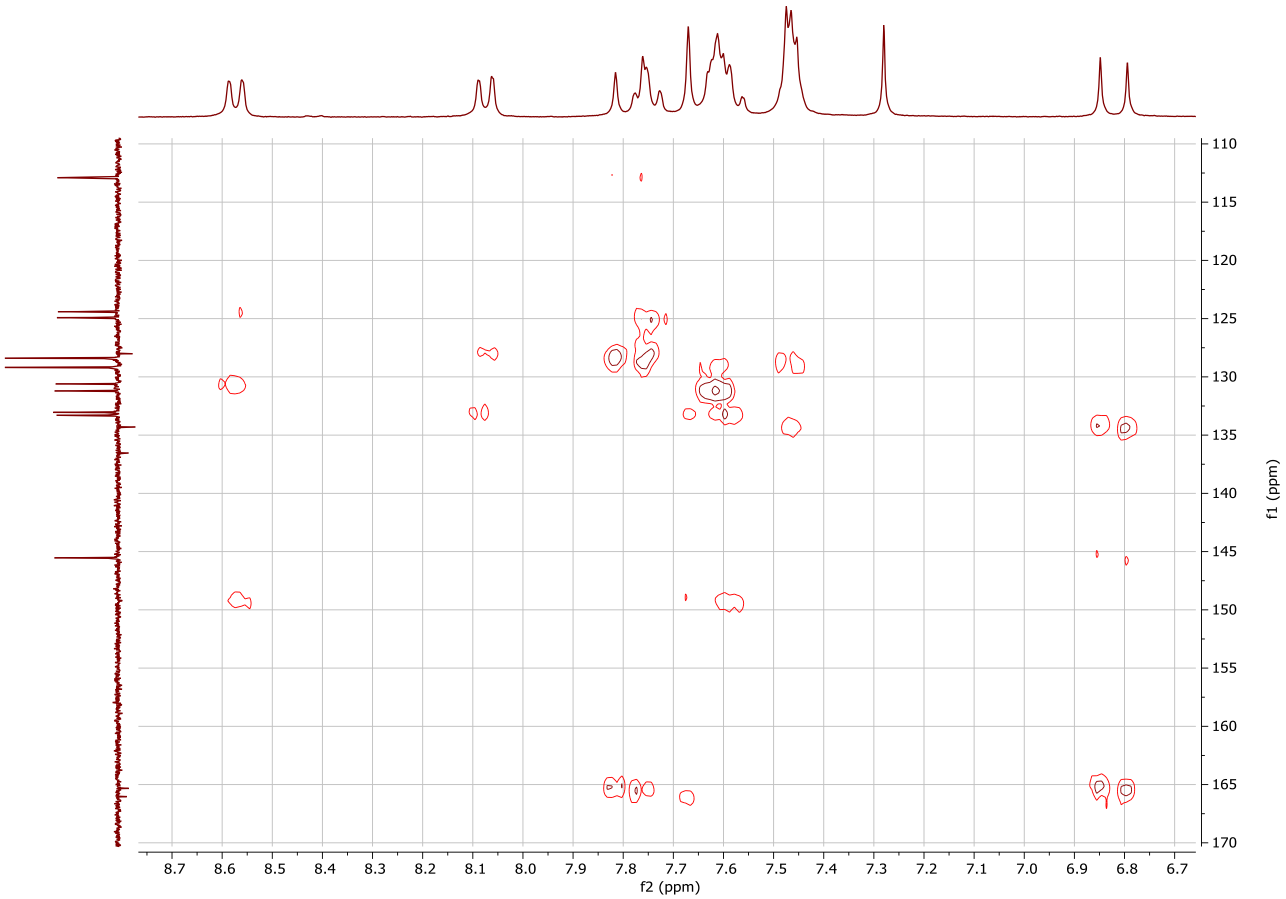

^{1}H-^{13}C HMBC NMR spectrum of **2i**

4-((*Z*)-4-Nitrobenzylidene)-2-((*E*)-styryl)oxazol-5(4*H*)-one (2j)

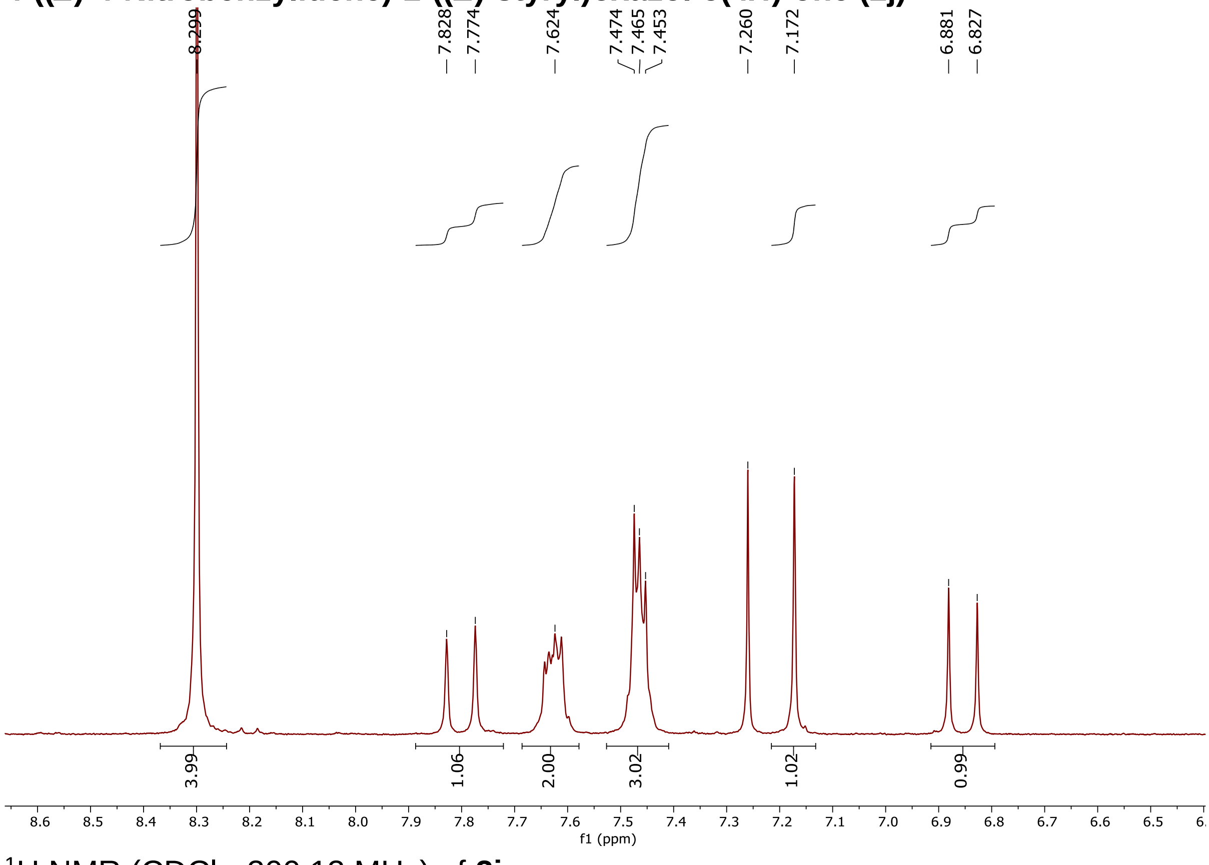

^{1}H NMR ($CDCl_3$, 300.13 MHz) of **2j**

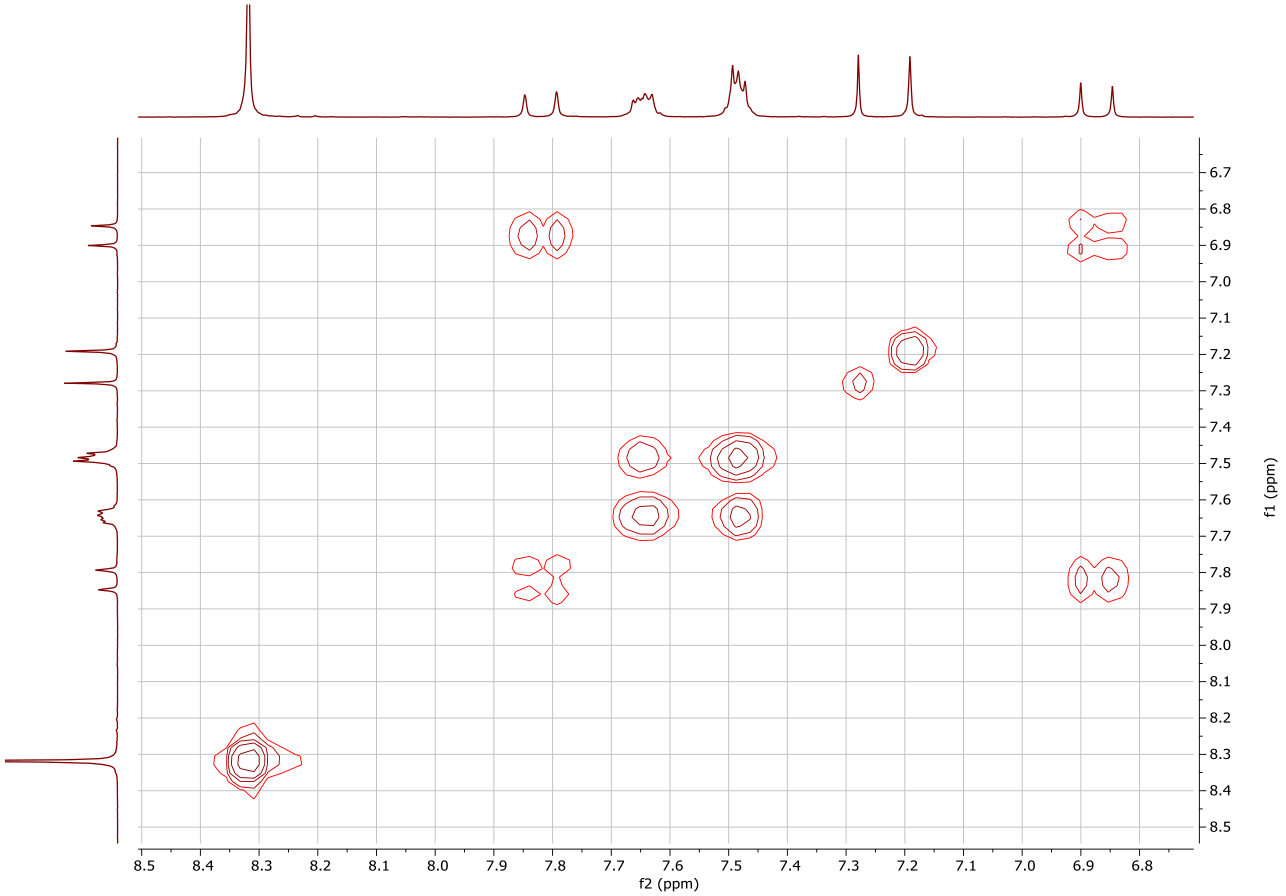

^{1}H-^{1}H COSY NMR spectrum of **2j**

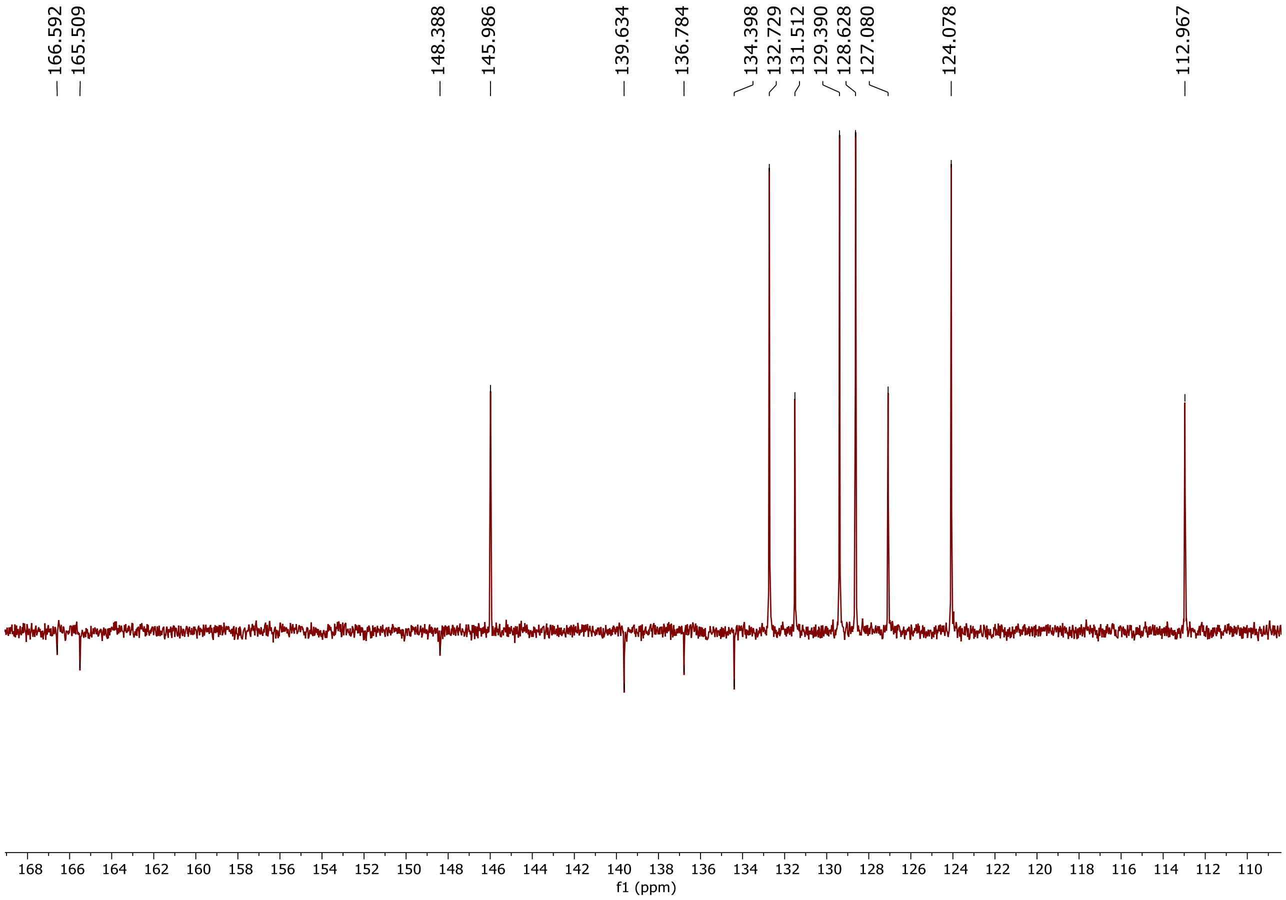

^{13}C{^{1}H}-(APT) NMR spectrum ($CDCl_3$, 75.47 MHz) of **2j**

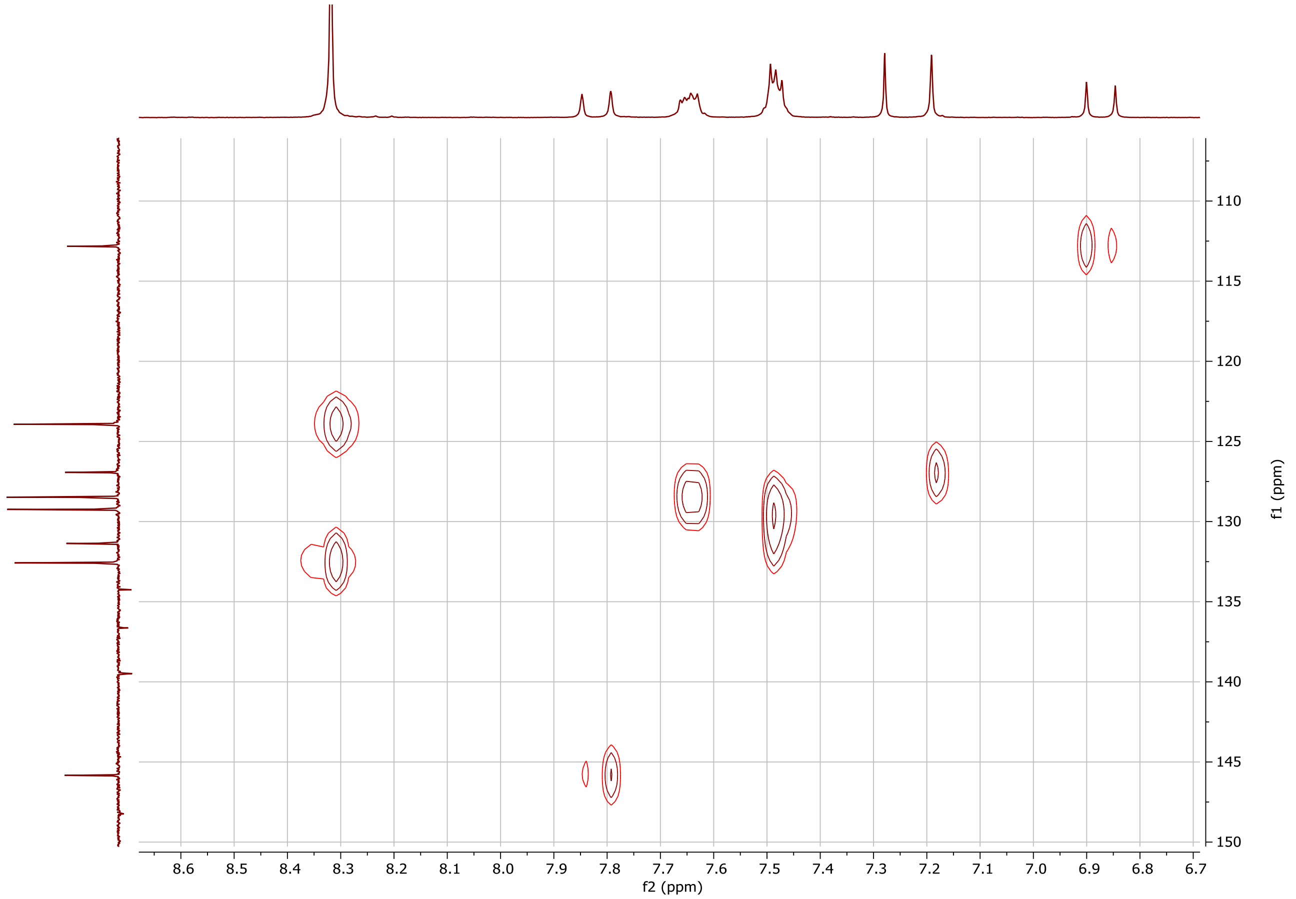

^{1}H-^{13}C HSQC NMR spectrum of **2j**

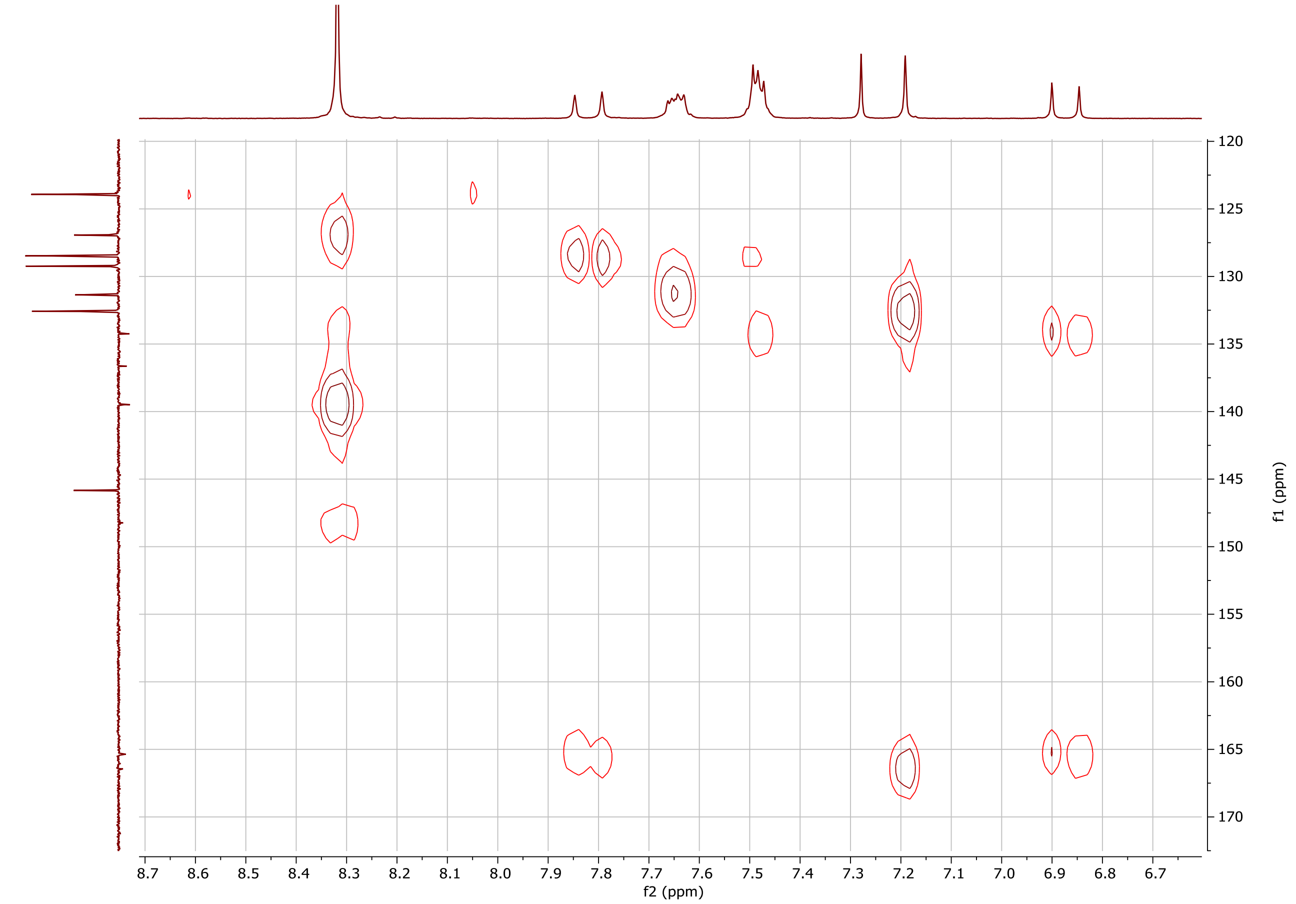

^{1}H-^{13}C HMBC NMR spectrum of **2j**

2. NMR spectra of orthopalladated dinuclear complexes 3a–f, 3h–j.

Orthopalladated dinuclear derivative 3a

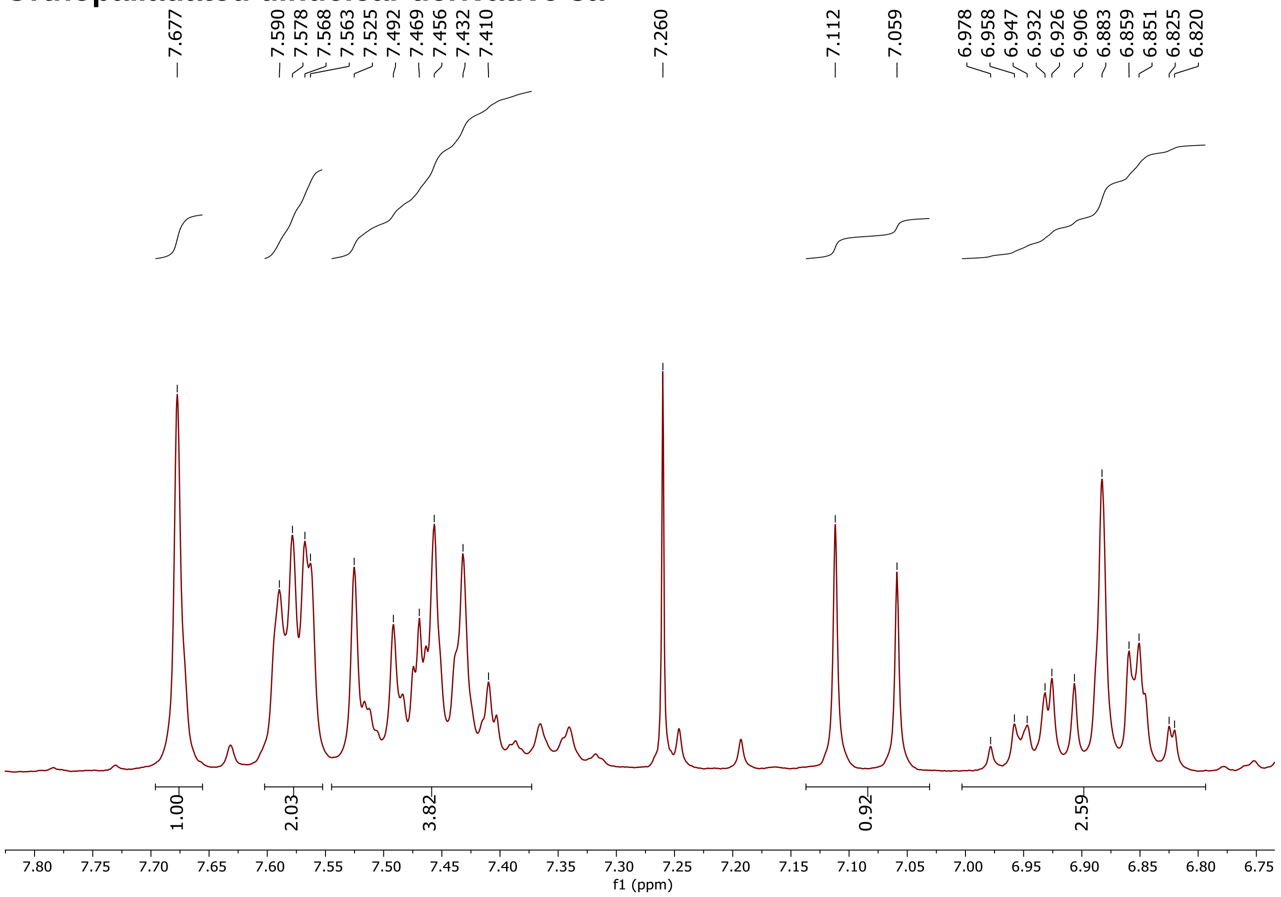

^{1}H NMR ($CDCl_3$, 300.13 MHz) of **3a**

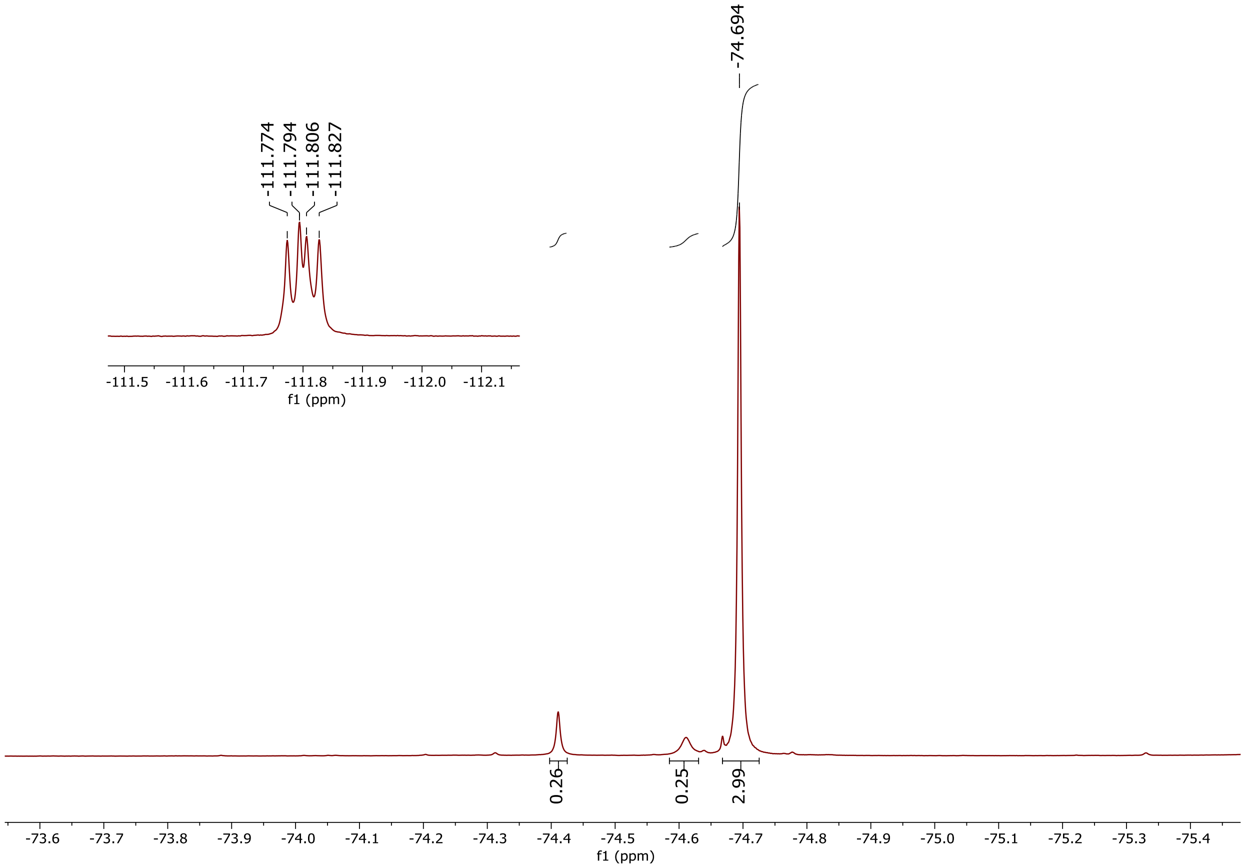

^{19}F-NMR spectrum ($CDCl_3$, 282.40 MHz) of **3a**

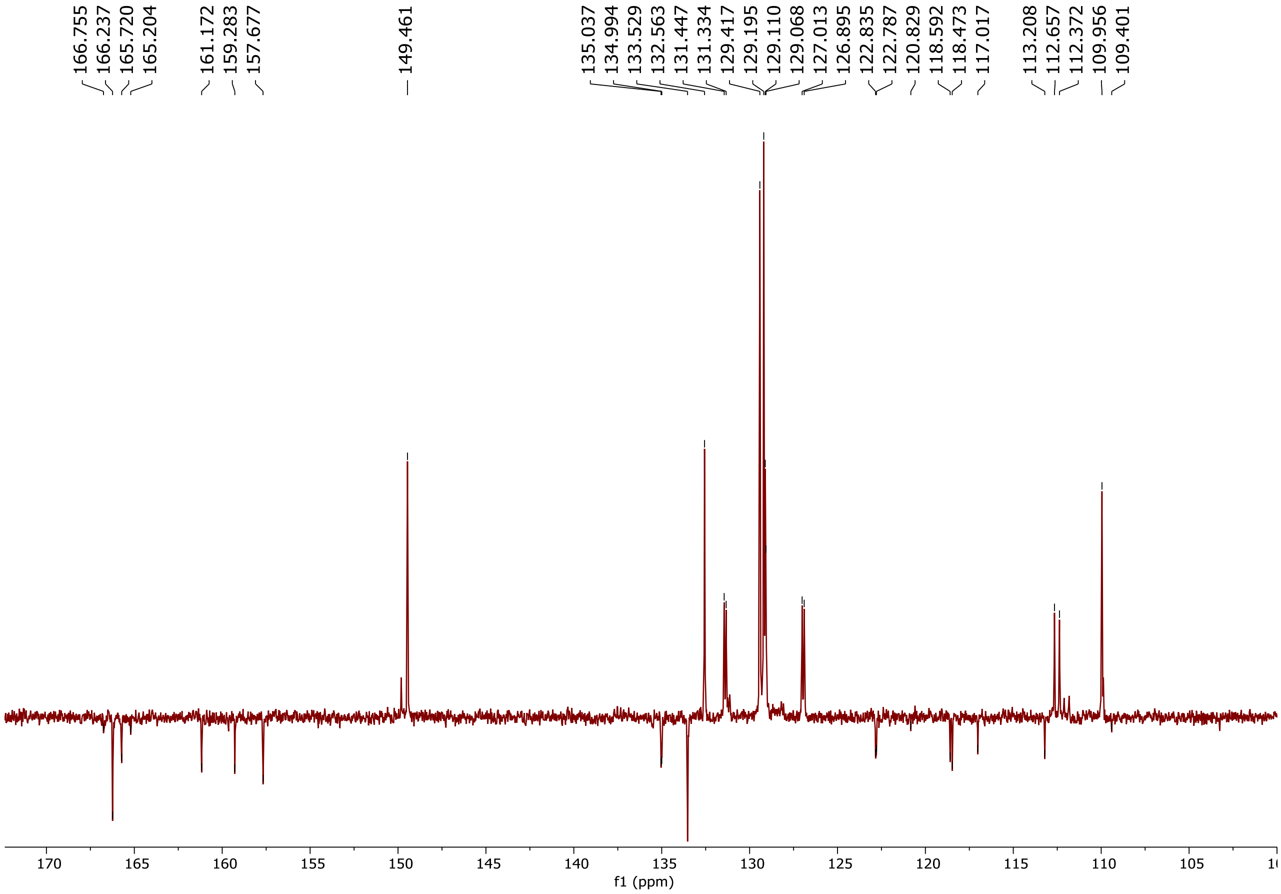

$^{13}C\{^{1}H\}$-(APT) NMR spectrum ($CDCl_3$, 75.47 MHz) of **3a**

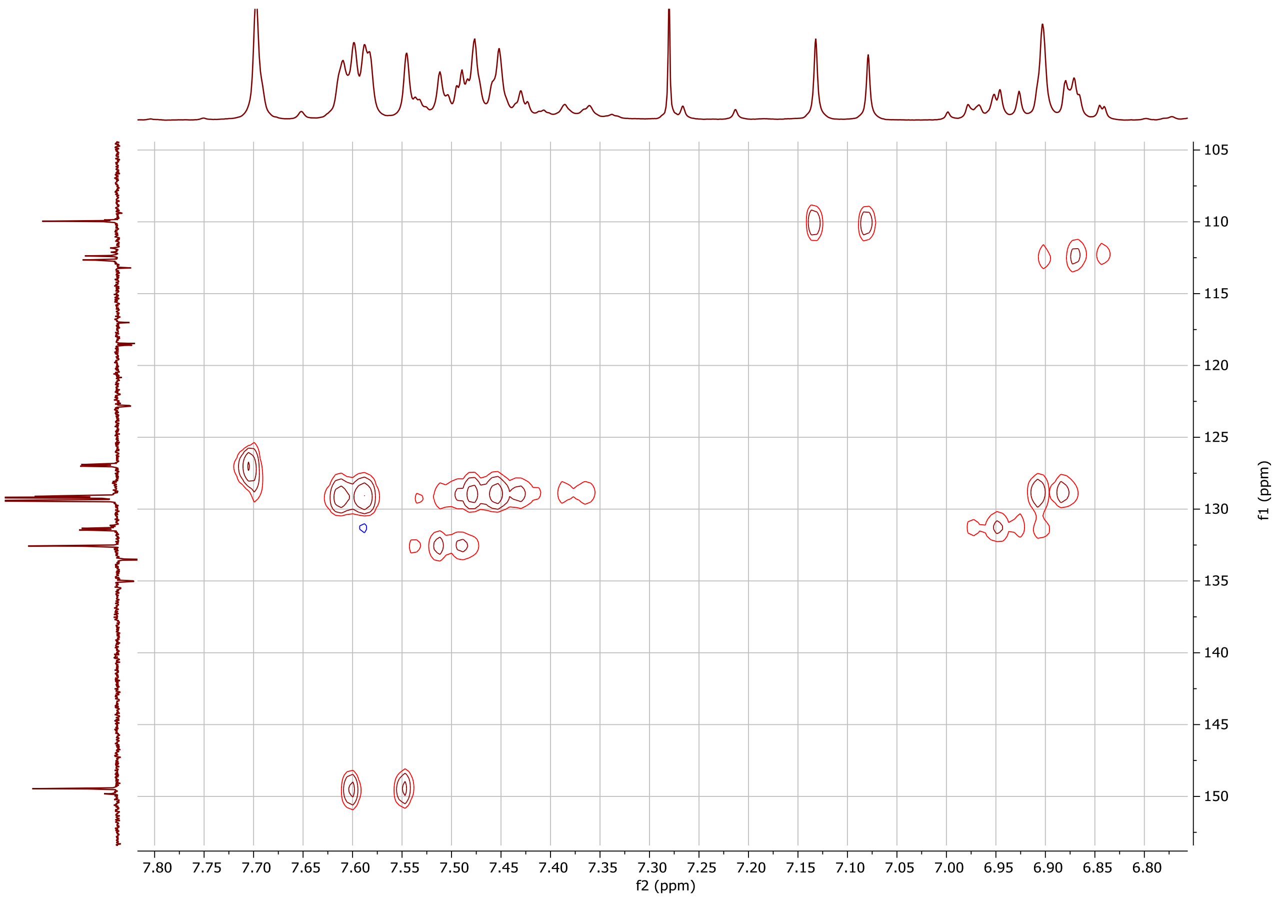

^{1}H-^{13}C HSQC NMR spectrum of **3a**

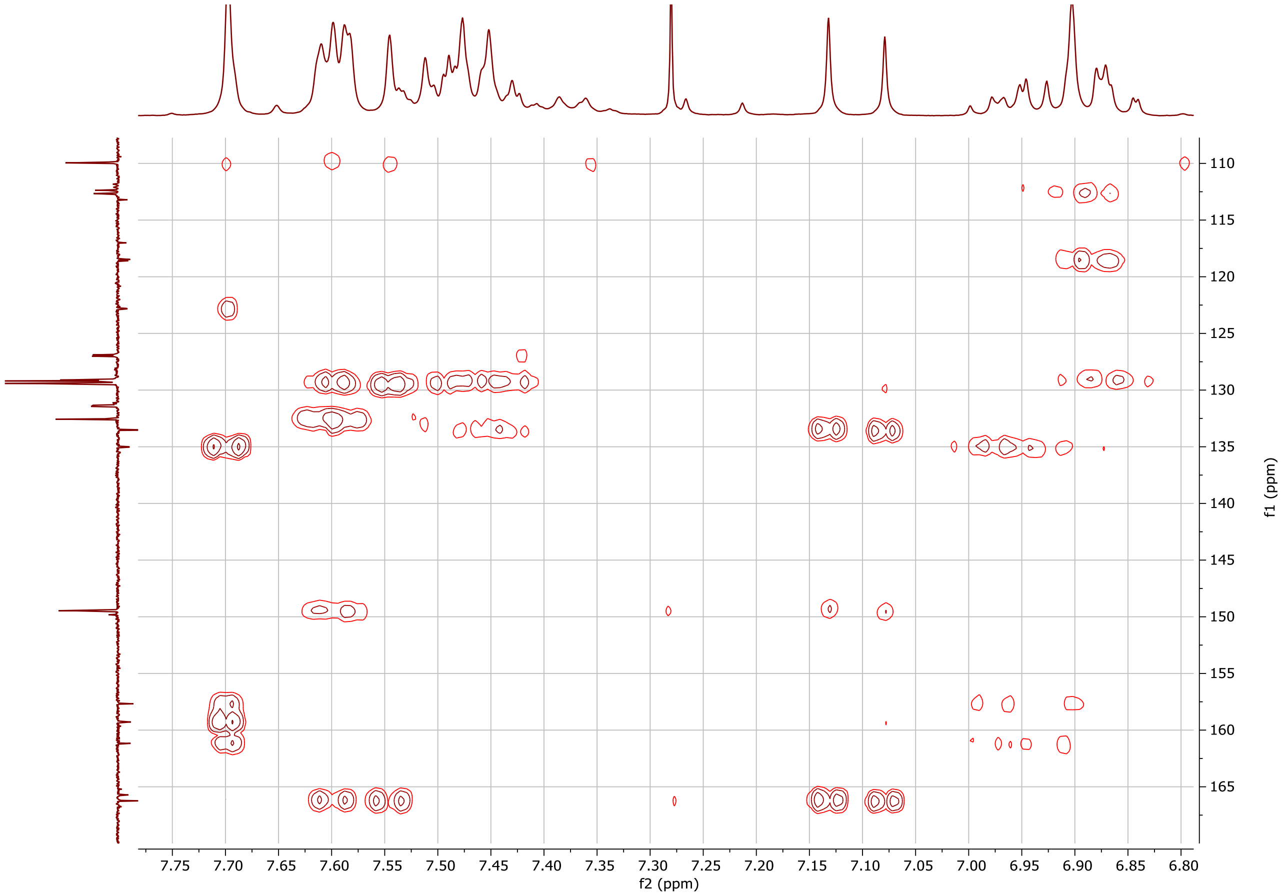

^{1}H-^{13}C HMBC NMR spectrum of **3a**

Orthopalladated dinuclear derivative 3b

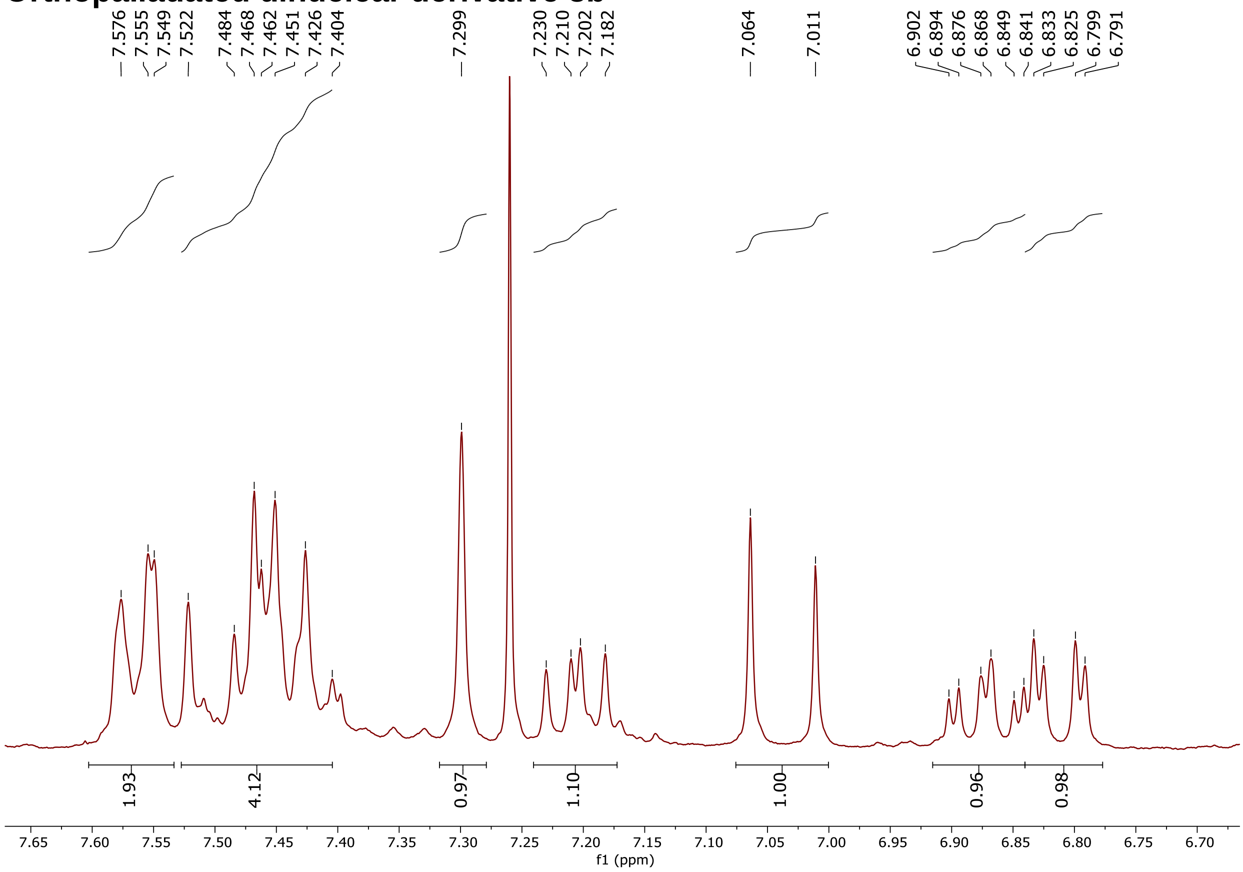

^{1}H NMR ($CDCl_3$, 300.13 MHz) of **3b**

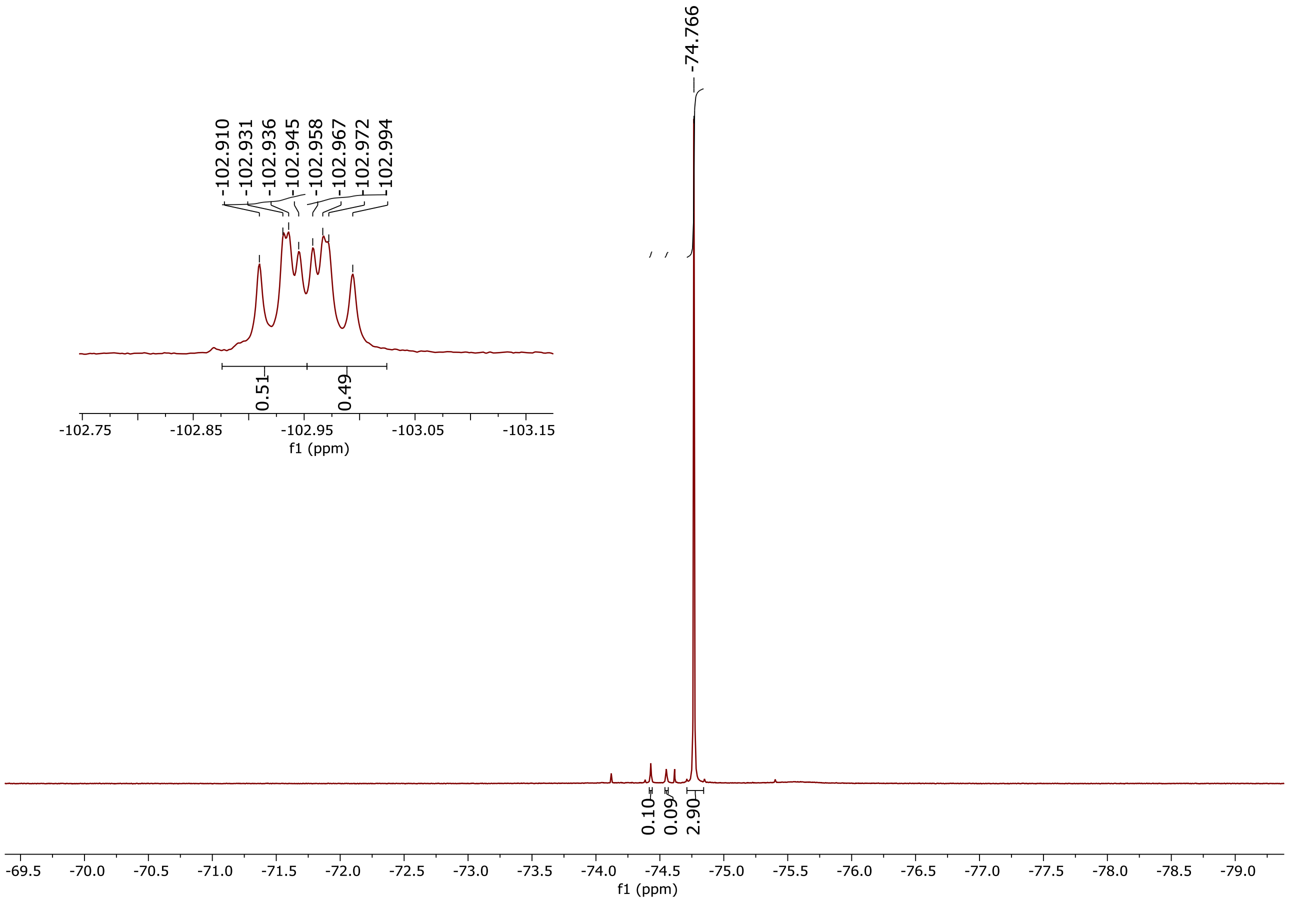

^{19}F-NMR spectrum ($CDCl_3$, 282.40 MHz) of **3b**

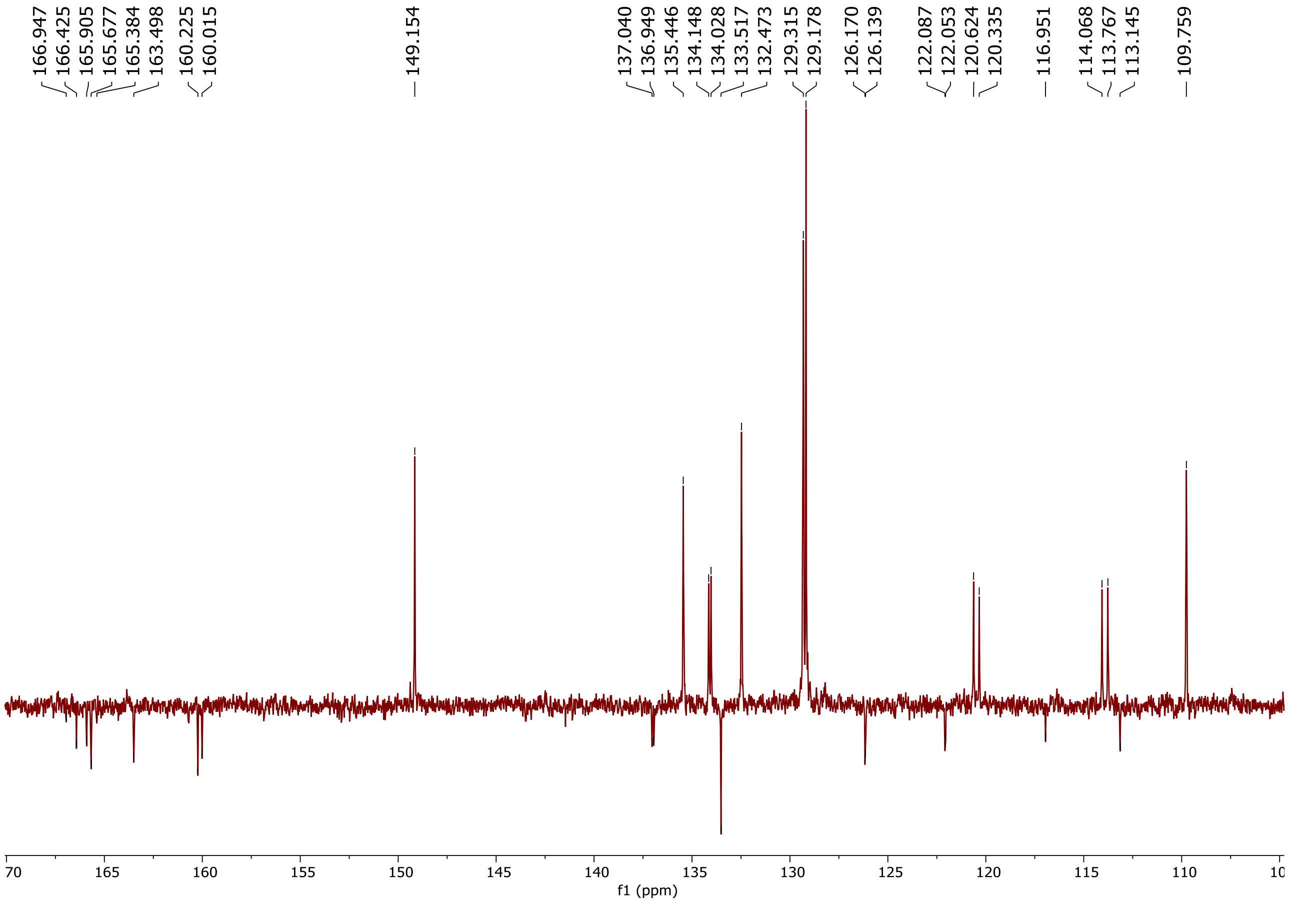

^{13}C{^{1}H}-(APT) NMR spectrum ($CDCl_3$, 75.47 MHz) of **3b**

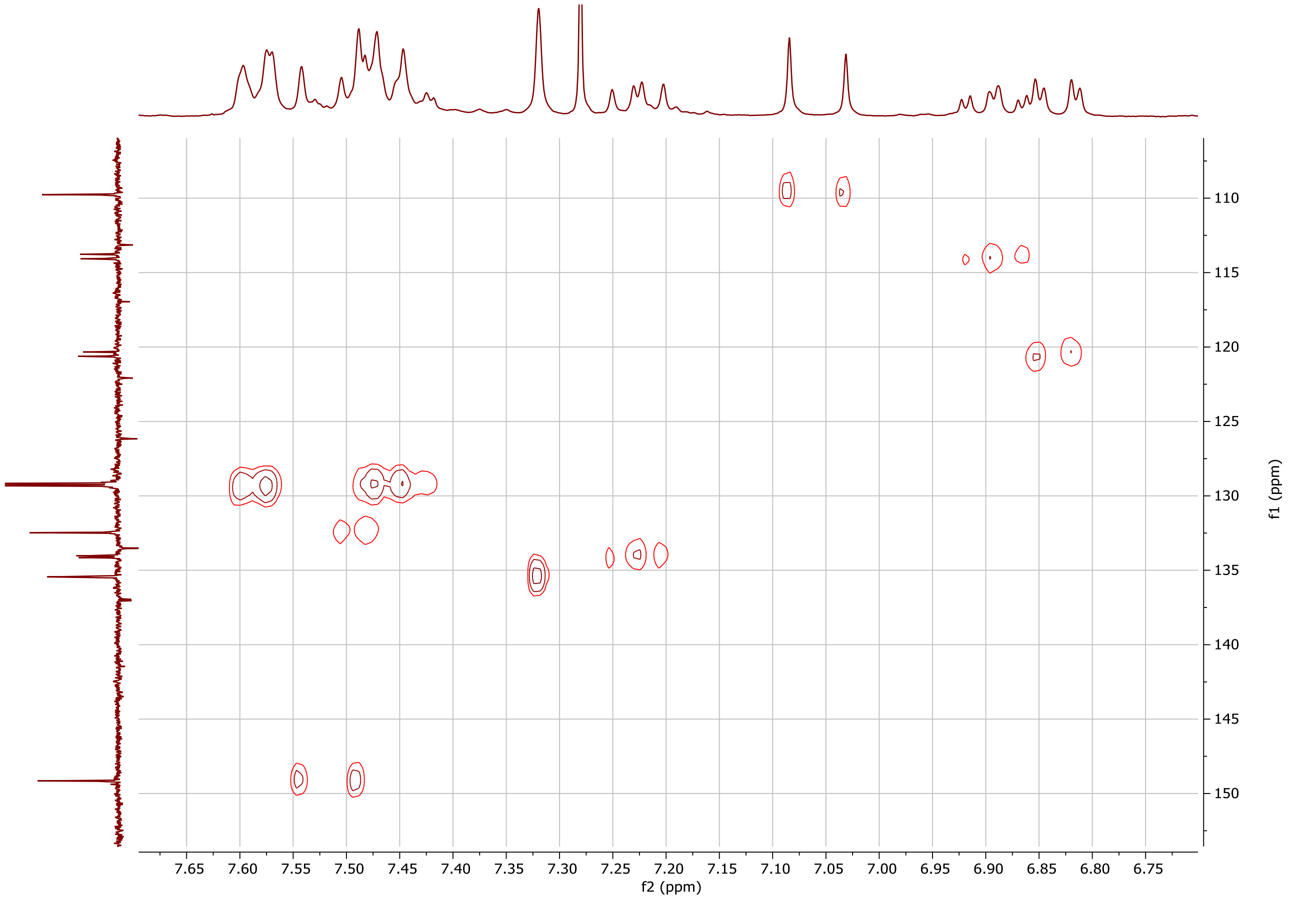

^{1}H-^{13}C HSQC NMR spectrum of **3b**

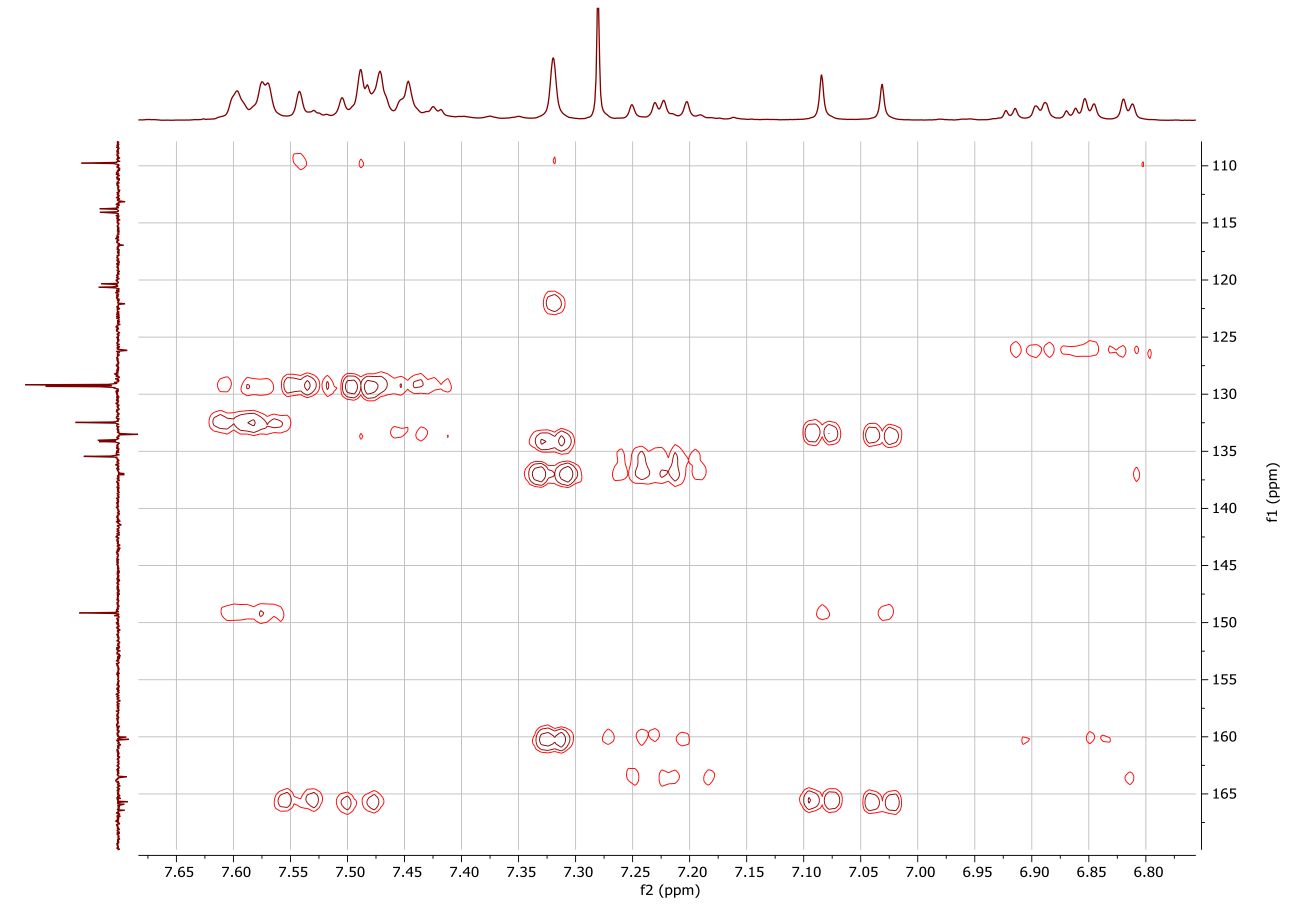

^{1}H-^{13}C HMBC NMR spectrum of **3b**

Orthopalladated dinuclear derivative 3c

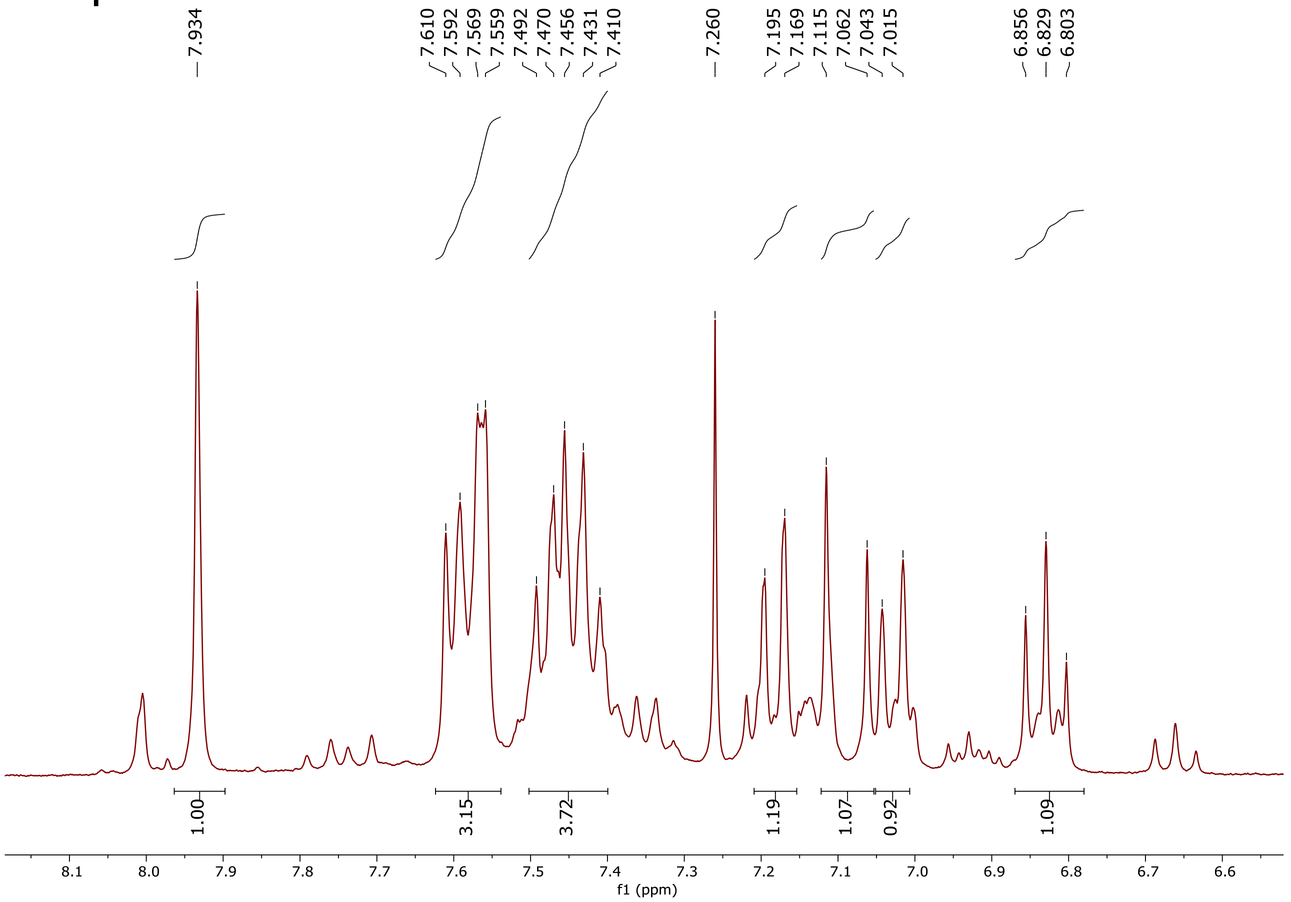

^{1}H NMR ($CDCl_3$, 300.13 MHz) of **3c**

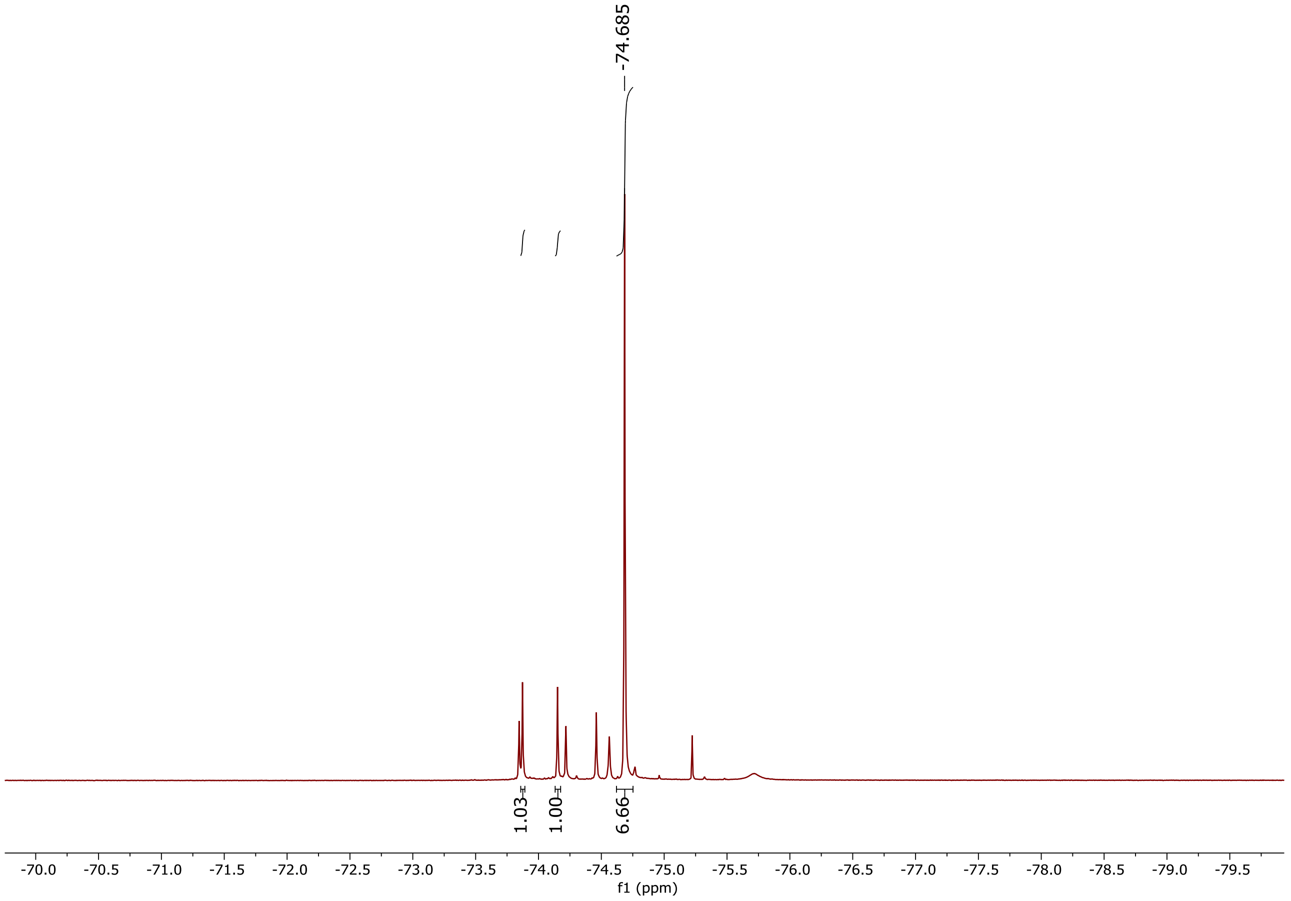

^{19}F-NMR spectrum ($CDCl_3$, 282.40 MHz) of **3c**

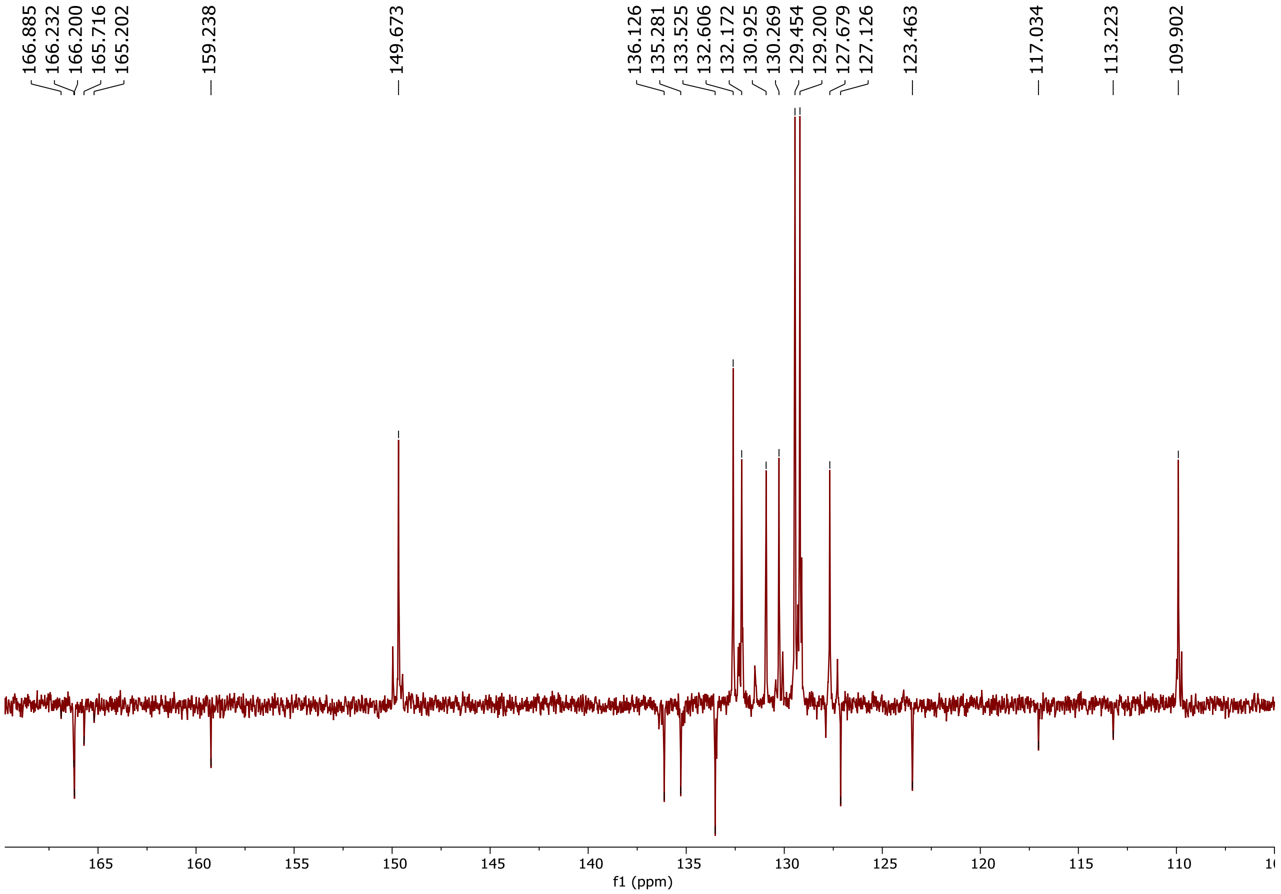

$^{13}C\{^{1}H\}$-(APT) NMR spectrum ($CDCl_3$, 75.47 MHz) of **3c**

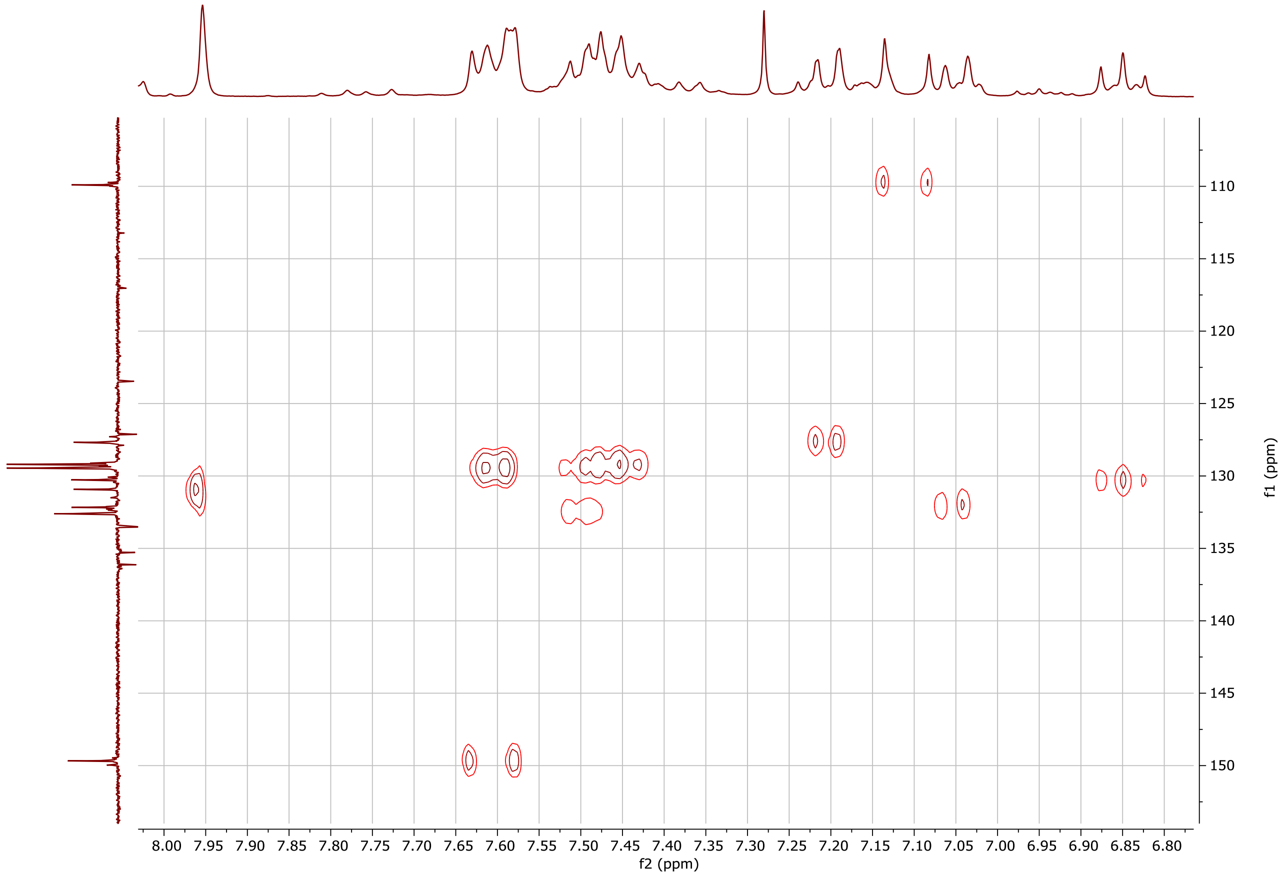

^{1}H-^{13}C HSQC NMR spectrum of **3c**

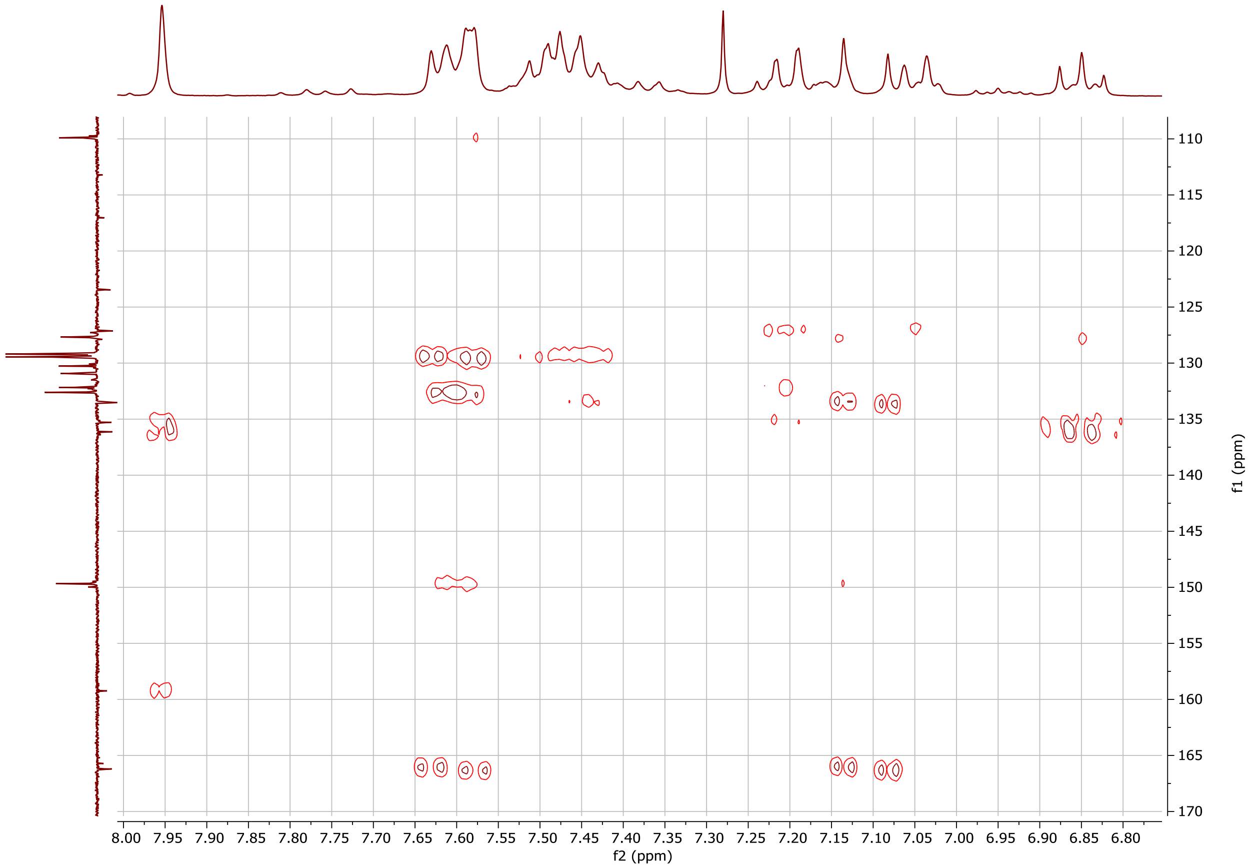

^1H-^{13}C HMBC NMR spectrum of **3c**

Orthopalladated dinuclear derivative 3d

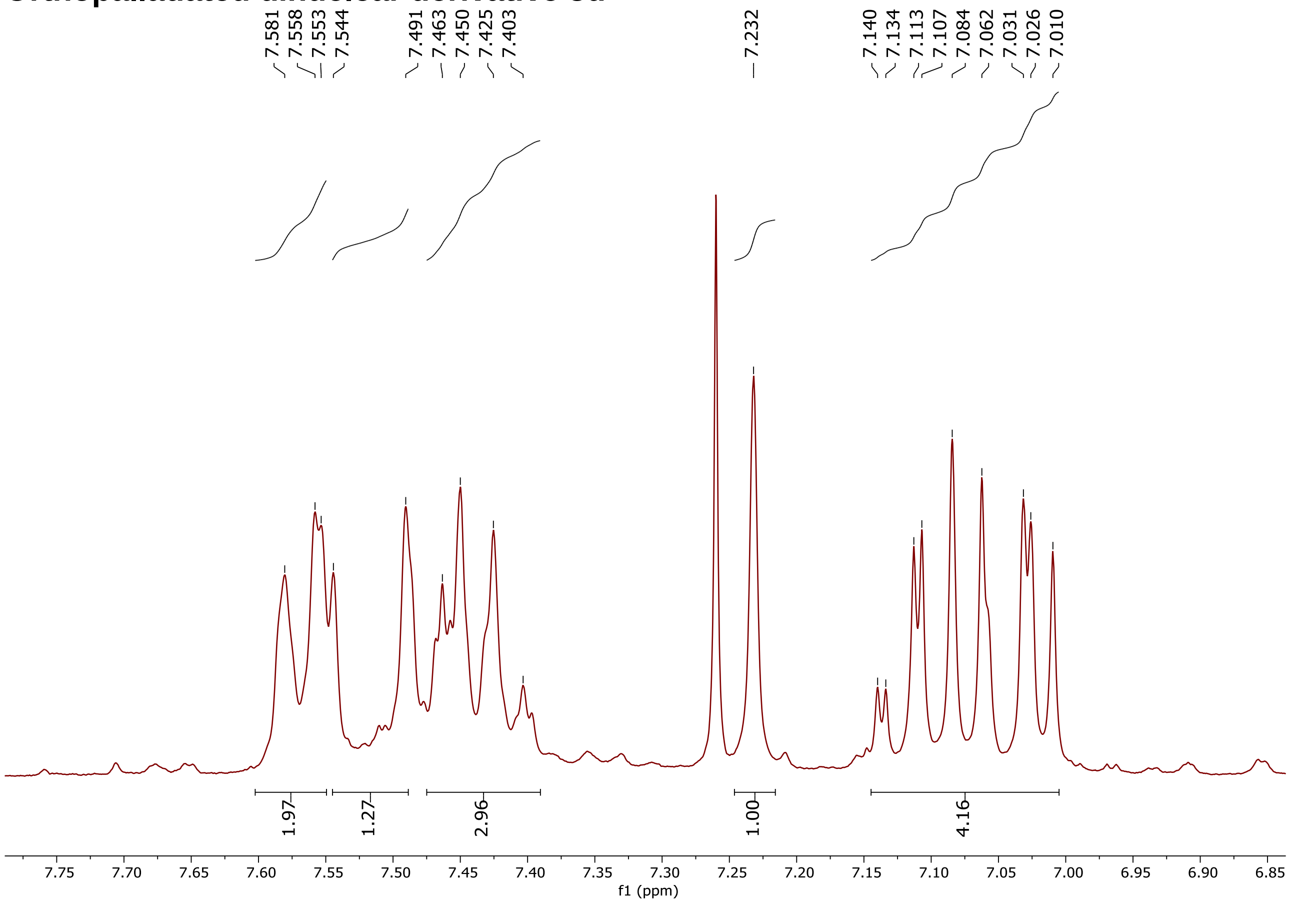

^1H NMR ($CDCl_3$, 300.13 MHz) of **3d**

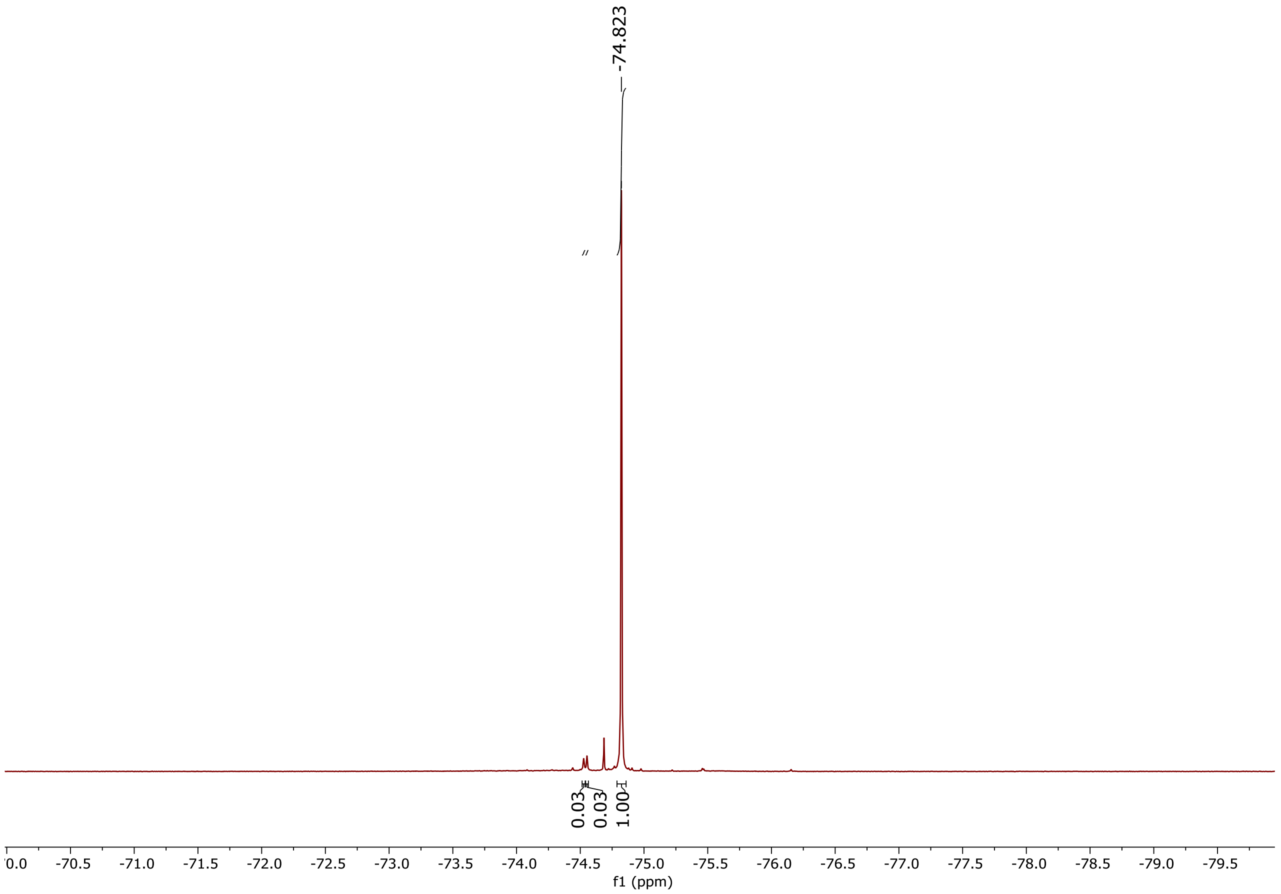

^{19}F-NMR spectrum ($CDCl_3$, 282.40 MHz) of **3d**

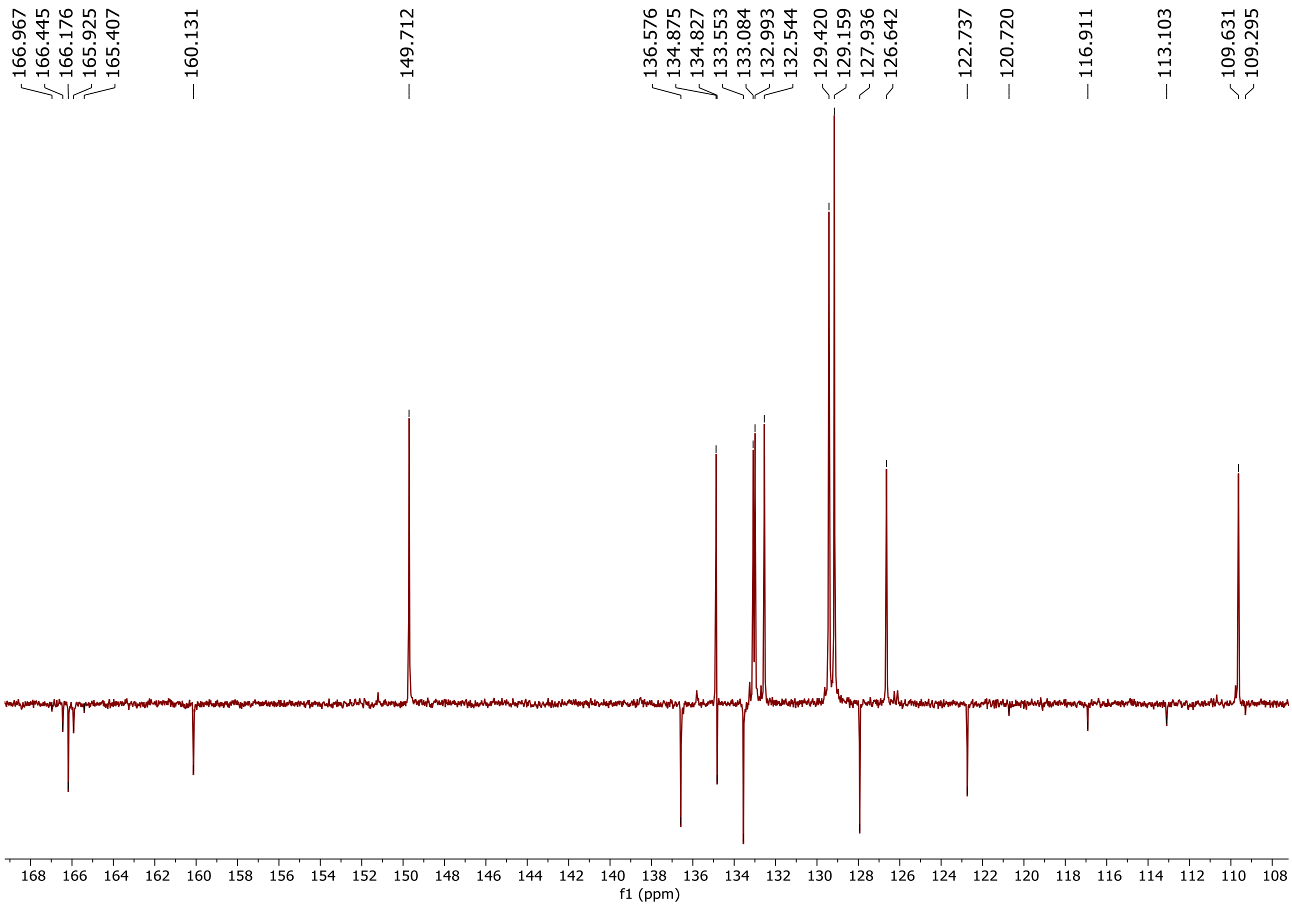

^{13}C{^{1}H}-(APT) NMR spectrum ($CDCl_3$, 75.47 MHz) of **3d**

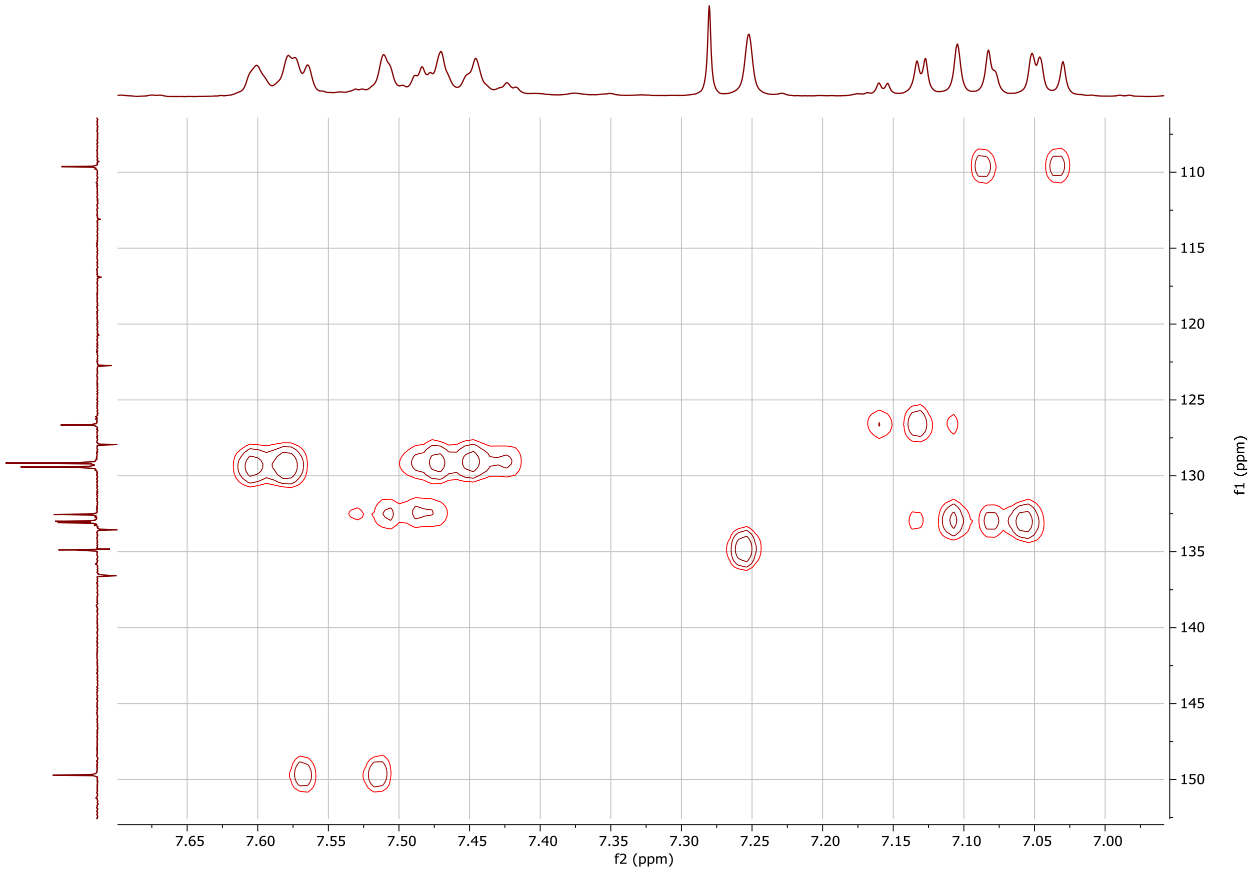

^{1}H-^{13}C HSQC NMR spectrum of **3d**

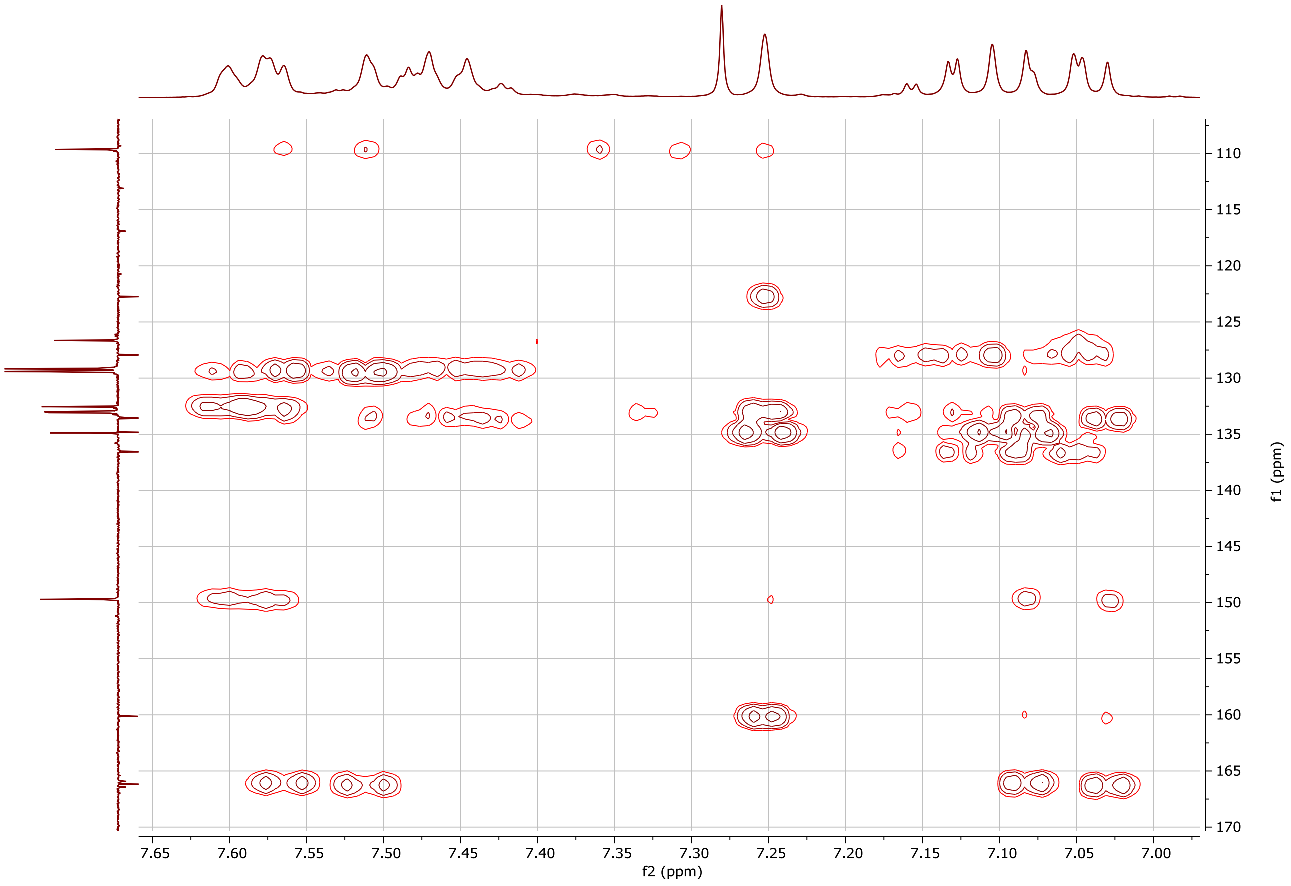

^{1}H-^{13}C HMBC NMR spectrum of **3d**

Orthopalladated dinuclear derivative 3e

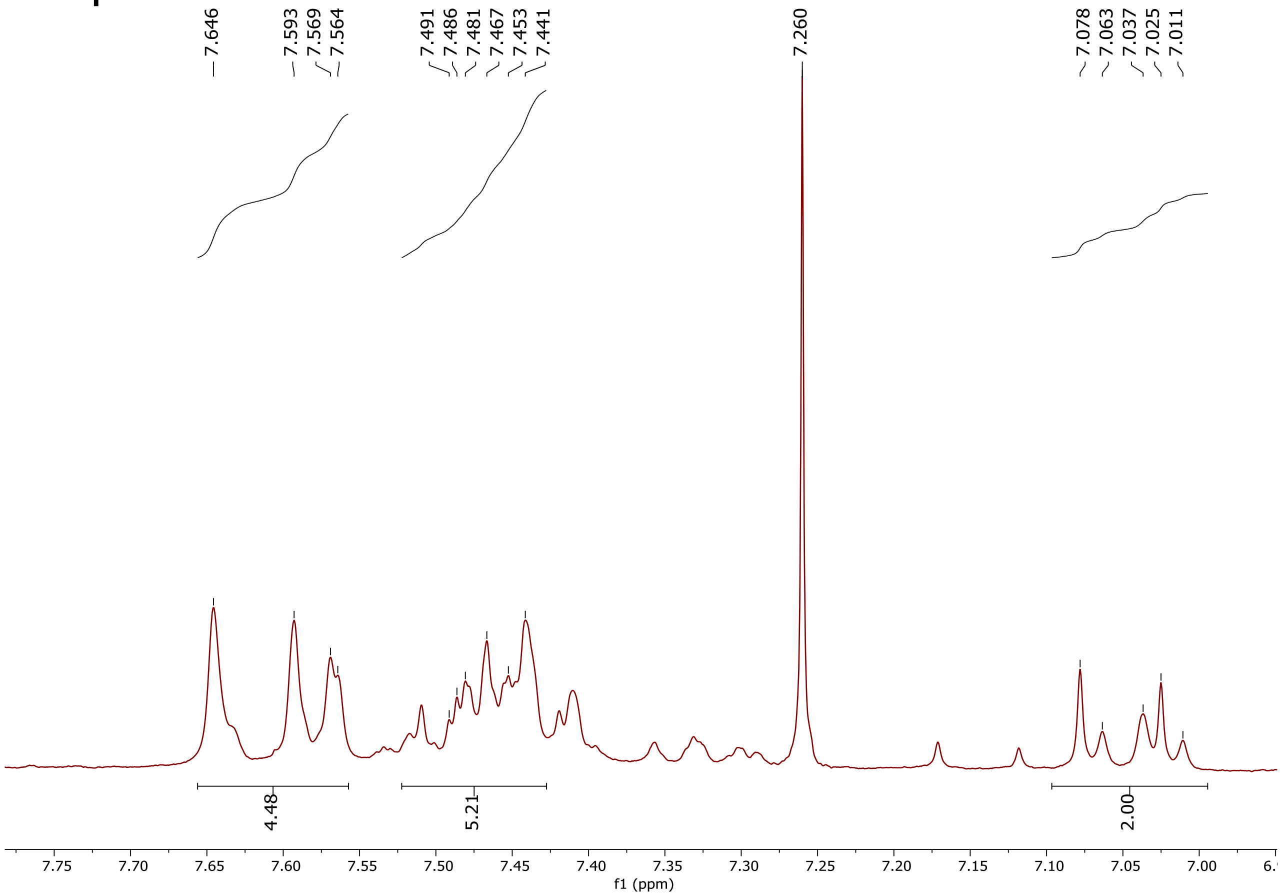

^{1}H NMR ($CDCl_3$, 300.13 MHz) of **3e**

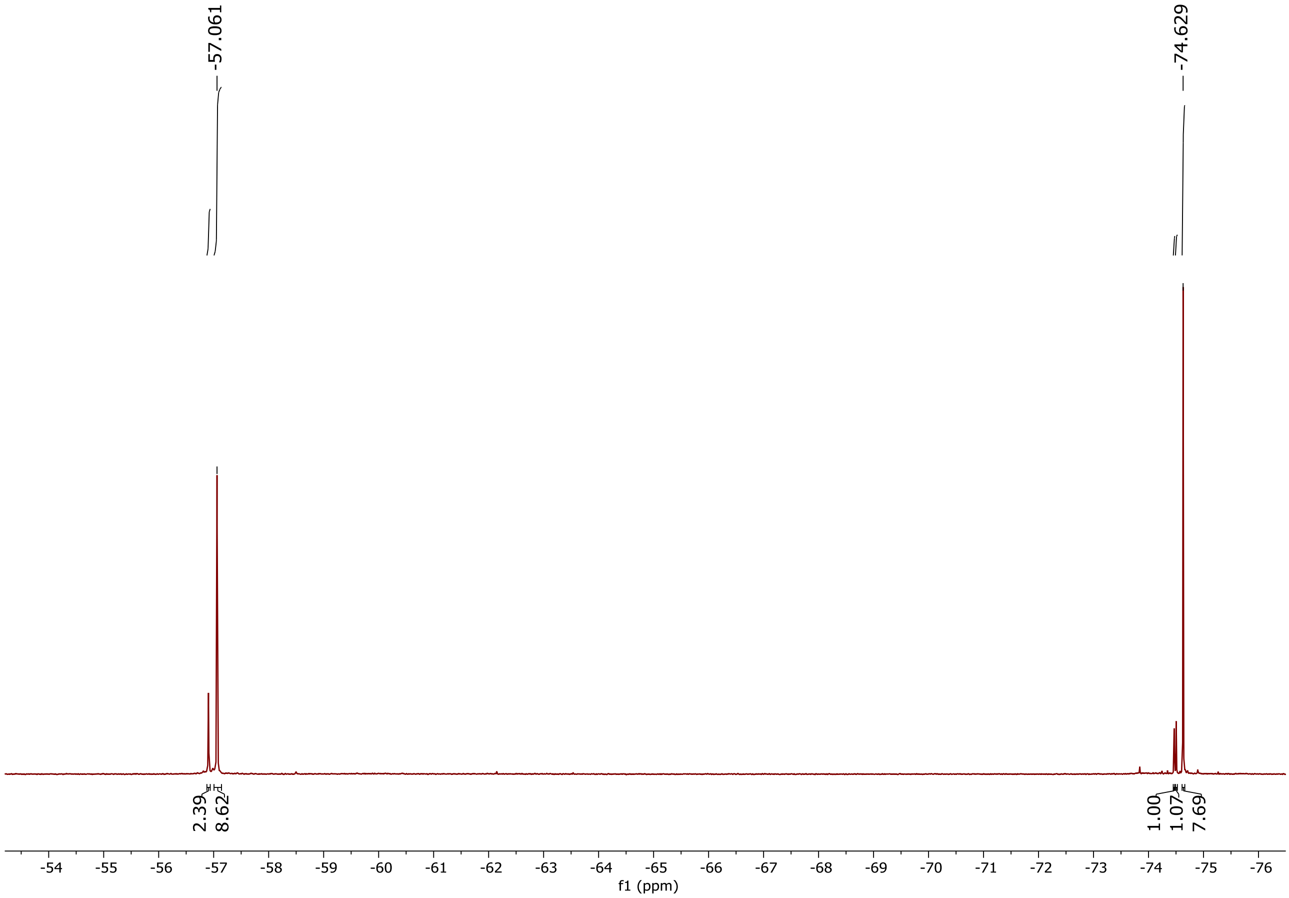

^{19}F-NMR spectrum ($CDCl_3$, 282.40 MHz) of **3e**

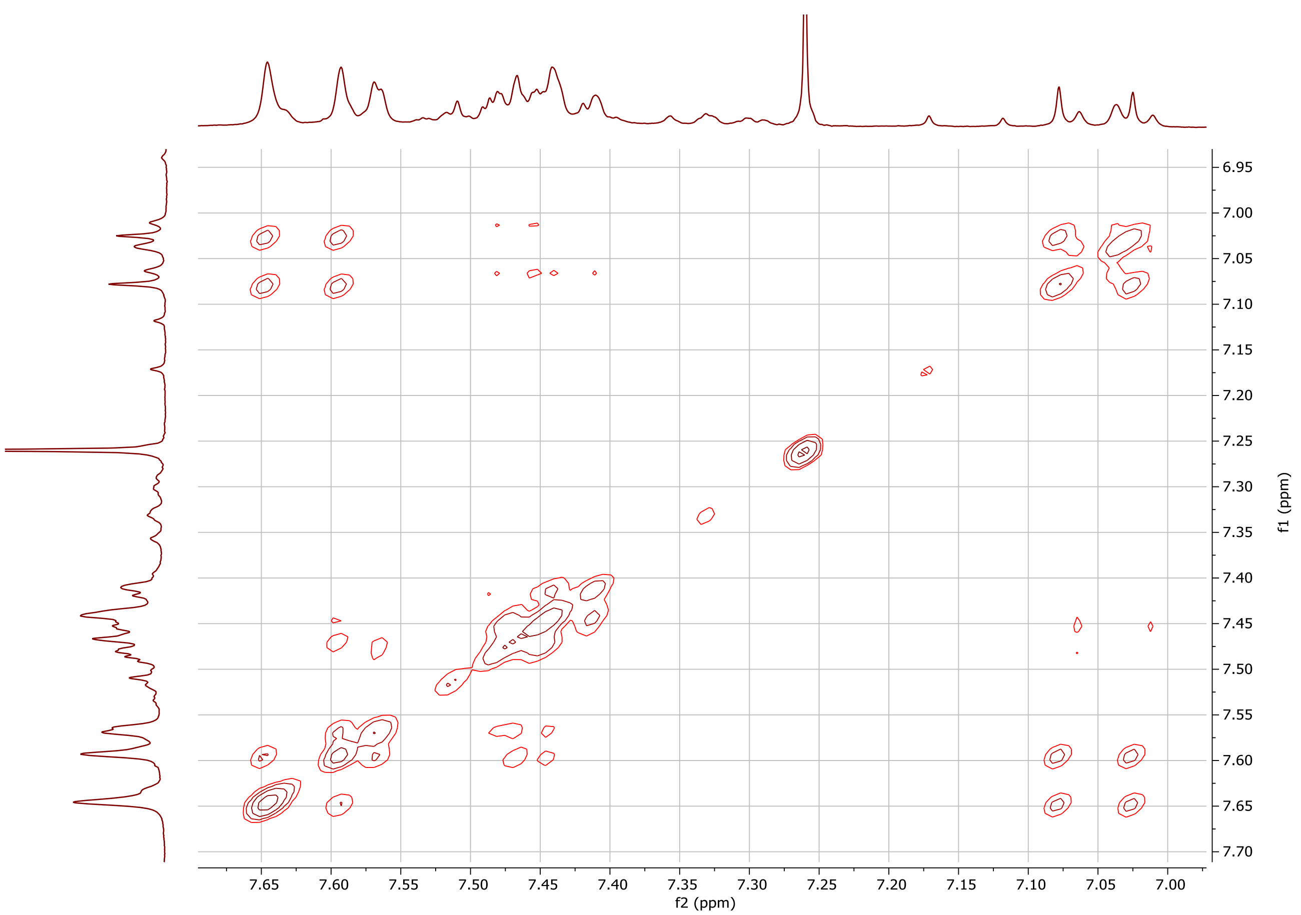

^{1}H-^{1}H COSY NMR spectrum of **3e**

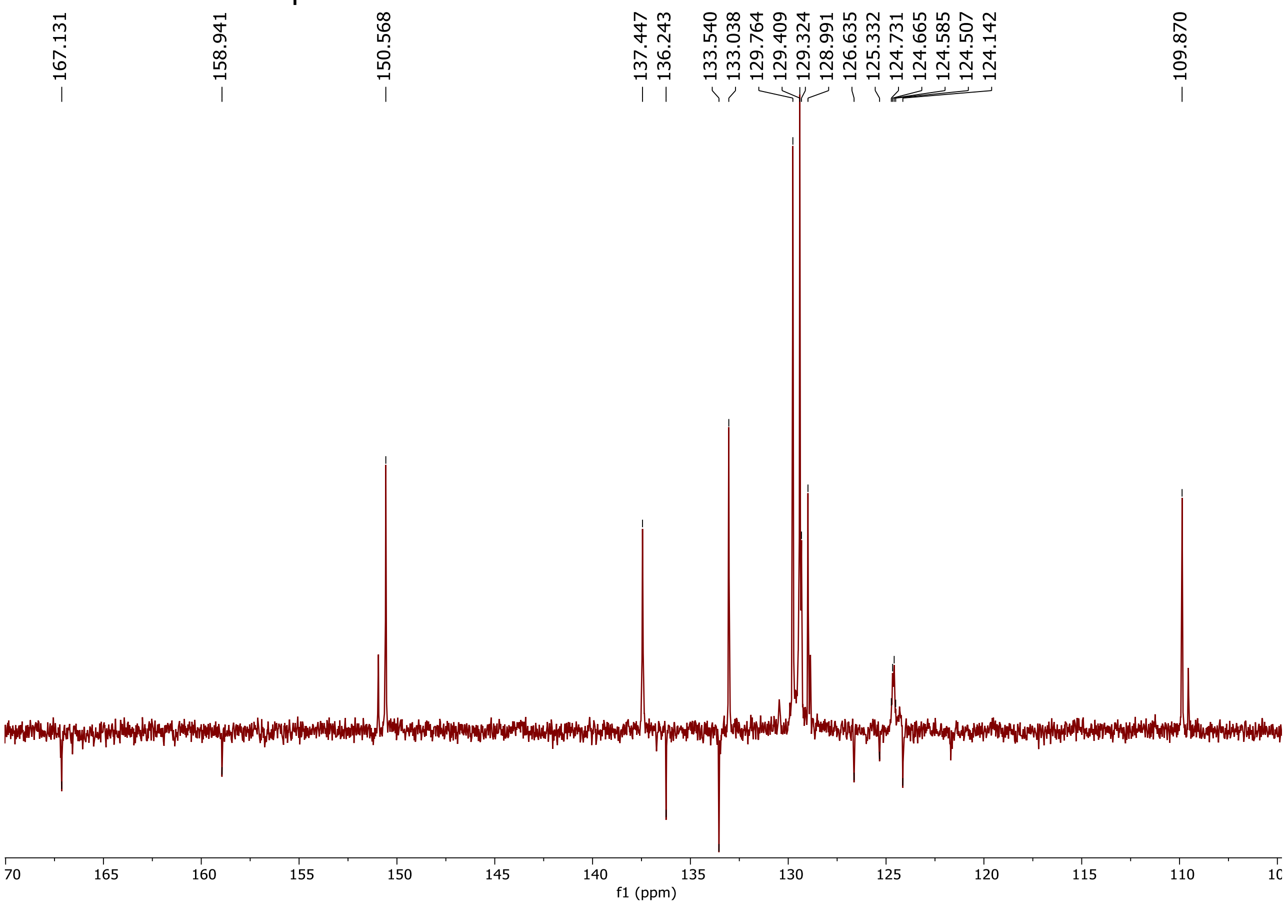

$^{13}C\{^{1}H\}$-(APT) NMR spectrum ($CDCl_3$, 75.47 MHz) of **3e**

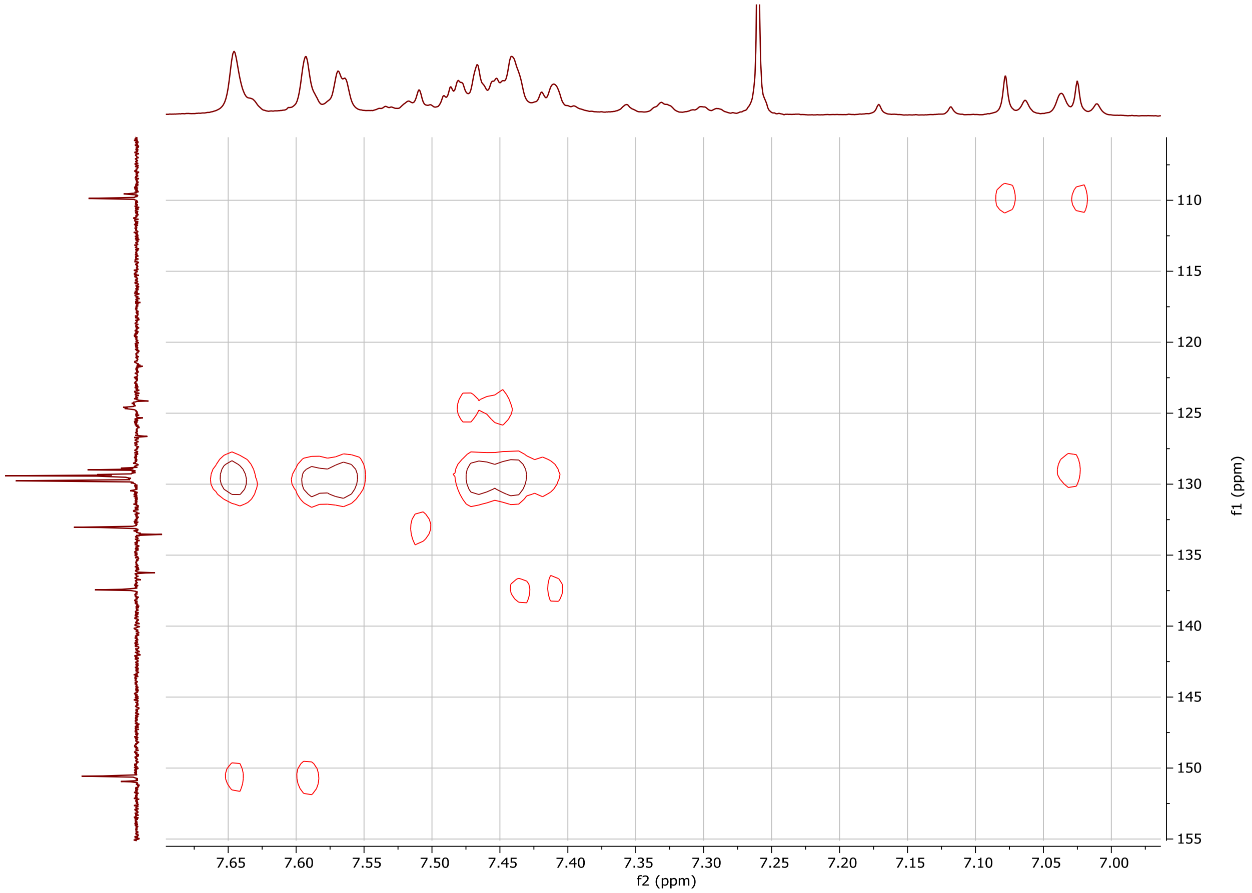

^{1}H-^{13}C HSQC NMR spectrum of **3e**

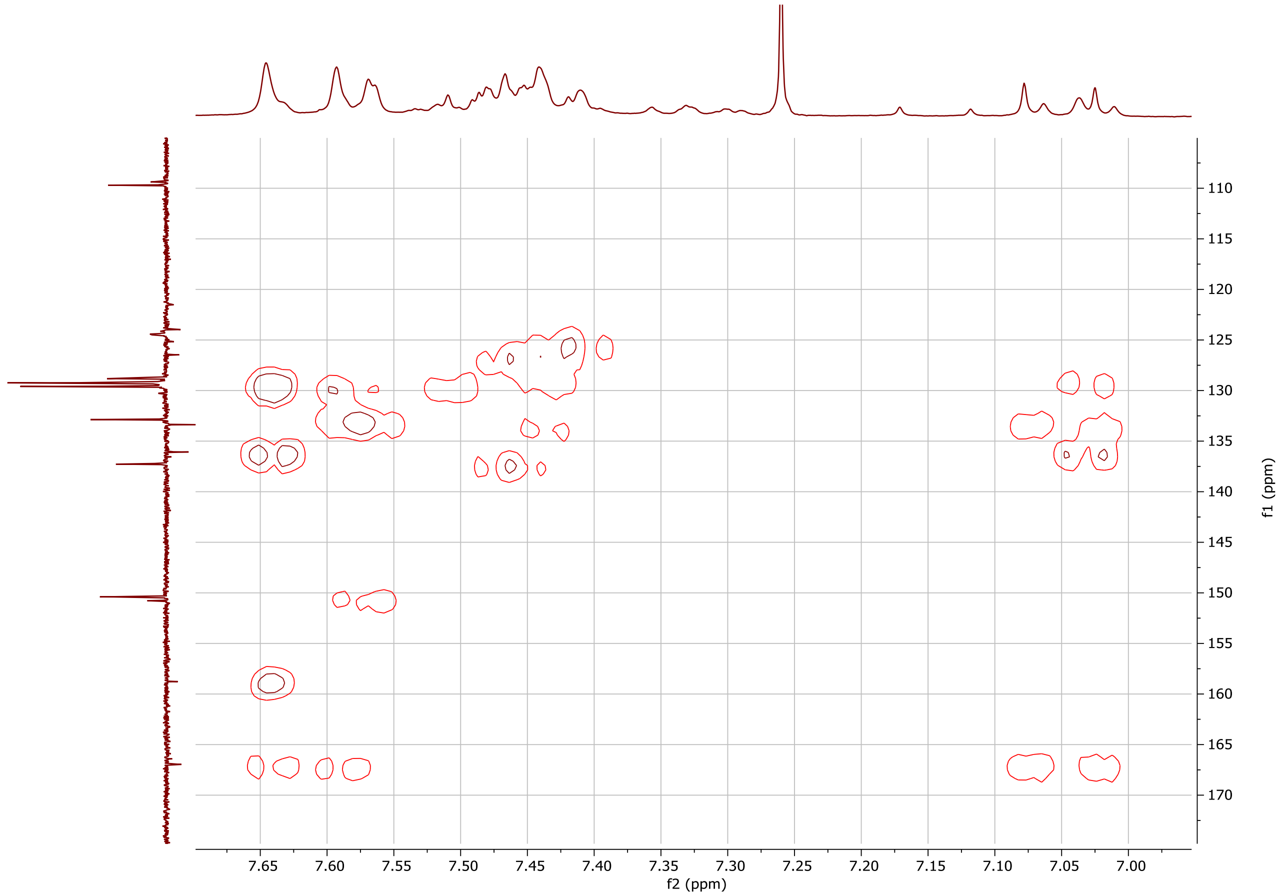

^{1}H-^{13}C HMBC NMR spectrum of **3e**

Orthopalladated dinuclear derivative 3f

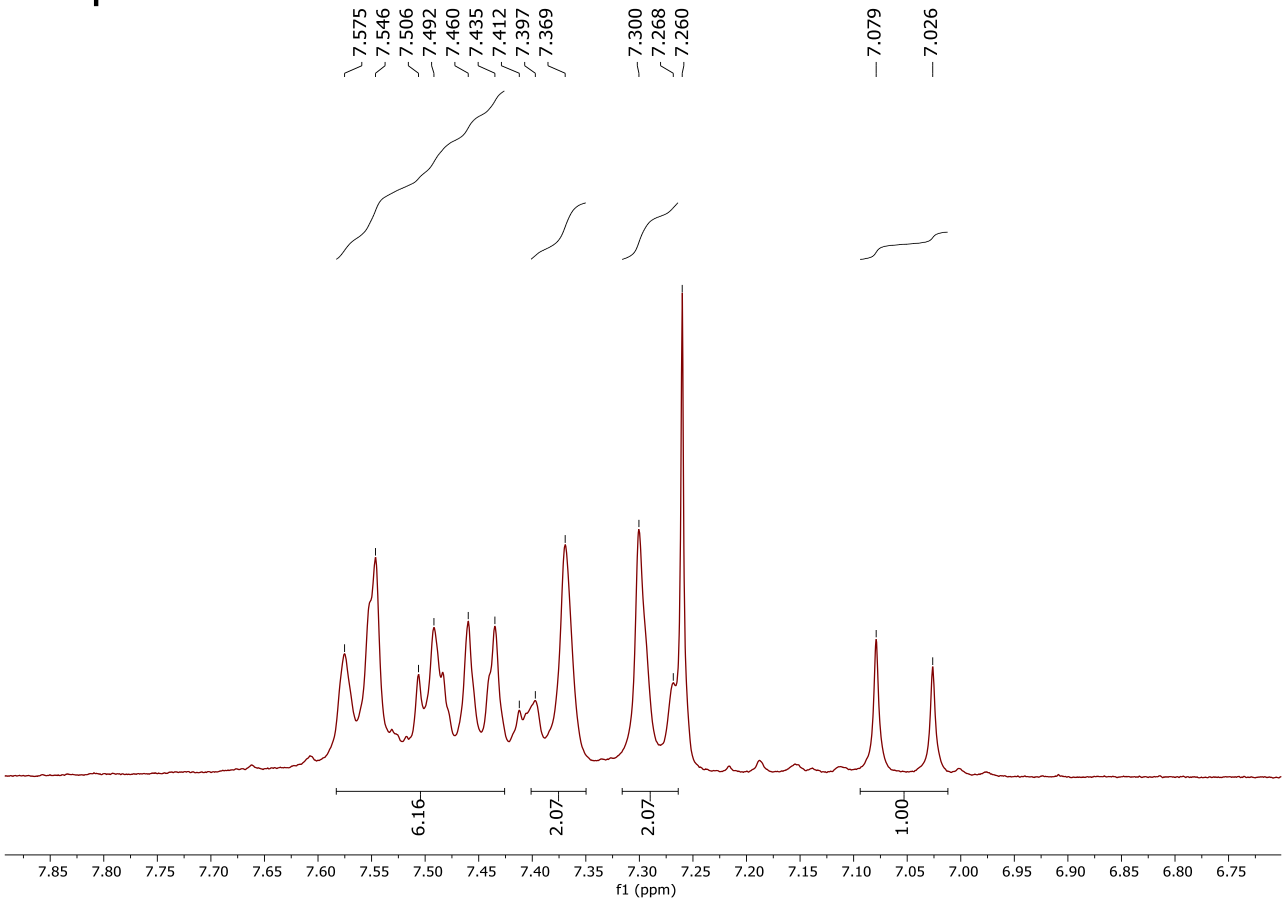

^{1}H NMR (CDCl3, 300.13 MHz) of **3f**

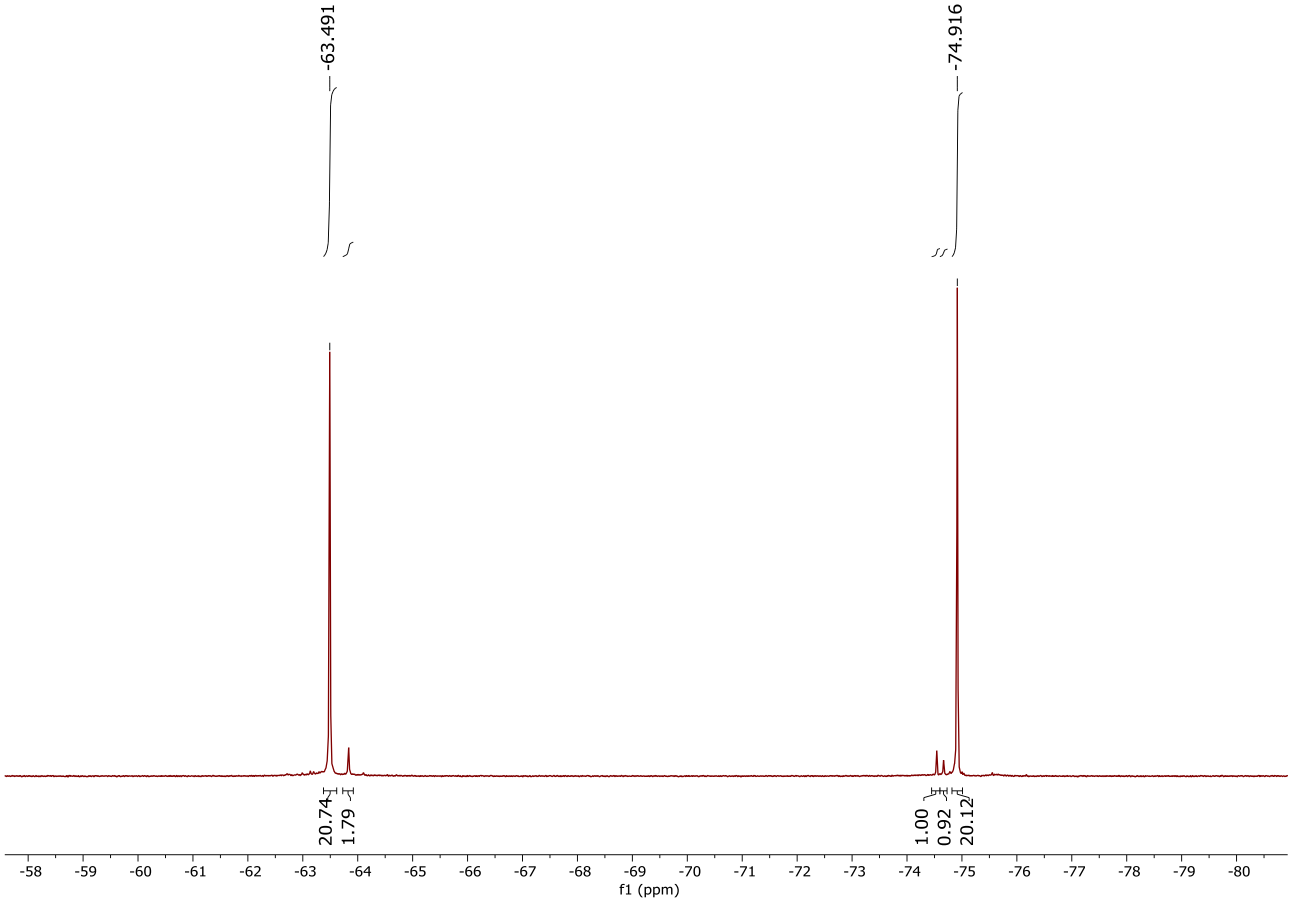

^{19}F-NMR spectrum (CDCl3, 282.40 MHz) of **3f**

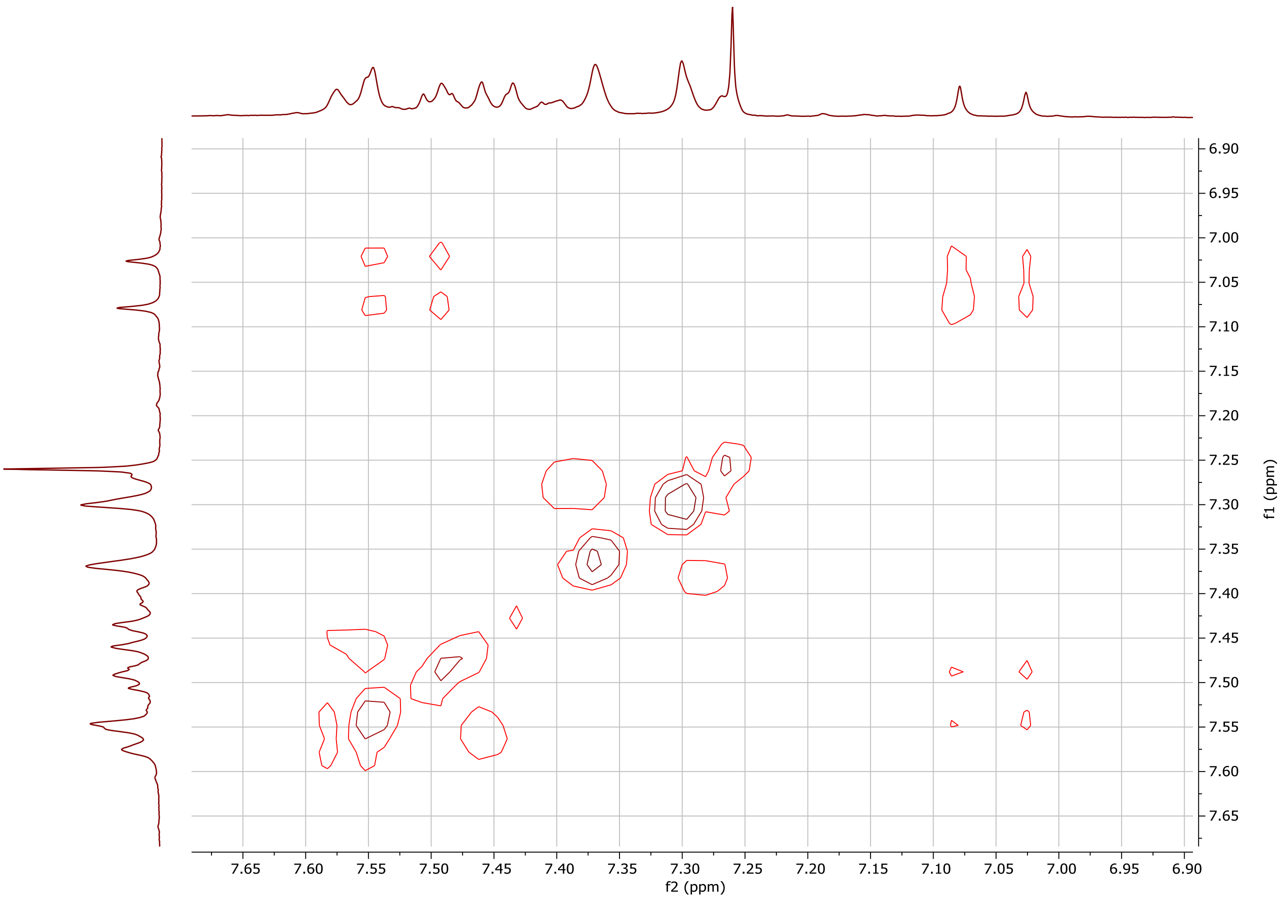

^{1}H-^{1}H COSY NMR spectrum of **3f**

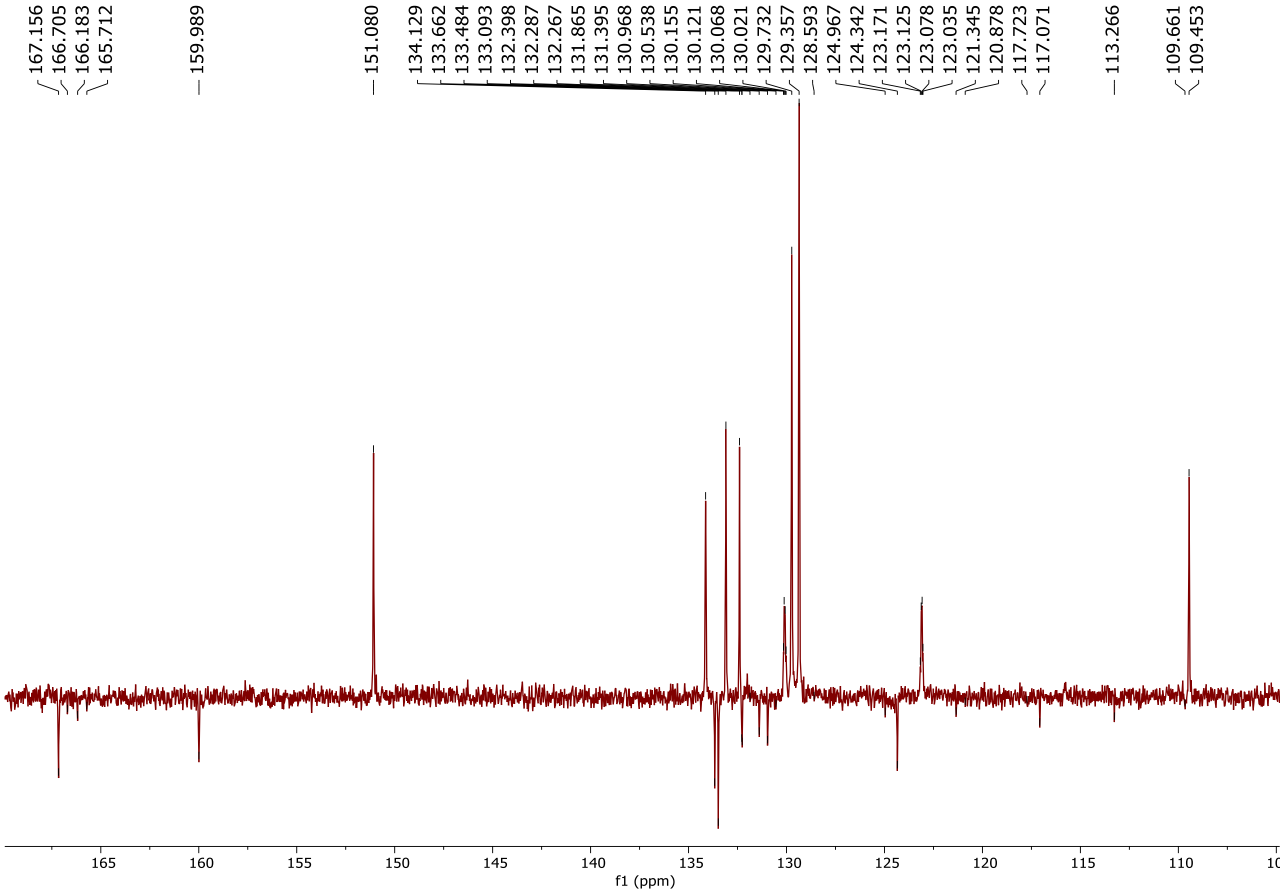

$^{13}C\{^{1}H\}$-(APT) NMR spectrum ($CDCl_3$, 75.47 MHz) of **3f**

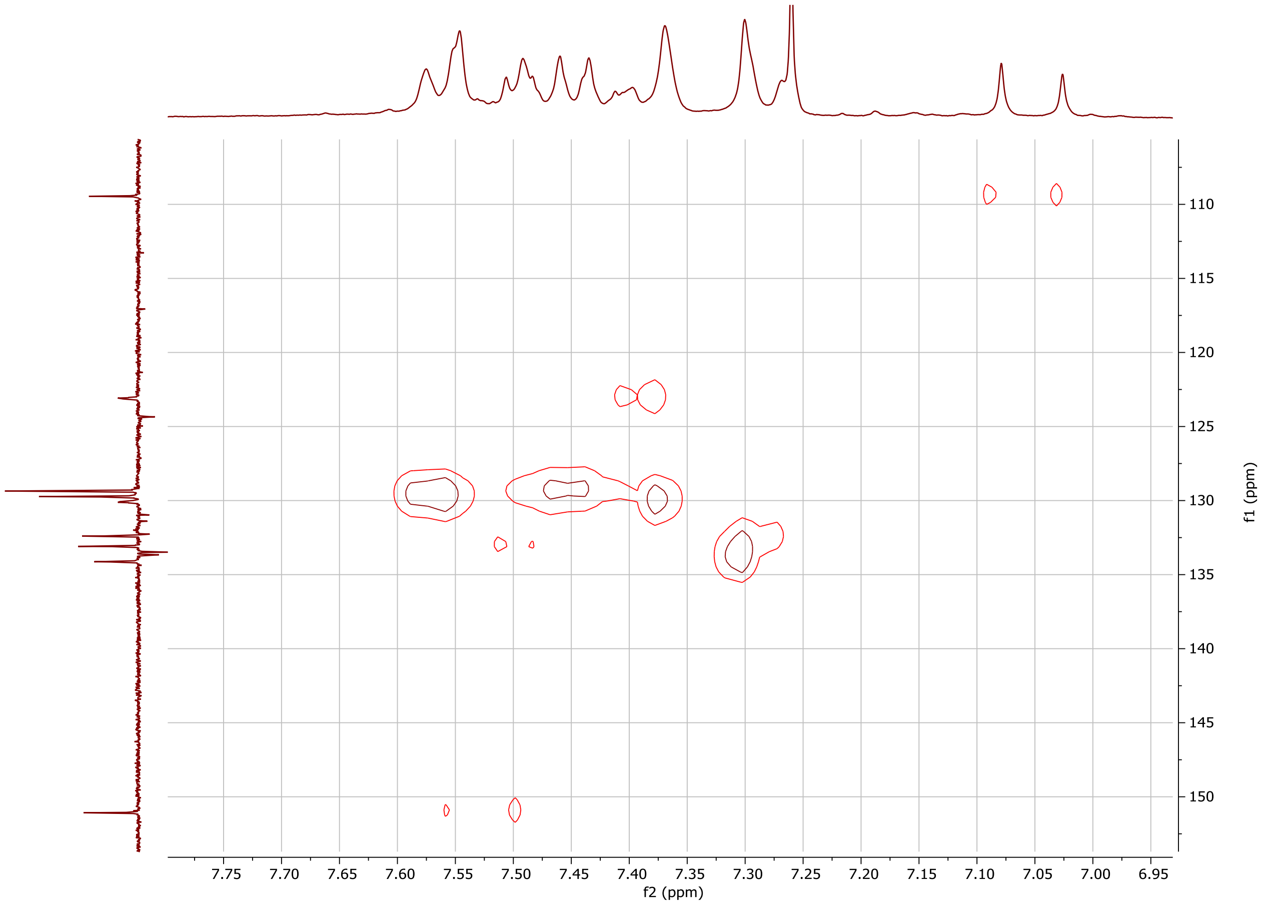

^{1}H-^{13}C HSQC NMR spectrum of **3f**

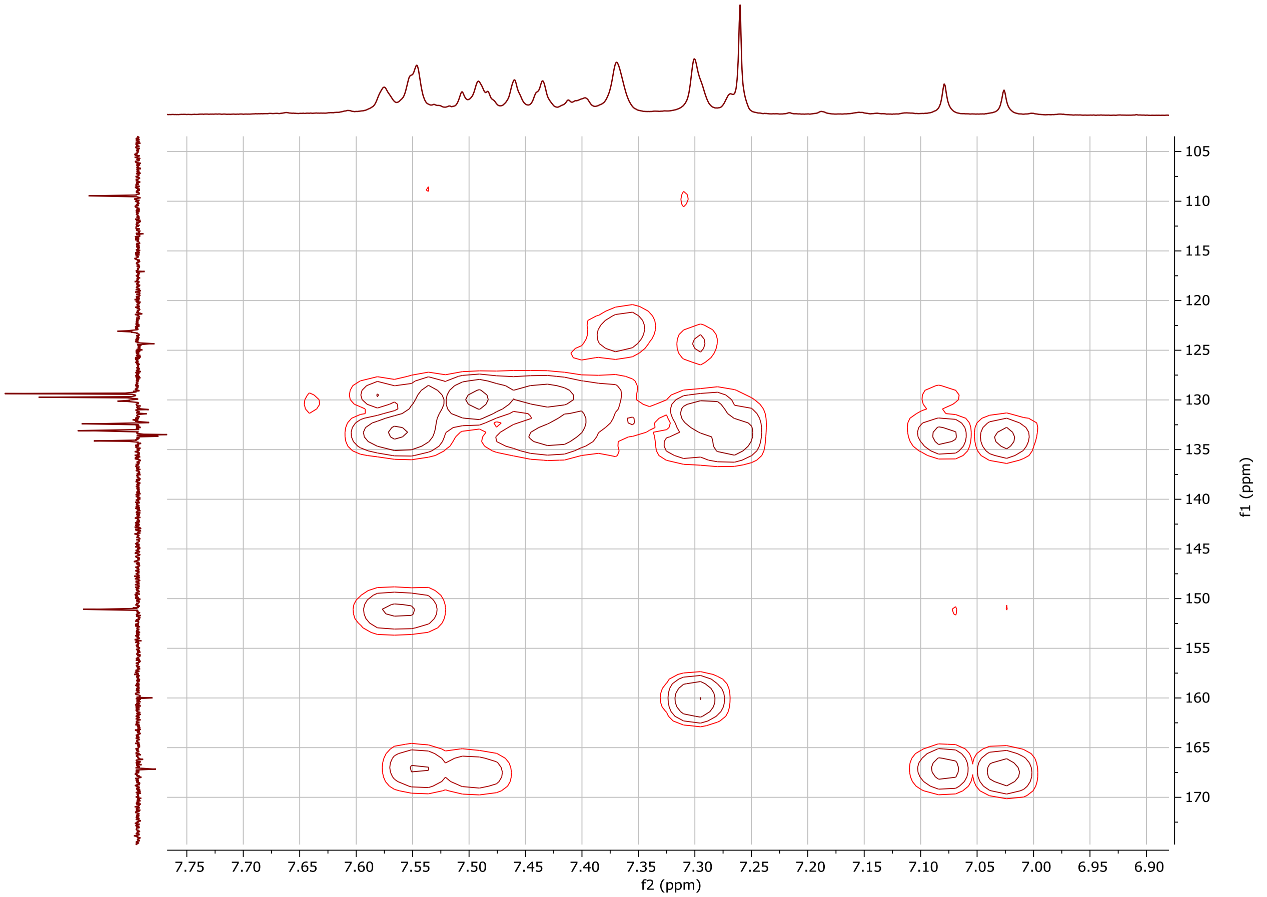

^{1}H-^{13}C HMBC NMR spectrum of **3f**

Orthopalladated dinuclear derivative 3h

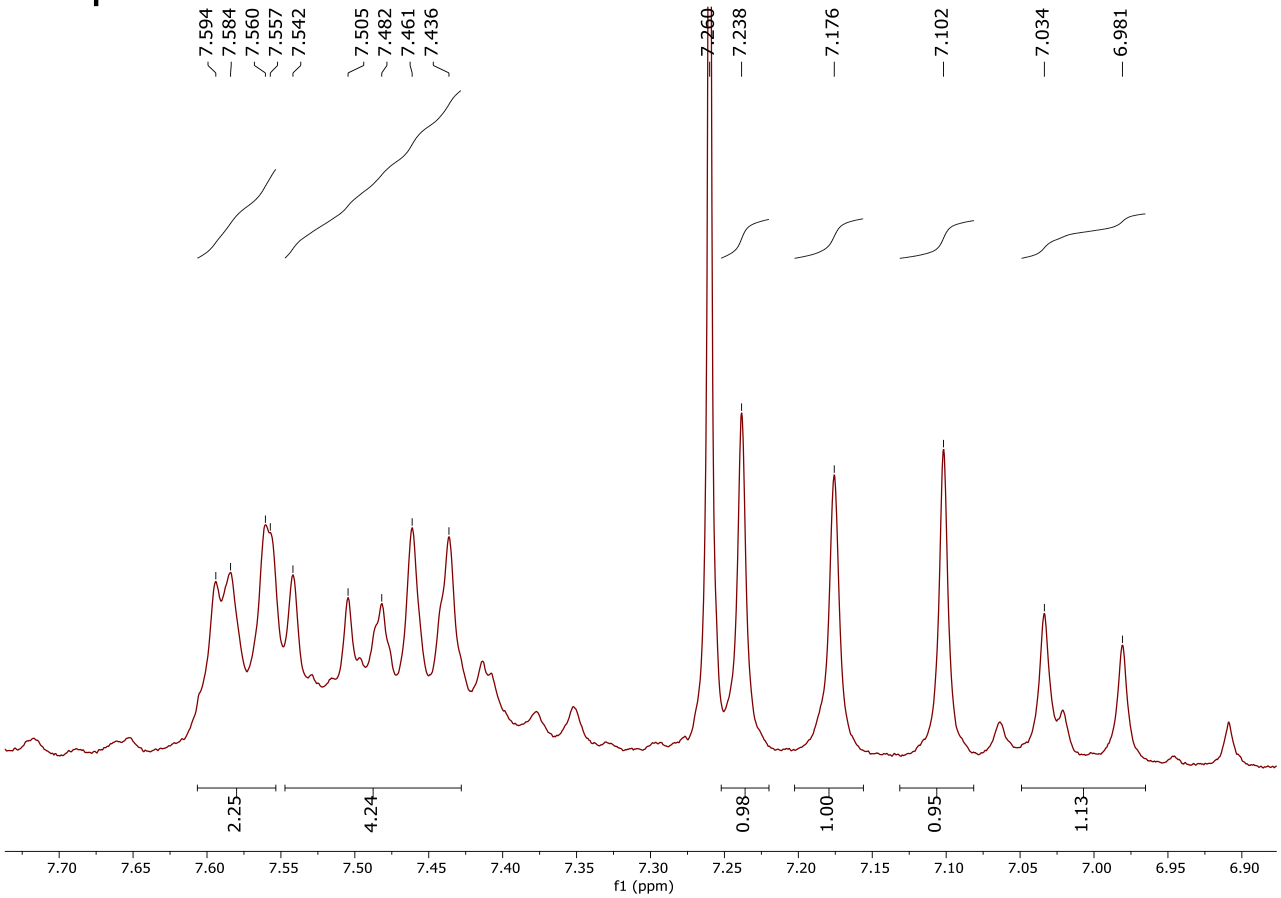

^{1}H NMR ($CDCl_3$, 300.13 MHz) of **3h**

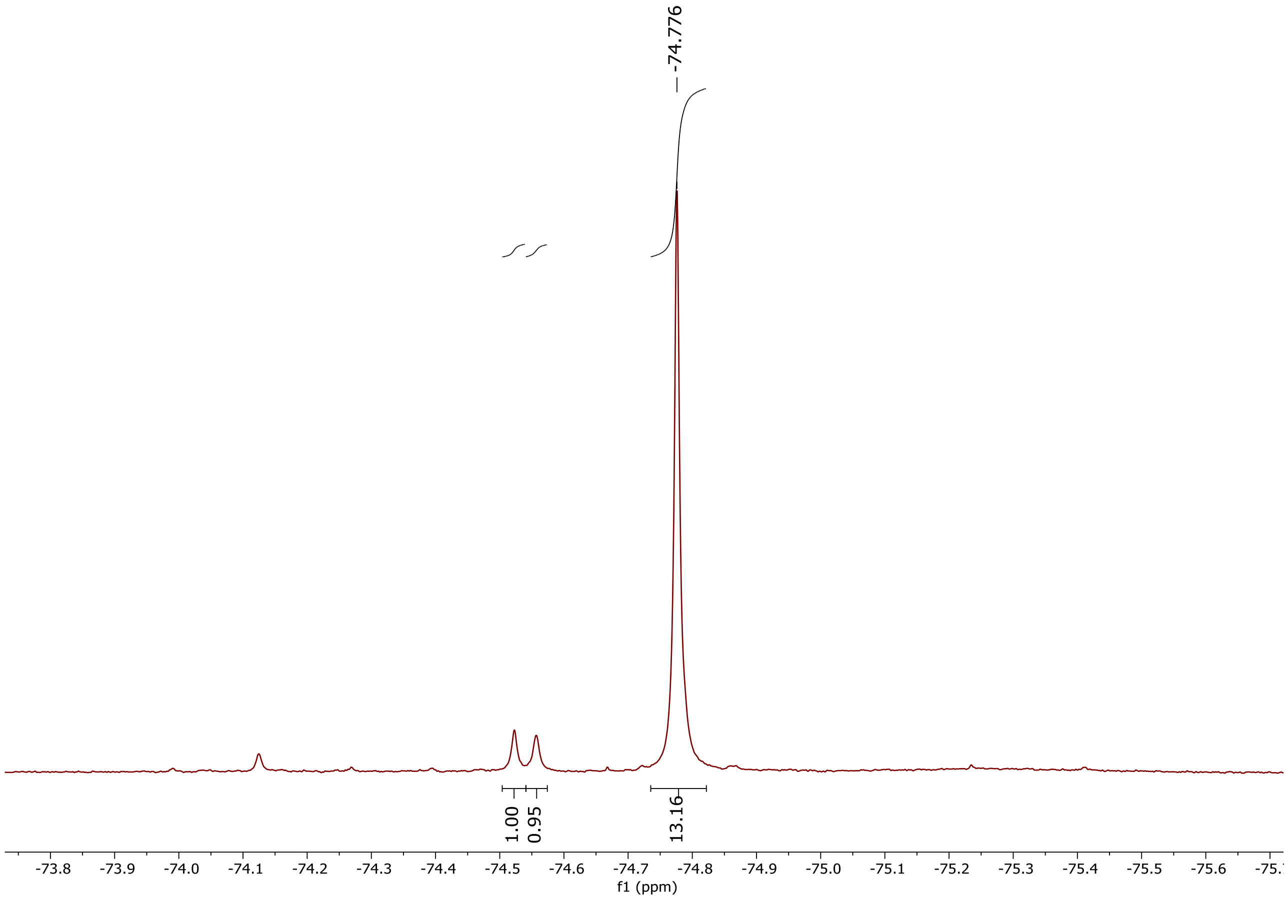

^{19}F-NMR spectrum ($CDCl_3$, 282.40 MHz) of **3h**

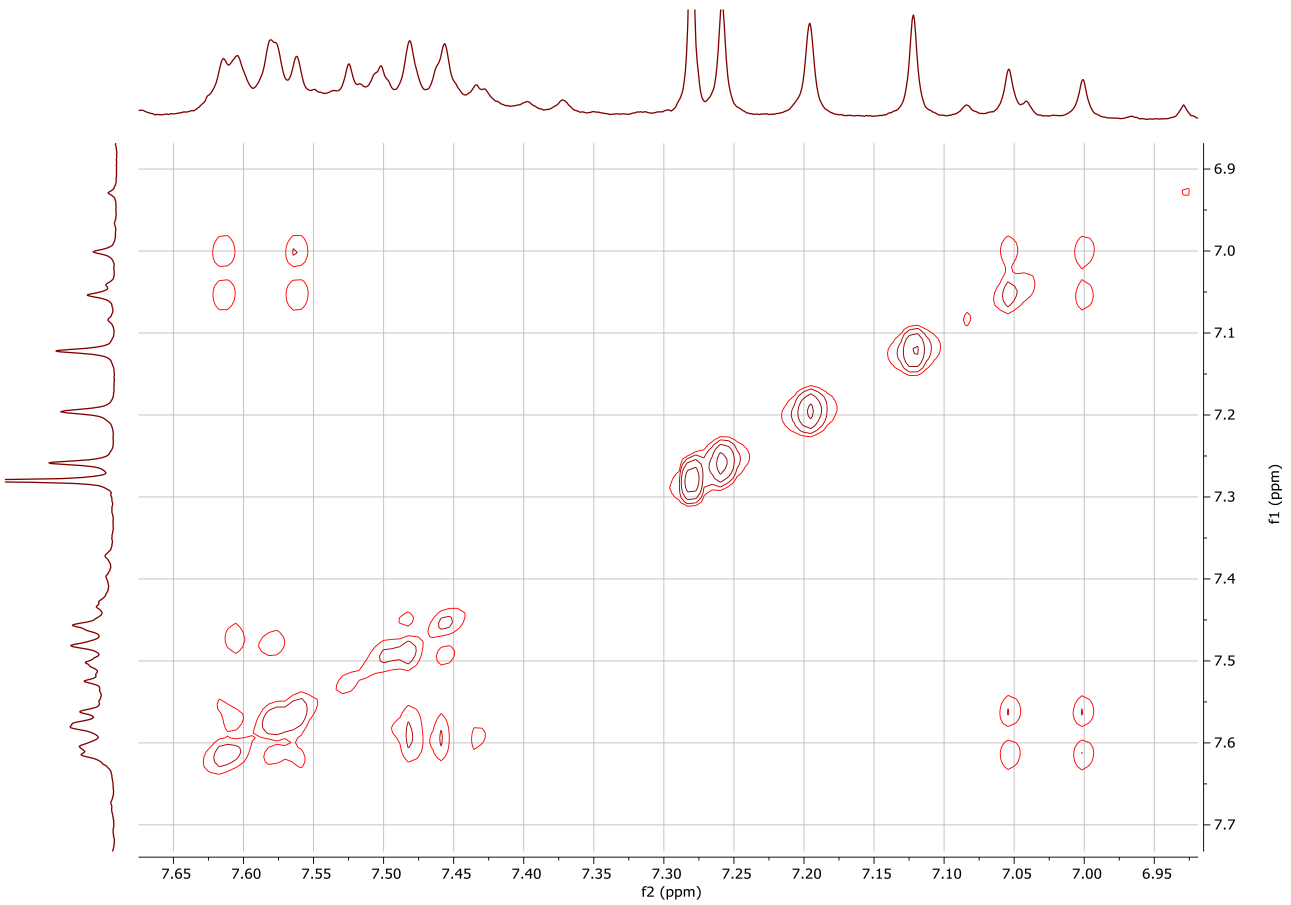

^{1}H-^{1}H COSY NMR spectrum of **3h**

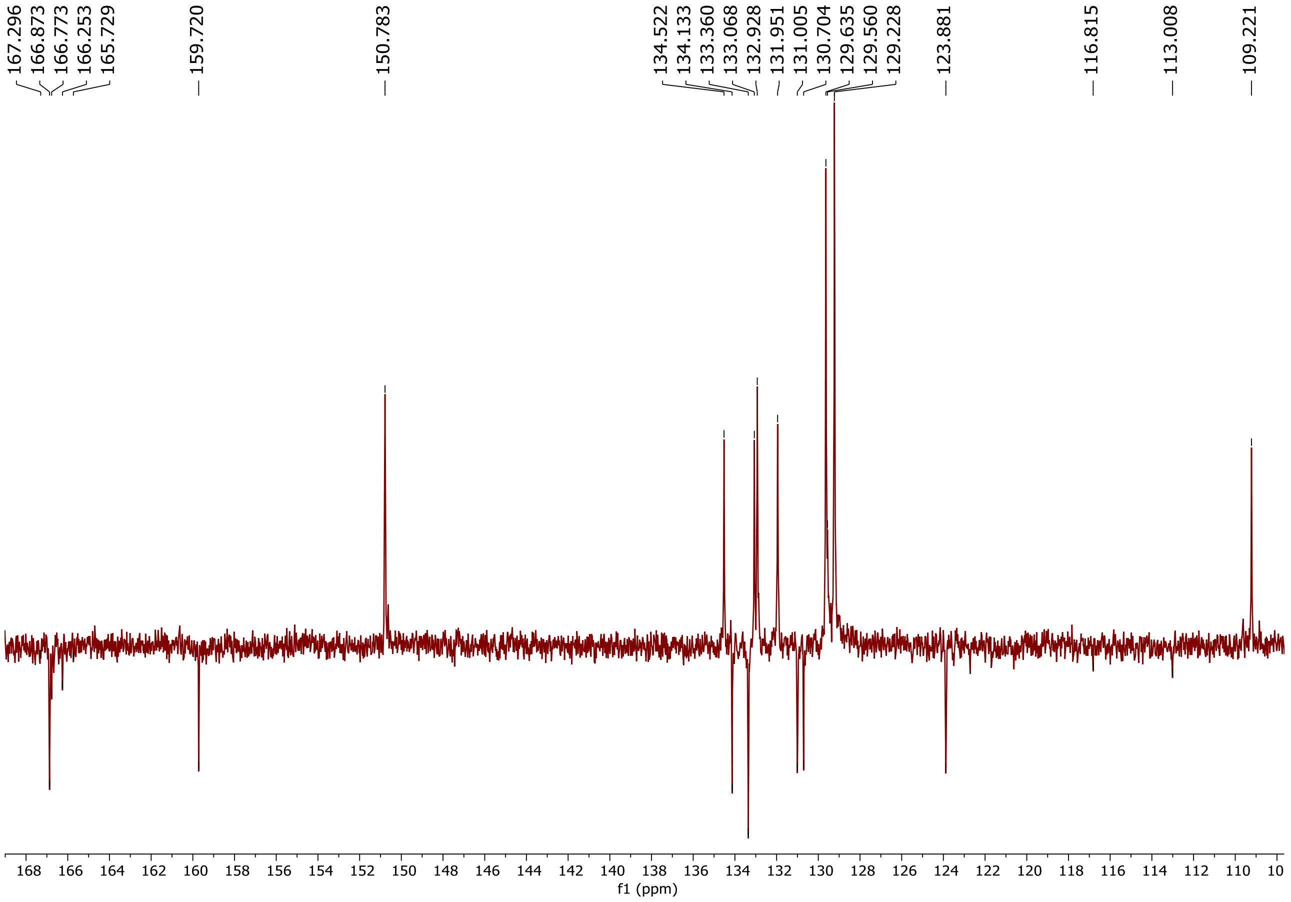

^{13}C{^{1}H}-(APT) NMR spectrum ($CDCl_3$, 75.47 MHz) of **3h**

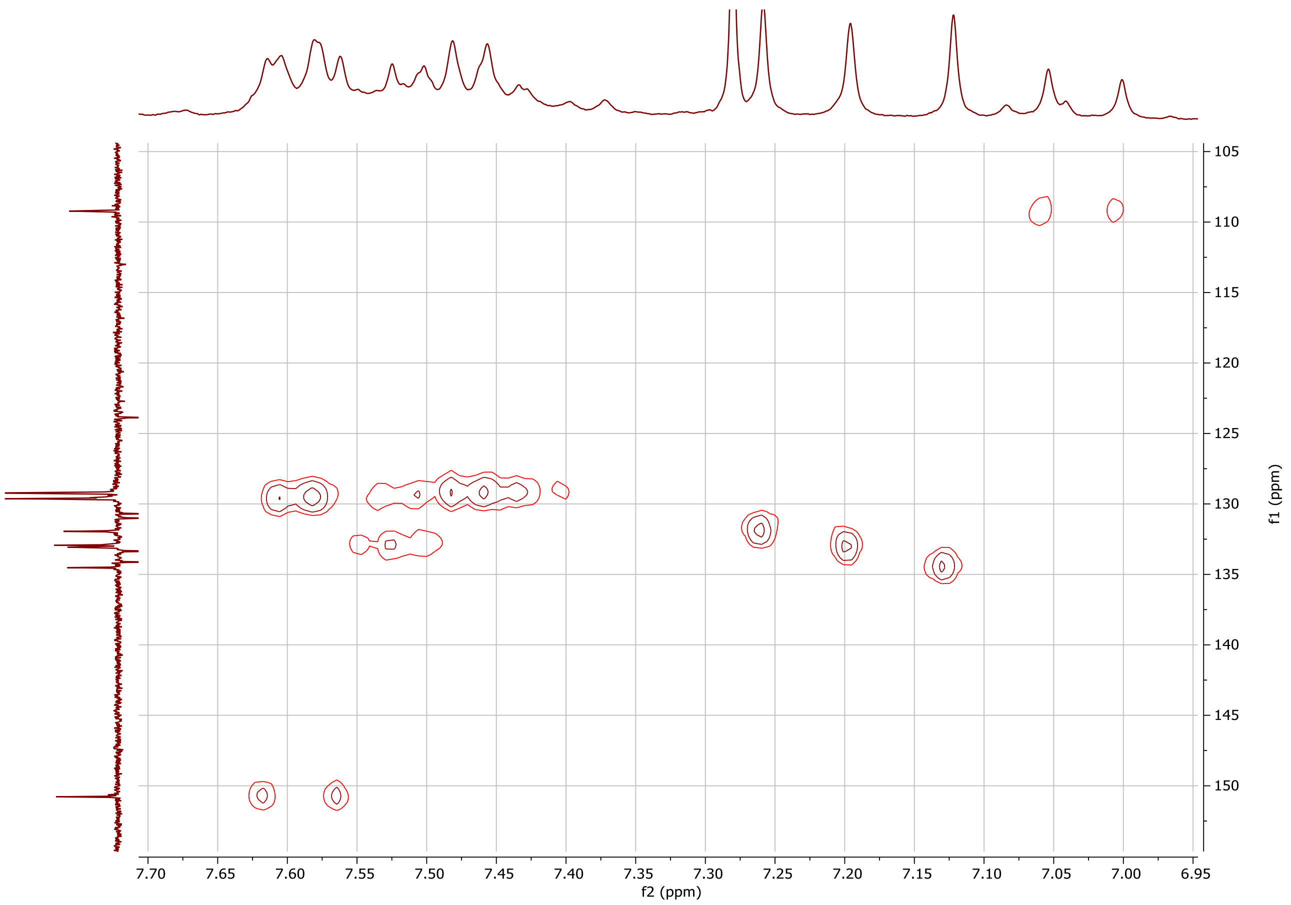

^1H-^{13}C HSQC NMR spectrum of **3h**

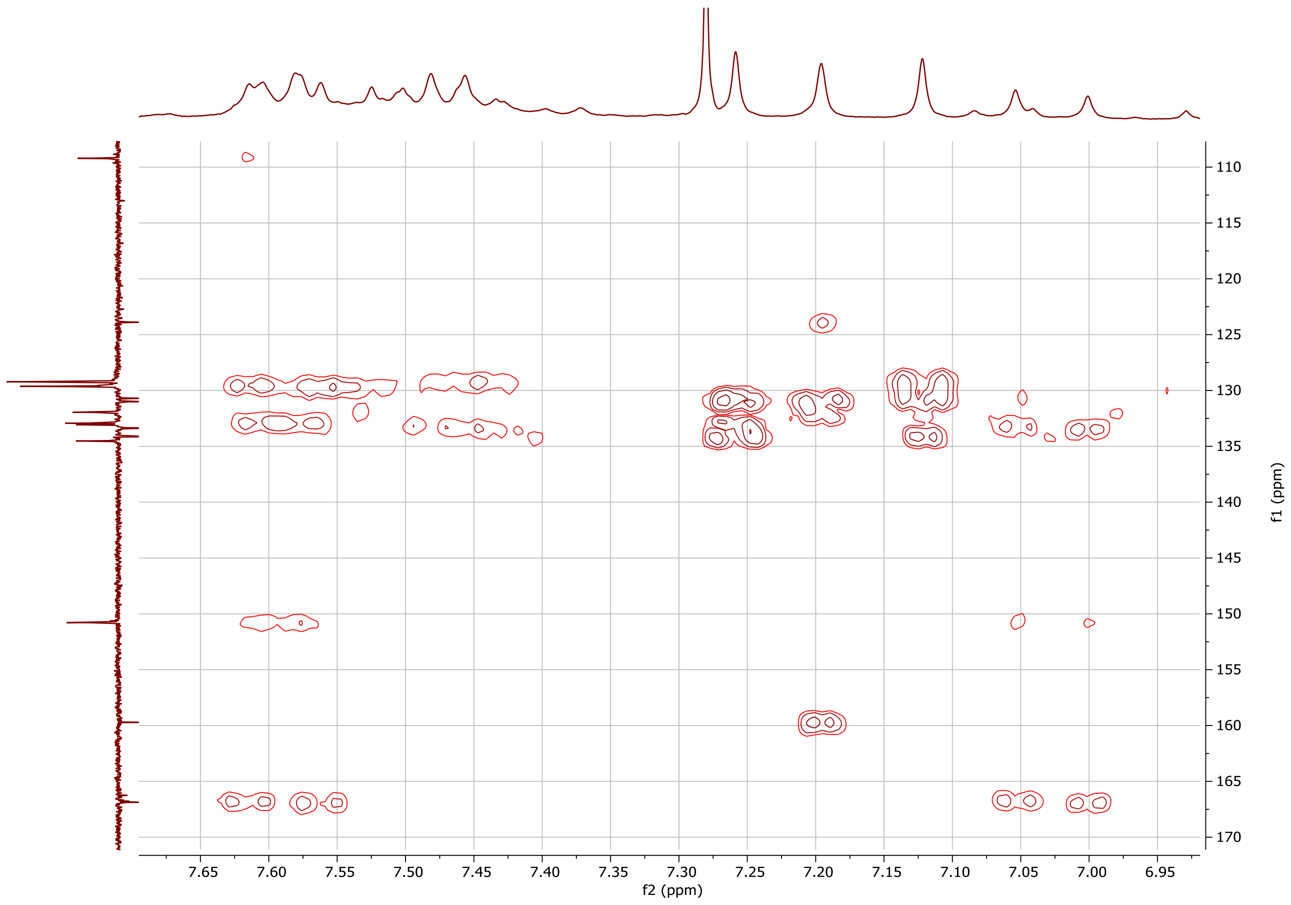

^1H-^{13}C HMBC NMR spectrum of **3h**

Orthopalladated dinuclear derivative 3i

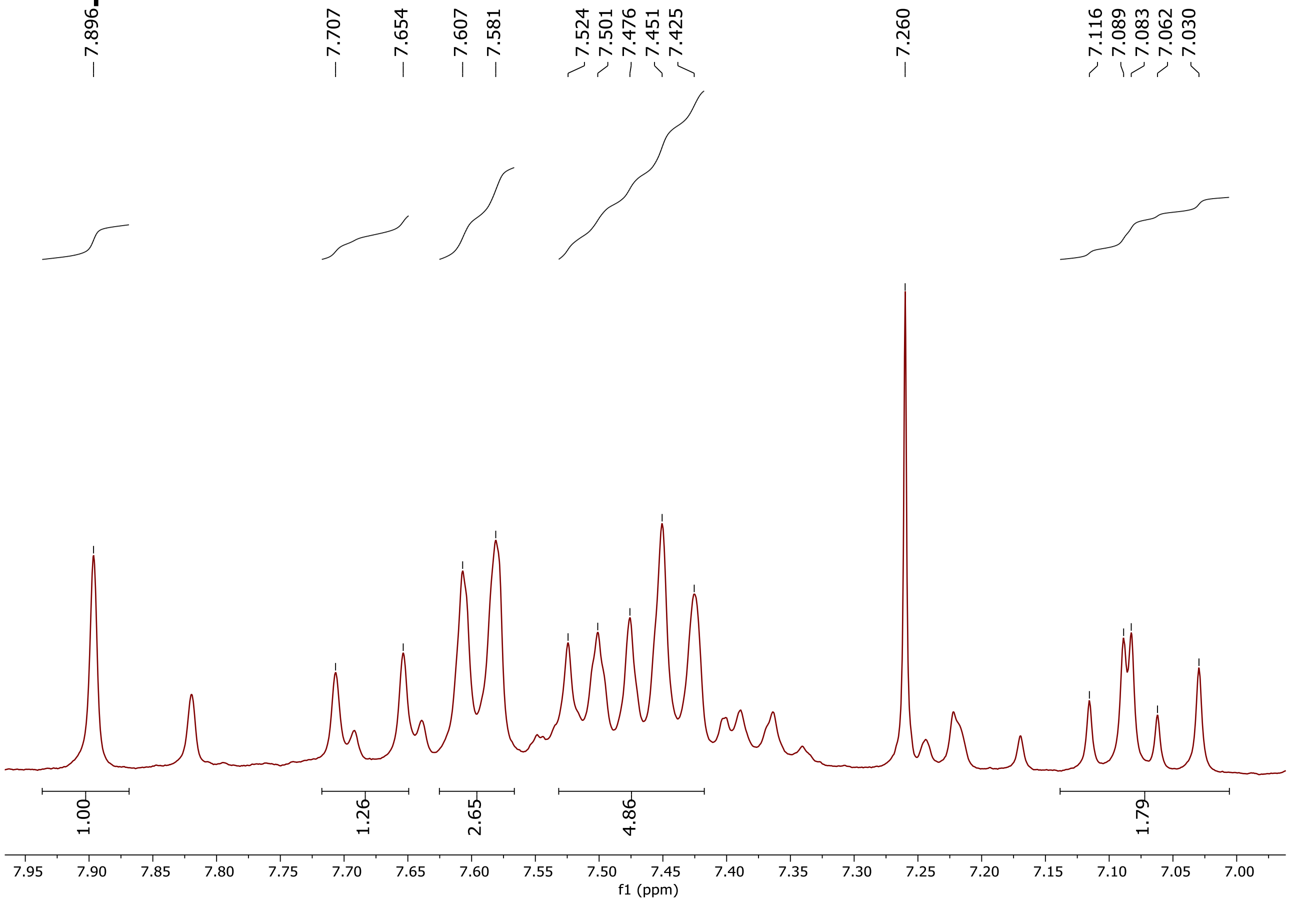

^{1}H NMR ($CDCl_3$, 300.13 MHz) of **3i**

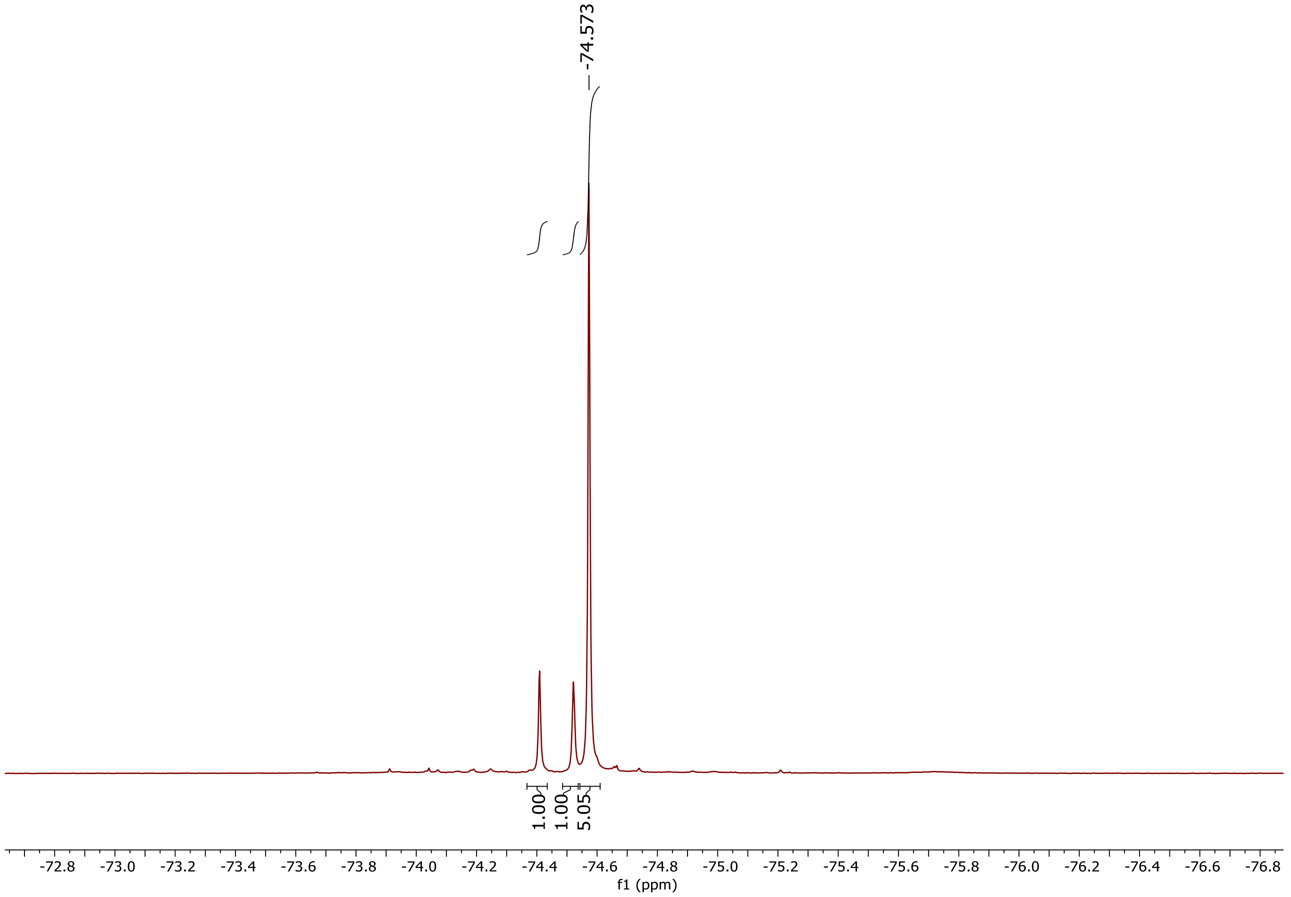

^{19}F-NMR spectrum ($CDCl_3$, 282.40 MHz) of **3i**

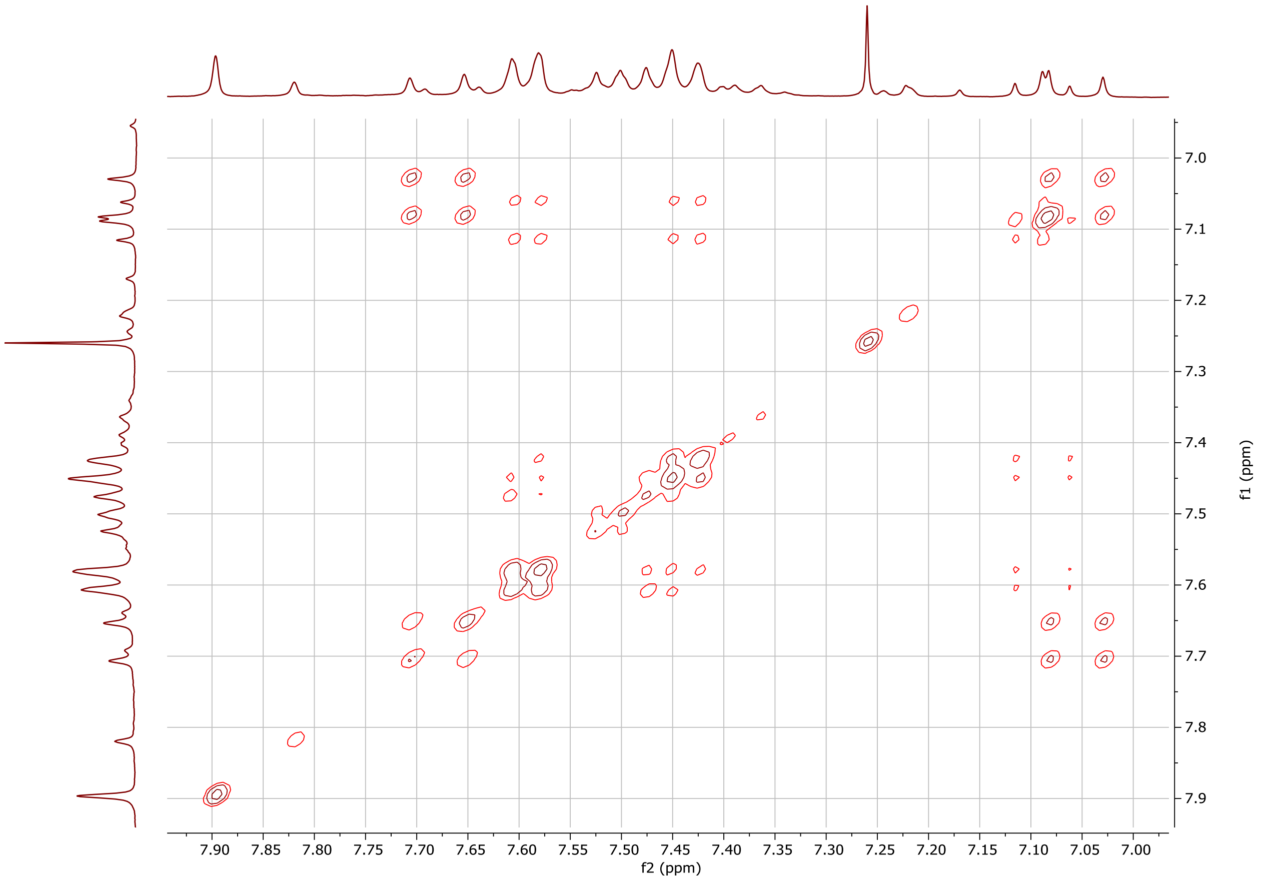

^{1}H-^{1}H COSY NMR spectrum of **3i**

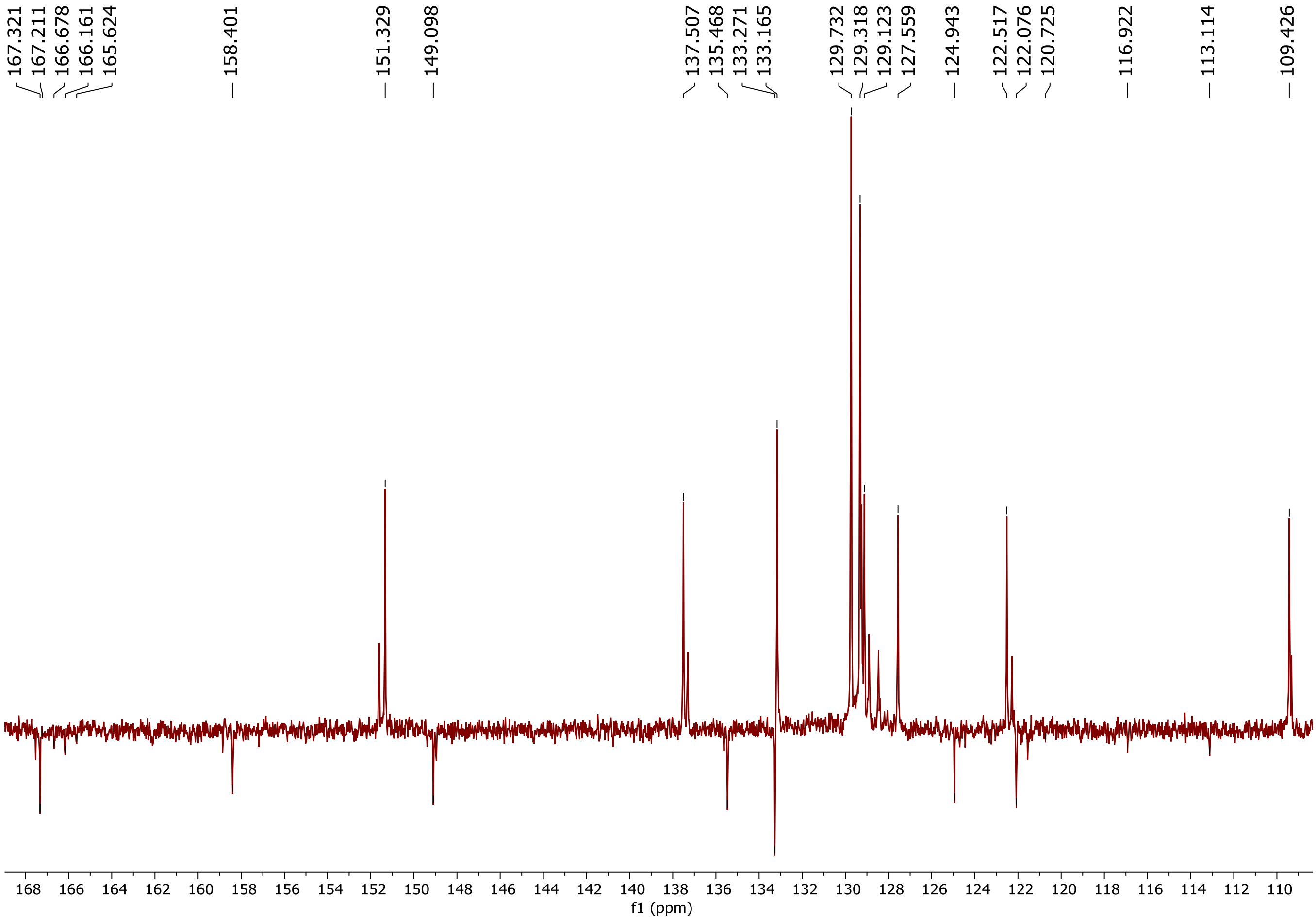

$^{13}C\{^{1}H\}$-(APT) NMR spectrum ($CDCl_3$, 75.47 MHz) of **3i**

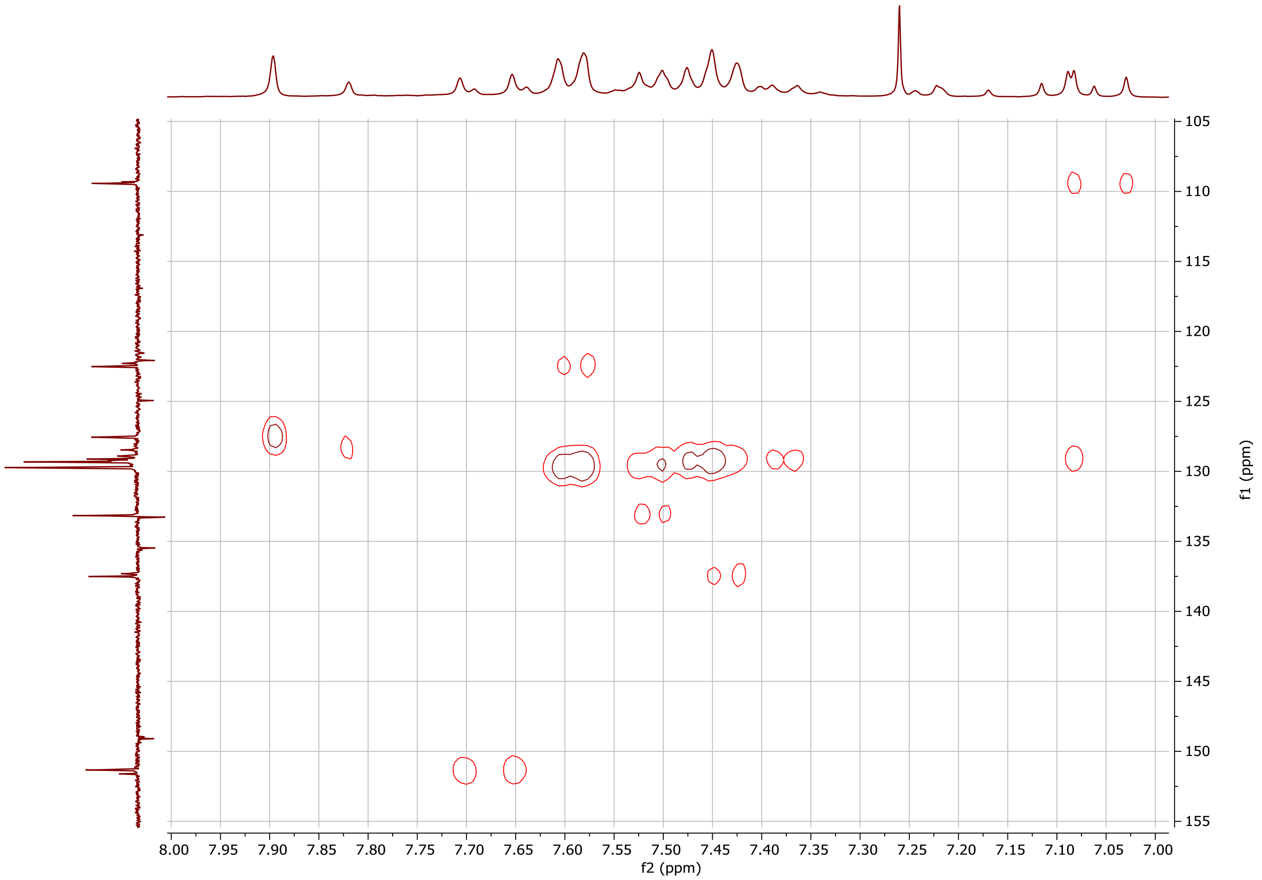

^{1}H-^{13}C HSQC NMR spectrum of **3i**

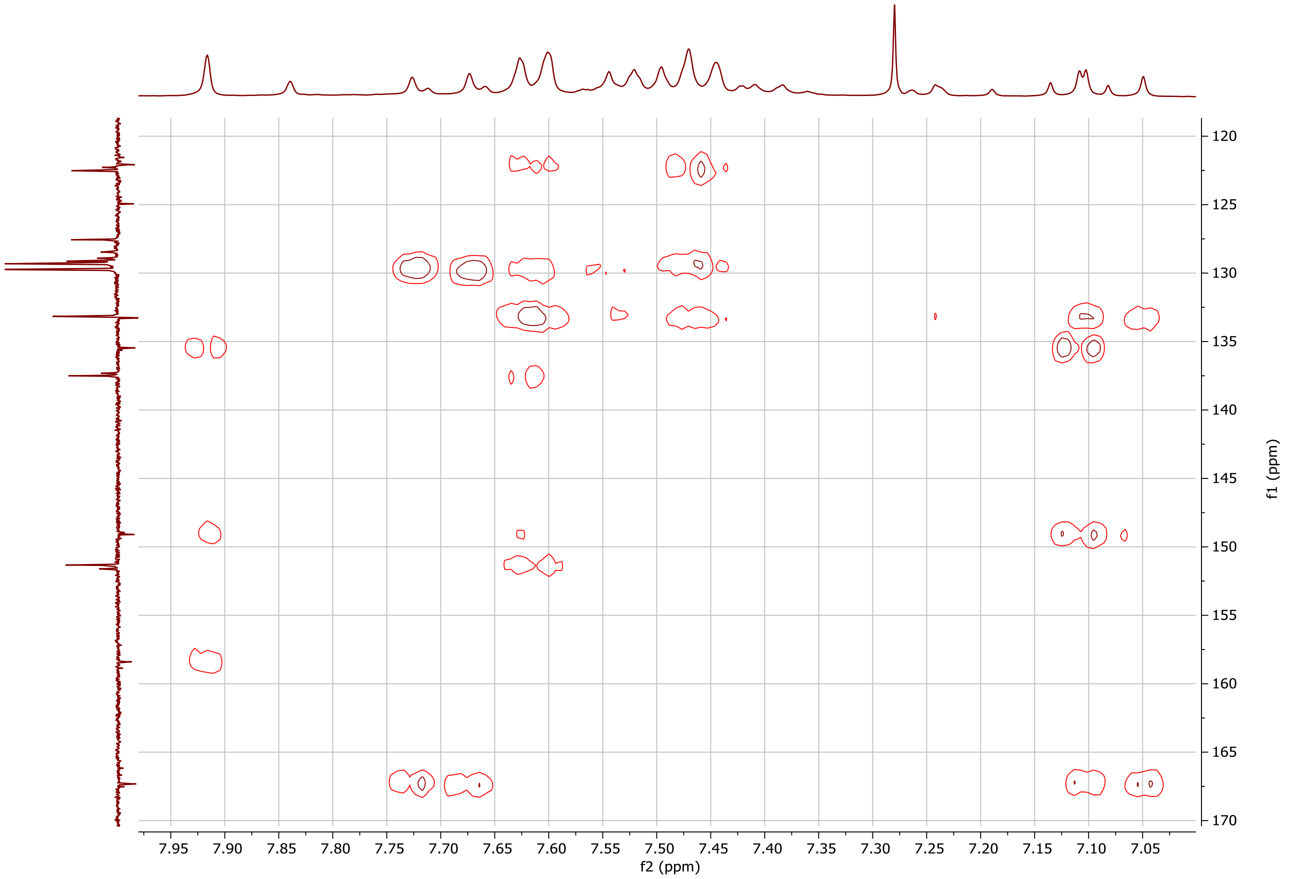

^{1}H-^{13}C HMBC NMR spectrum of **3i**

Orthopalladated dinuclear derivative 3j

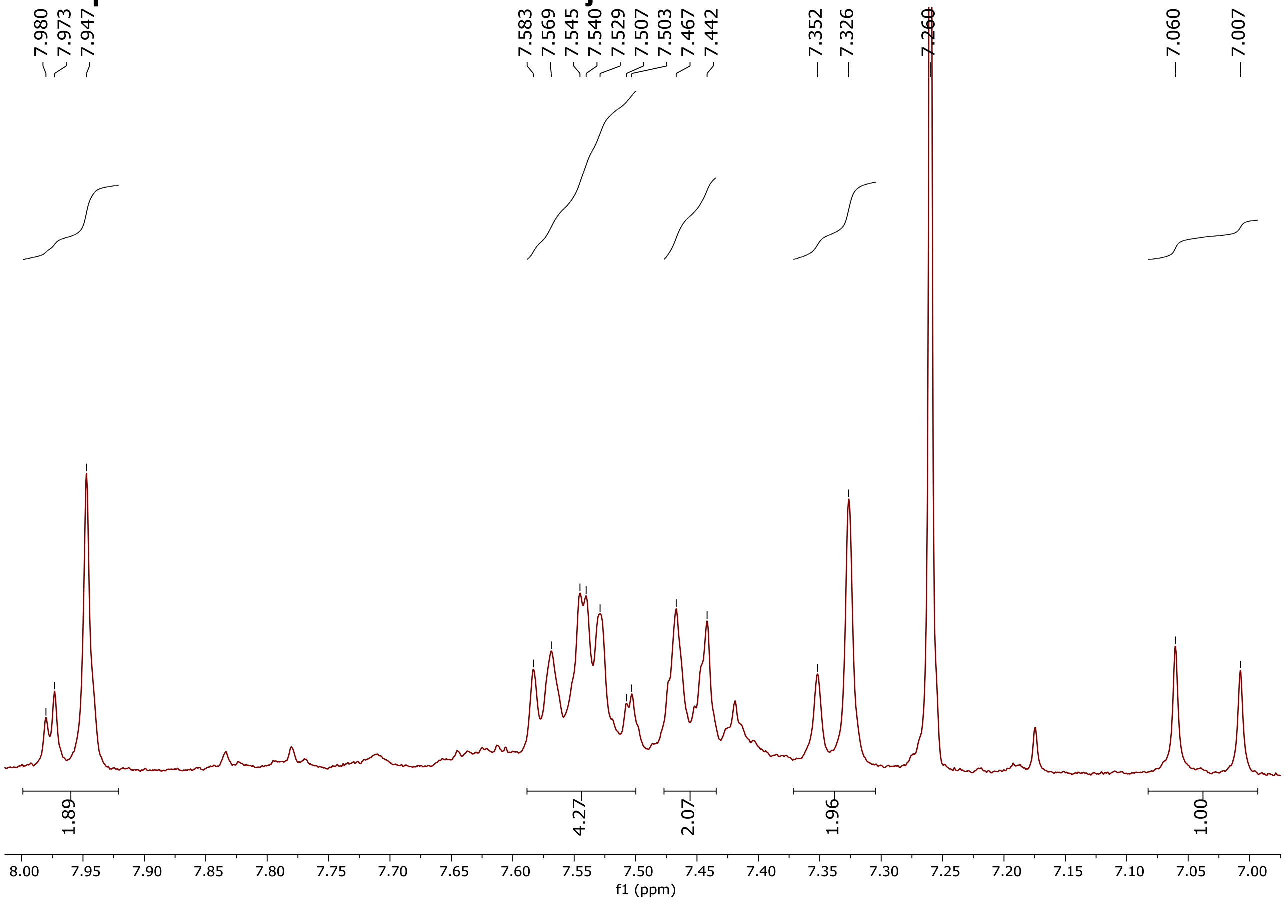

^{1}H NMR ($CDCl_3$, 300.13 MHz) of **3j**

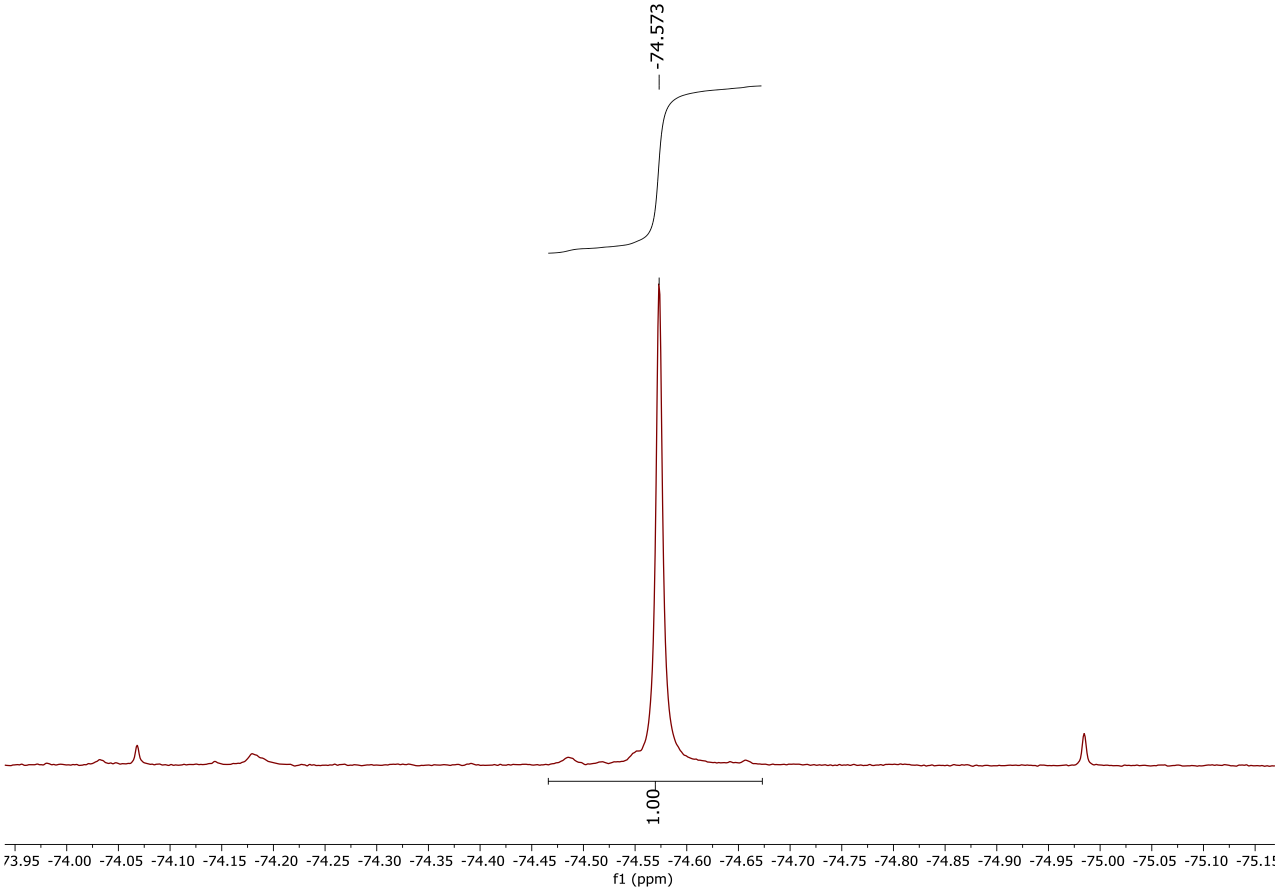

^{19}F-NMR spectrum ($CDCl_3$, 282.40 MHz) of **3j**

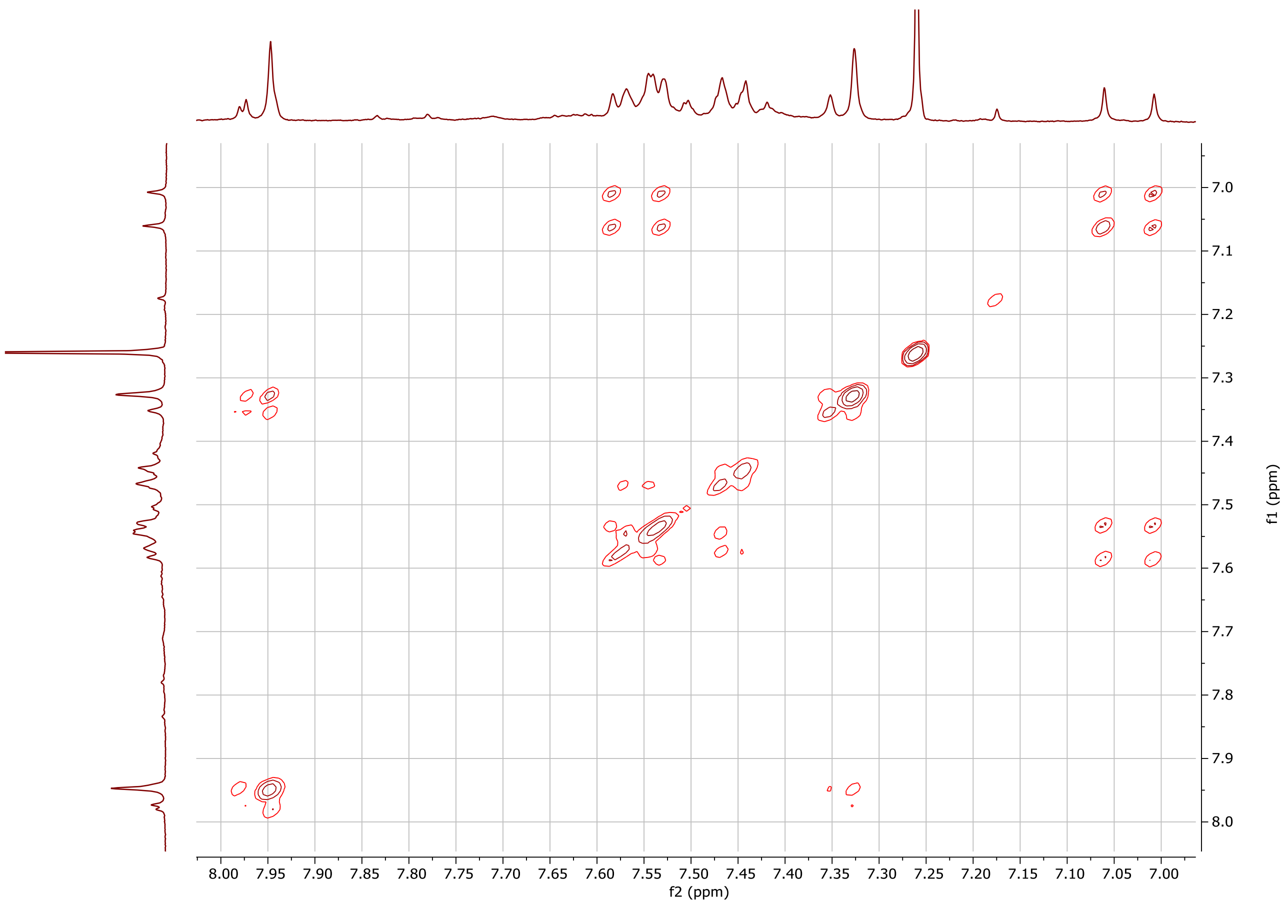

^{1}H-^{1}H COSY NMR spectrum of **3j**

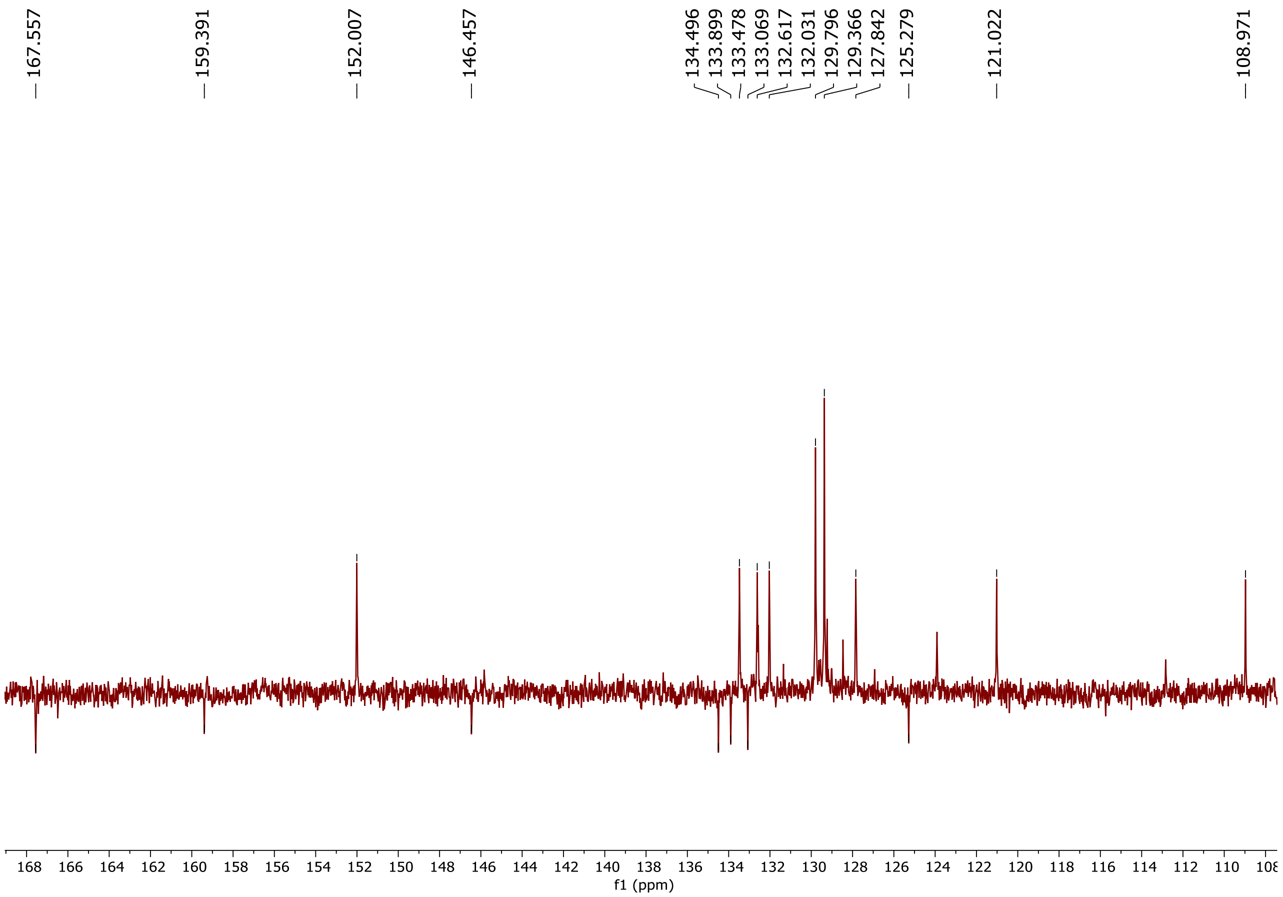

$^{13}C\{^{1}H\}$-(APT) NMR spectrum ($CDCl_3$, 75.47 MHz) of **3j**

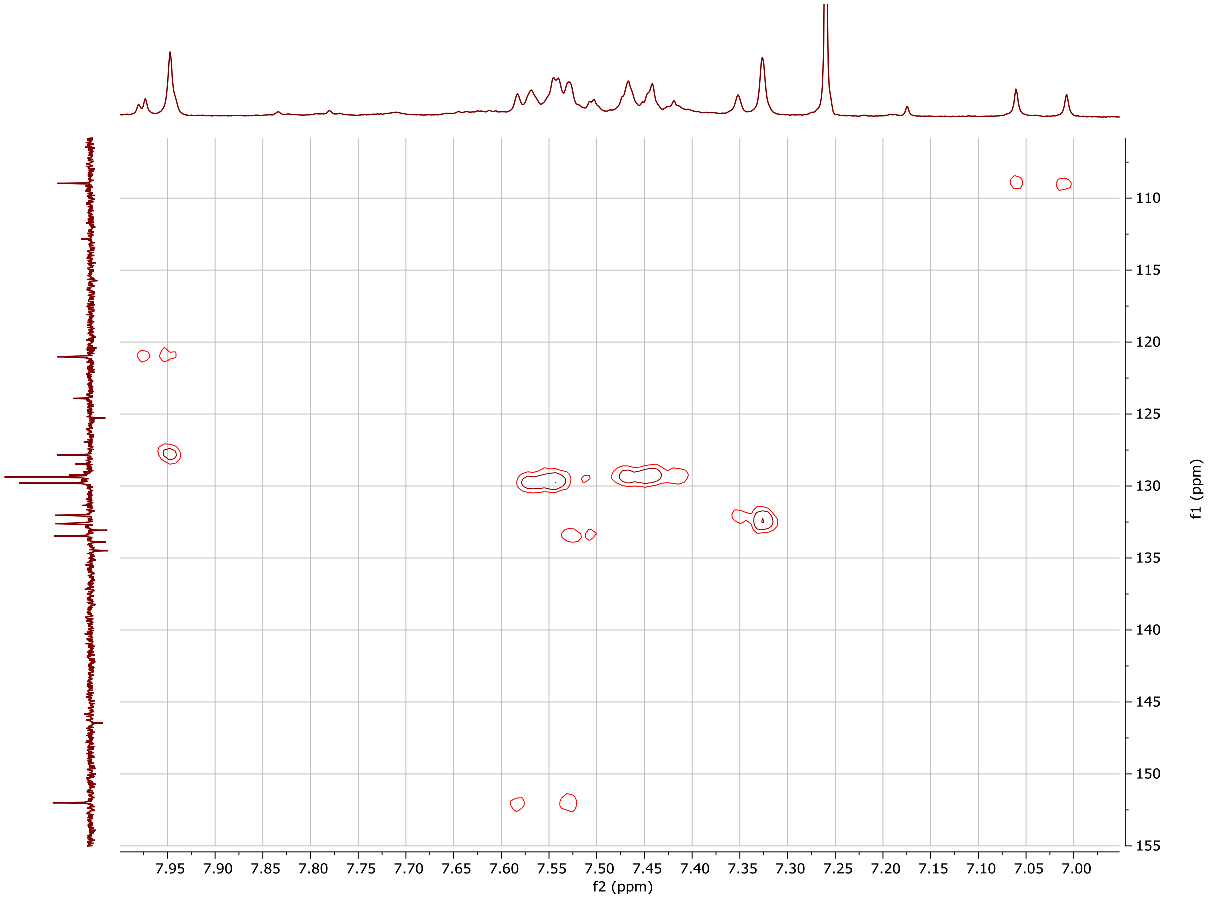

^{1}H-^{13}C HSQC NMR spectrum of **3j**

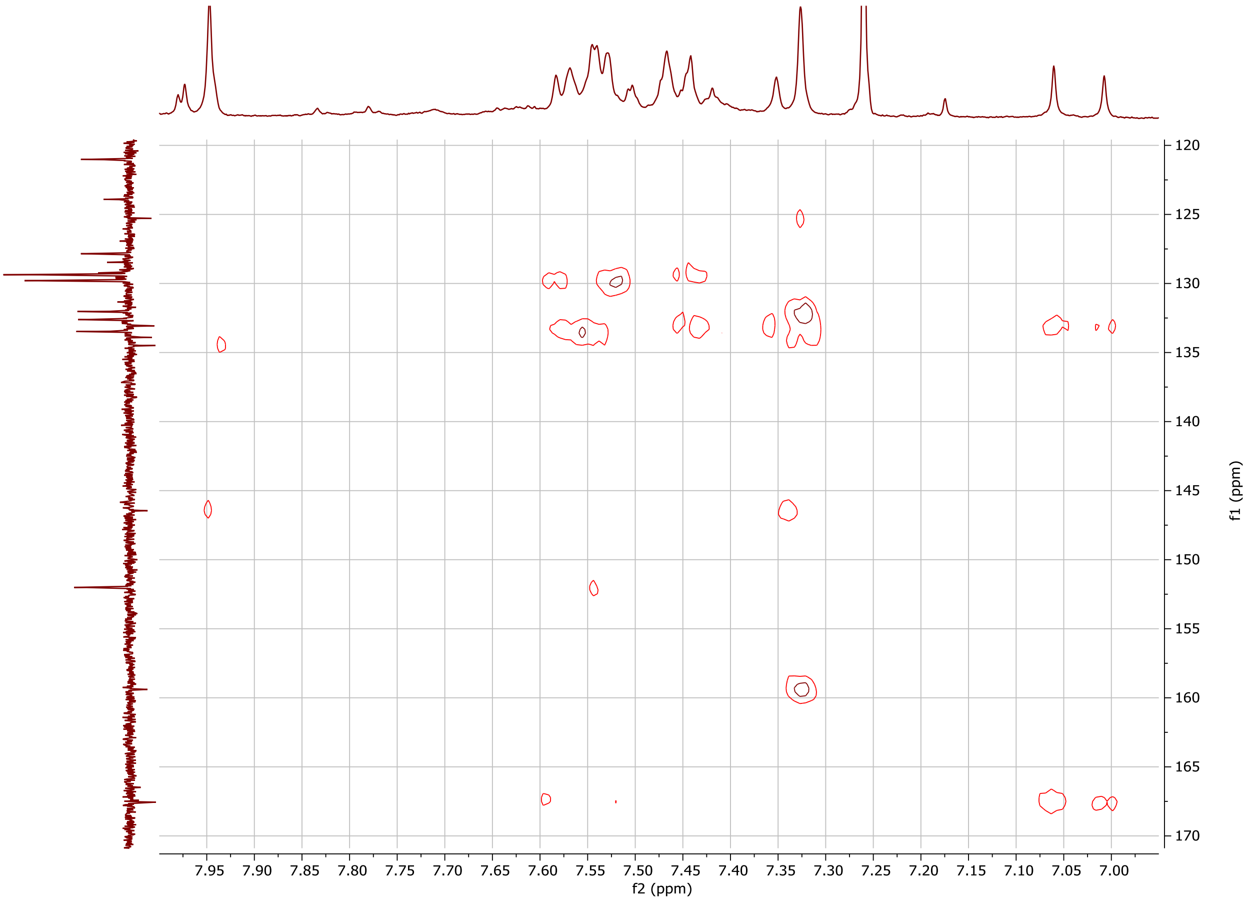

^{1}H-^{13}C HMBC NMR spectrum of **3j**
