# Supplementary material for: Synthesis of esters of diaminotruxillic bis-amino acids by Pd-mediated photocycloaddition of analogs of the Kaede protein chromophore
Source: Beilstein J Org Chem. 2020 May 25;16:1111–23. doi: 10.3762/bjoc.16.98 (PMC7277947; doi:10.3762/bjoc.16.98)
Supplement: File 2 — Copies on NMR spectra of compounds 4 and 5, crystallographic tables of compounds 2c and 4a. [file Beilstein_J_Org_Chem-16-1111-s002.pdf]

BEILSTEIN JOURNAL OF ORGANIC CHEMISTRY

Supporting Information

for

Synthesis of esters of diaminotruxillic bis-amino acids by Pd-mediated photocycloaddition of analogs of the Kaede protein chromophore

Esteban P. Urriolabeitia, Pablo Sánchez, Alexandra Pop, Cristian Silvestru, Eduardo Laga, Ana I. Jiménez and Carlos Cativiela

Beilstein J. Org. Chem. **2020,** *16,* 1111–1123. doi:10.3762/bjoc.16.98

Copies on NMR spectra of compounds 4 and 5, crystallographic tables of compounds 2c and 4a

License and Terms: This is a supporting information file under the terms of the Creative Commons Attribution License (http://creativecommons.org/licenses/by/4.0). Please note that the reuse, redistribution and reproduction in particular requires that the authors and source are credited.
The license is subject to the *Beilstein Journal of Organic Chemistry* terms and conditions: (https://www.beilstein-journals.org/bjoc)

3. NMR spectra of orthopalladated dinuclear cyclobutane complexes 4a–4f, 4h–4j.

Orthopalladated dinuclear cyclobutane 4a

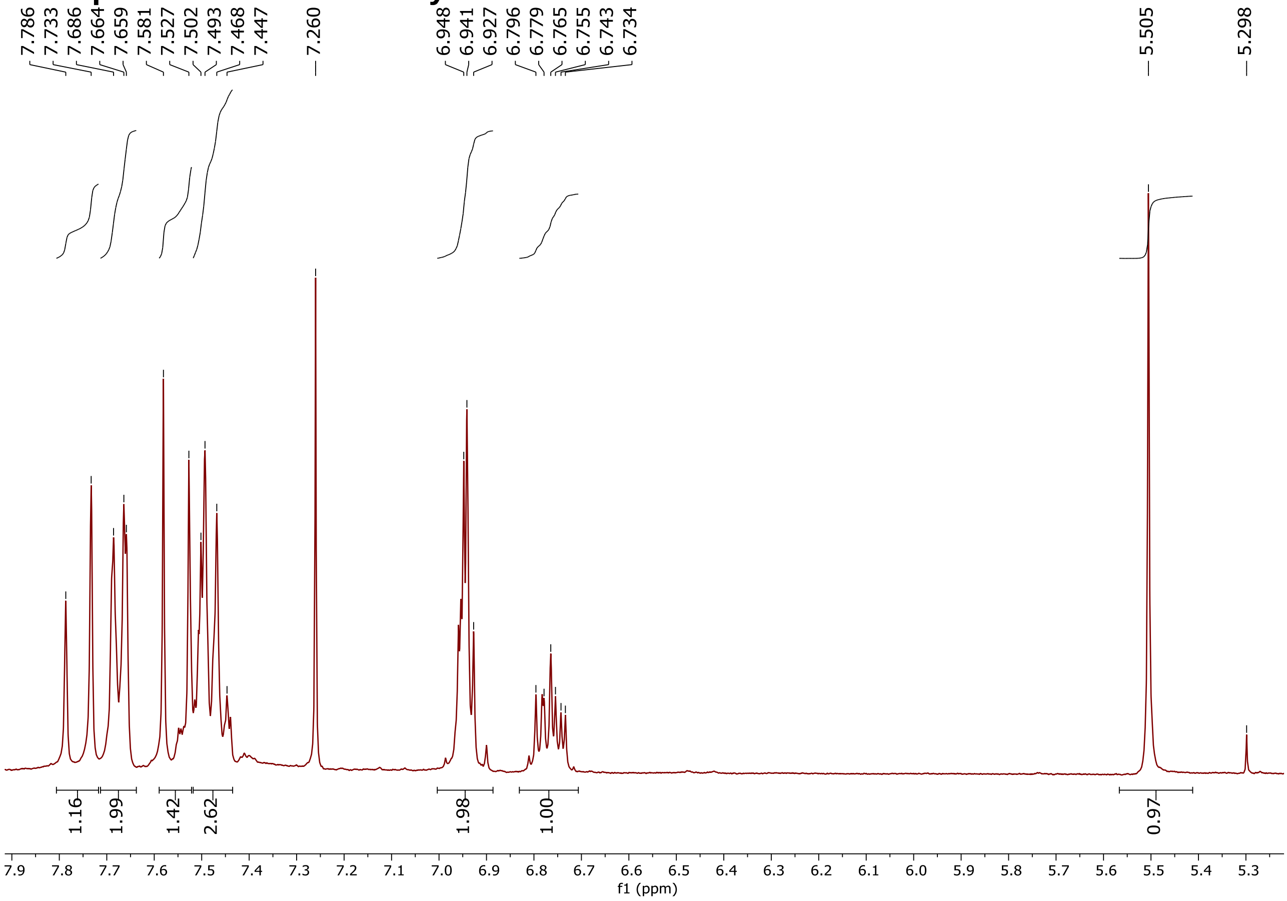

^{1}H NMR (CDCl$_3$, 300.13 MHz) of **4a**

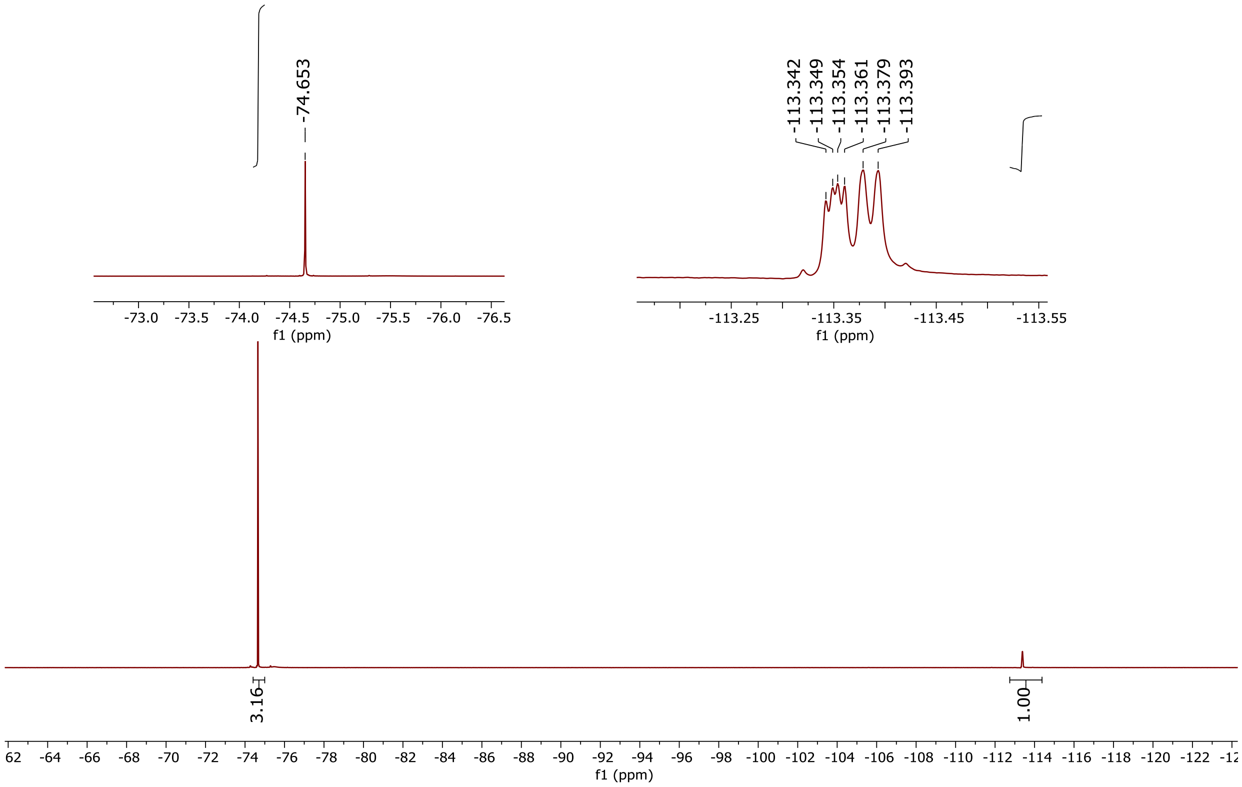

^{19}F NMR spectrum ($CDCl_3$, 282.40 MHz) of **4a**

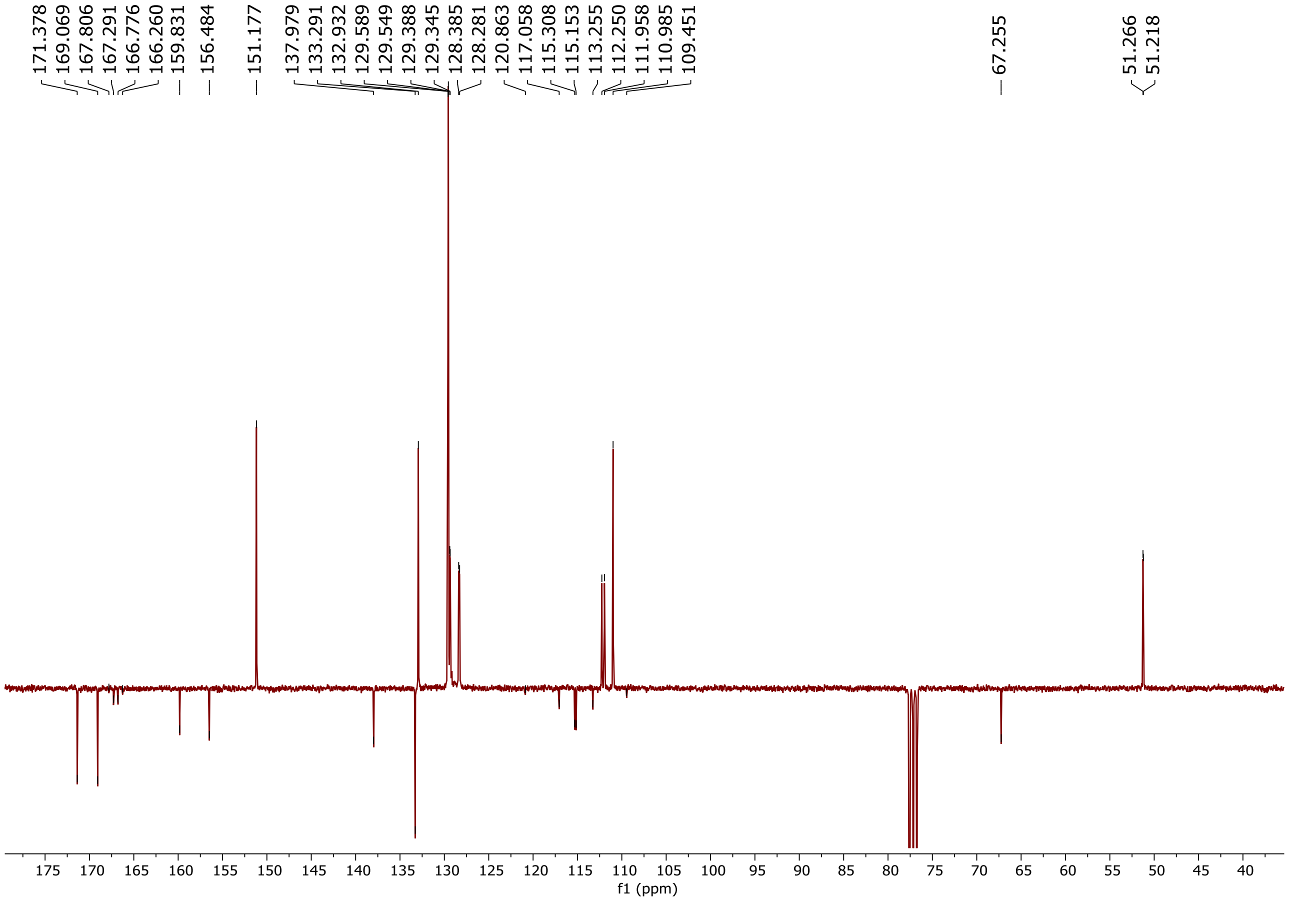

^{13}C{^{1}H}-(APT) NMR spectrum ($CDCl_3$, 75.47 MHz) of **4a**

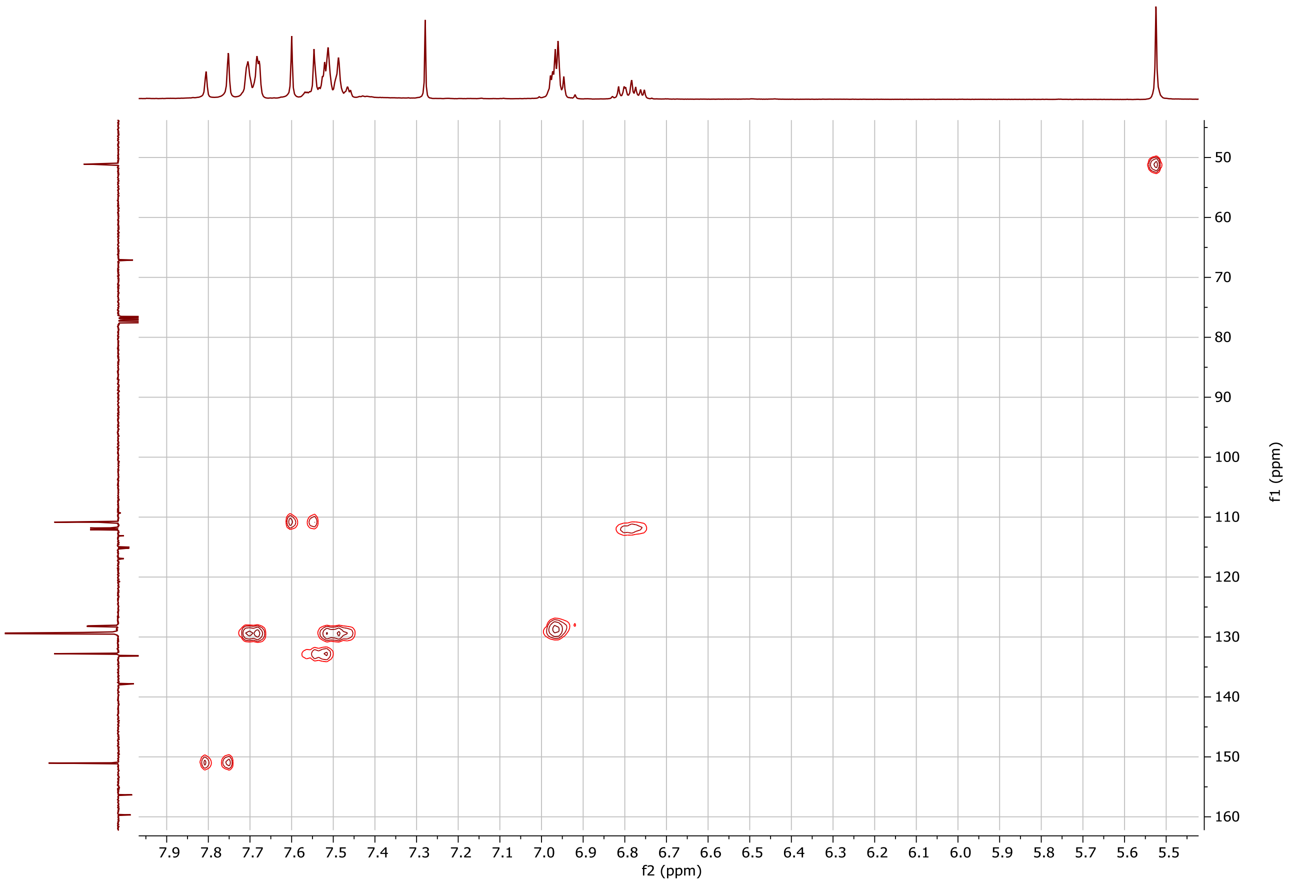

^{1}H-^{13}C HSQC NMR spectrum of **4a**

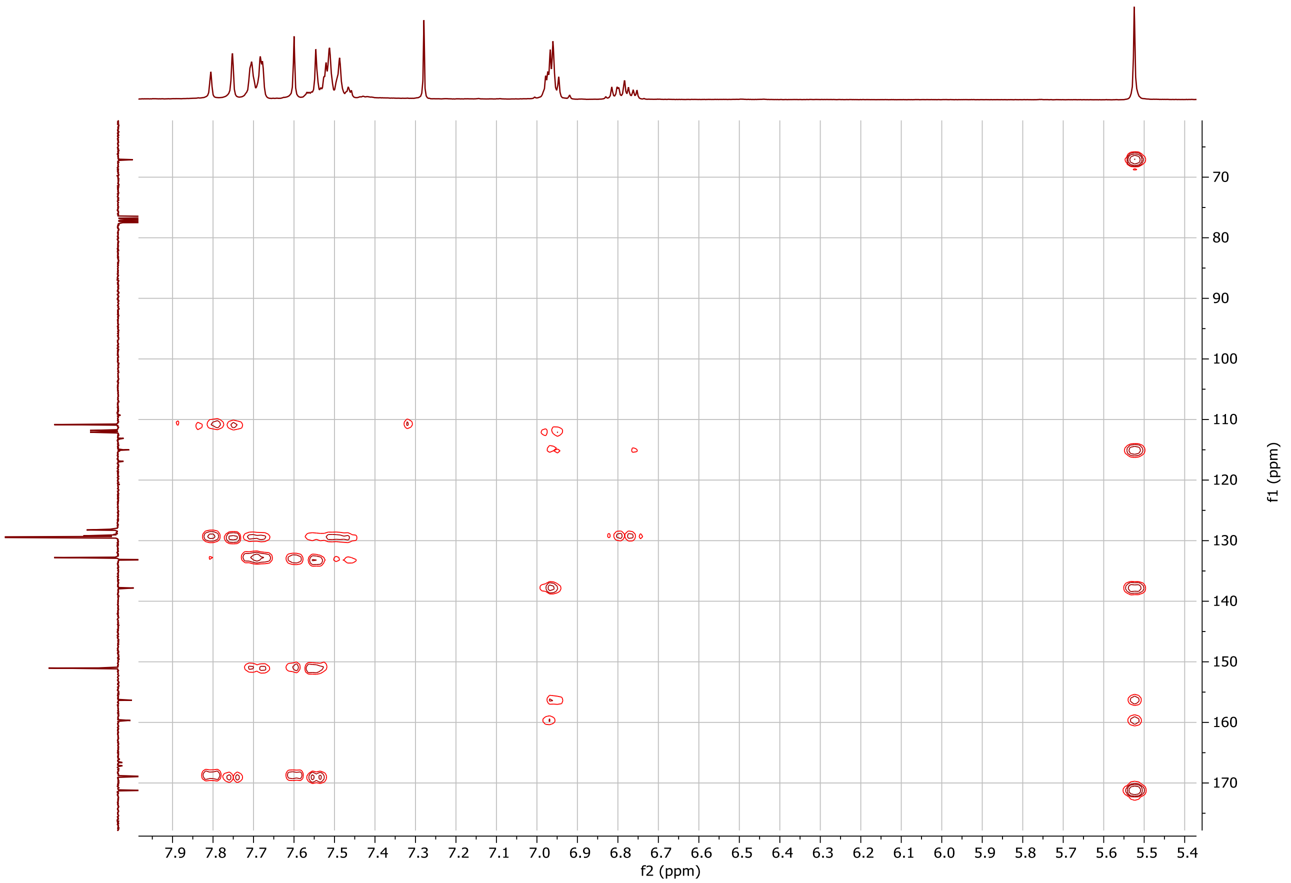

^{1}H-^{13}C HMBC NMR spectrum of **4a**

Orthopalladated dinuclear cyclobutane 4b

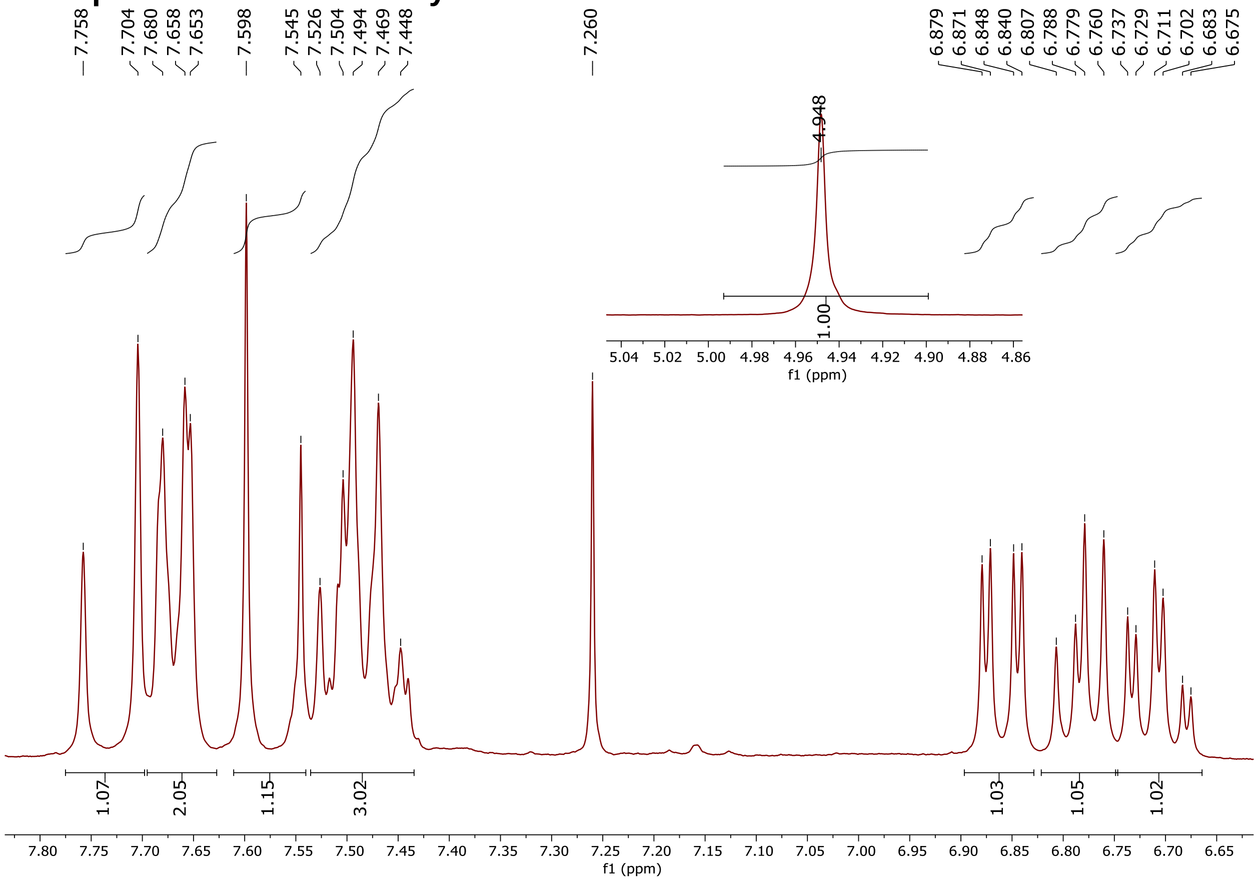

^{1}H NMR ($CDCl_3$, 300.13 MHz) of **4b**

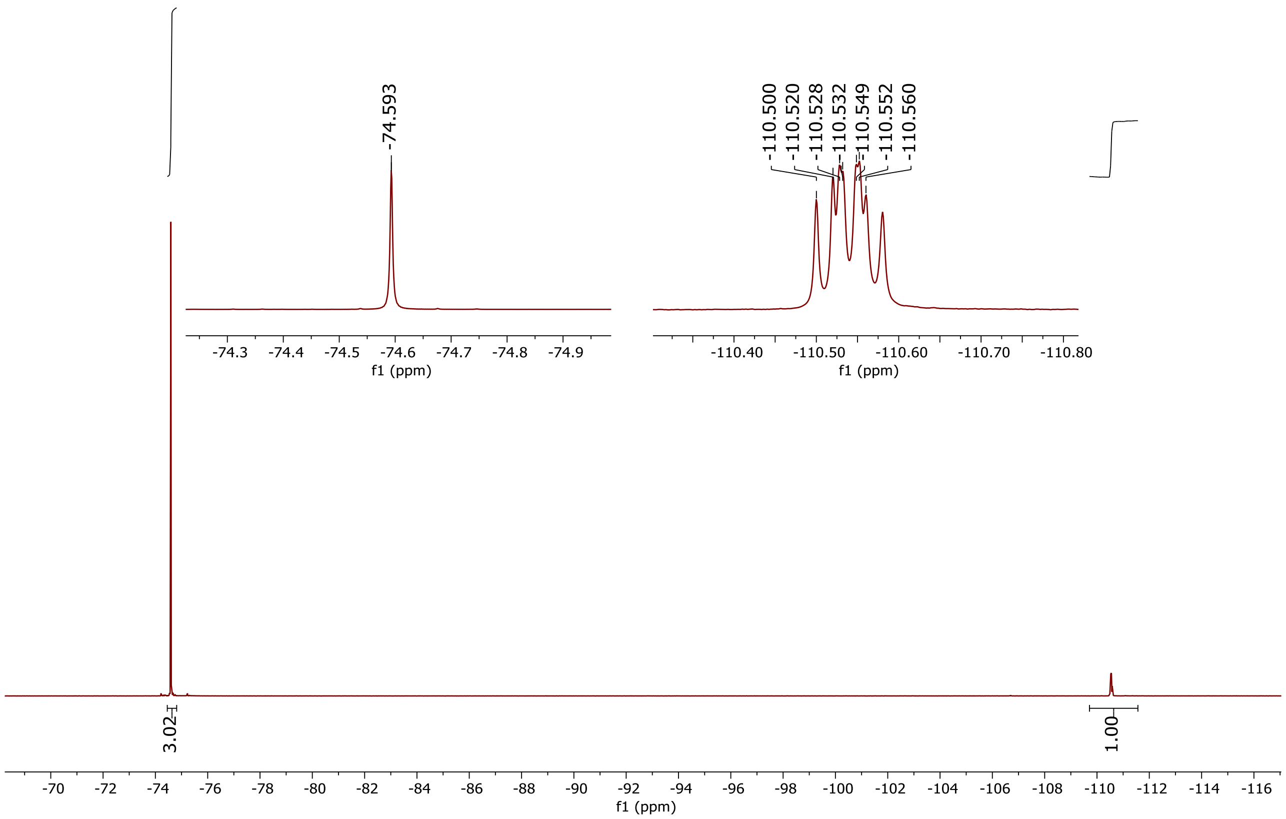

^{19}F NMR spectrum ($CDCl_3$, 282.40 MHz) of **4b**

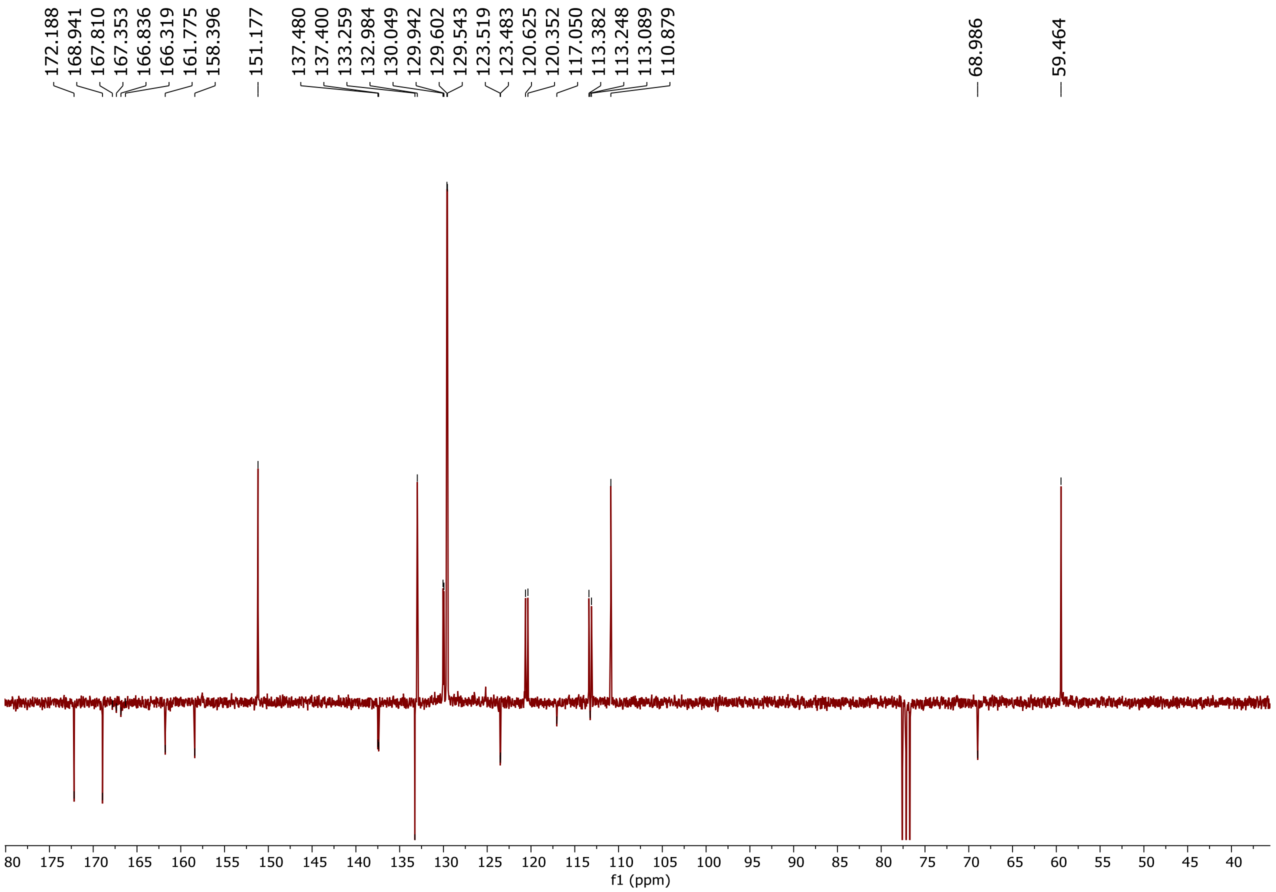

$^{13}C\{^{1}H\}$-(APT) NMR spectrum ($CDCl_3$, 75.47 MHz) of **4b**

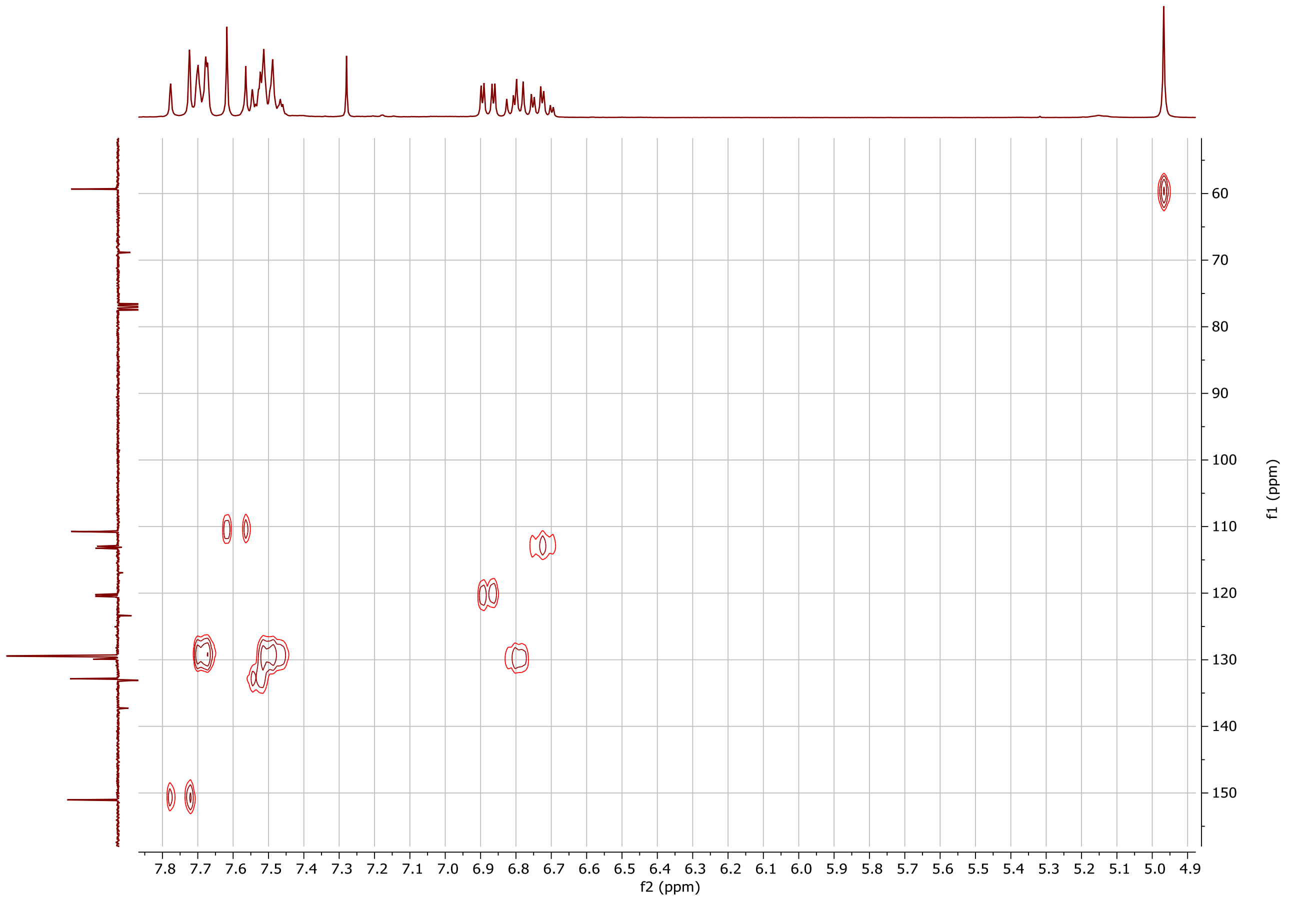

^{1}H-^{13}C HSQC NMR spectrum of **4b**

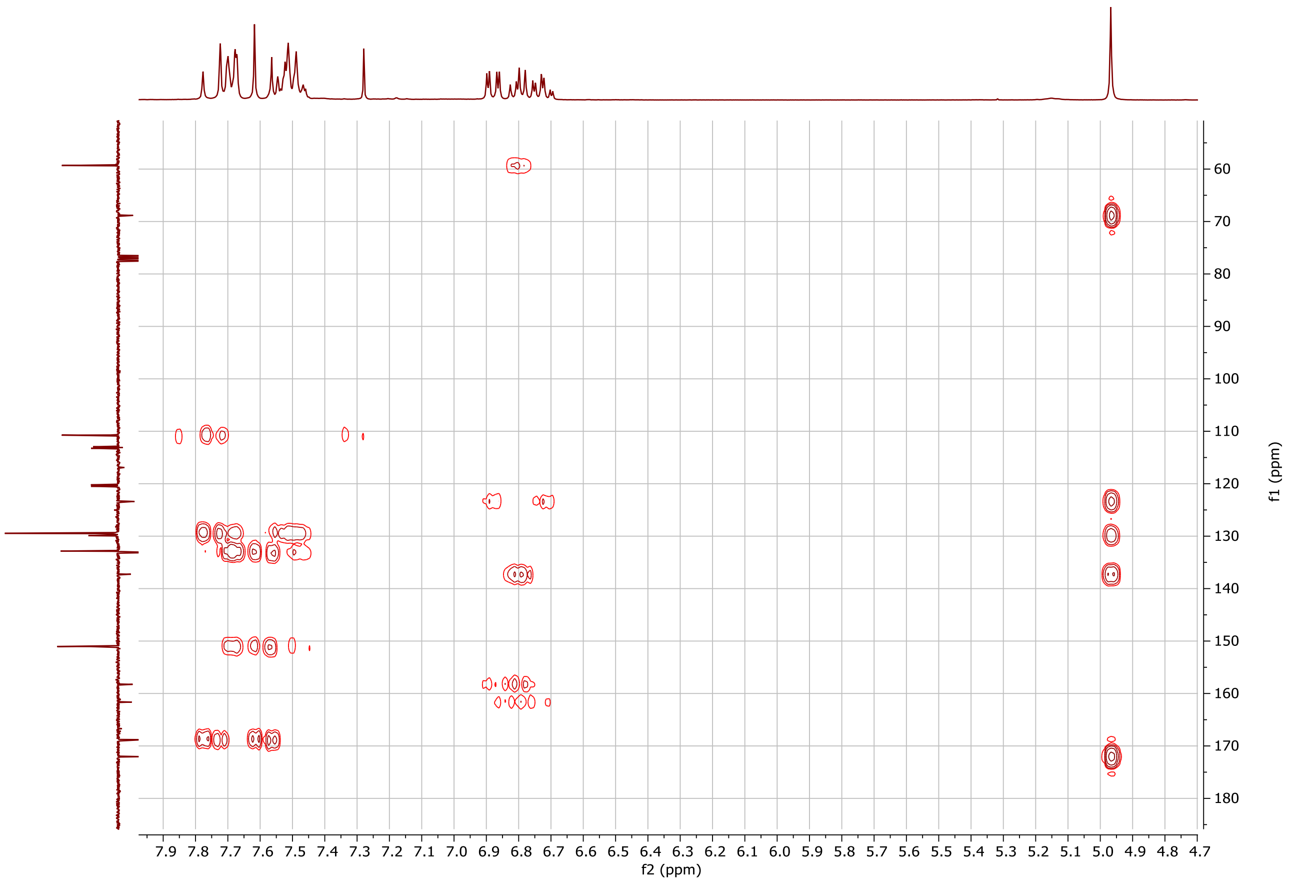

^{1}H-^{13}C HMBC NMR spectrum of **4b**

Orthopalladated dinuclear cyclobutane 4c

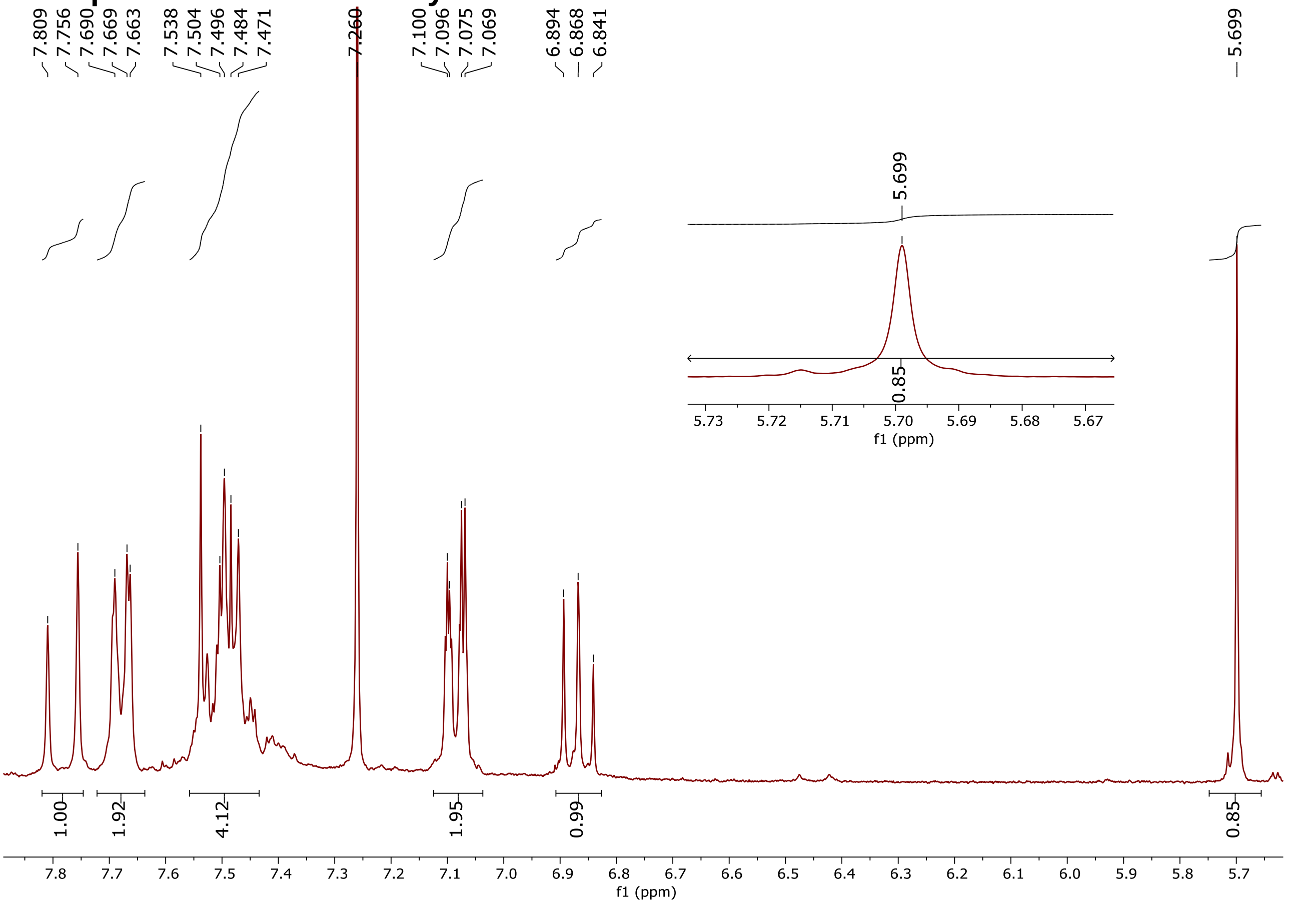

^{1}H NMR ($CDCl_3$, 300.13 MHz) of **4c**

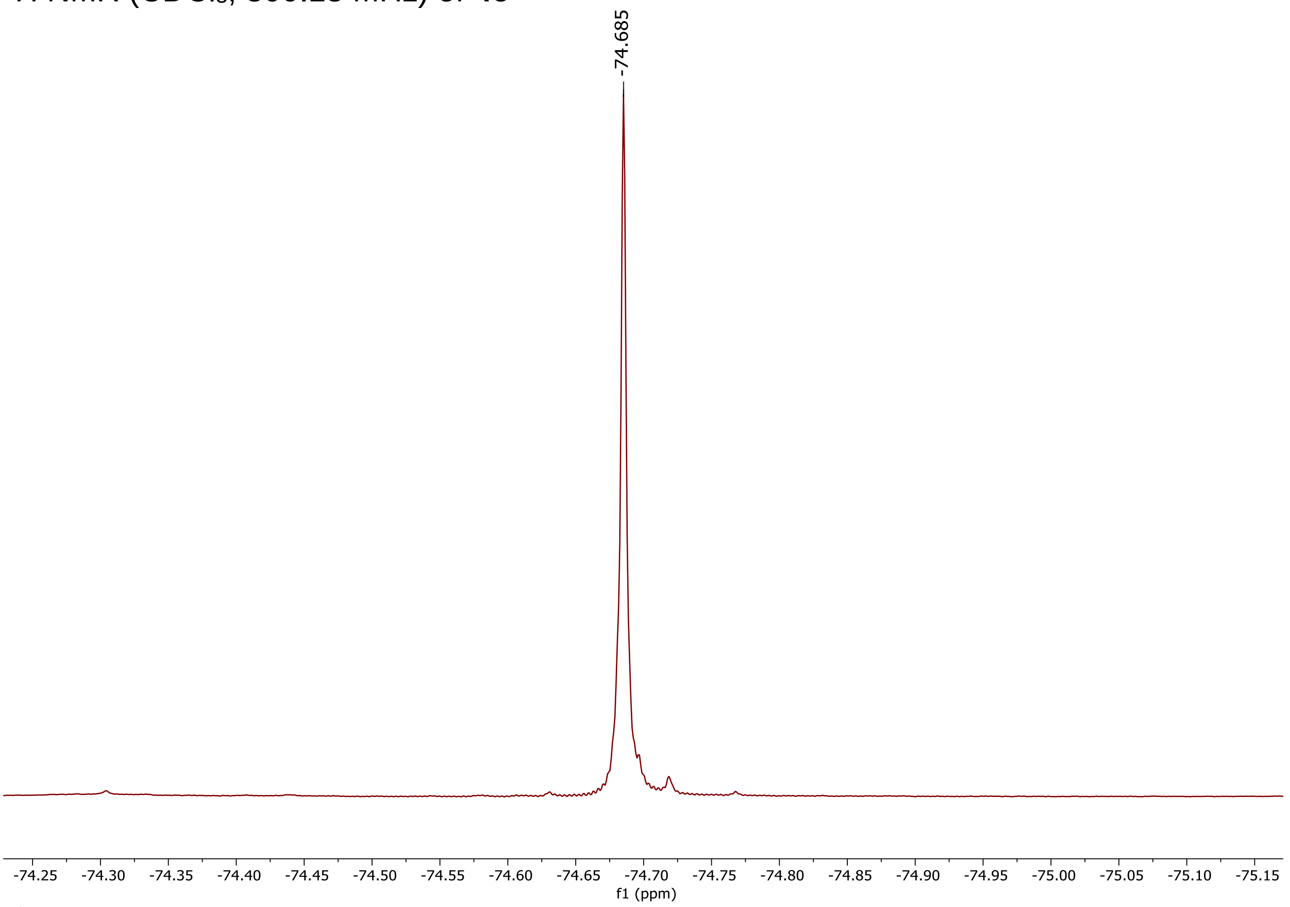

^{19}F NMR spectrum ($CDCl_3$, 282.40 MHz) of **4c**

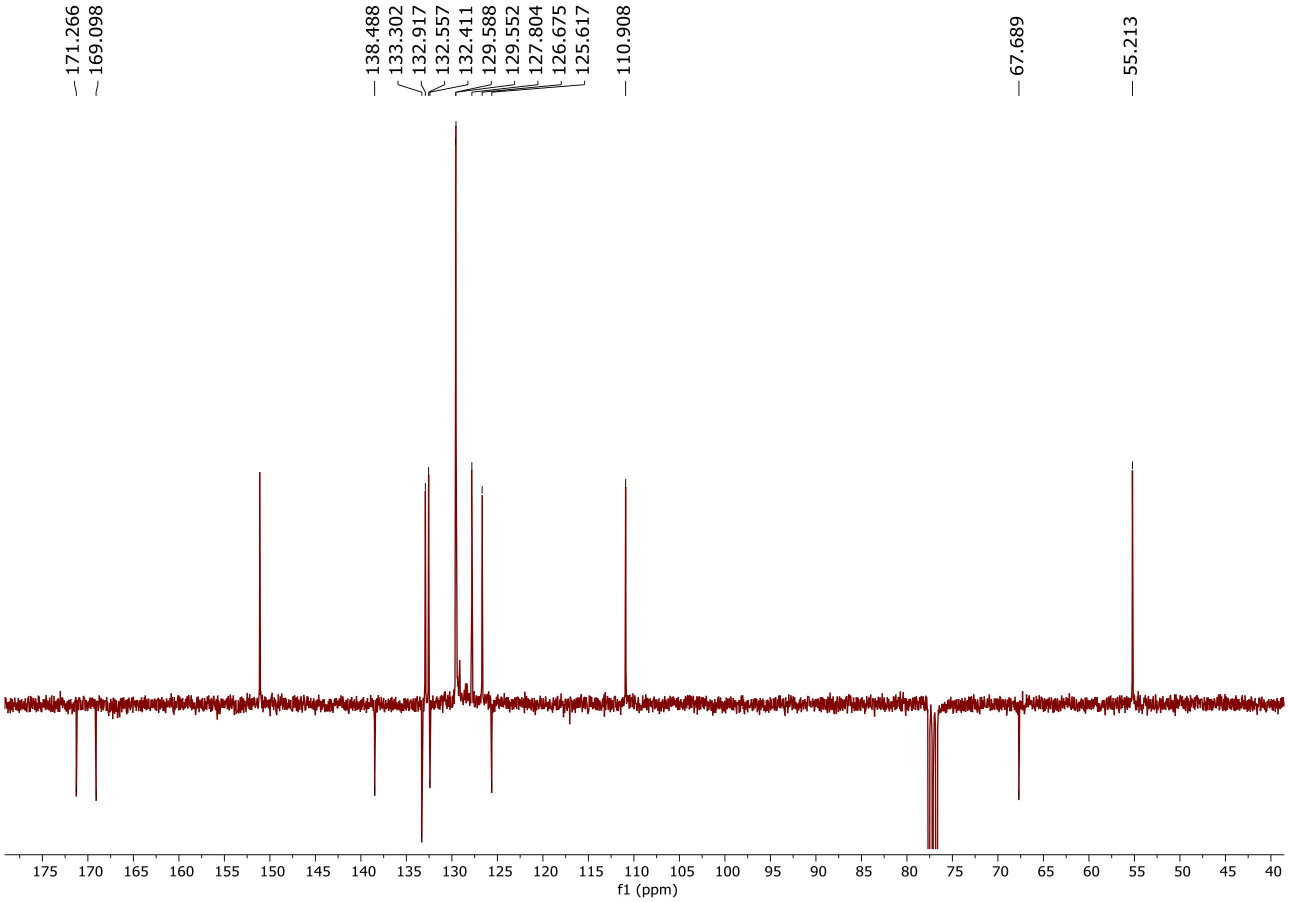

$^{13}C\{^{1}H\}$-(APT) NMR spectrum ($CDCl_3$, 75.47 MHz) of **4c**

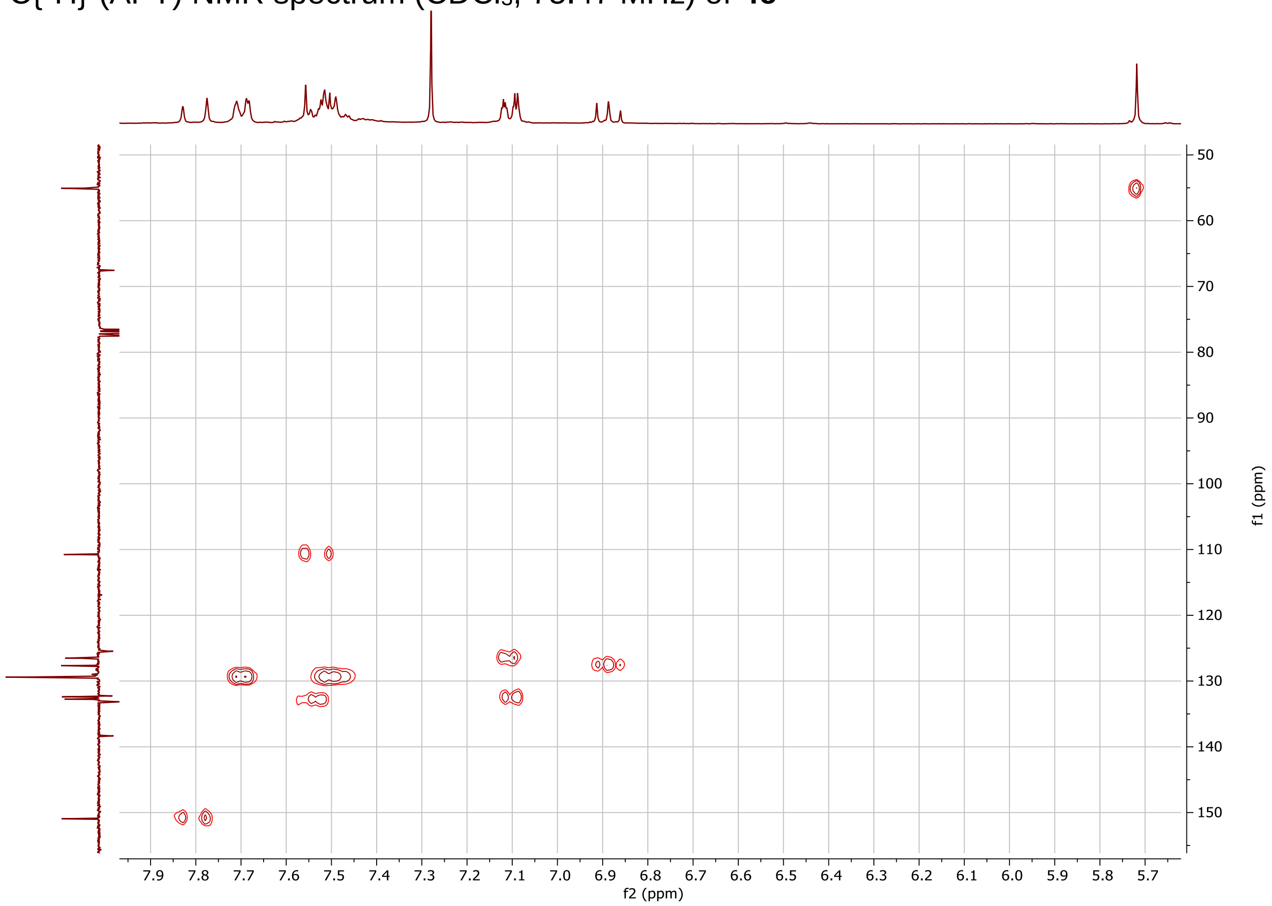

^{1}H-^{13}C HSQC NMR spectrum of **4c**

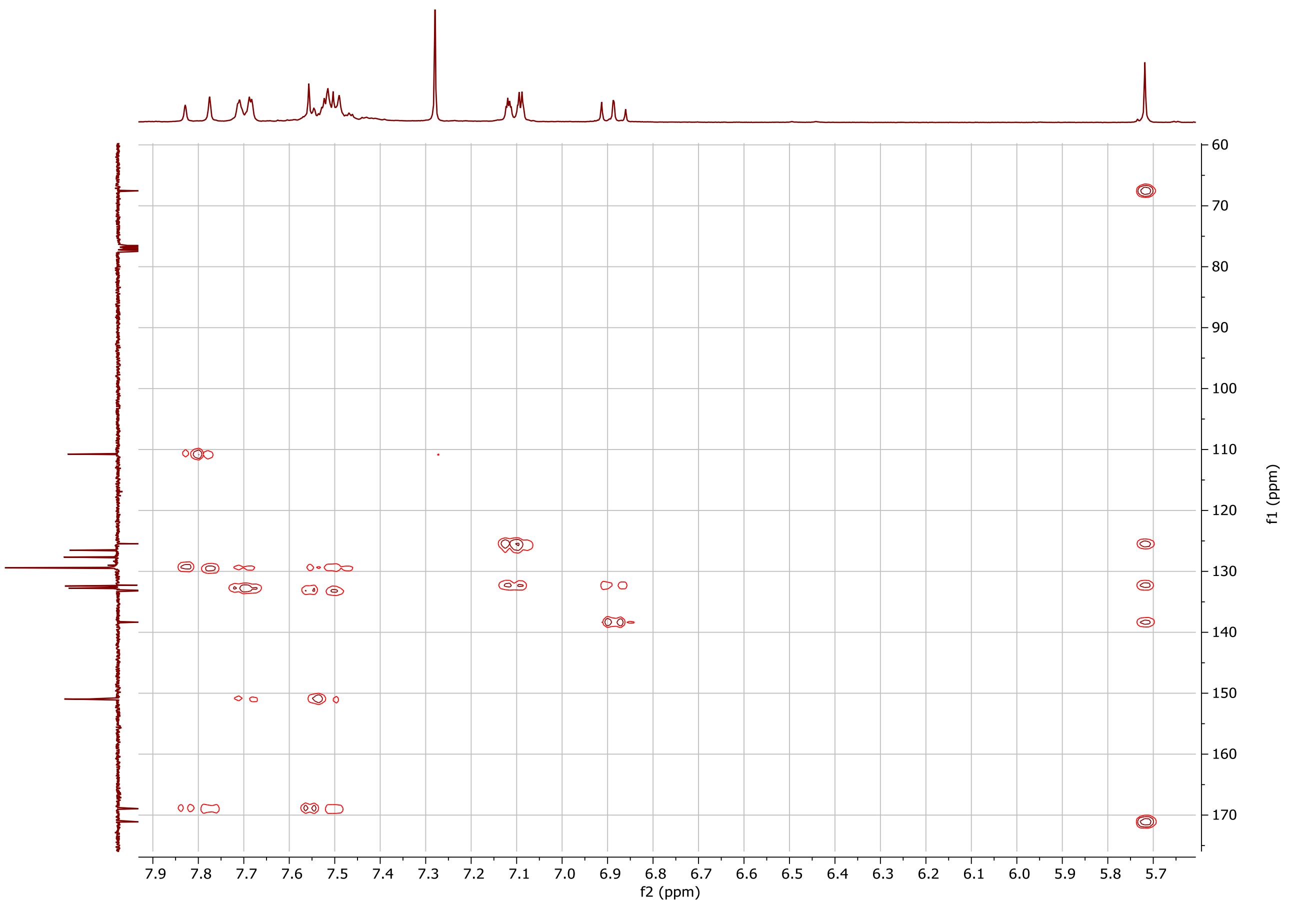

^{1}H-^{13}C HMBC NMR spectrum of **4c**

Orthopalladated dinuclear cyclobutane 4d

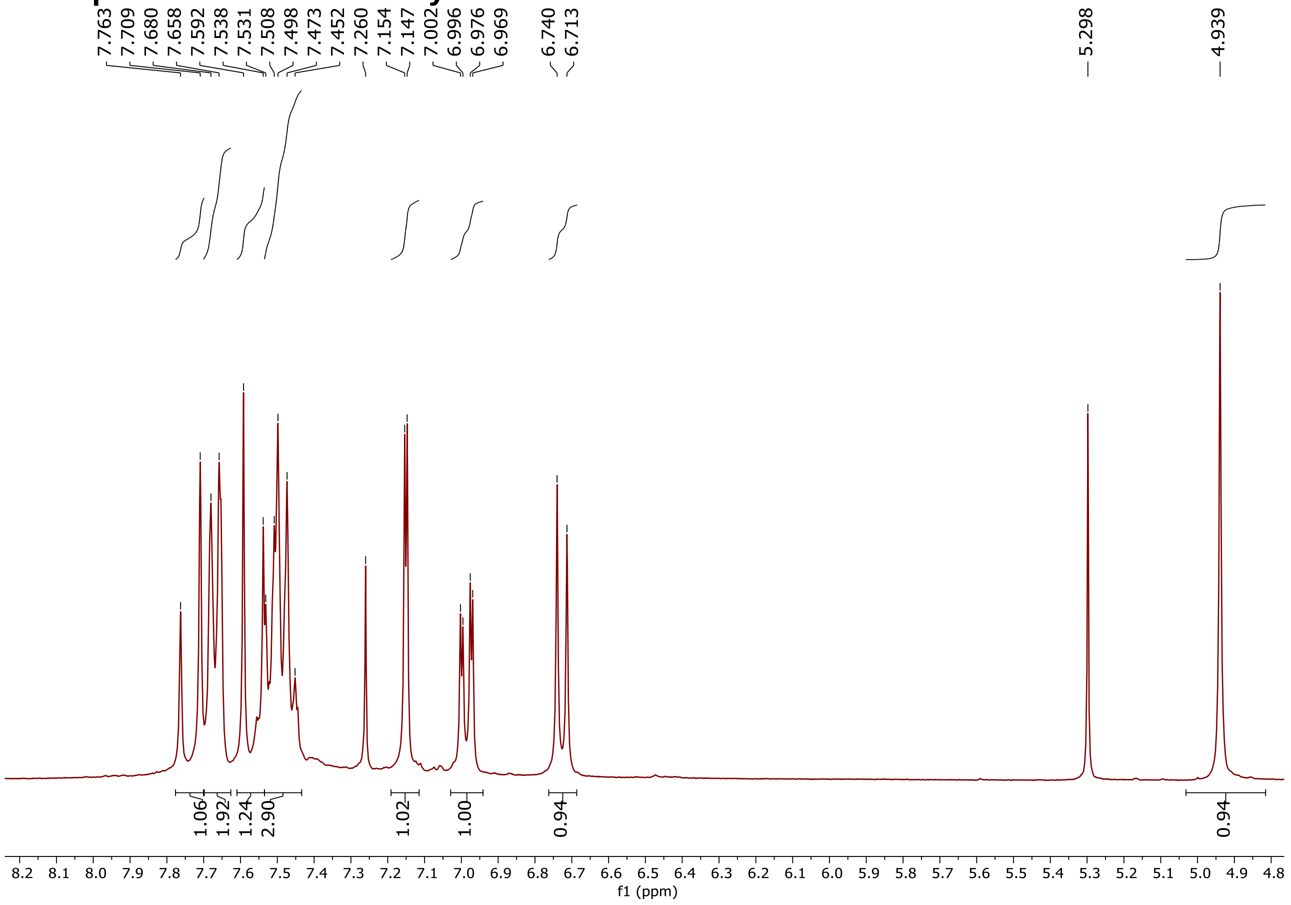

^{1}H NMR ($CDCl_3$, 300.13 MHz) of **4d**

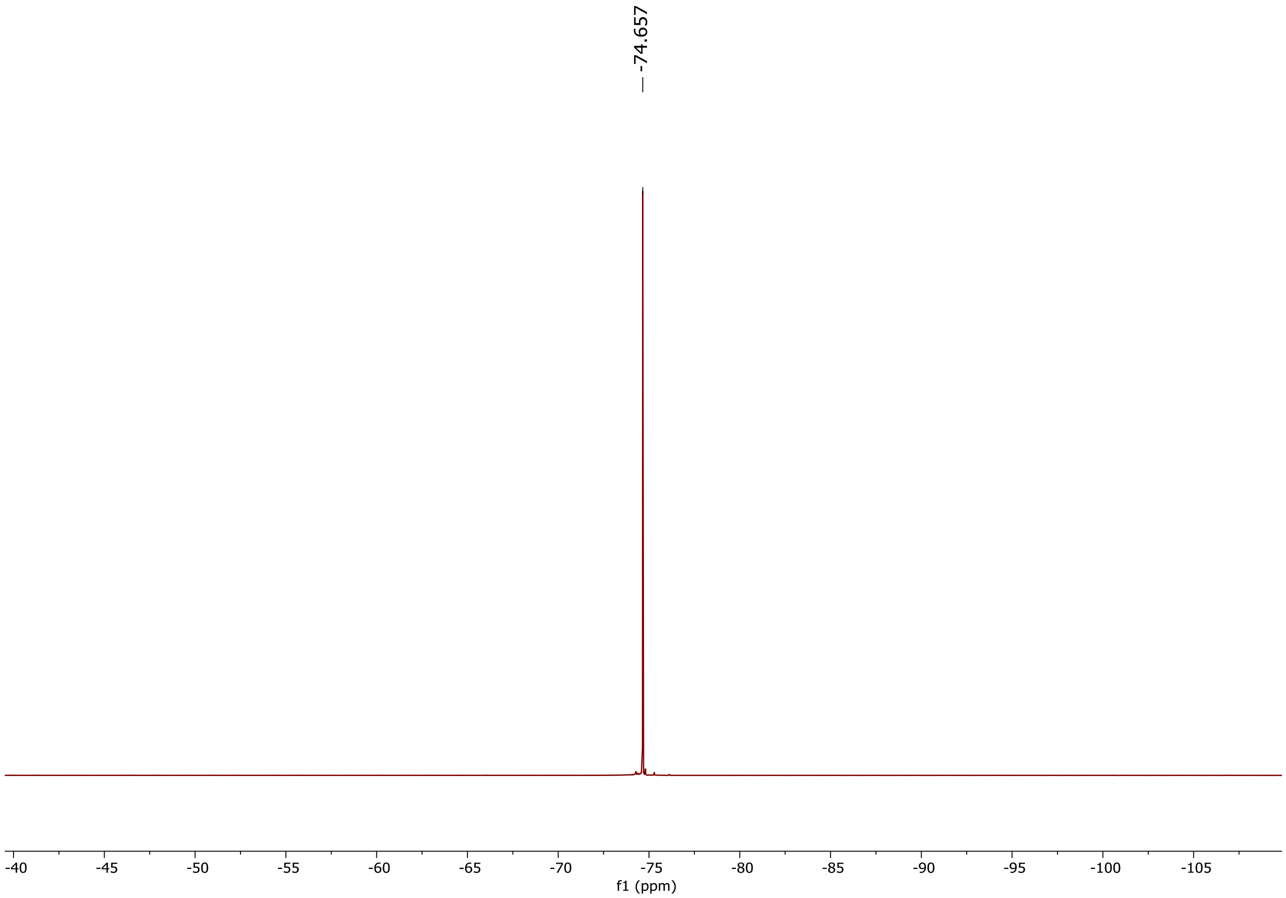

^{19}F NMR spectrum ($CDCl_3$, 282.40 MHz) of **4d**

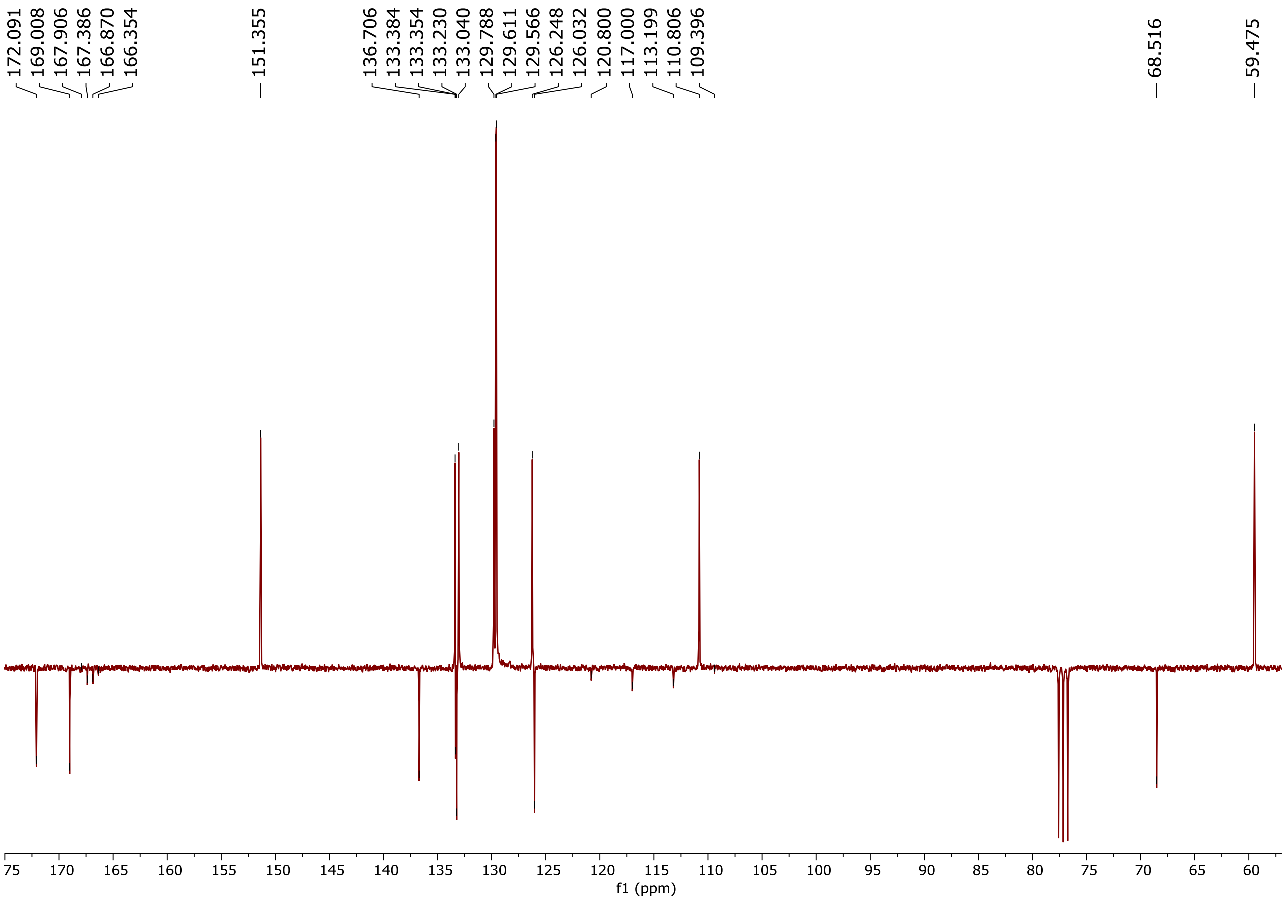

$^{13}C\{^{1}H\}$-(APT) NMR spectrum ($CDCl_3$, 75.47 MHz) of **4d**

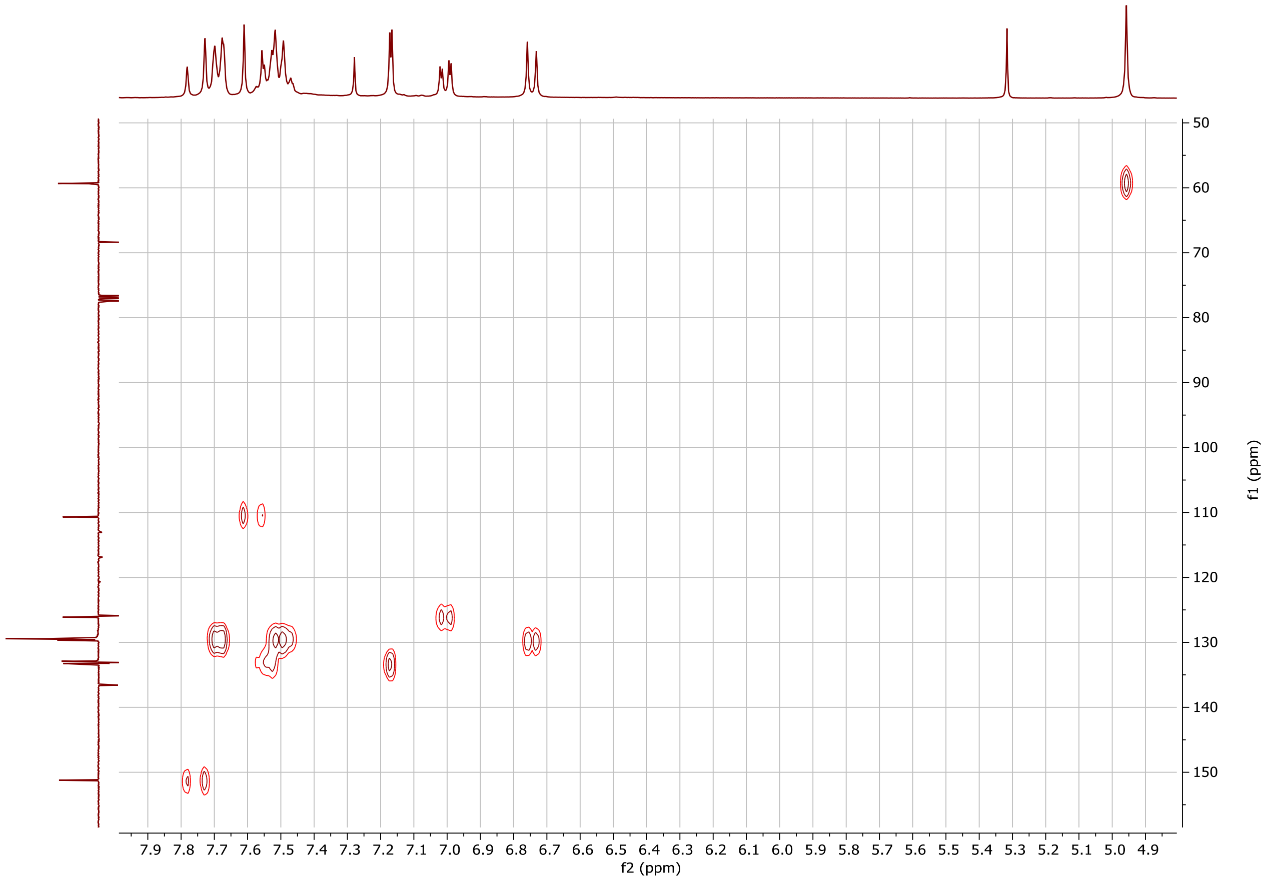

^{1}H-^{13}C HSQC NMR spectrum of **4d**

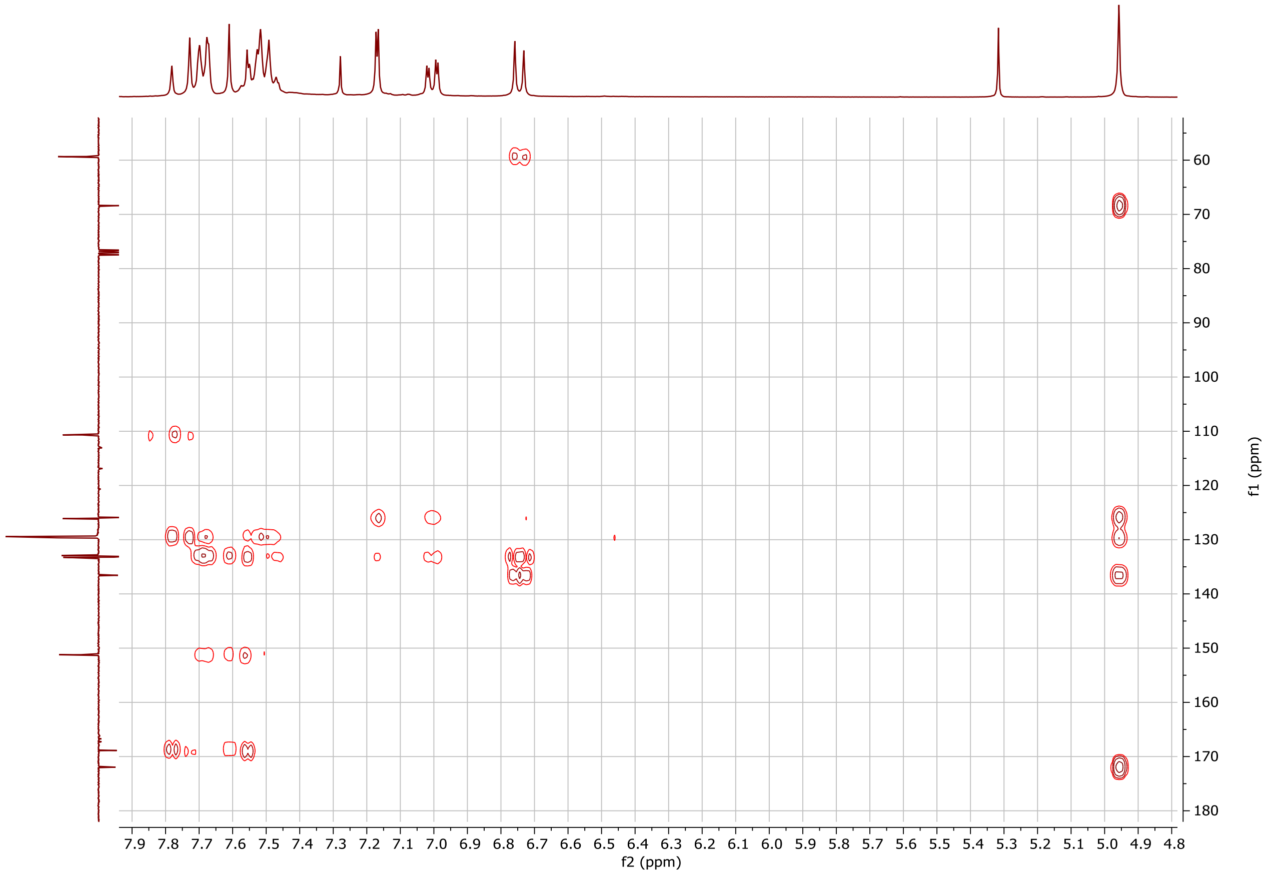

^{1}H-^{13}C HMBC NMR spectrum of **4d**

Orthopalladated dinuclear cyclobutane 4e

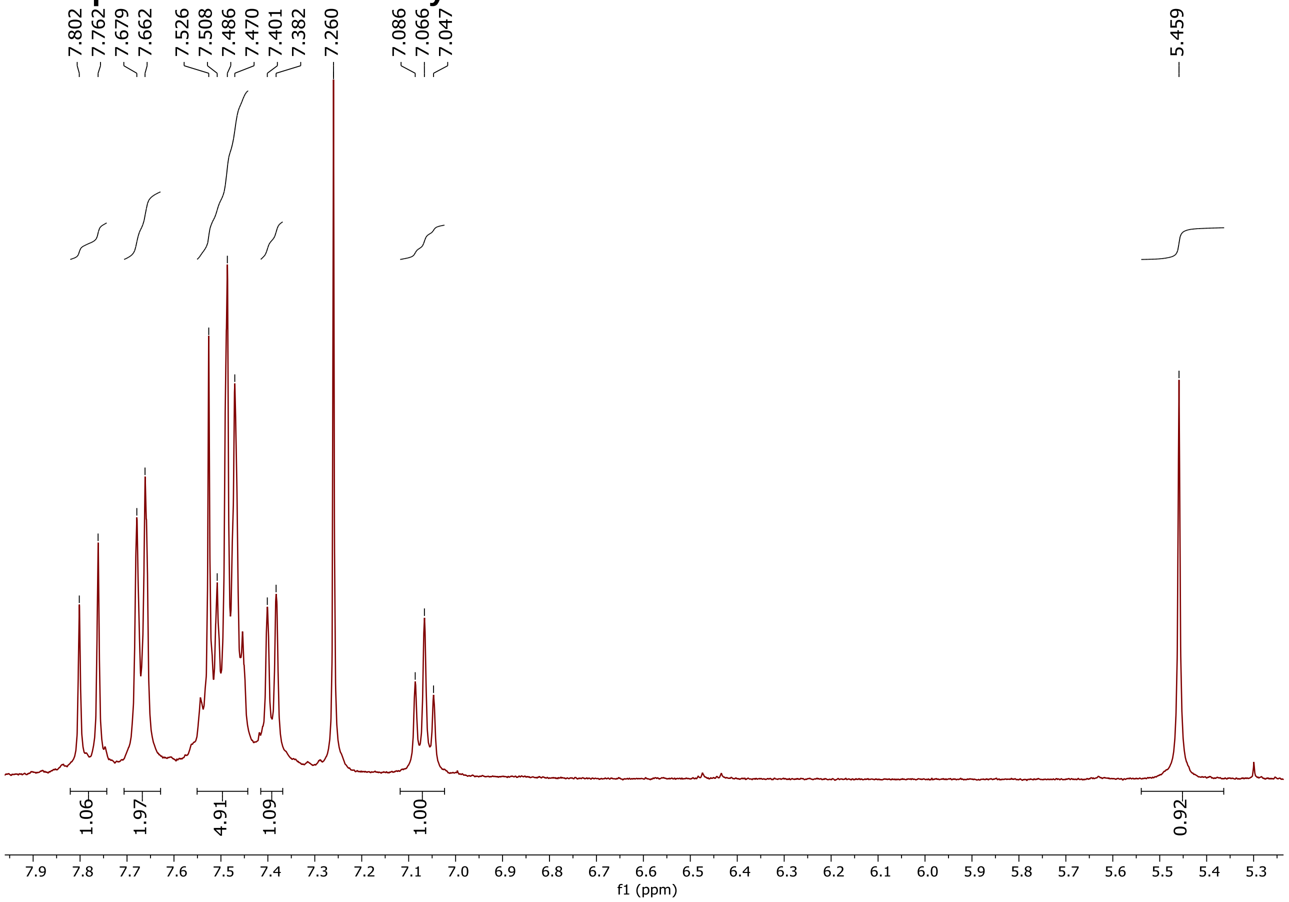

^{1}H NMR ($CDCl_3$, 300.13 MHz) of **4e**

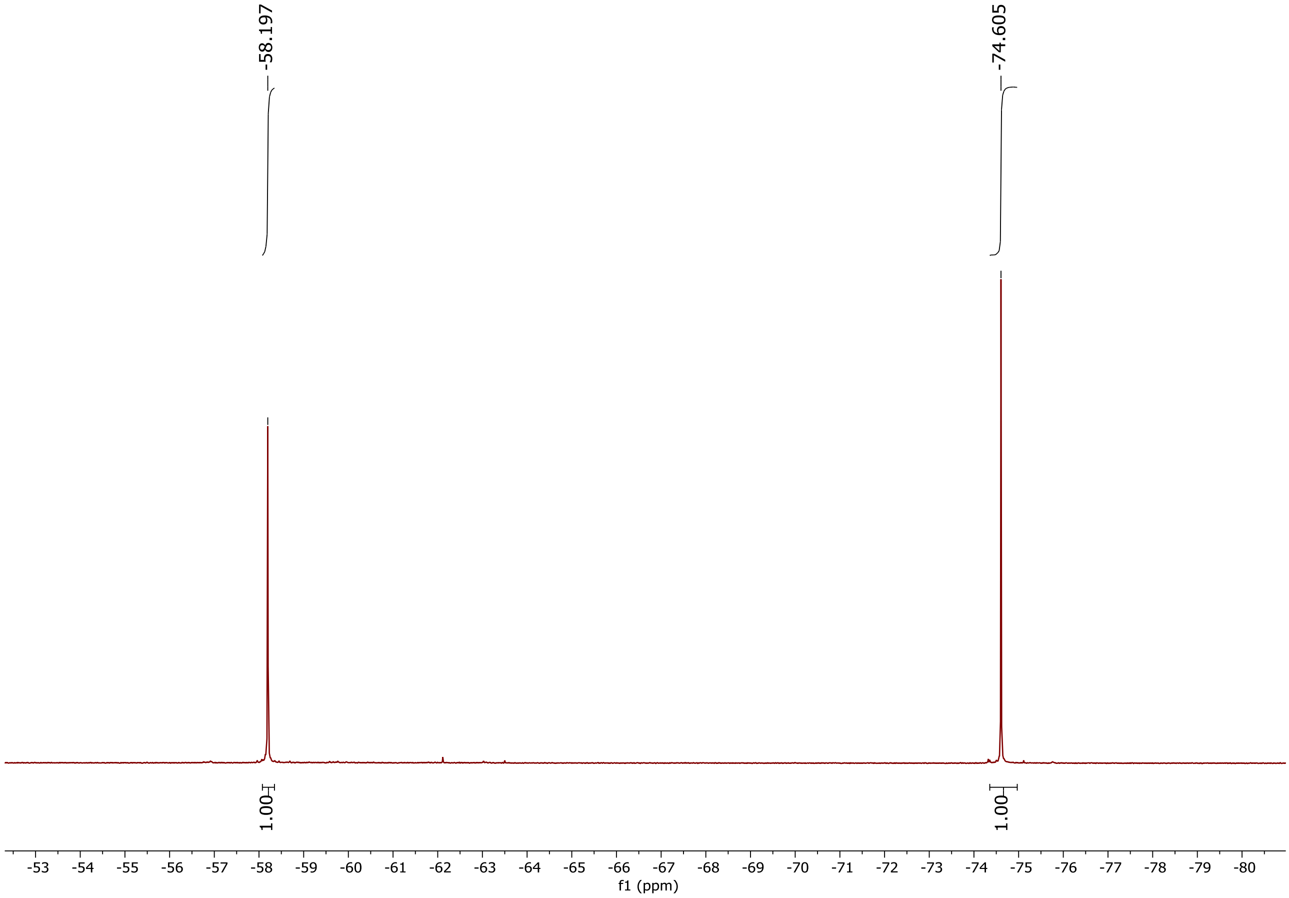

^{19}F NMR spectrum ($CDCl_3$, 282.40 MHz) of **4e**

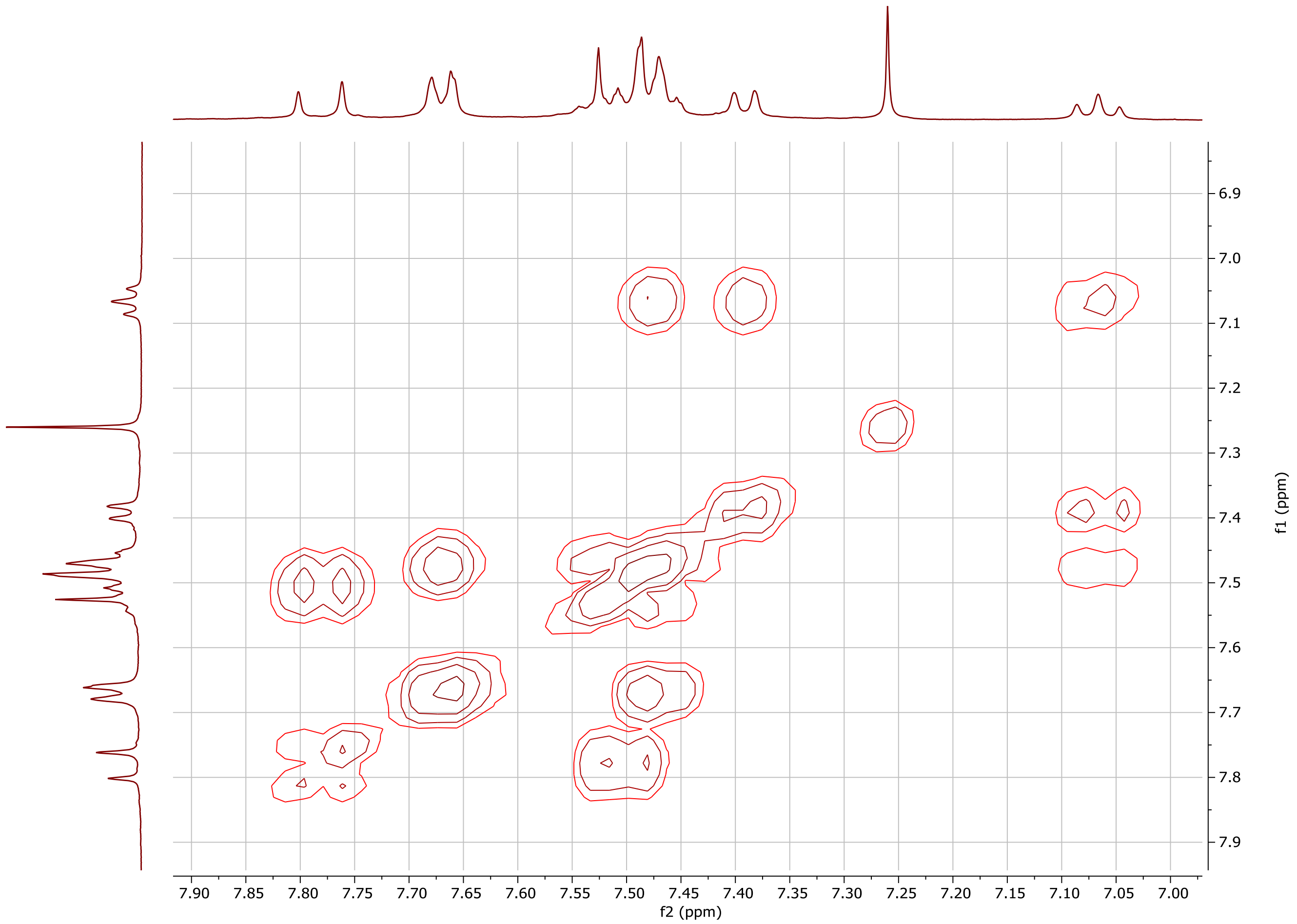

^1H-^1H COSY NMR spectrum of **4e**

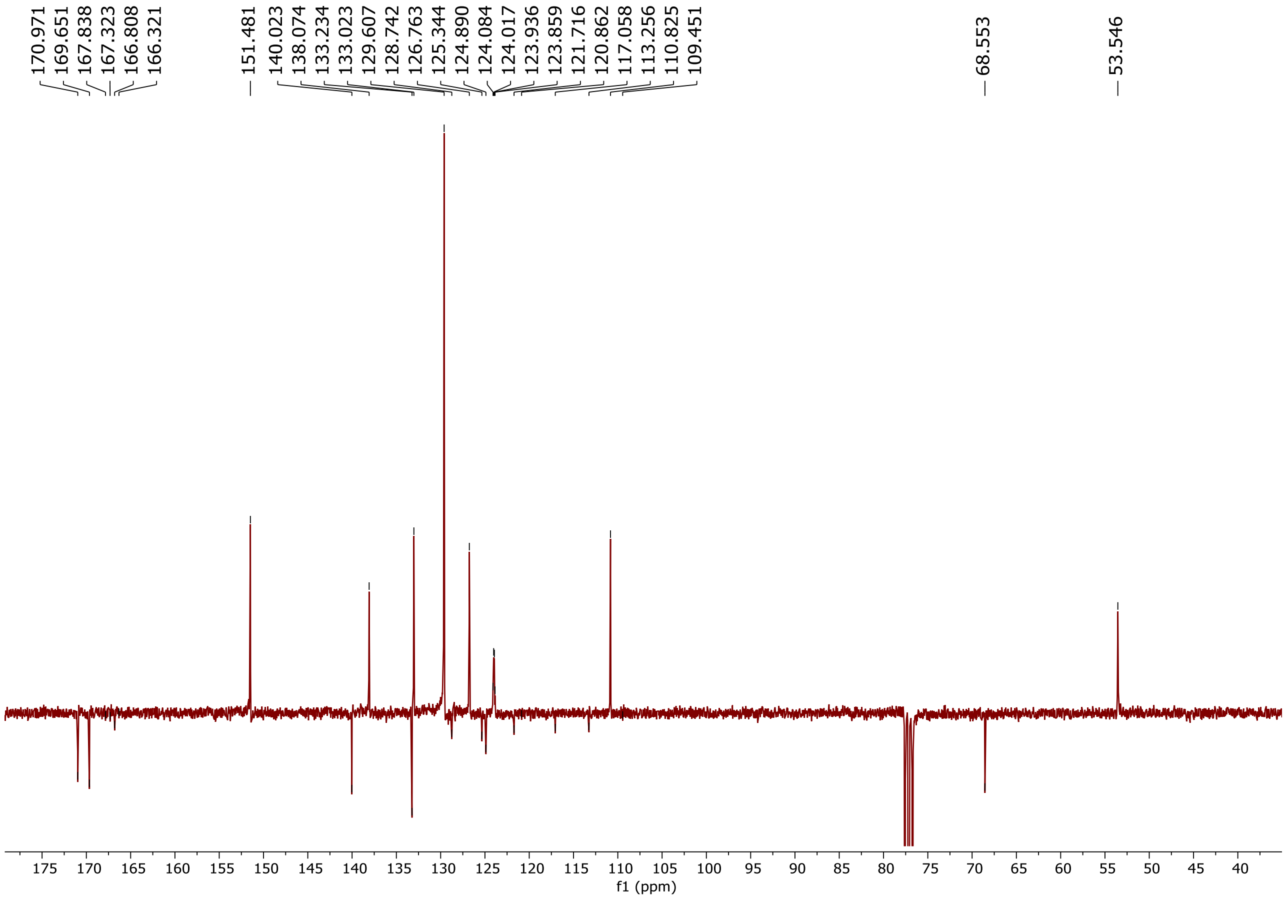

$^{13}C\{^1H\}$-(APT) NMR spectrum ($CDCl_3$, 75.47 MHz) of **4e**

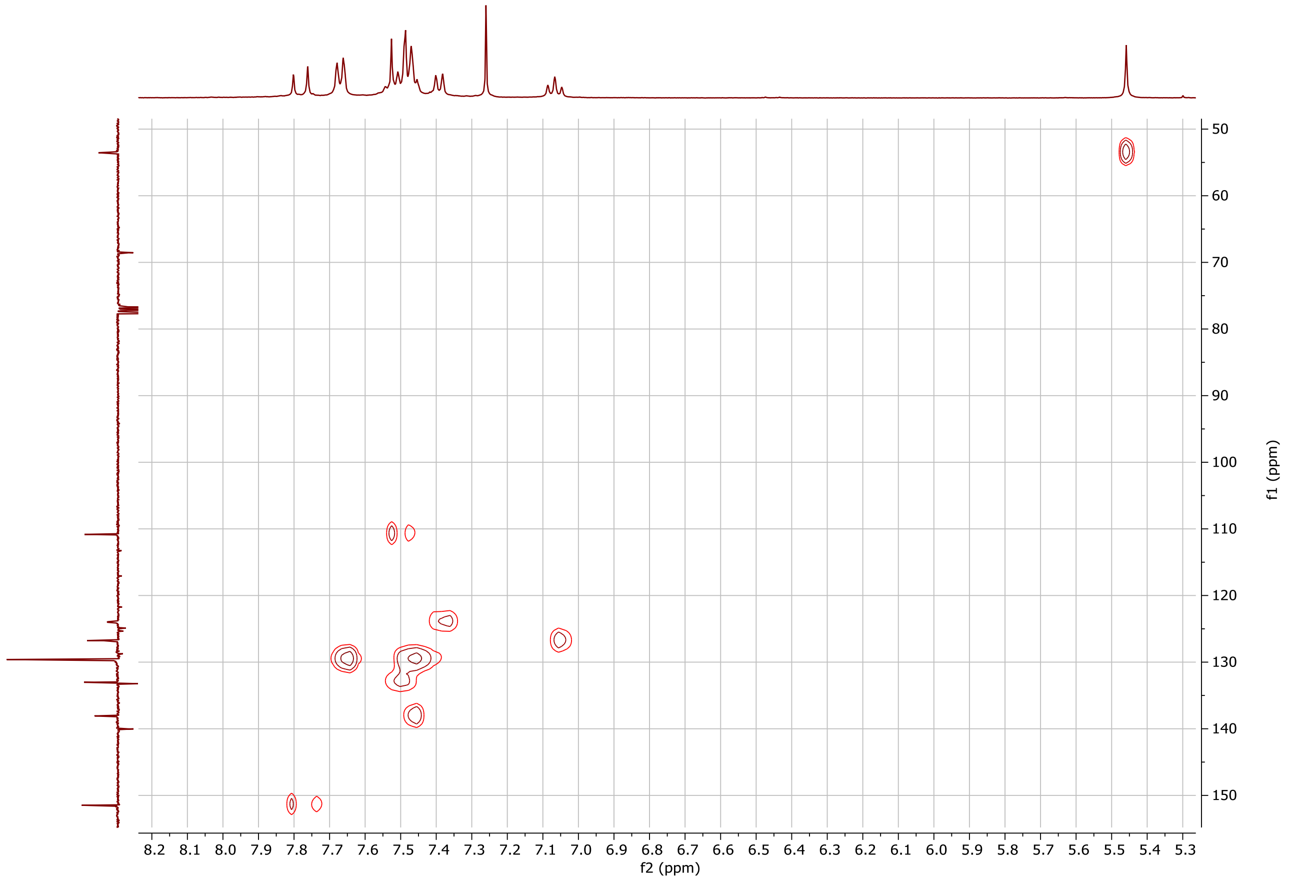

^{1}H-^{13}C HSQC NMR spectrum of **4e**

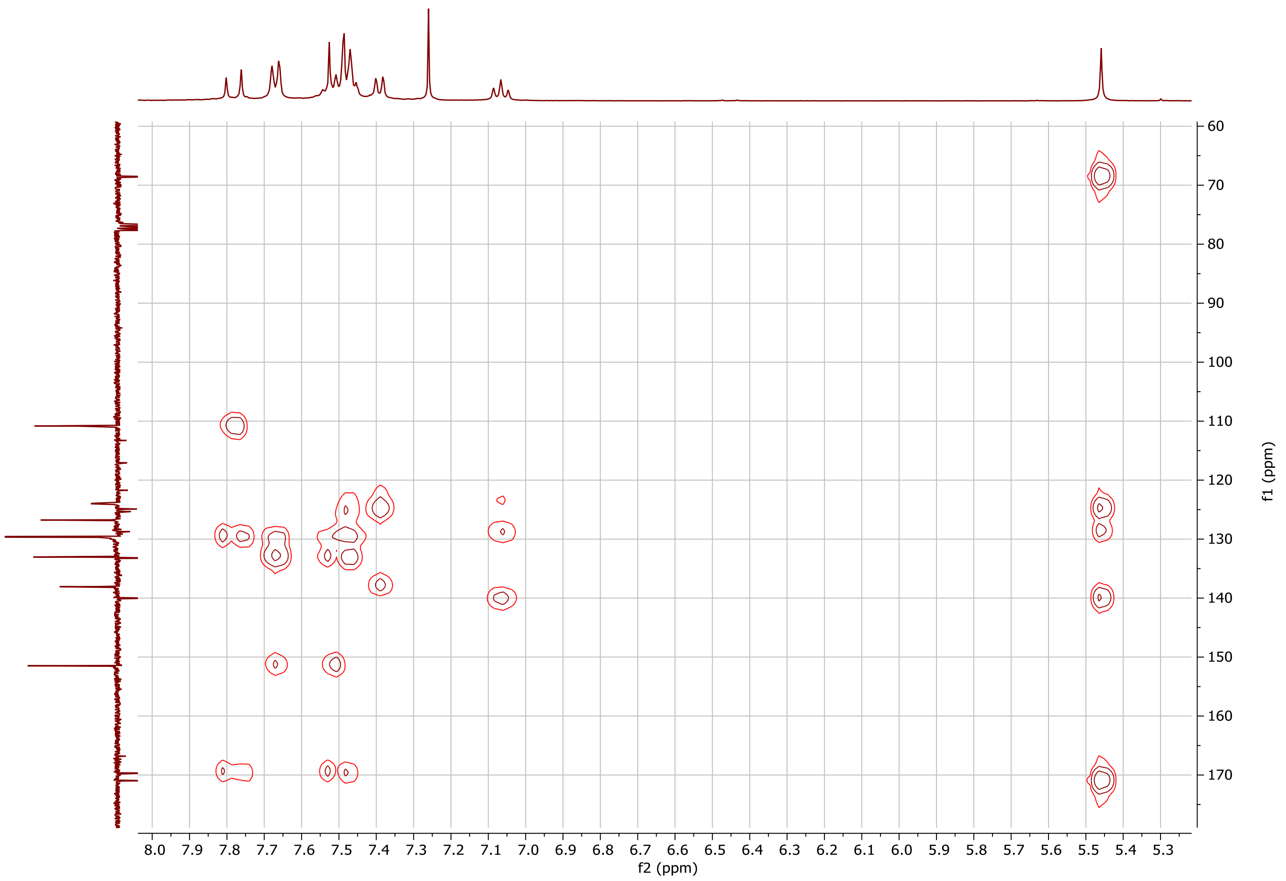

^{1}H-^{13}C HMBC NMR spectrum of **4e**

Orthopalladated dinuclear cyclobutane 4f

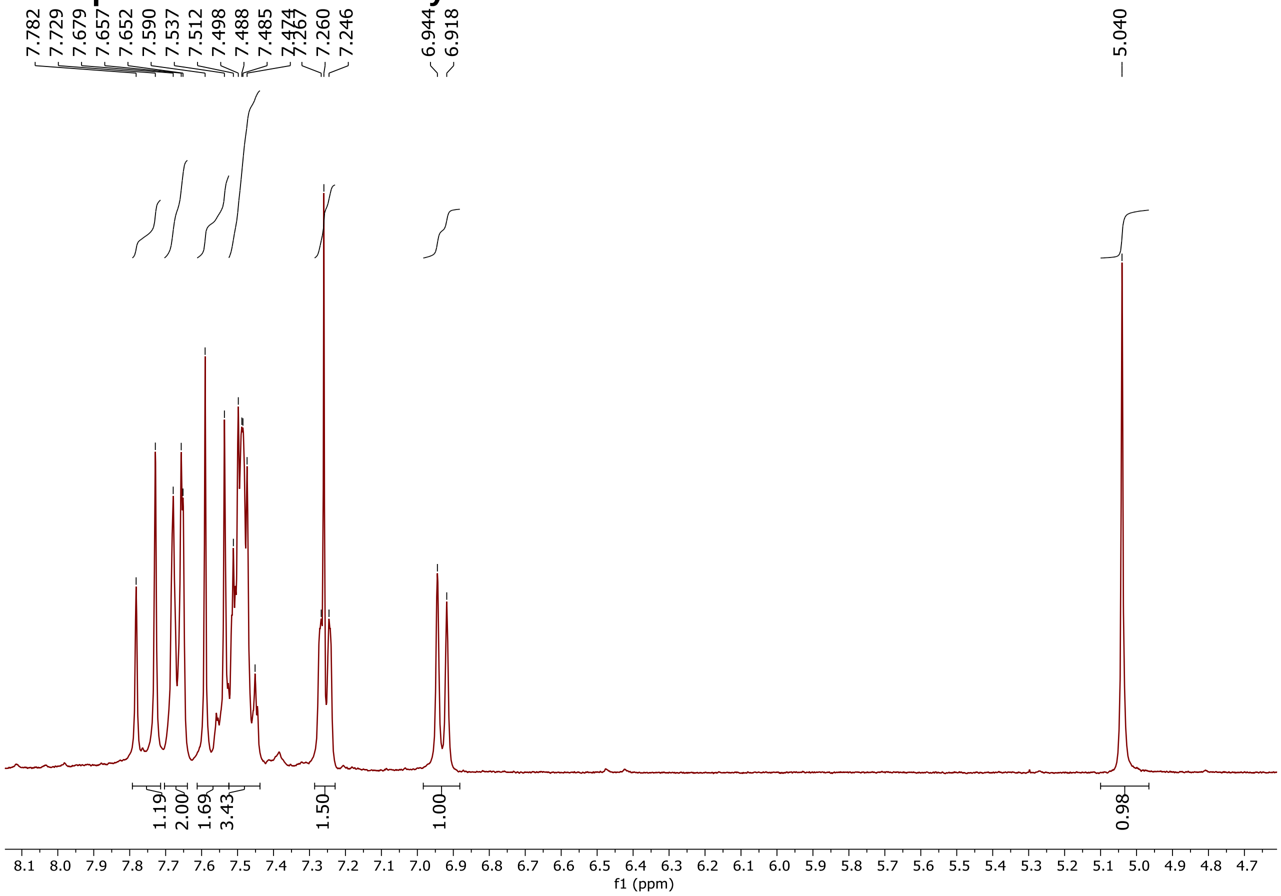

^{1}H NMR ($CDCl_3$, 300.13 MHz) of **4f**

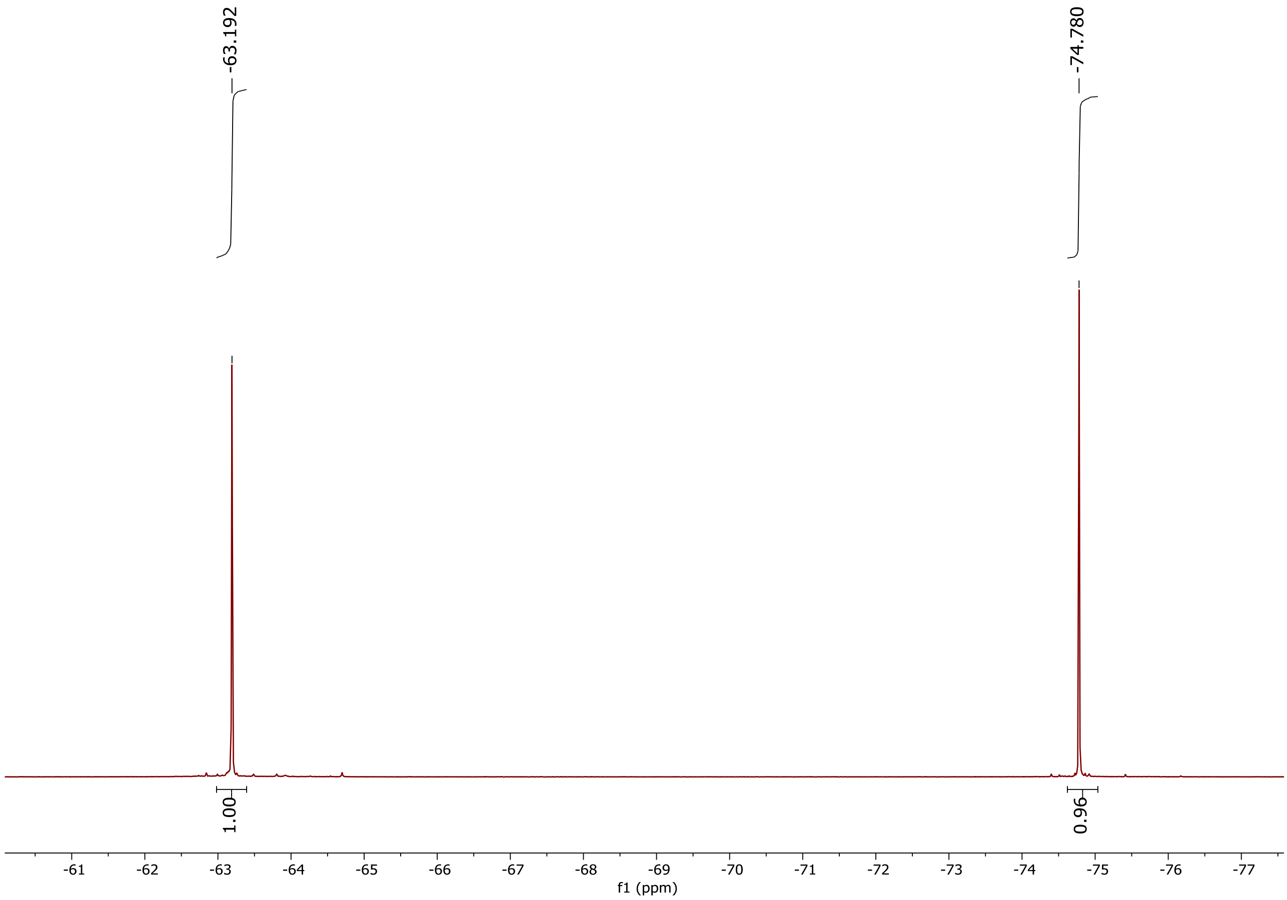

^{19}F NMR spectrum ($CDCl_3$, 282.40 MHz) of **4f**

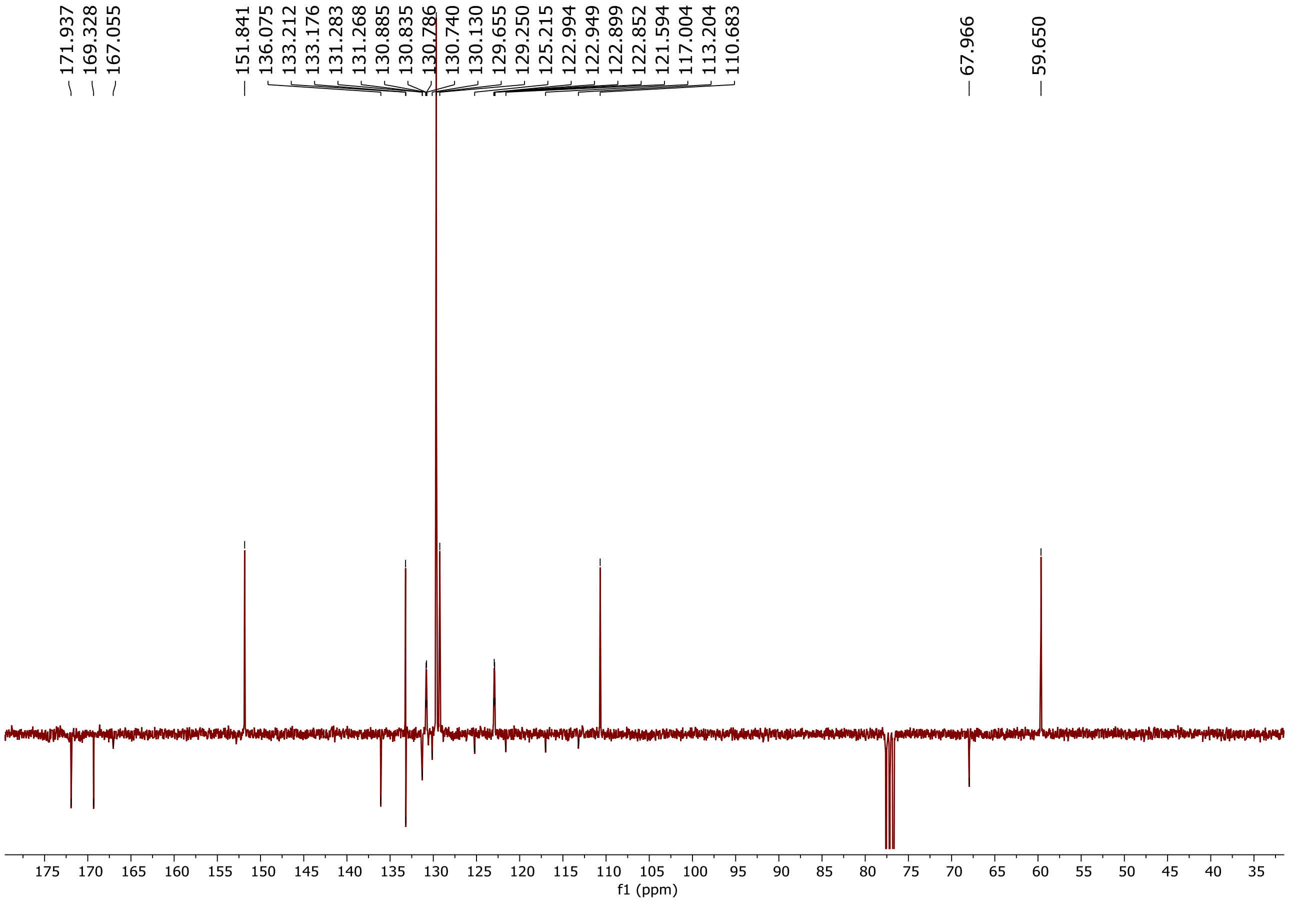

$^{13}C\{^{1}H\}$-(APT) NMR spectrum ($CDCl_3$, 75.47 MHz) of **4f**

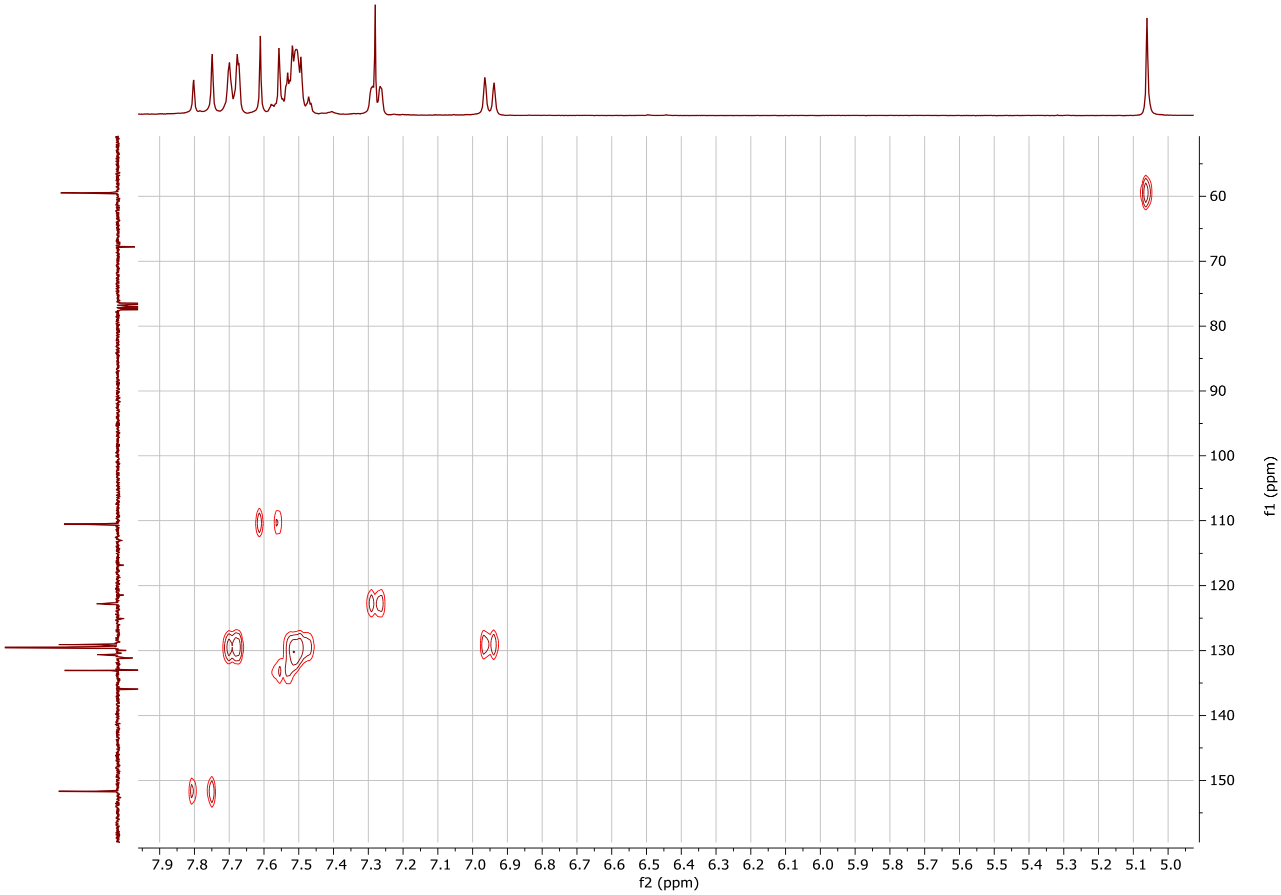

^{1}H-^{13}C HSQC NMR spectrum of **4f**

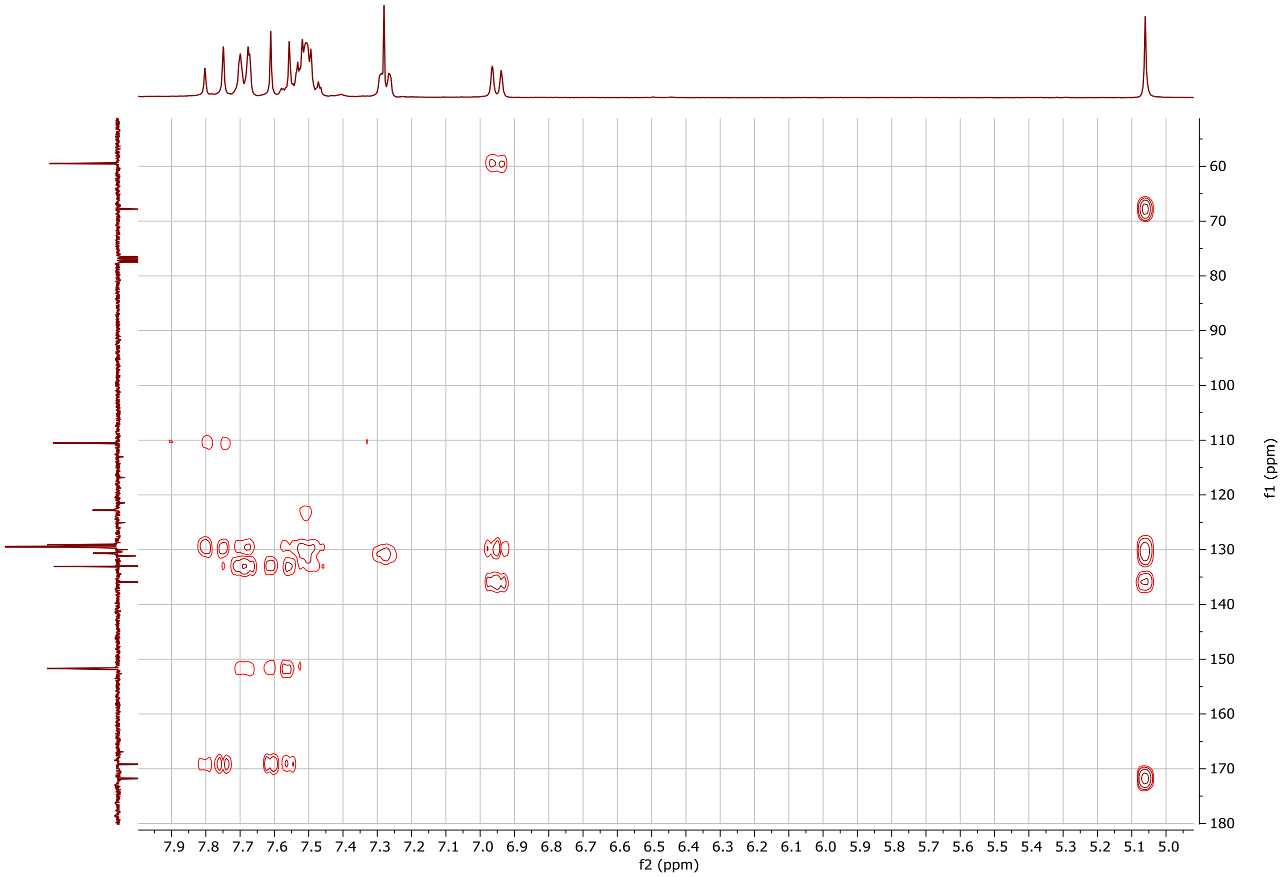

^{1}H-^{13}C HMBC NMR spectrum of **4f**

Orthopalladated dinuclear cyclobutane 4h

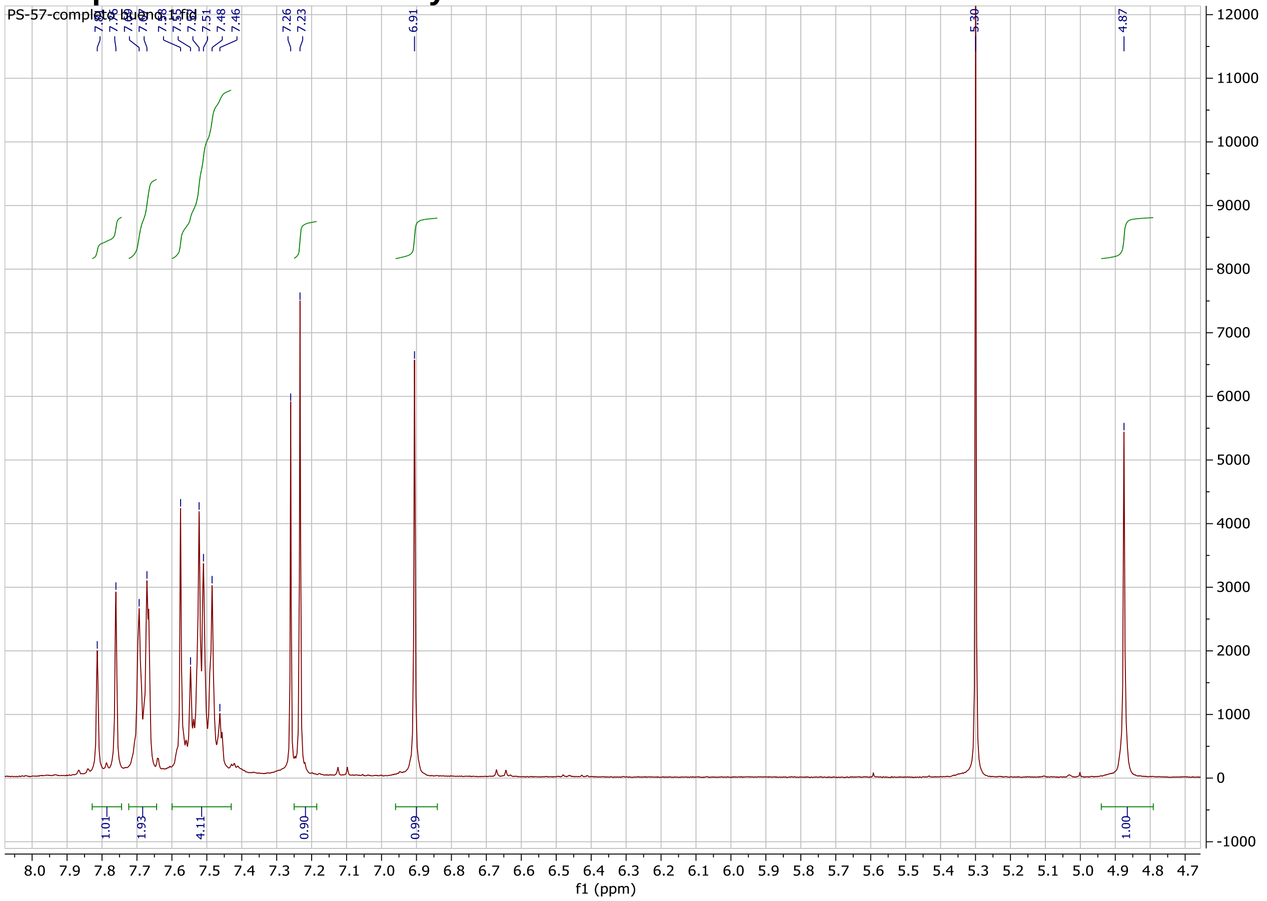

^{1}H NMR ($CDCl_3$, 300.13 MHz) of **4h**

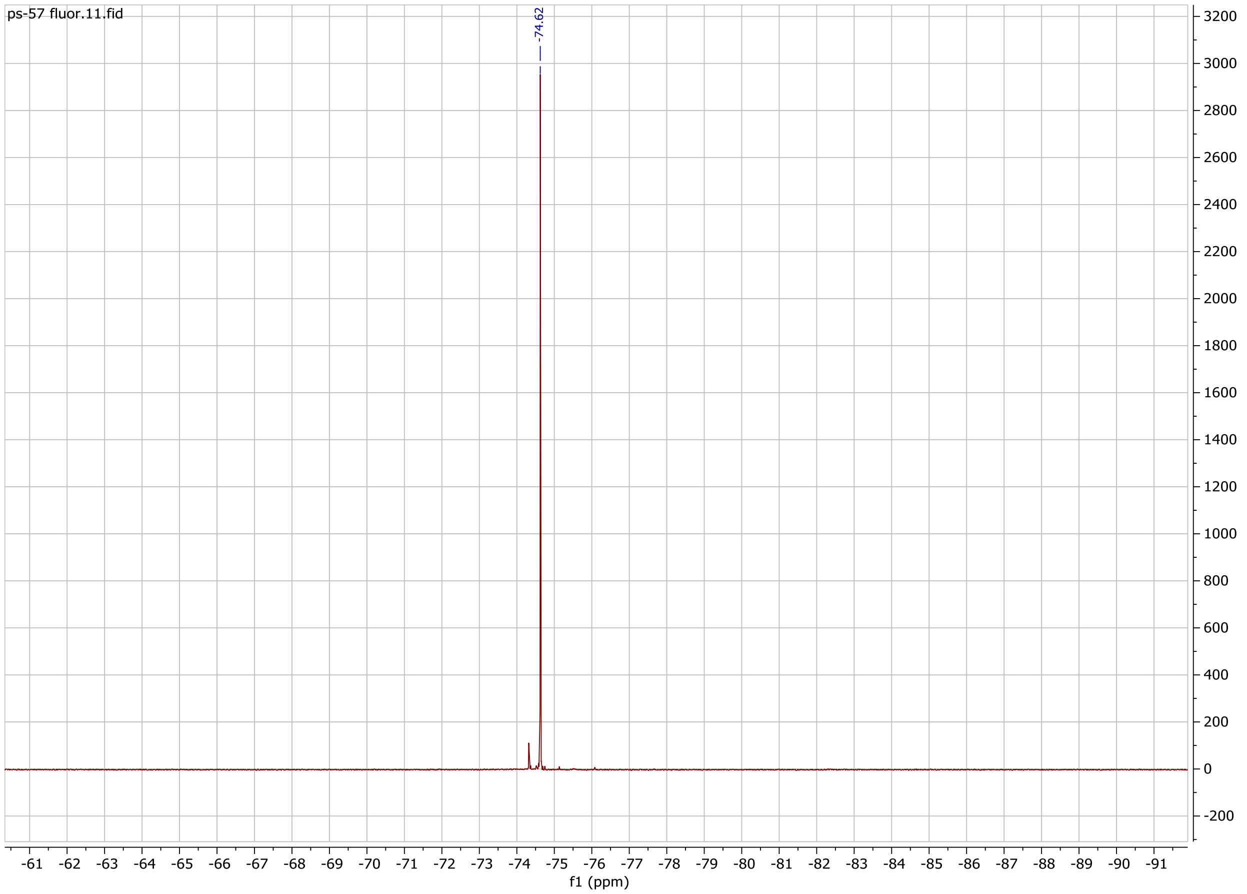

^{19}F NMR spectrum ($CDCl_3$, 282.40 MHz) of **4h**

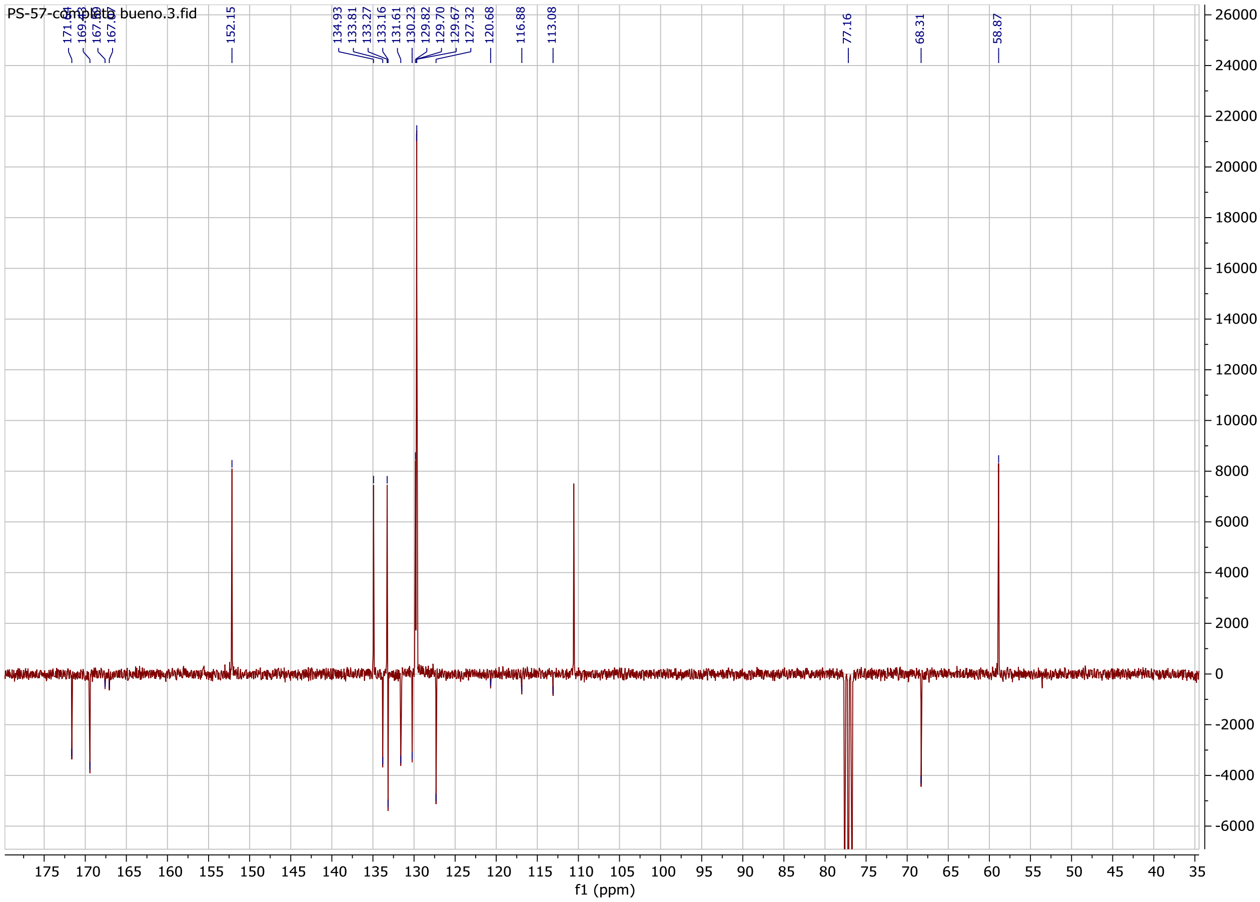

$^{13}C\{^1H\}$-(APT) NMR spectrum ($CDCl_3$, 75.47 MHz) of **4h**

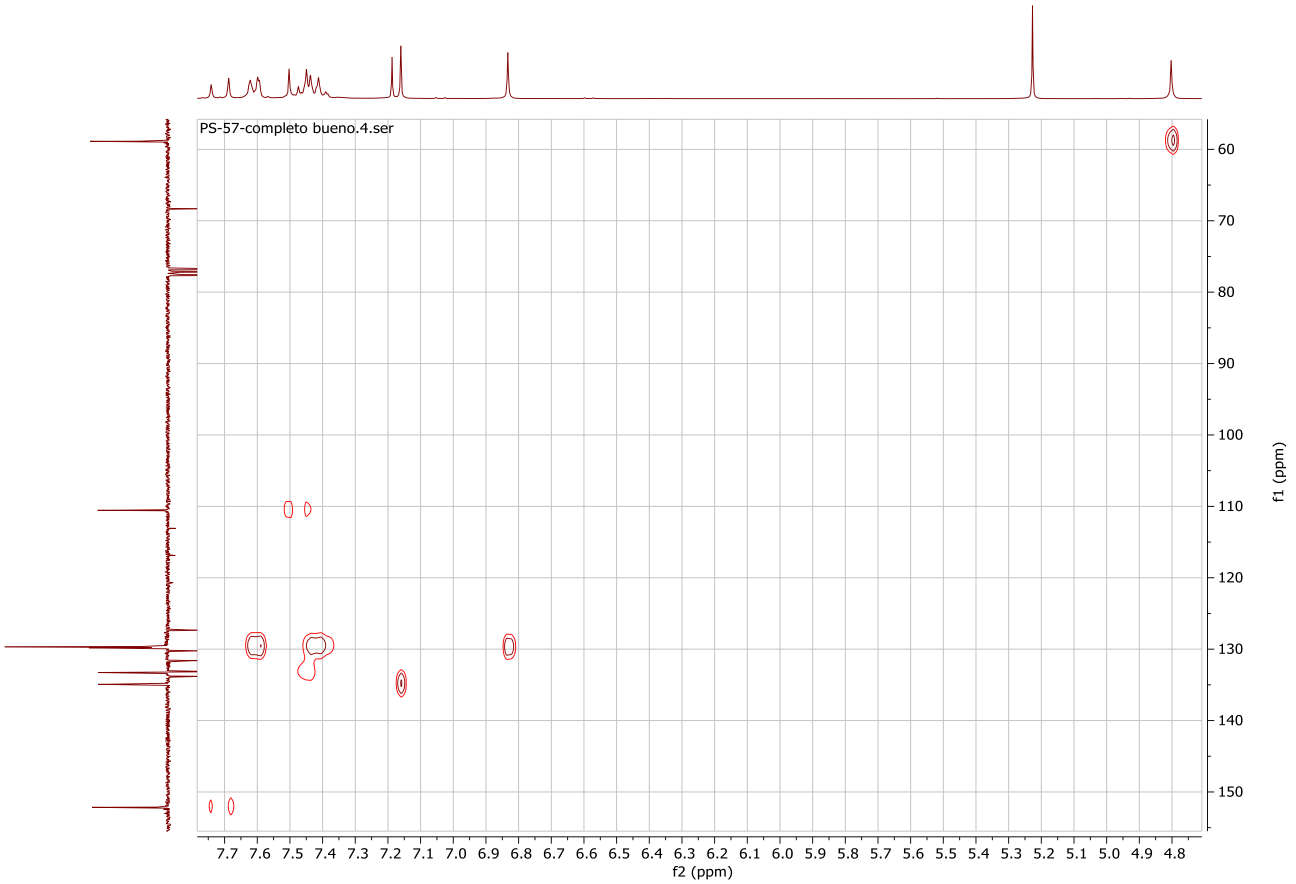

^{1}H-^{13}C HSQC NMR spectrum of **4h**

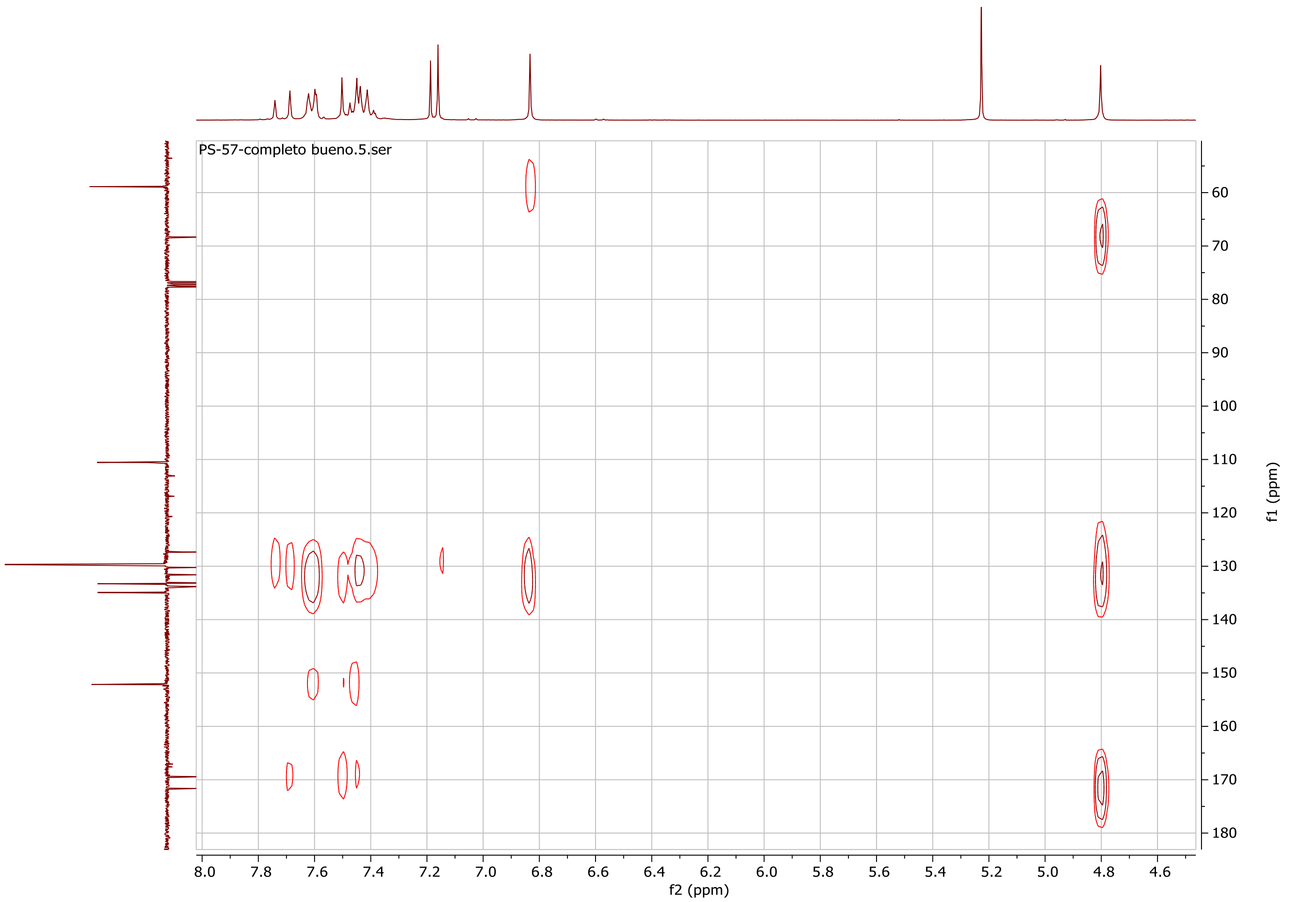

^{1}H-^{13}C HMBC NMR spectrum of **4h**

Orthopalladated dinuclear cyclobutane 4i

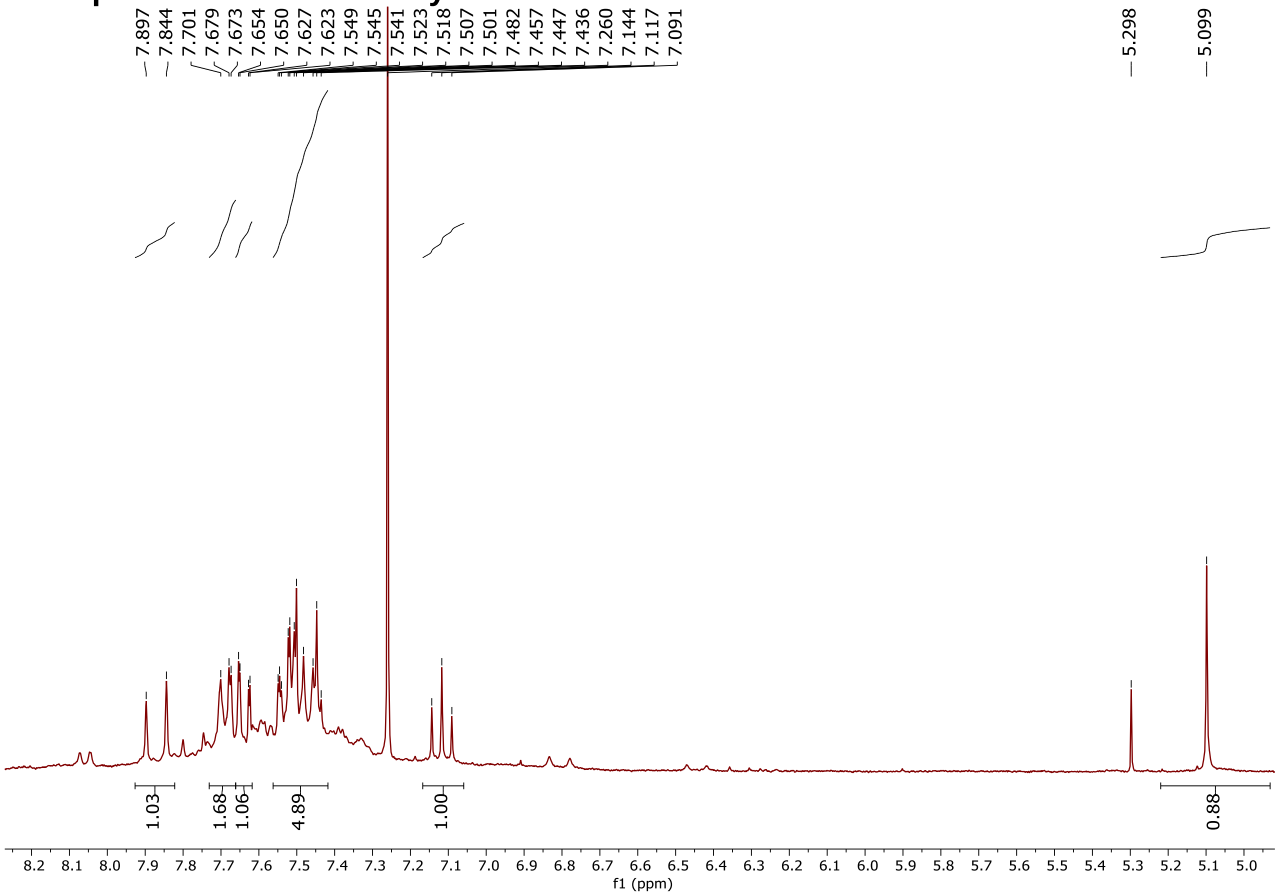

^{1}H NMR ($CDCl_3$, 300.13 MHz) of **4i**

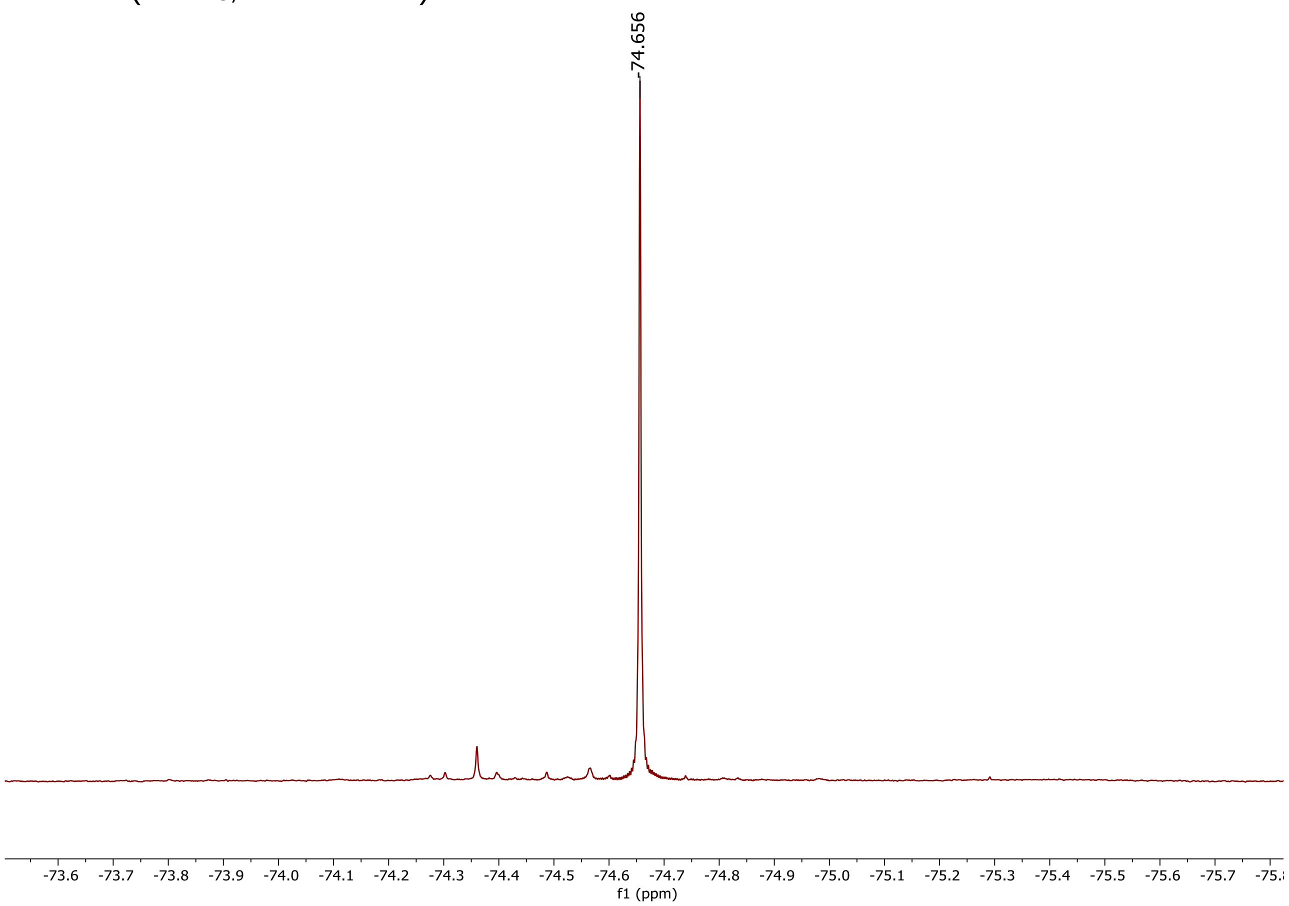

^{19}F NMR spectrum ($CDCl_3$, 282.40 MHz) of **4i**

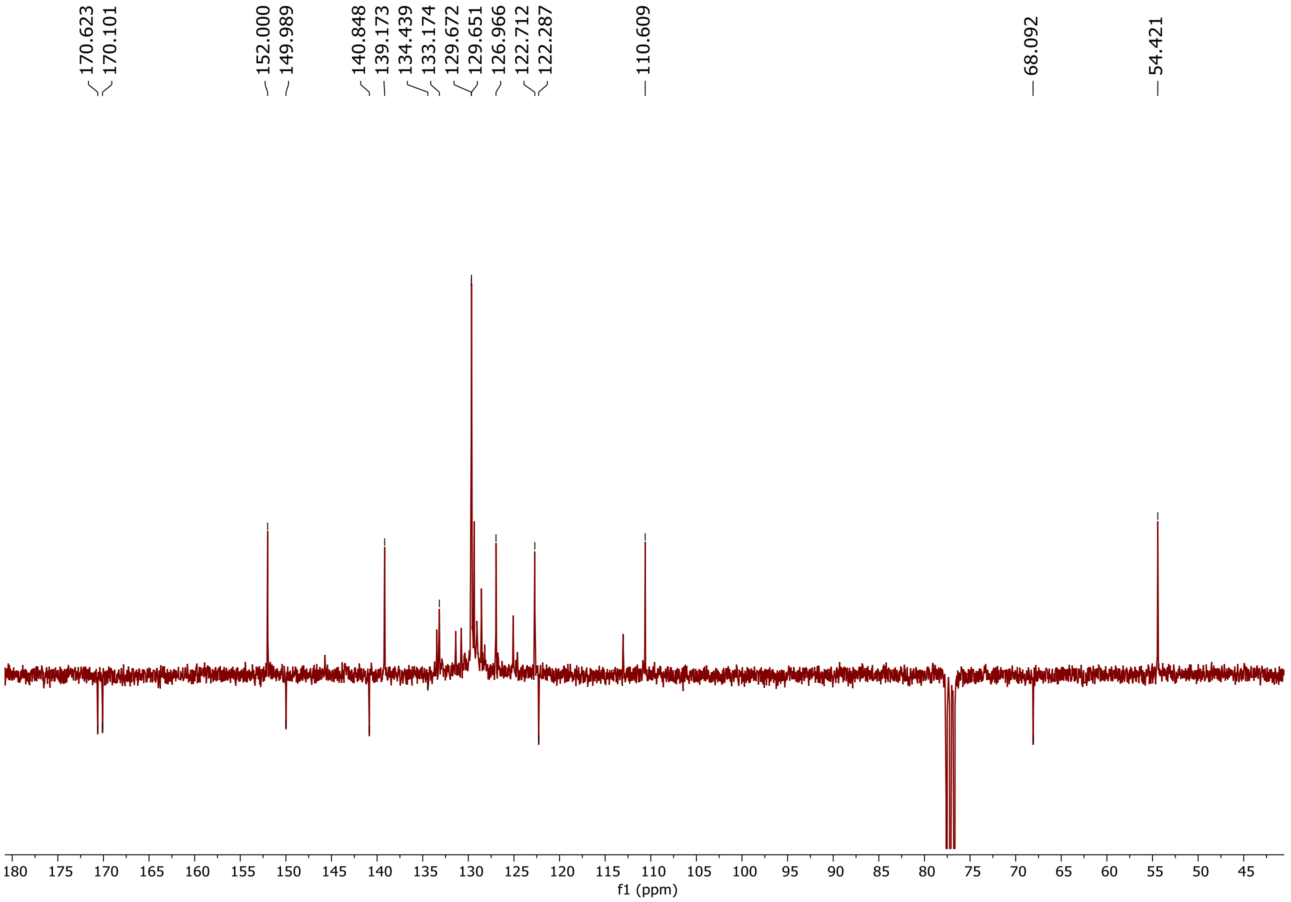

$^{13}C\{^{1}H\}$-(APT) NMR spectrum ($CDCl_3$, 75.47 MHz) of **4i**

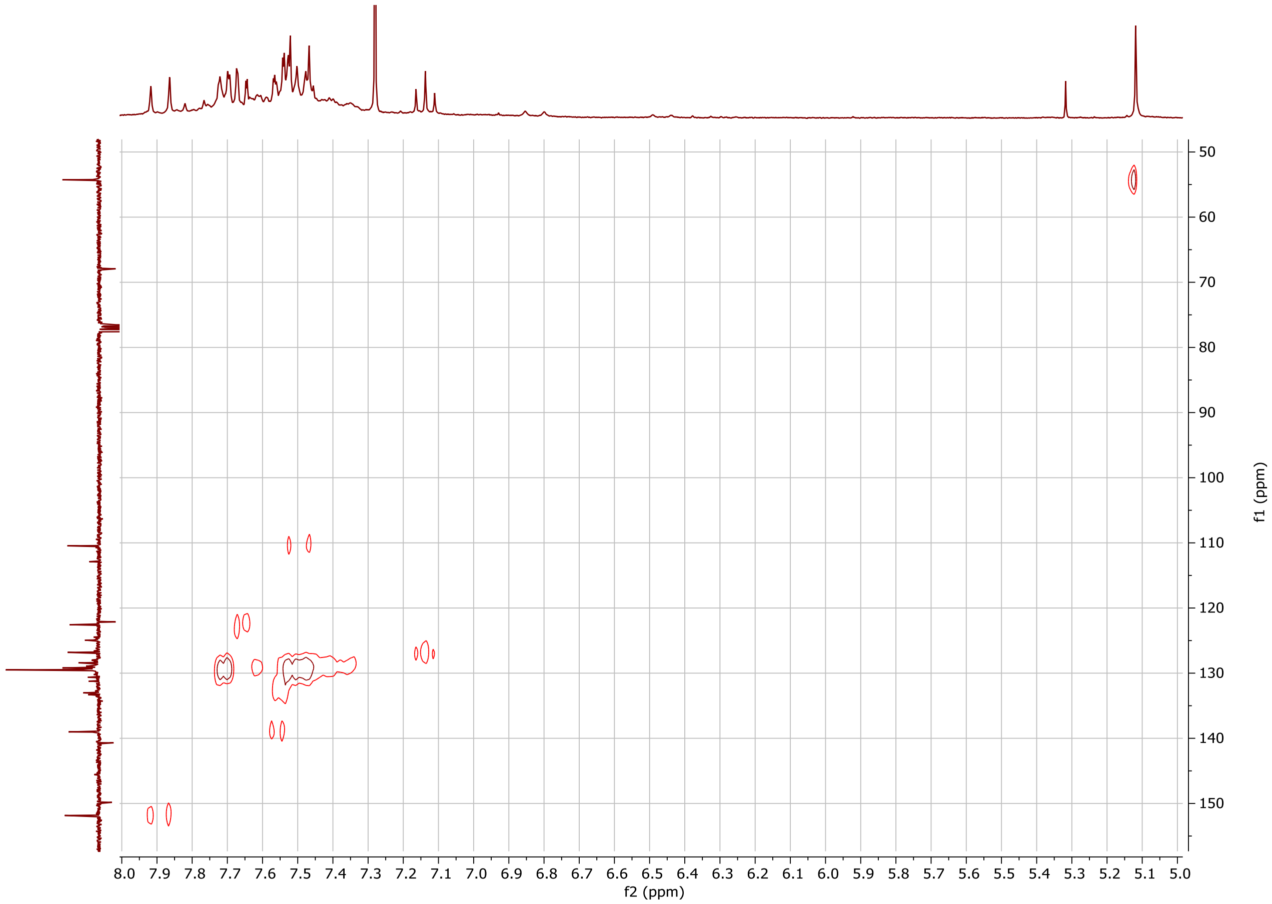

^{1}H-^{13}C HSQC NMR spectrum of **4i**

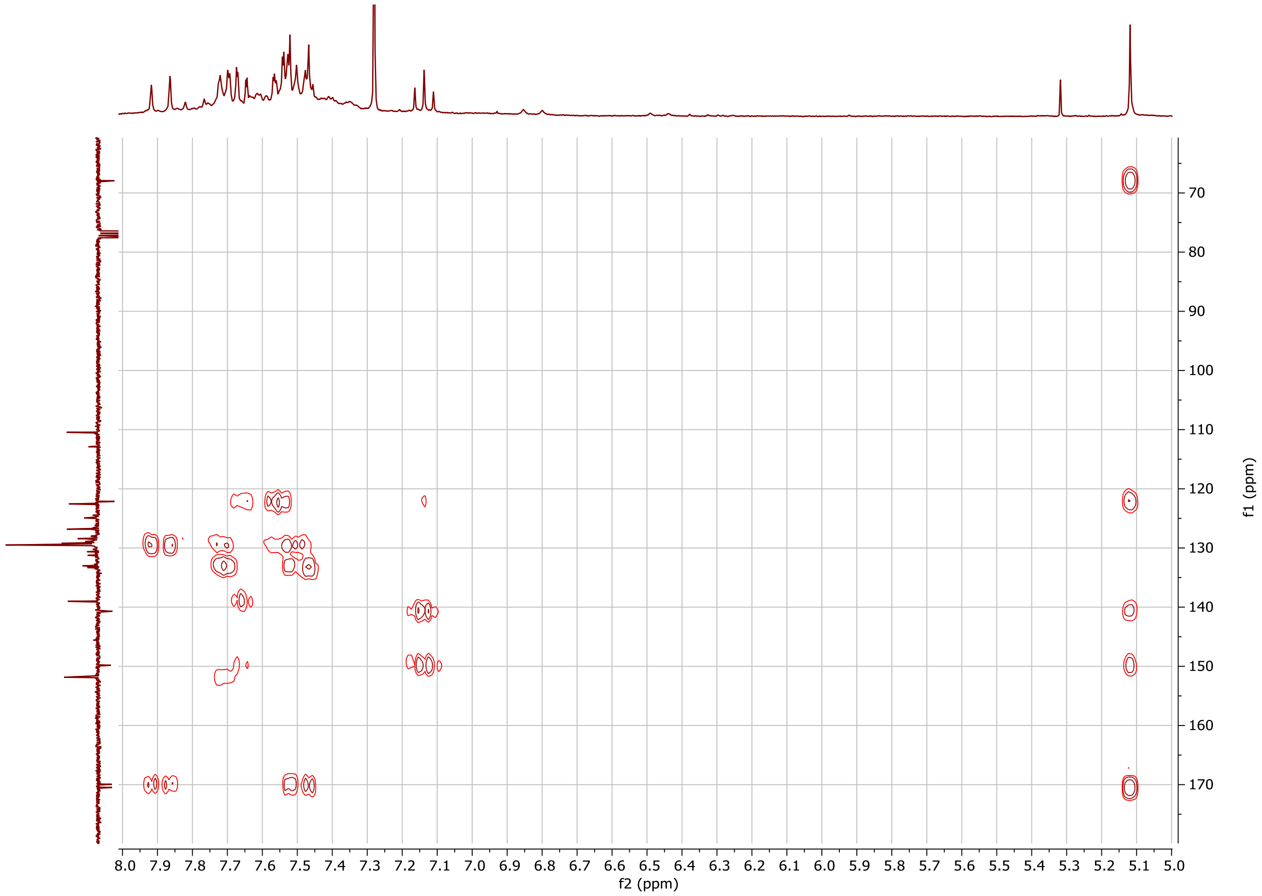

^{1}H-^{13}C HMBC NMR spectrum of **4i**

Orthopalladated dinuclear cyclobutane 4j

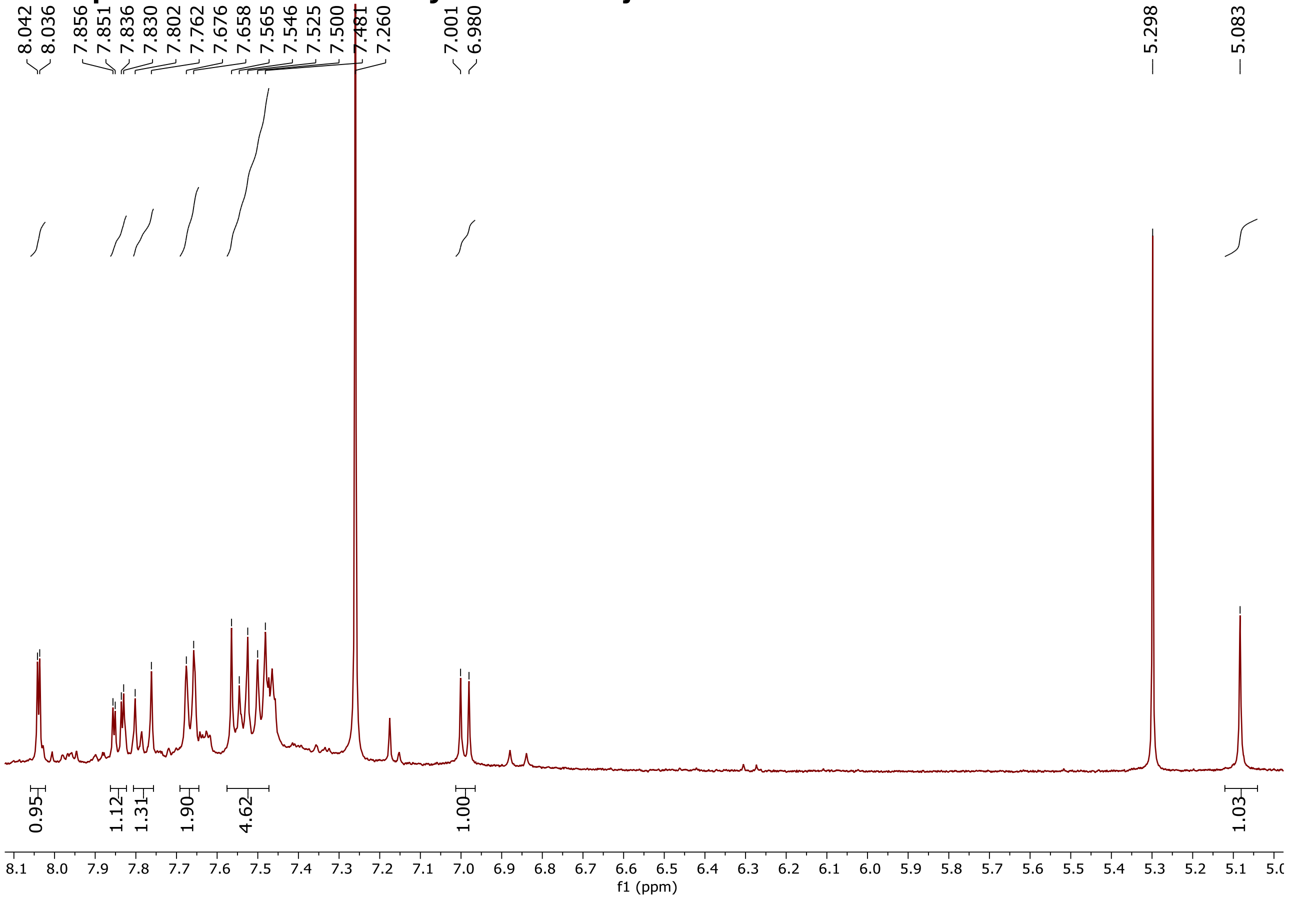

^{1}H NMR ($CDCl_3$, 300.13 MHz) of **4j**

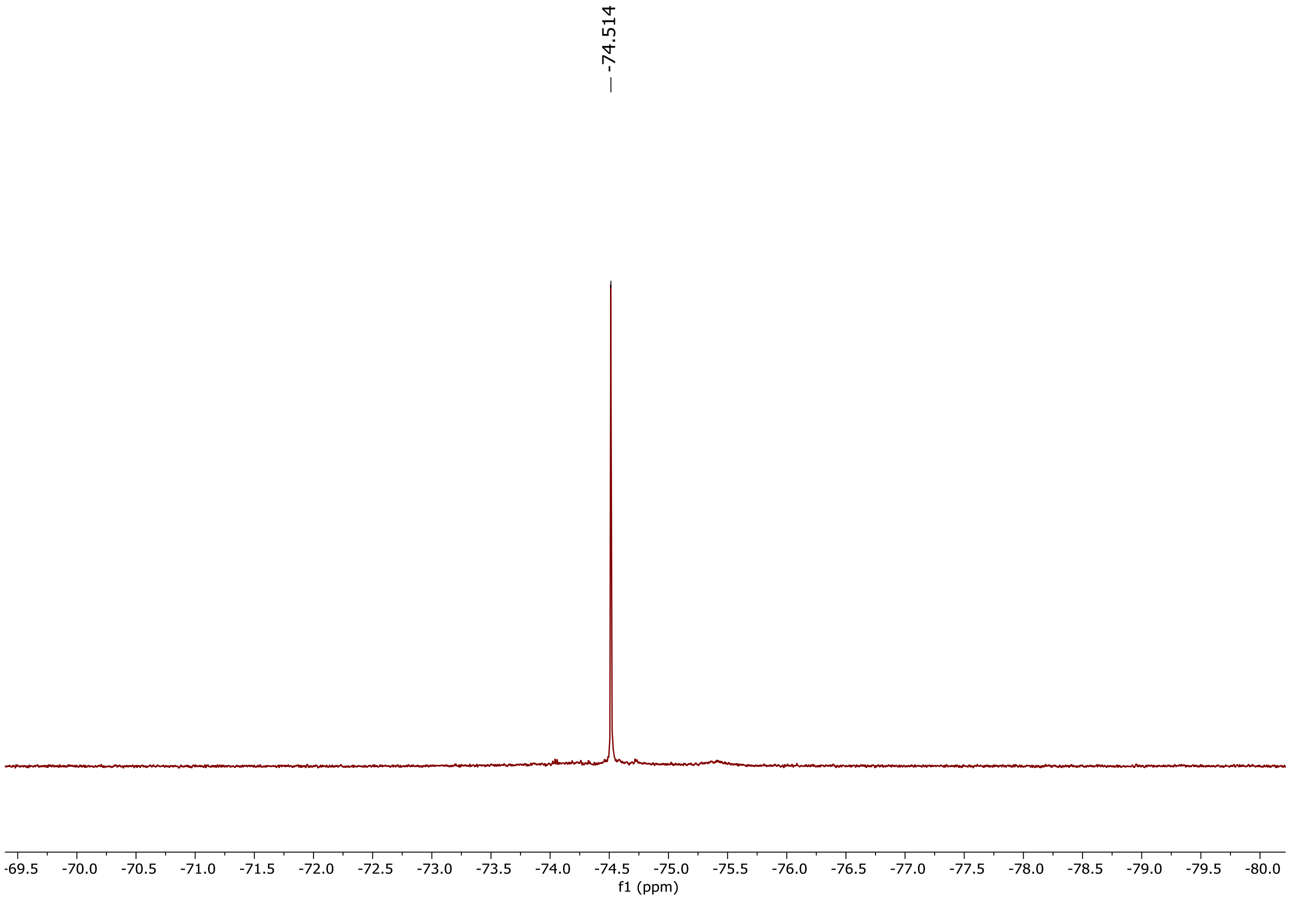

^{19}F NMR spectrum ($CDCl_3$, 282.40 MHz) of **4j**

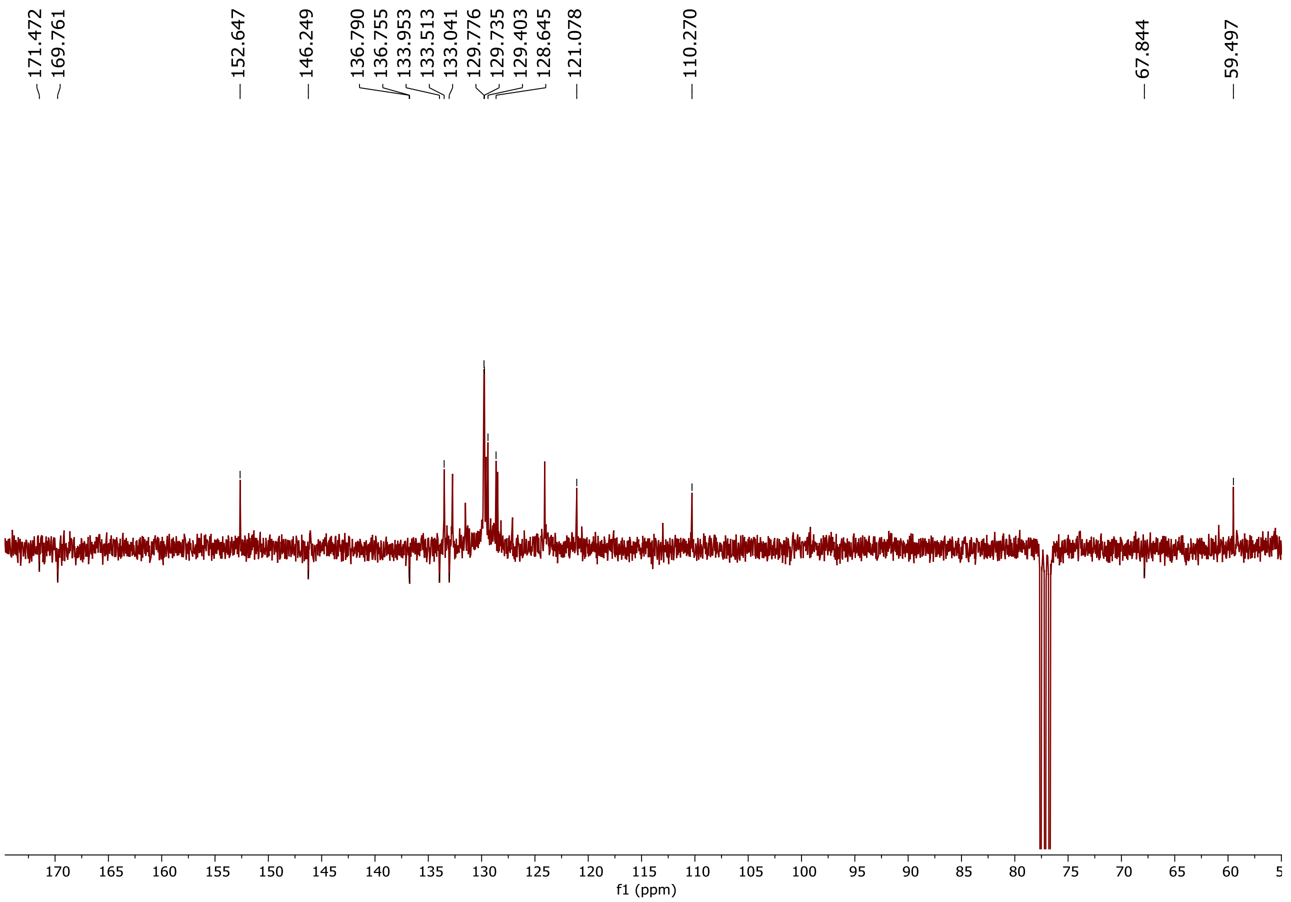

$^{13}C\{^1H\}$-(APT) NMR spectrum ($CDCl_3$, 75.47 MHz) of **4j**

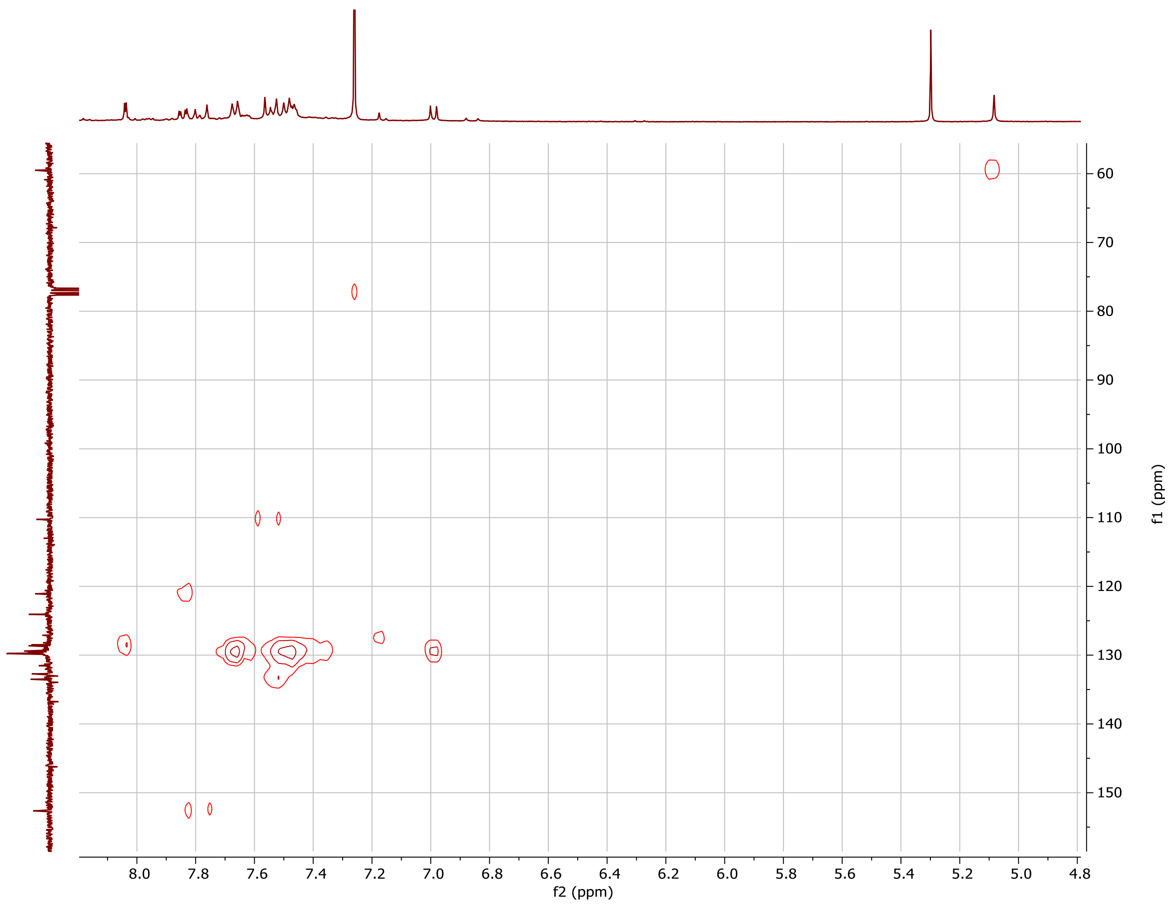

^{1}H-^{13}C HSQC NMR spectrum of **4j**

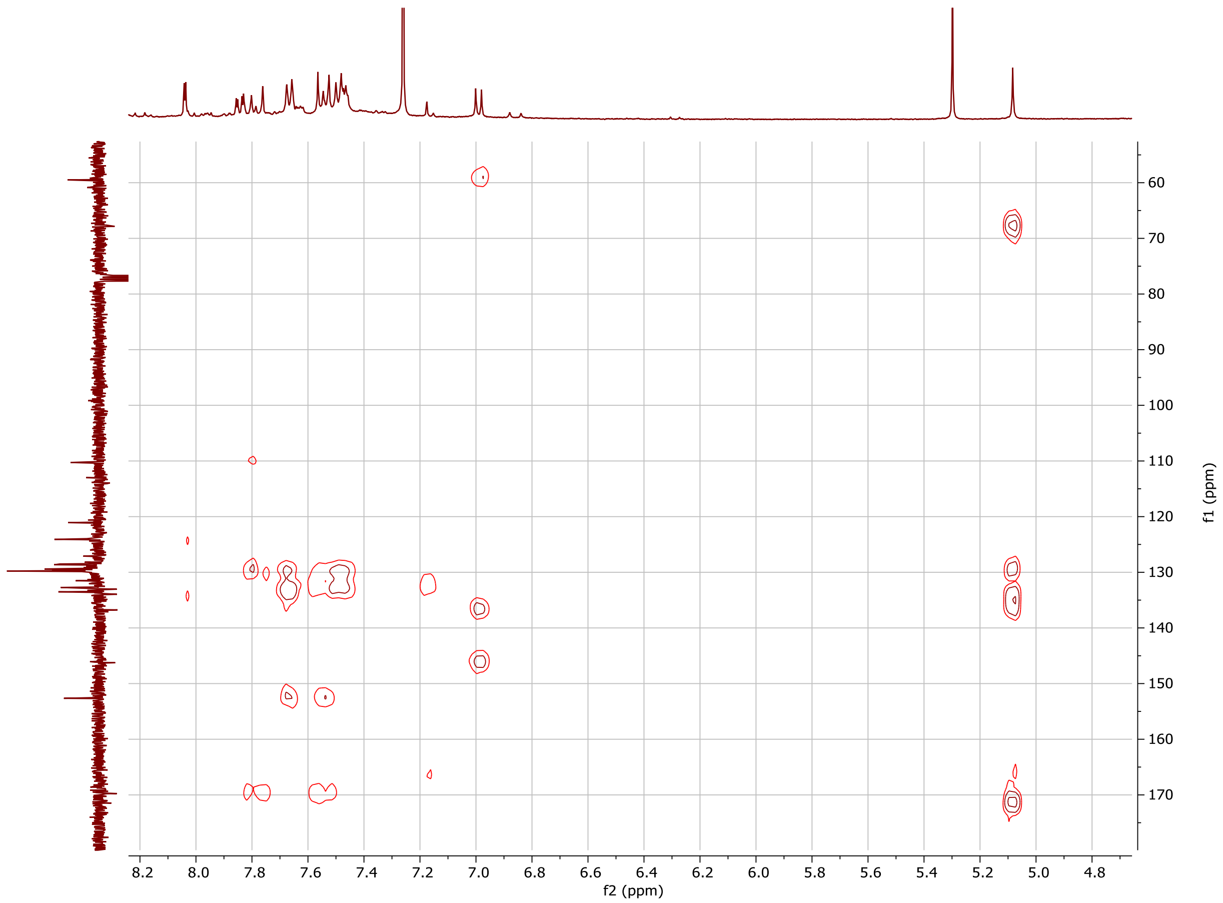

^{1}H-^{13}C HMBC NMR spectrum of **4j**

4. NMR spectra of *ortho*-alkoxycarbonylated diaminotruxillic cyclobutanes 5.

Diaminotruxillic cyclobutane 5b.

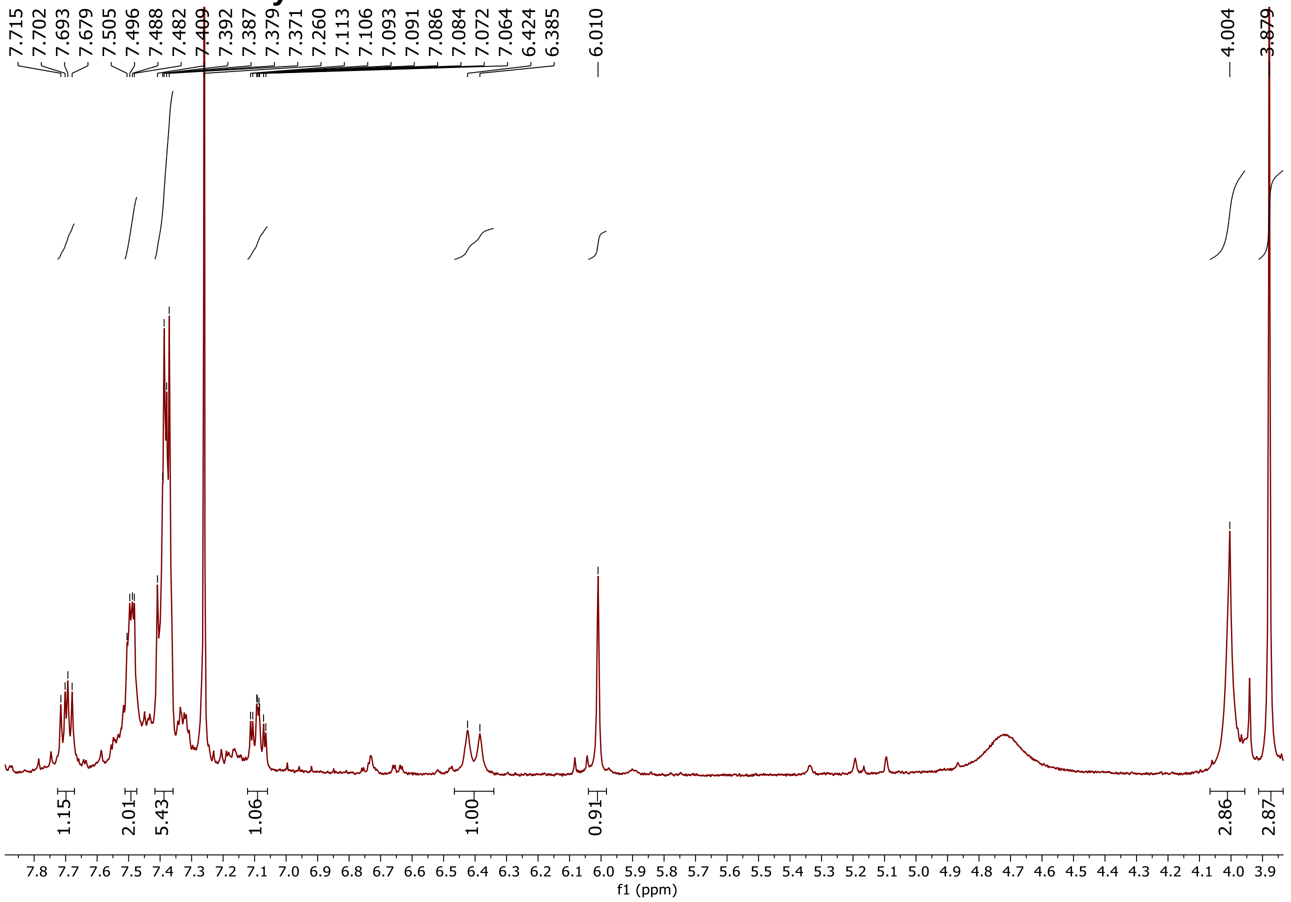

^{1}H NMR ($CDCl_3$, 400.13 MHz) of **5b**

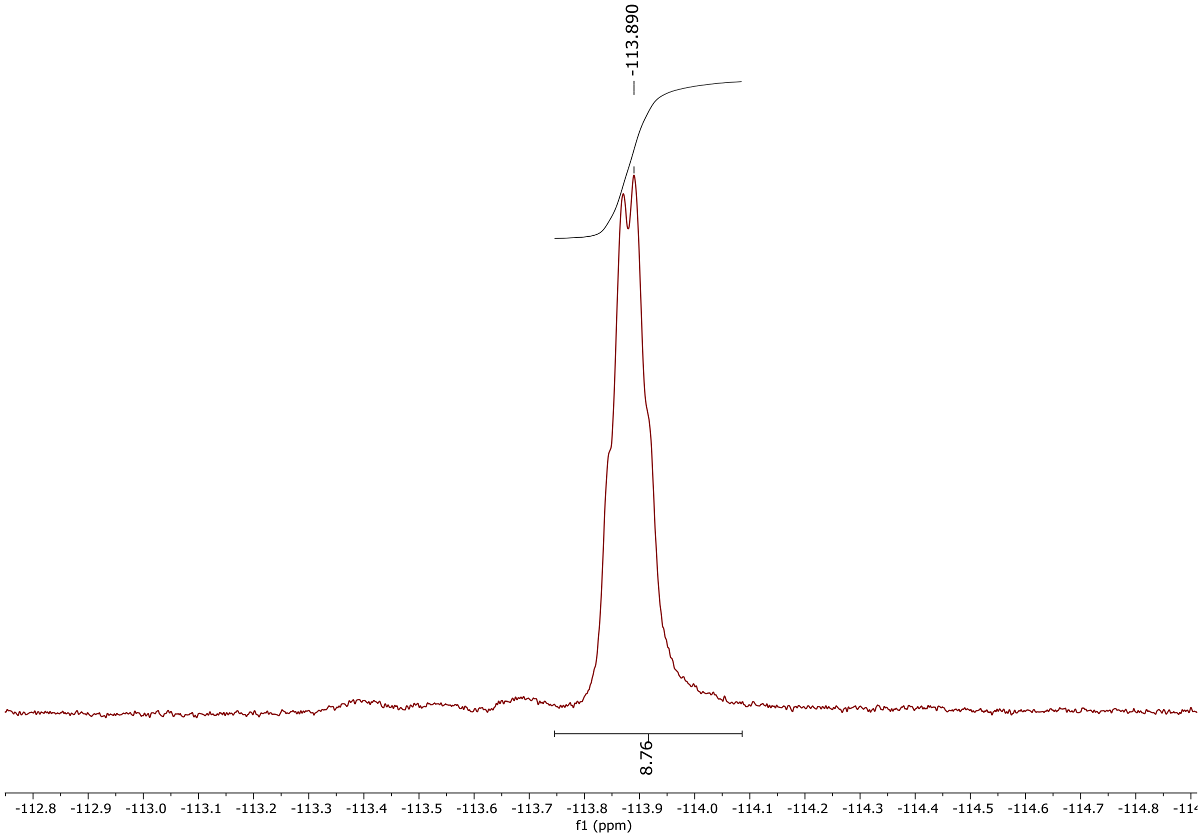

^{19}F NMR spectrum ($CDCl_3$, 282.40 MHz) of **5b**

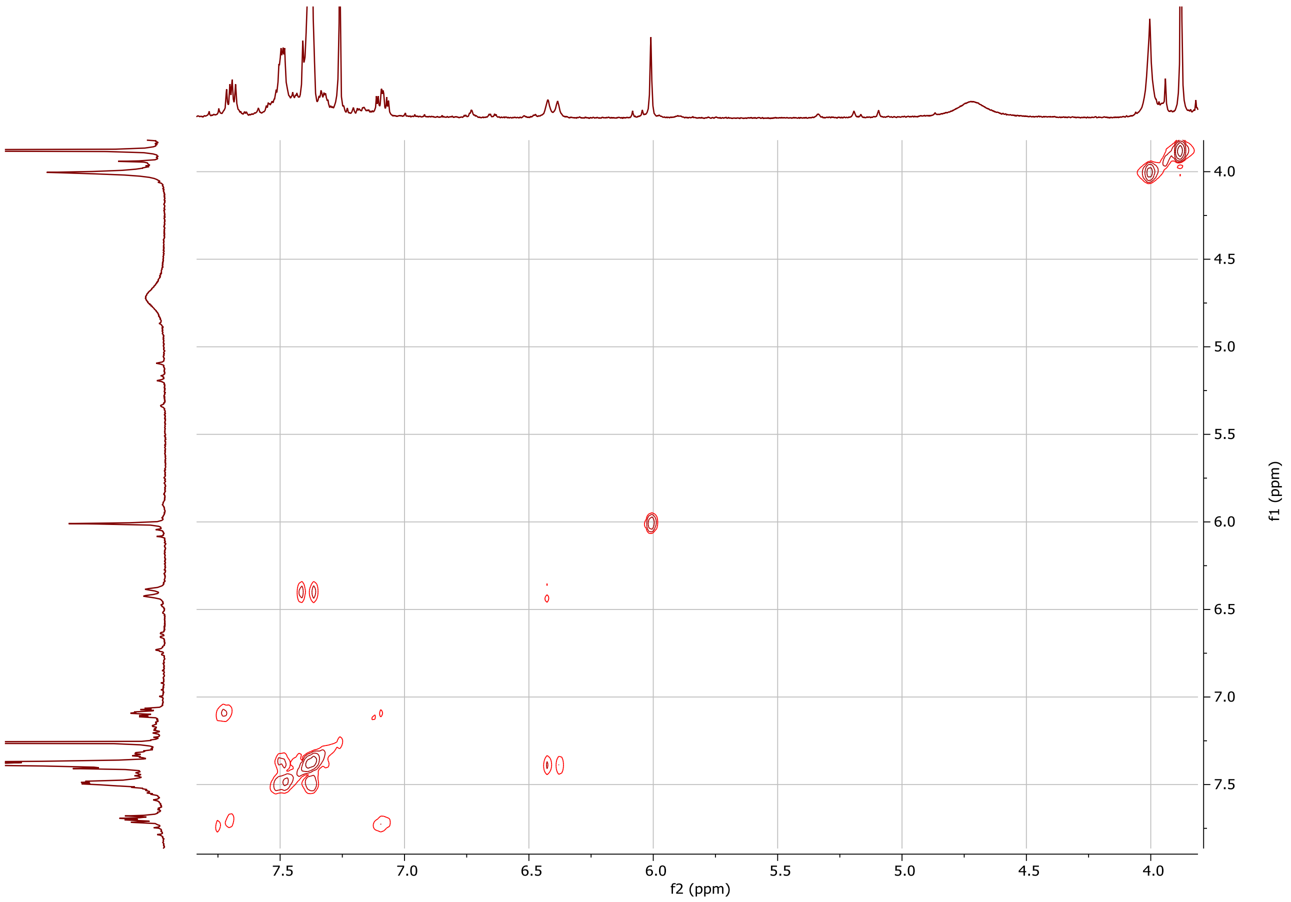

^1H-^1H COSY NMR spectrum of **5b**

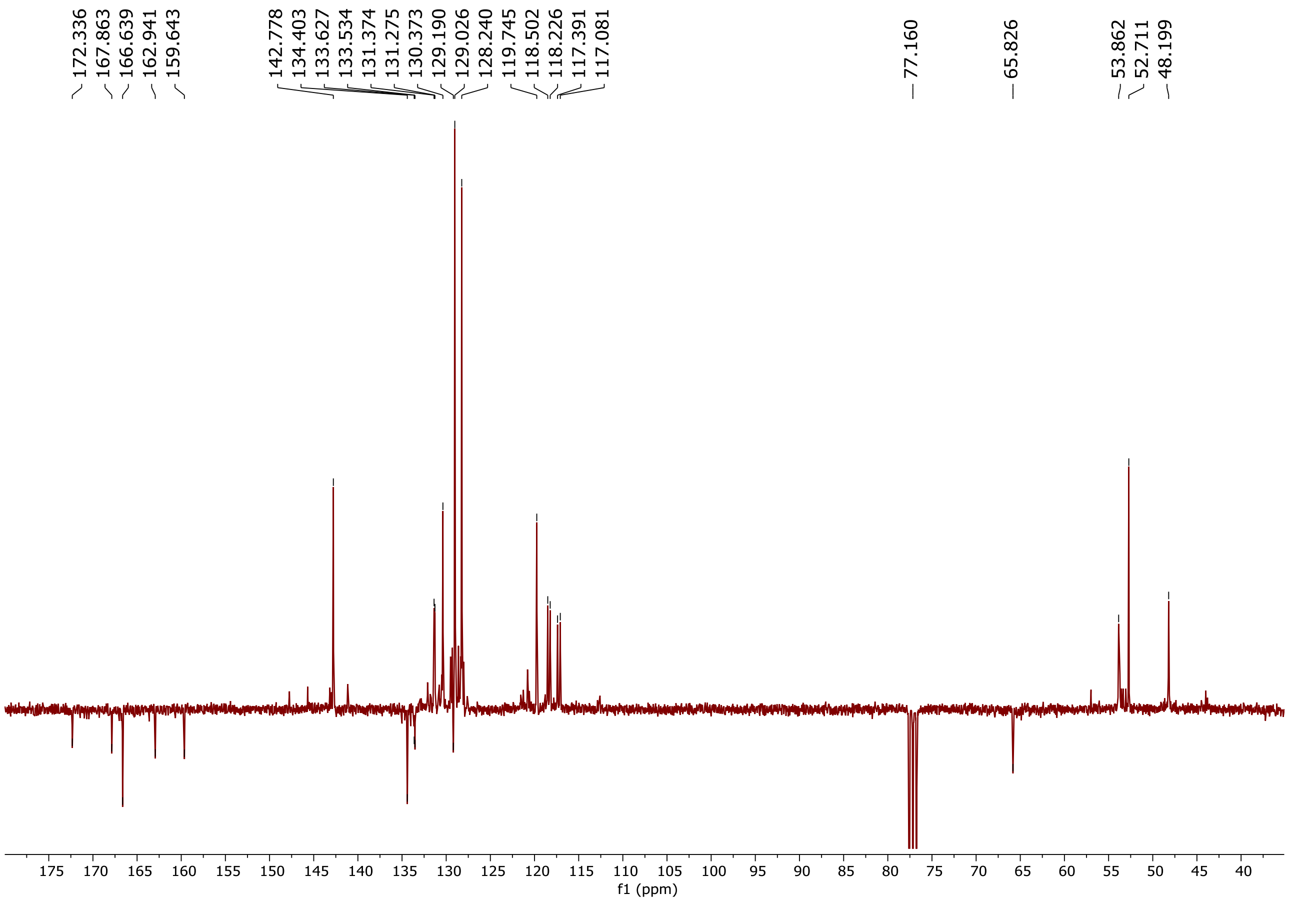

$^{13}C\{^1H\}$-(APT) NMR spectrum ($CDCl_3$, 75.47 MHz) of **5b**

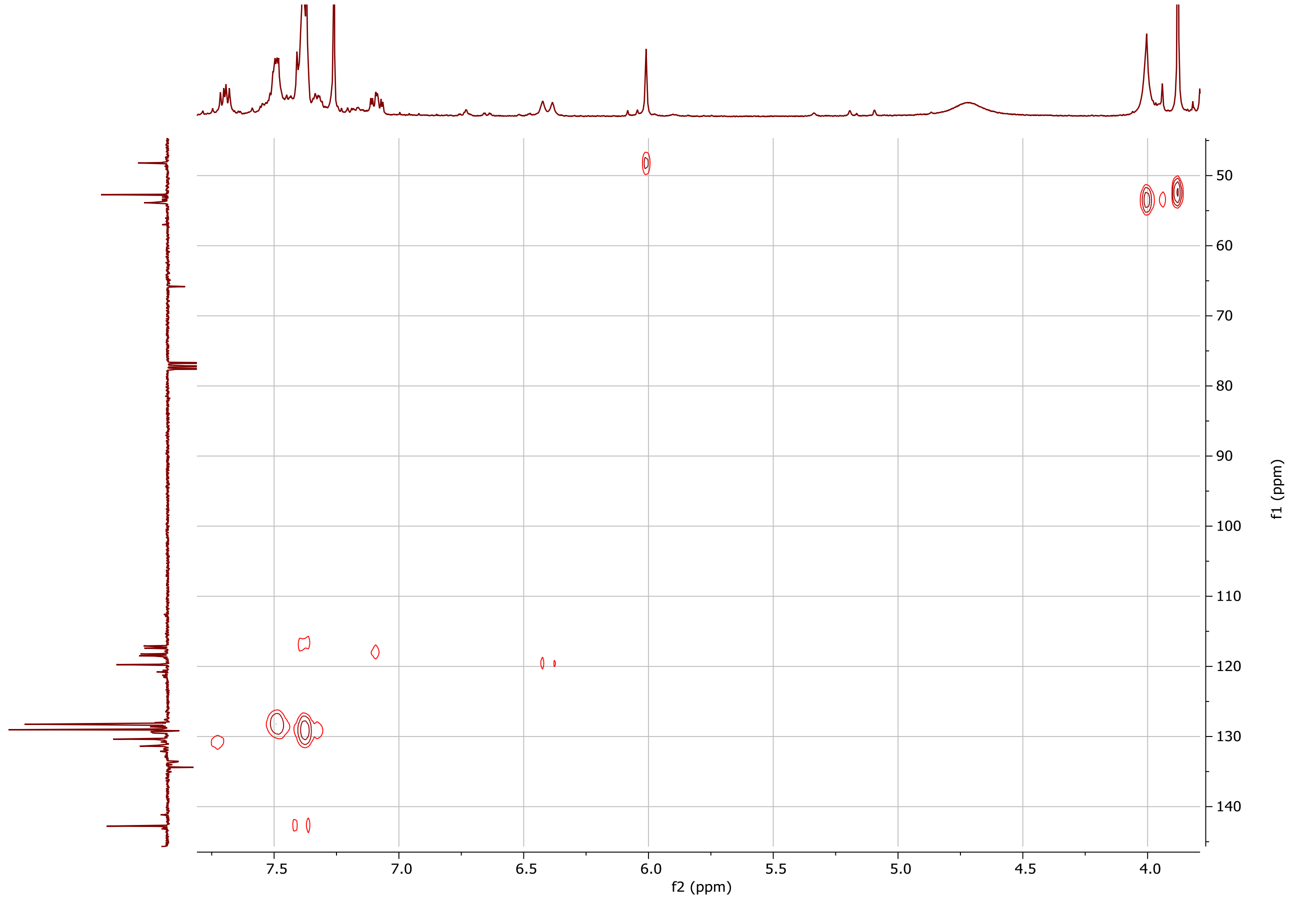

^{1}H-^{13}C HSQC NMR spectrum of **5b**

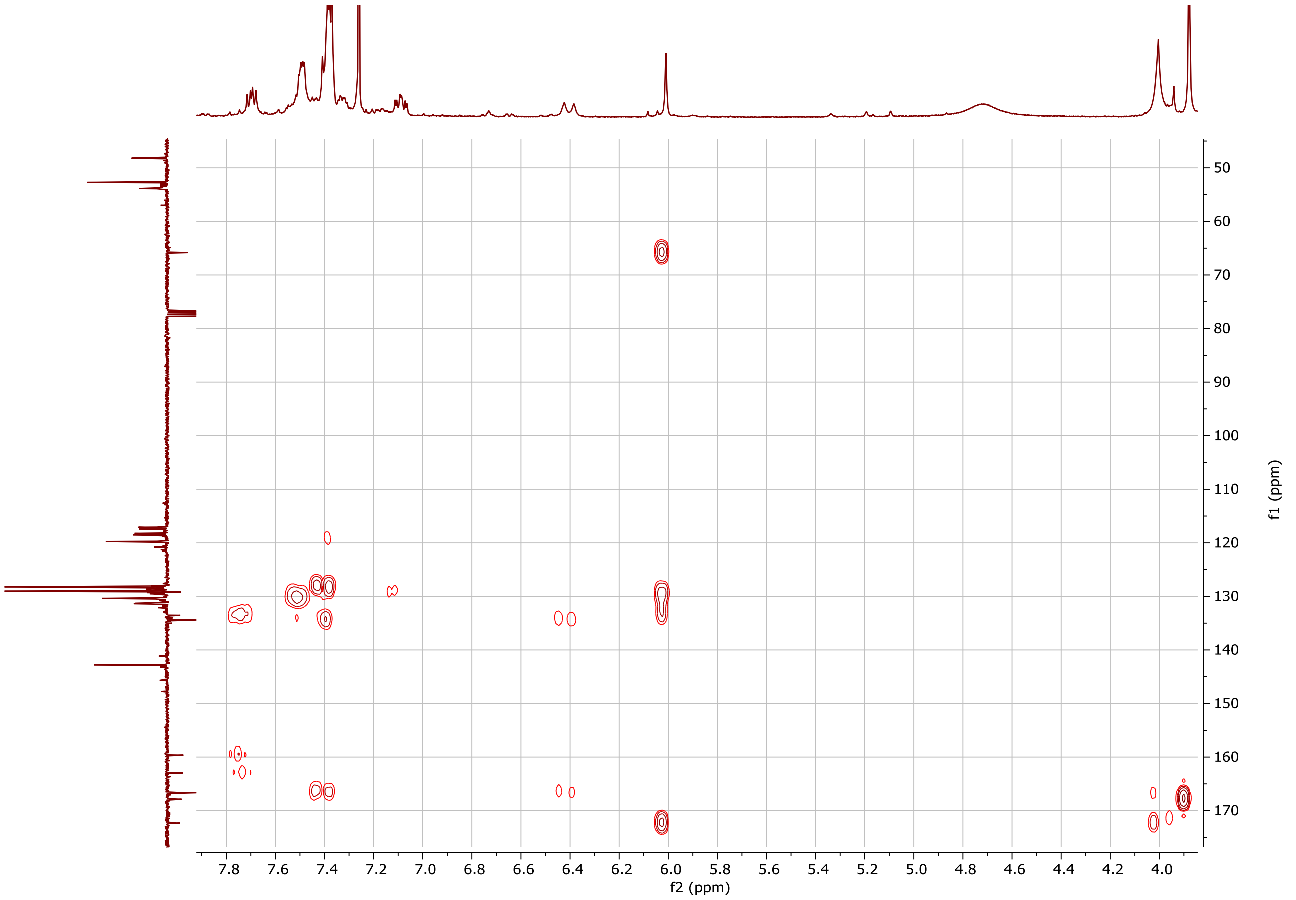

^{1}H-^{13}C HMBC NMR spectrum of **5b**

Diaminotruxillic cyclobutane 5d.

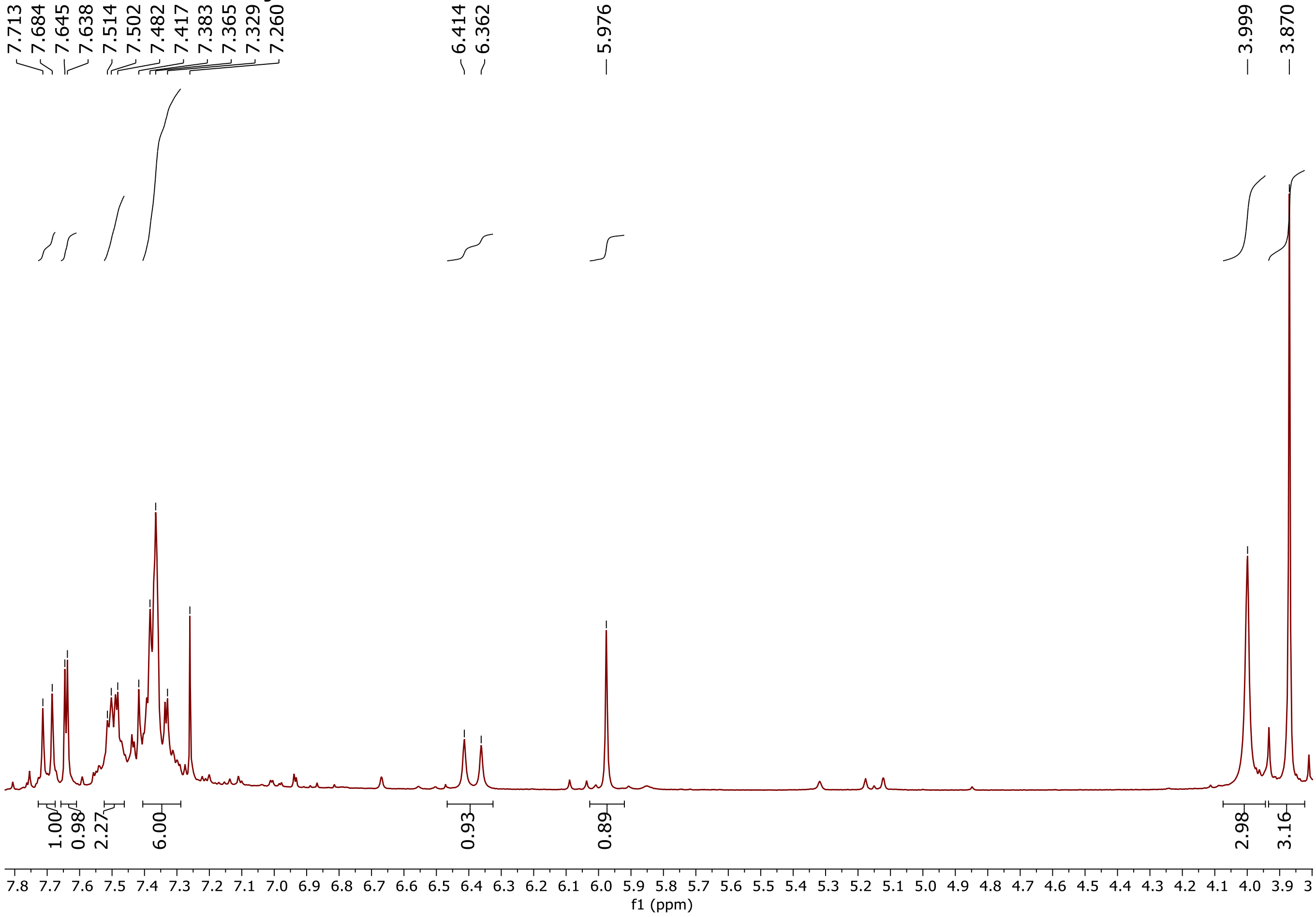

^{1}H NMR ($CDCl_3$, 300.13 MHz) of **5d**

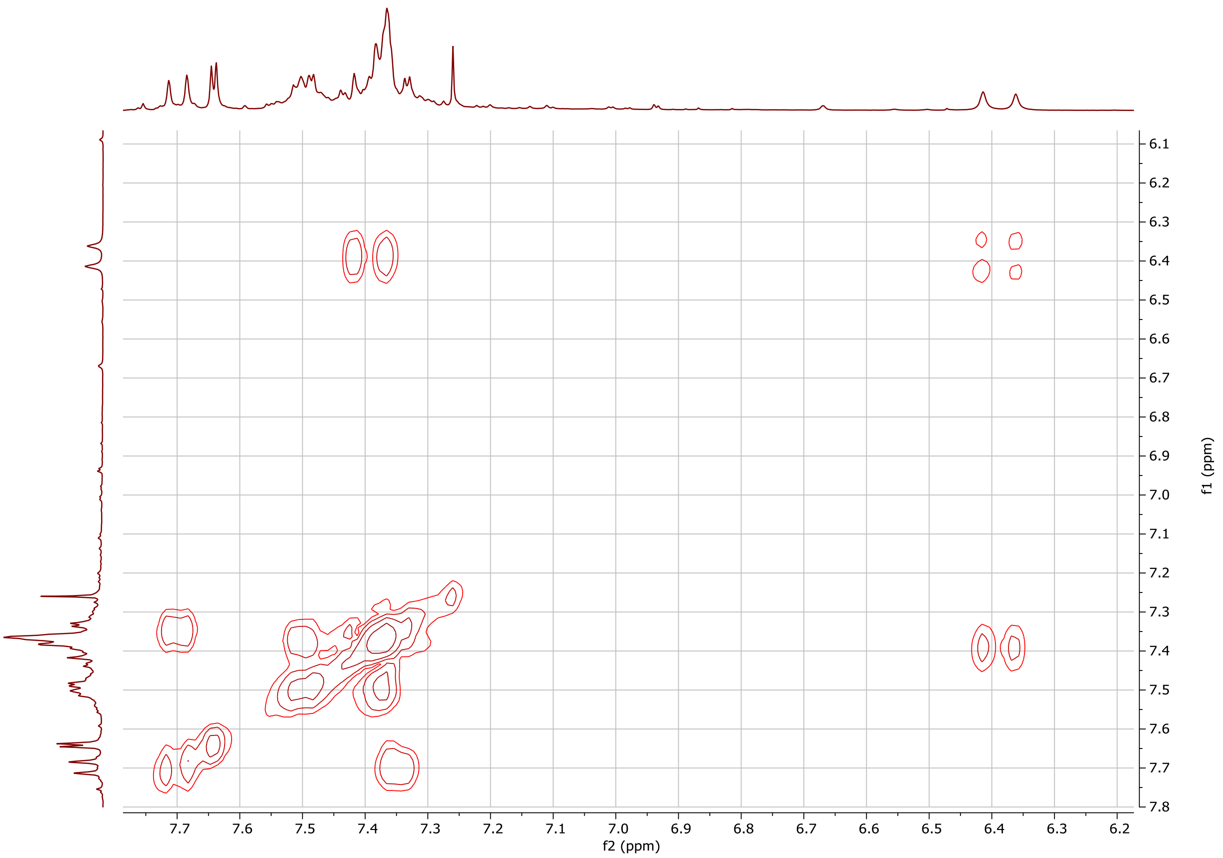

^{1}H-^{1}H COSY NMR spectrum of **5d**

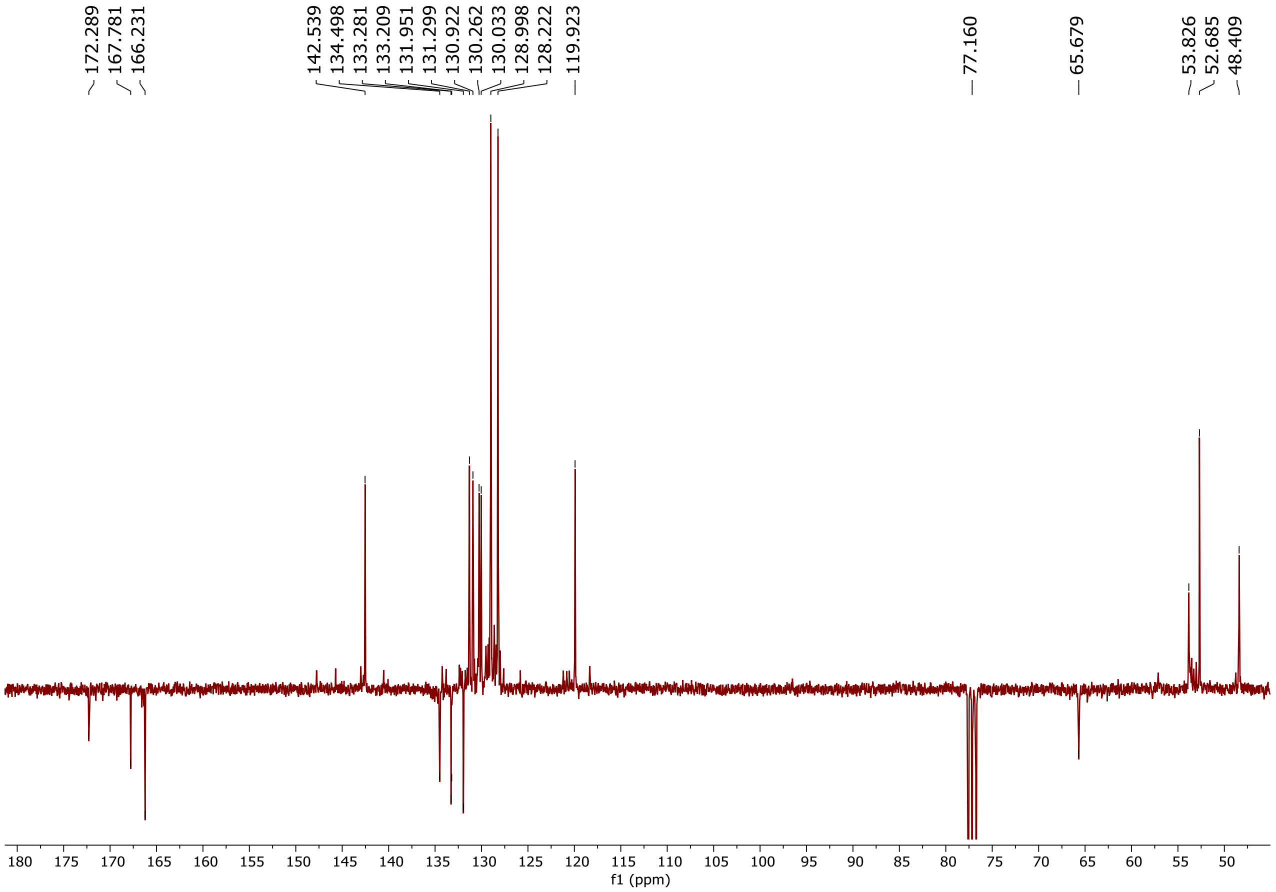

$^{13}C\{^{1}H\}$-(APT) NMR spectrum ($CDCl_3$, 75.47 MHz) of **5d**

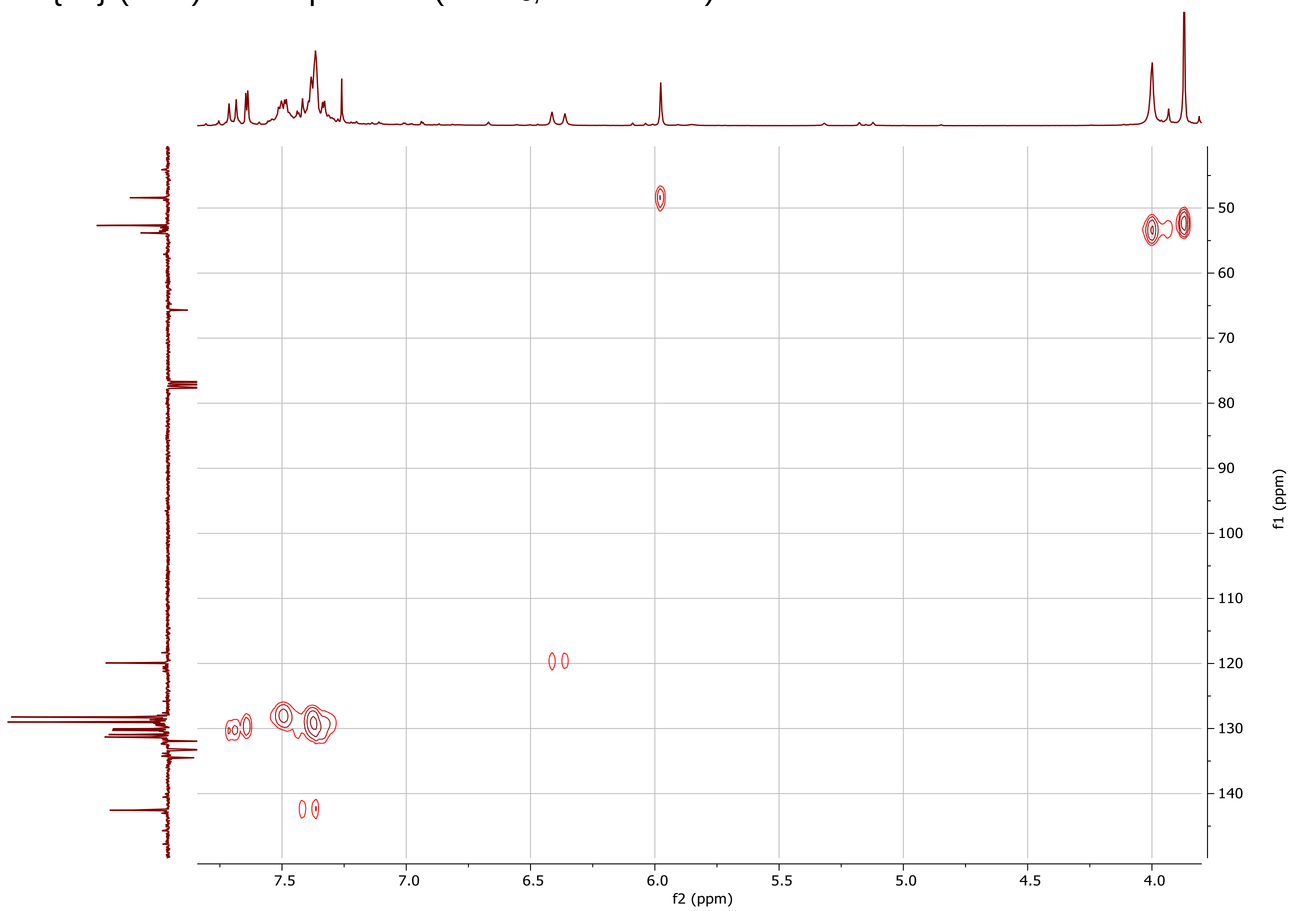

^{1}H-^{13}C HSQC NMR spectrum of **5d**

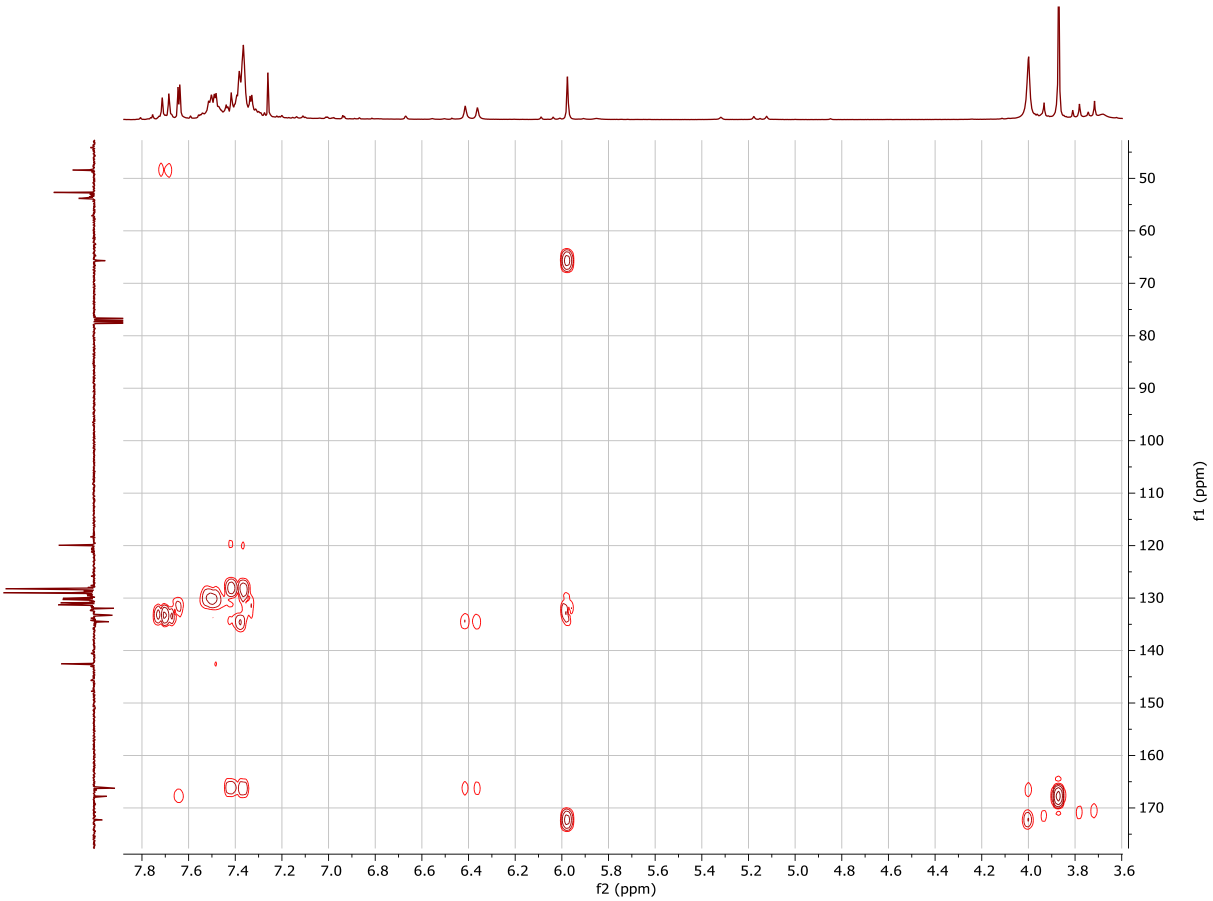

^{1}H-^{13}C HMBC NMR spectrum of **5d**

Diaminotruxillic cyclobutane 5f

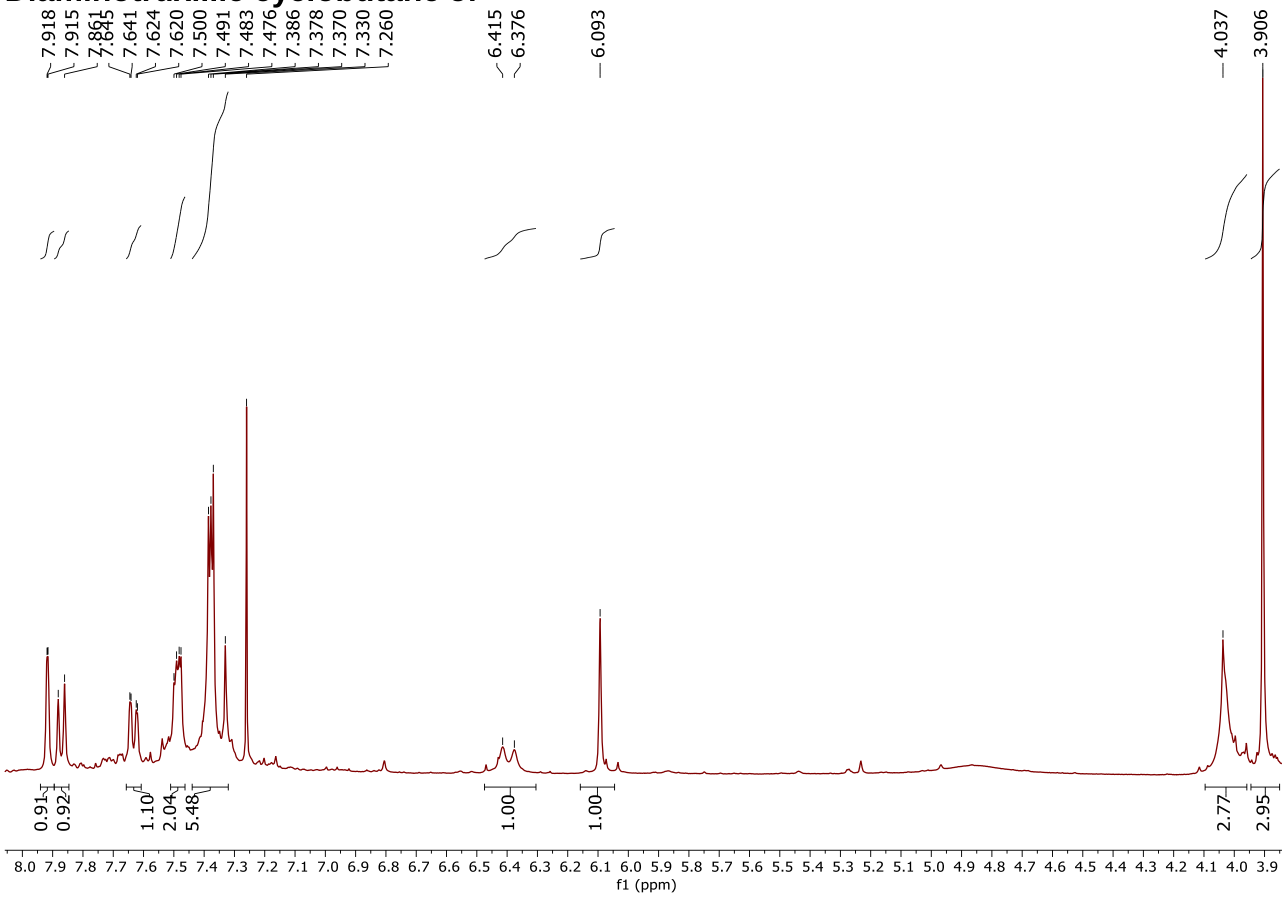

^{1}H NMR ($CDCl_3$, 300.13 MHz) of **5f**

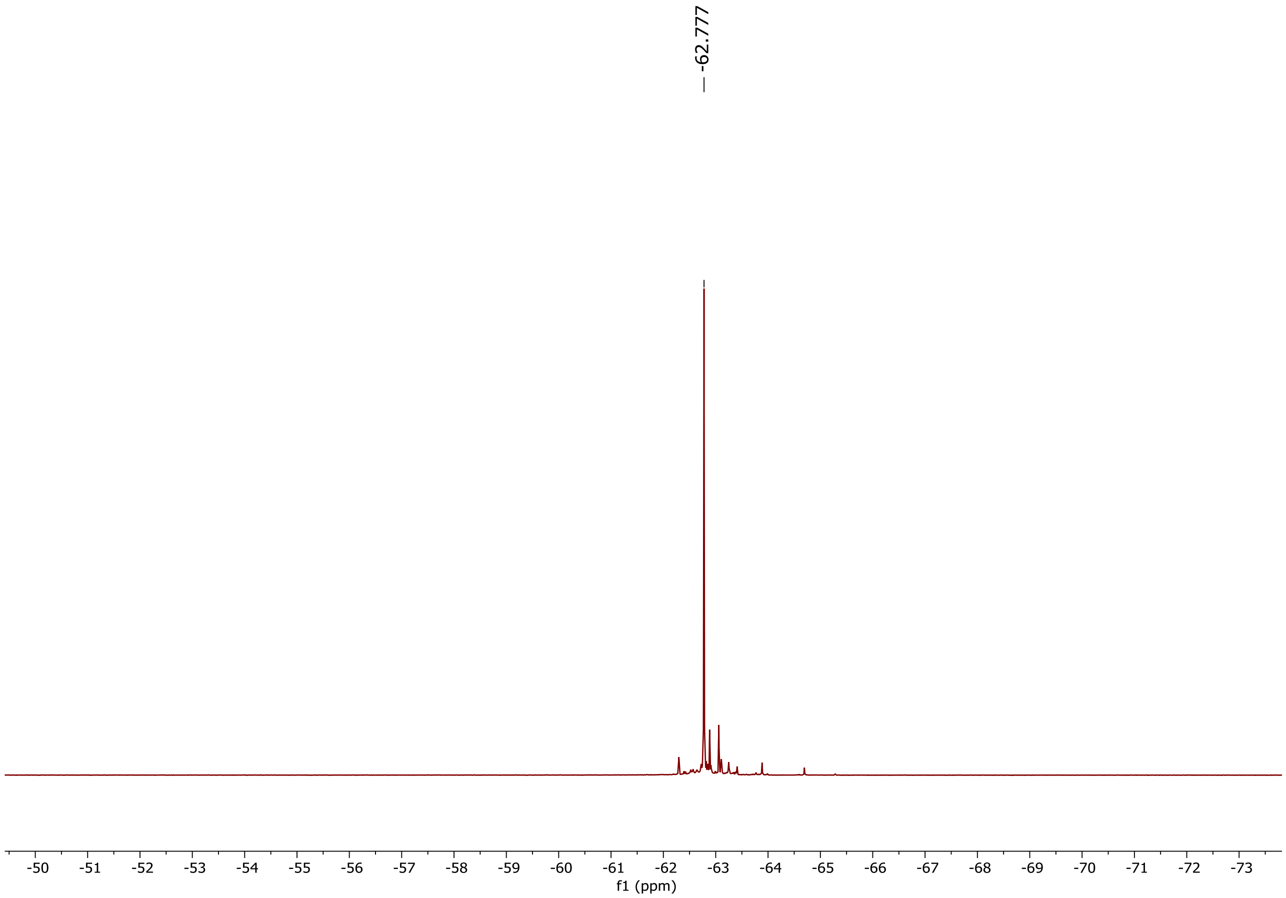

^{19}F NMR spectrum ($CDCl_3$, 282.40 MHz) of **5f**

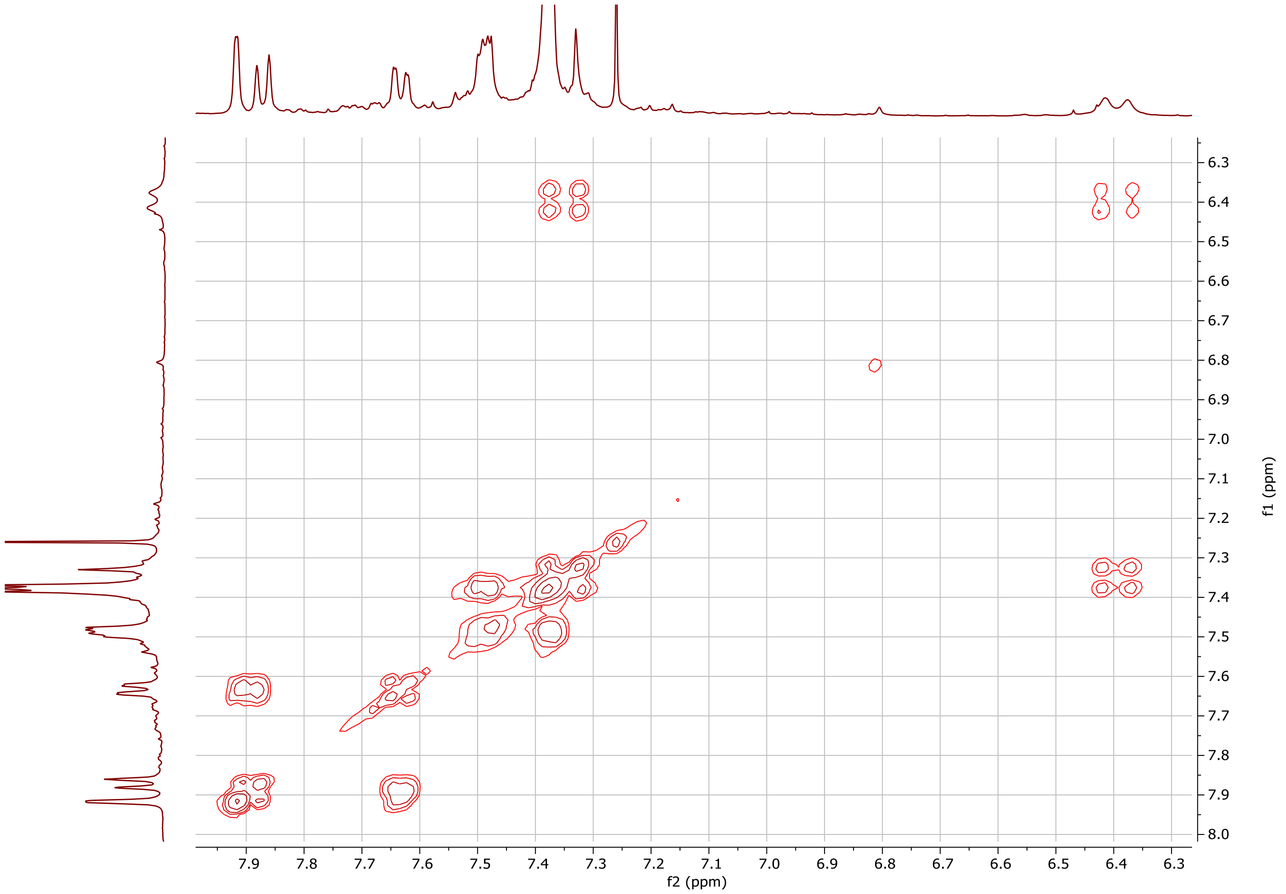

^1H-^1H COSY NMR spectrum of **5f**

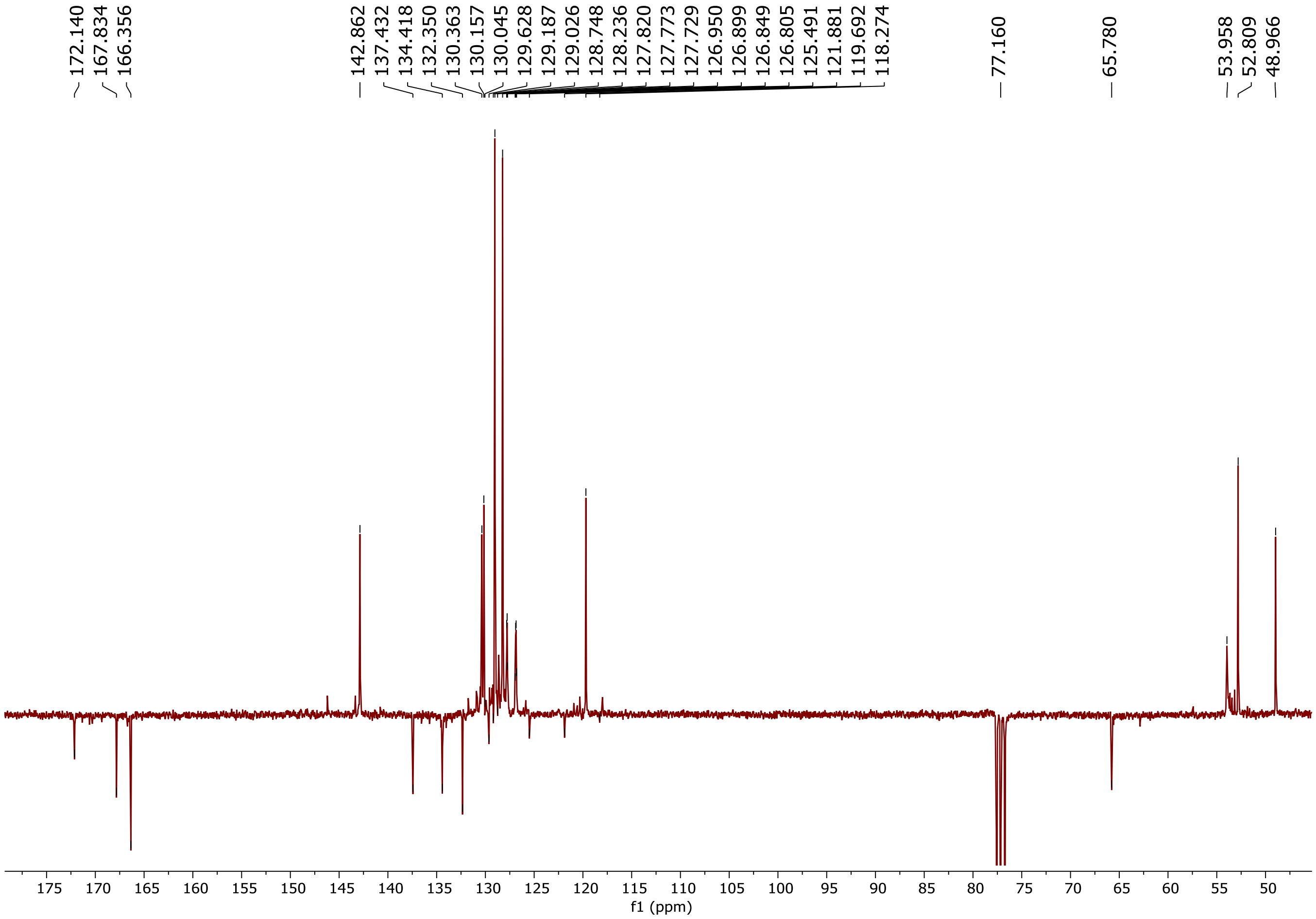

$^{13}C\{^1H\}$-(APT) NMR spectrum ($CDCl_3$, 75.47 MHz) of **5f**

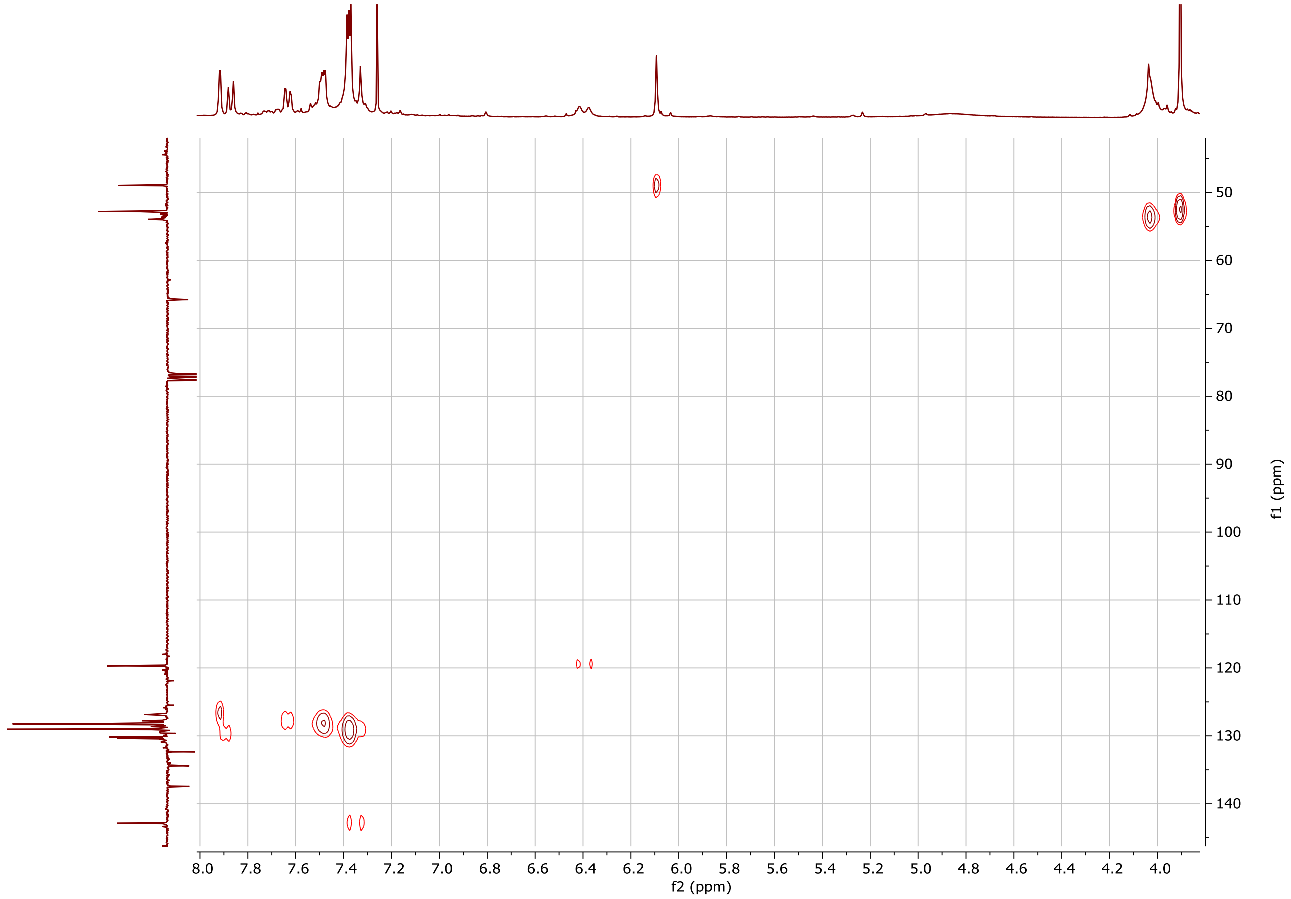

^{1}H-^{13}C HSQC NMR spectrum of **5f**

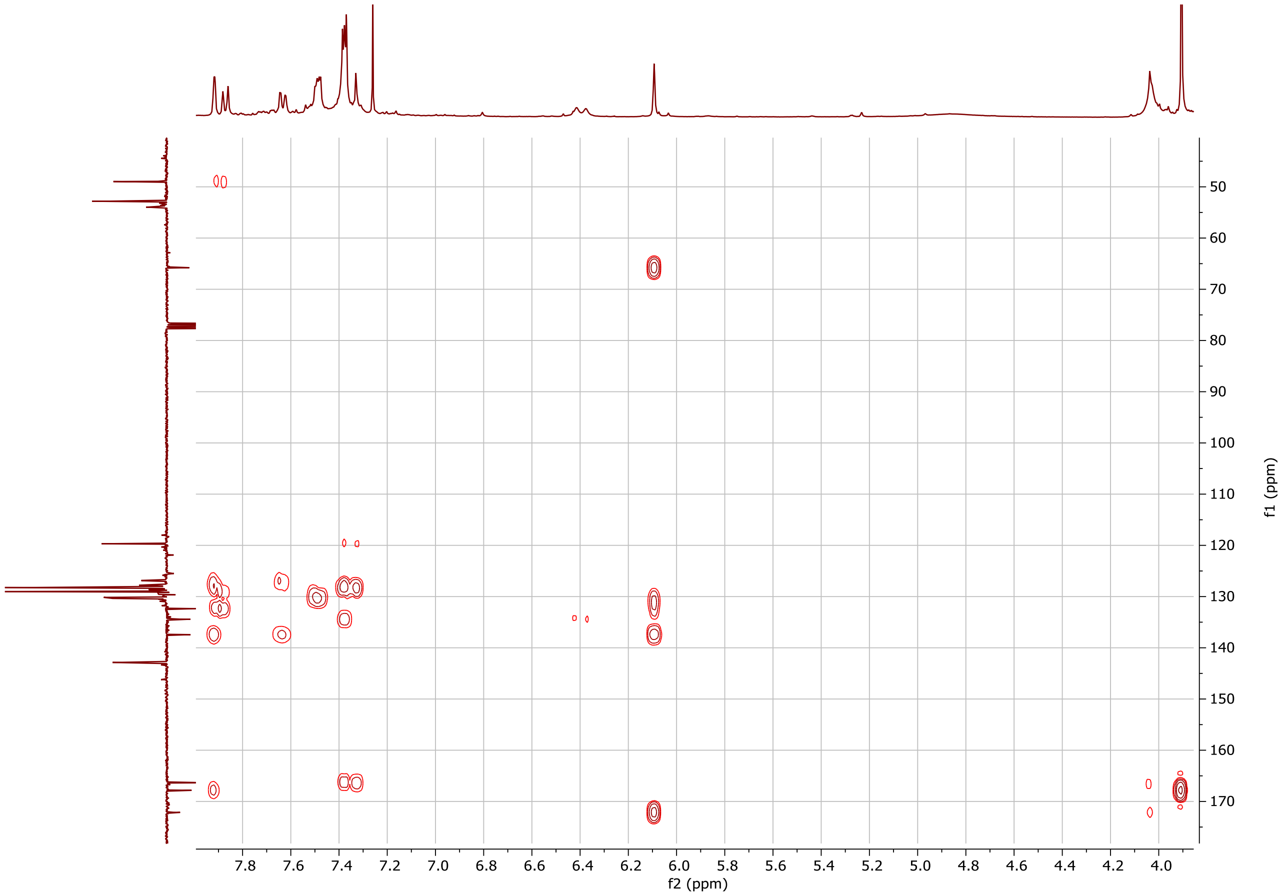

^{1}H-^{13}C HMBC NMR spectrum of **5f**

Diaminotruxillic cyclobutane 5h

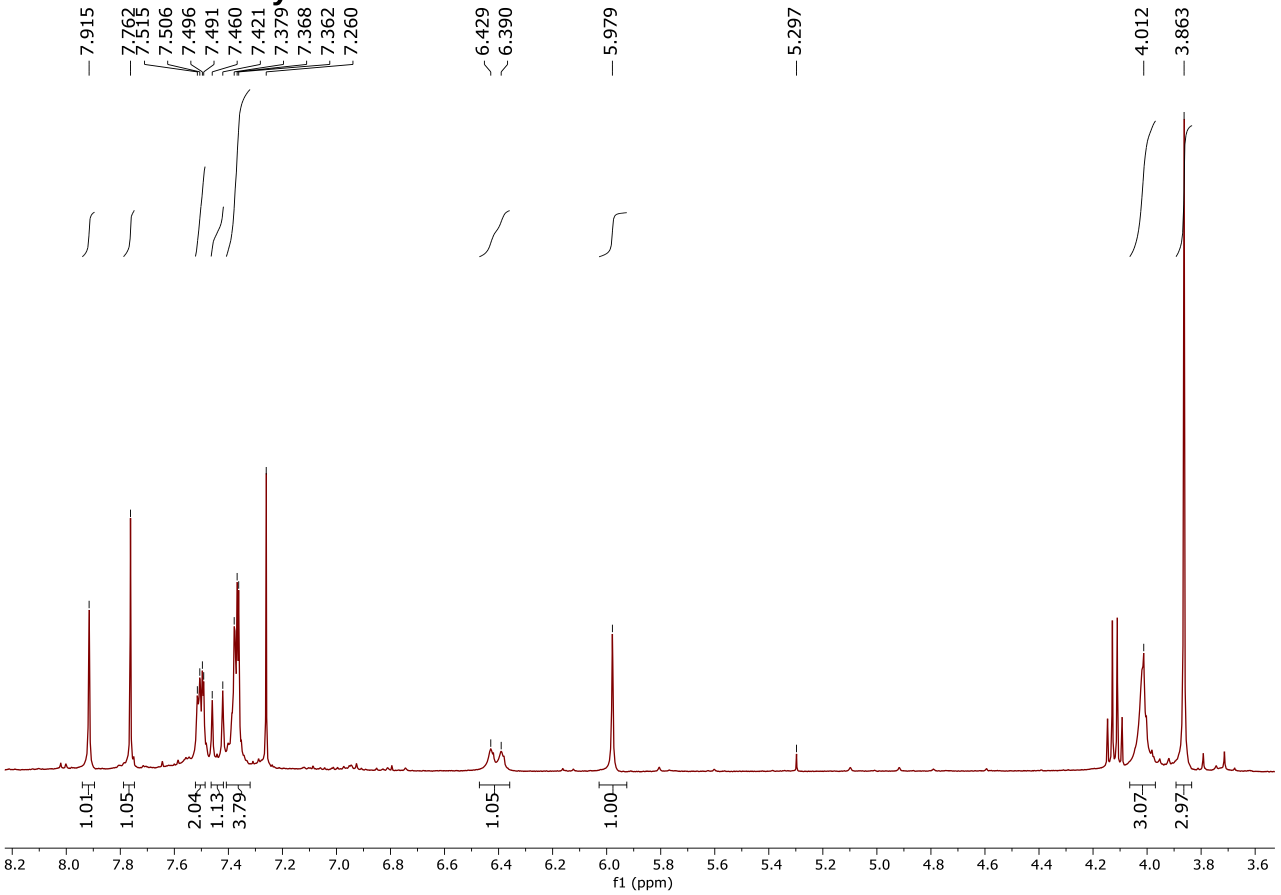

^{1}H NMR ($CDCl_3$, 300.13 MHz) of **5h**

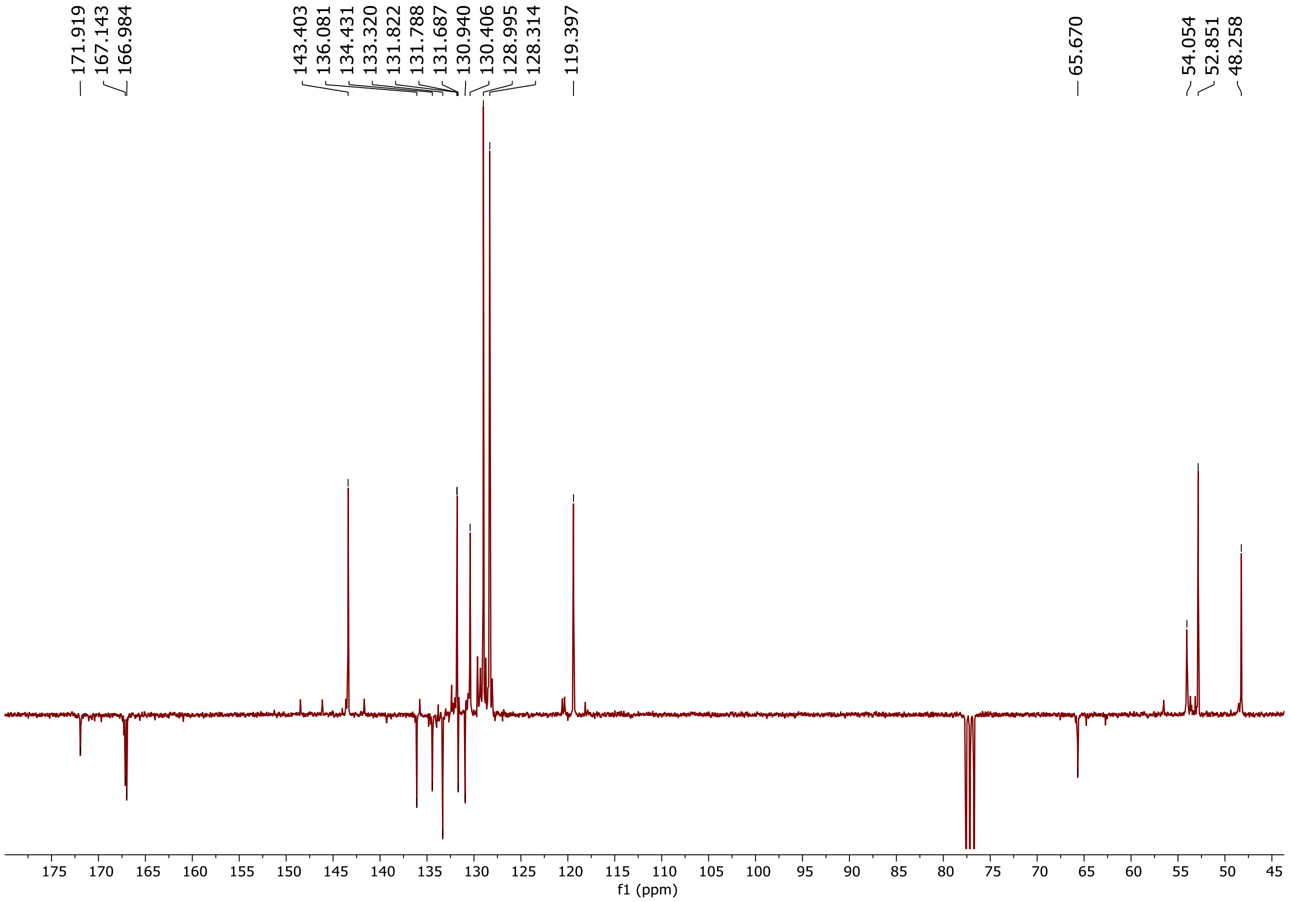

$^{13}C\{^{1}H\}$-(APT) NMR spectrum ($CDCl_3$, 75.47 MHz) of **5h**

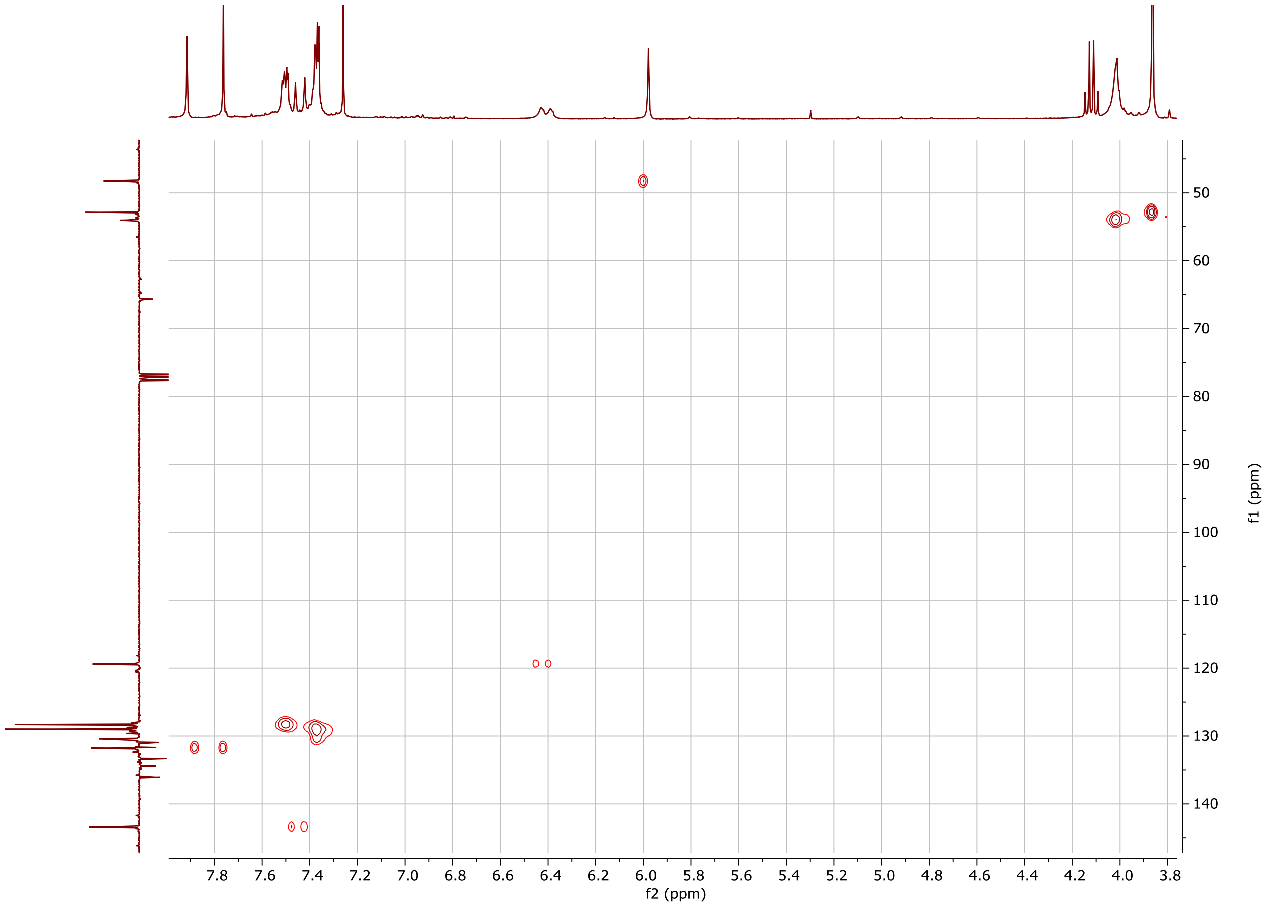

^{1}H-^{13}C HSQC NMR spectrum of **5h**

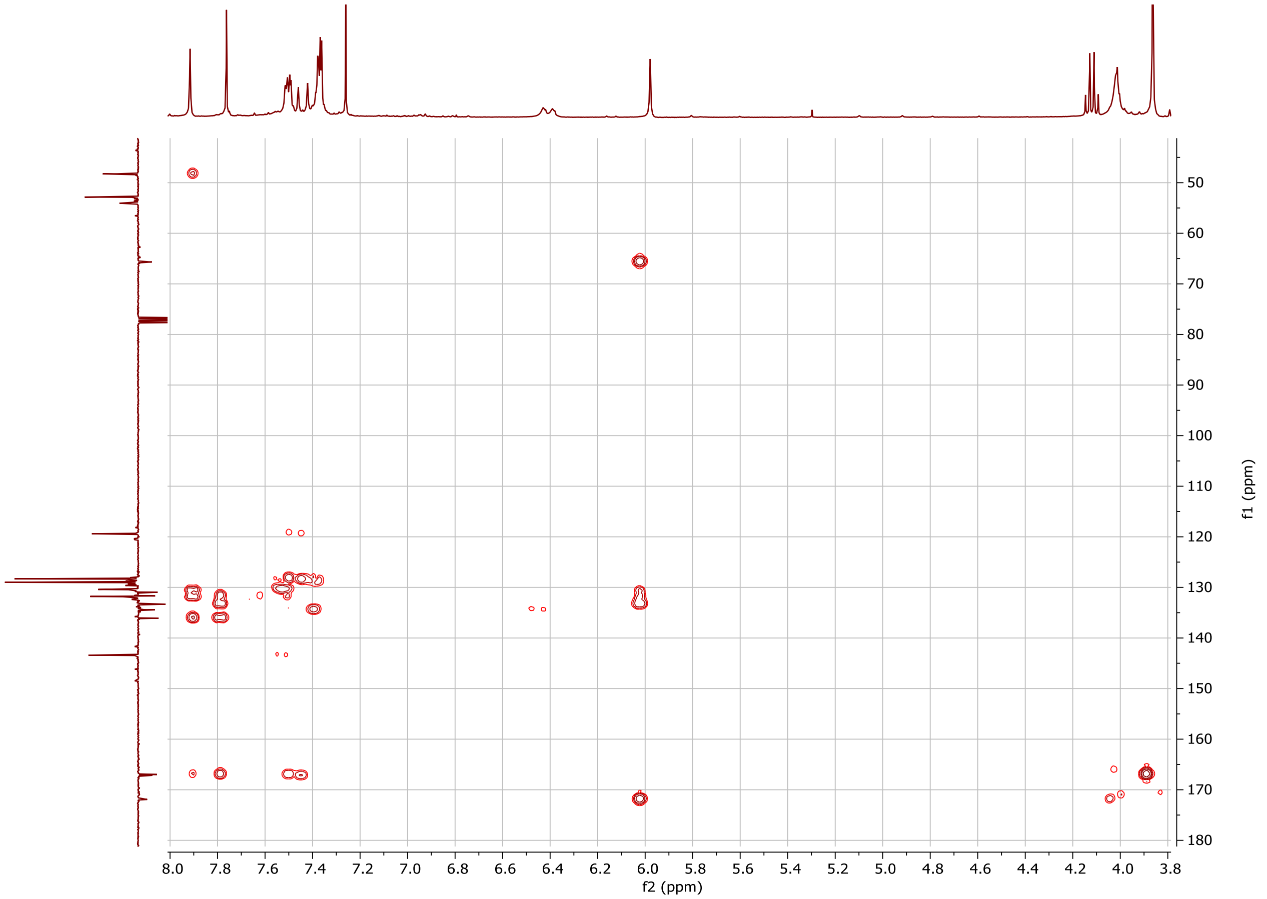

^{1}H-^{13}C HMBC NMR spectrum of **5h**

Diaminotruxillic cyclobutane 5j

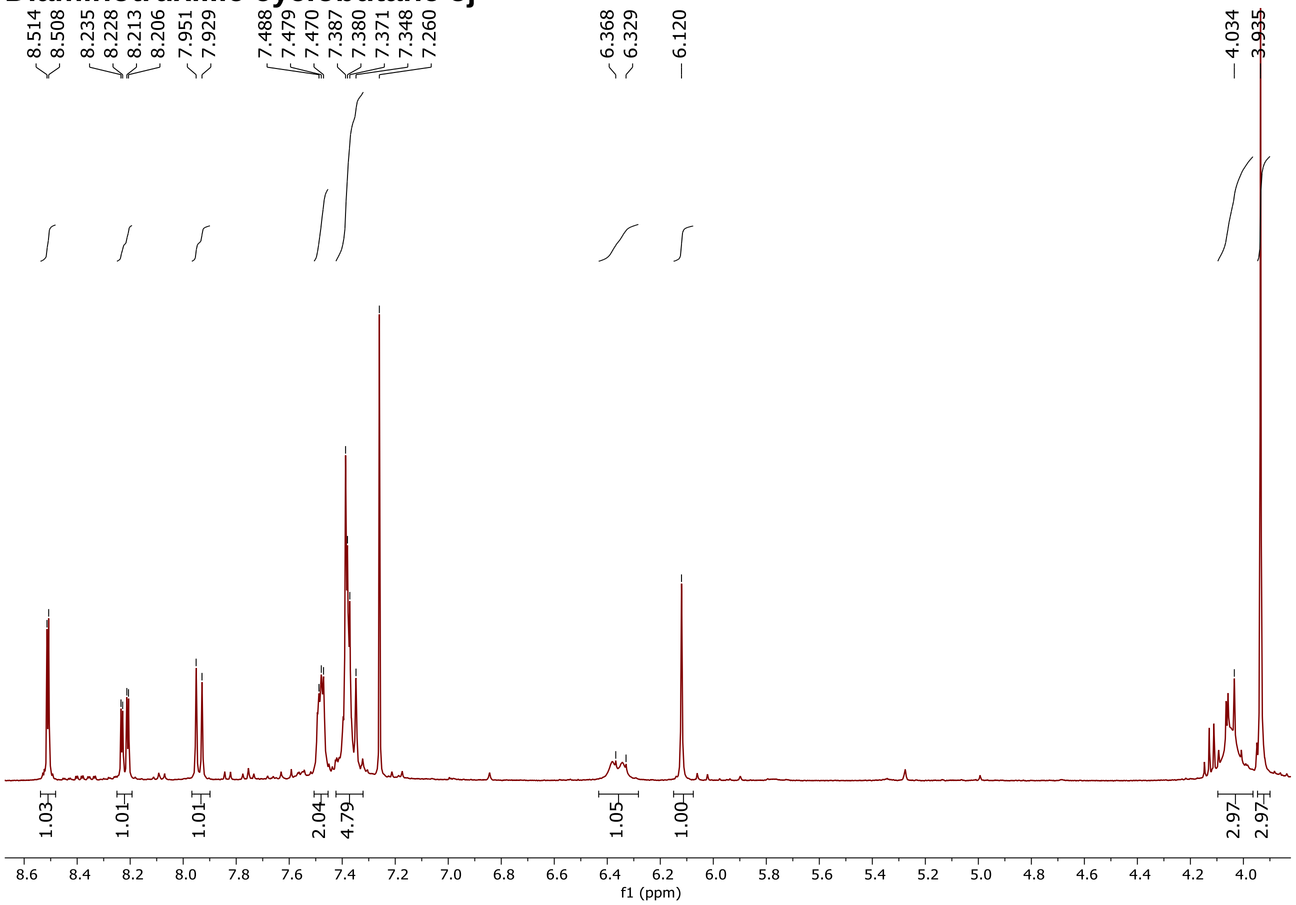

^{1}H NMR ($CDCl_3$, 400.13 MHz) of **5j**

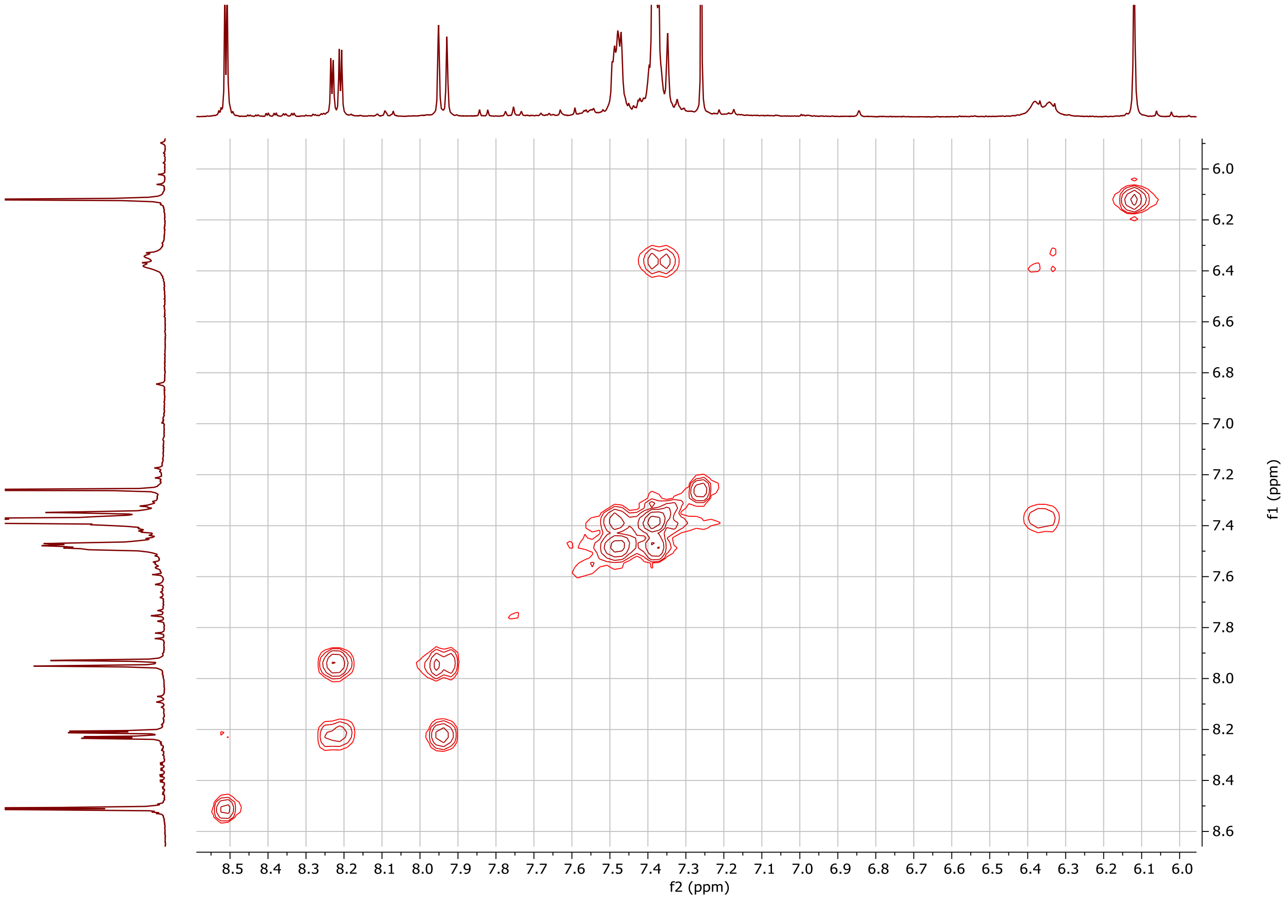

^1H-^1H COSY NMR spectrum of **5j**

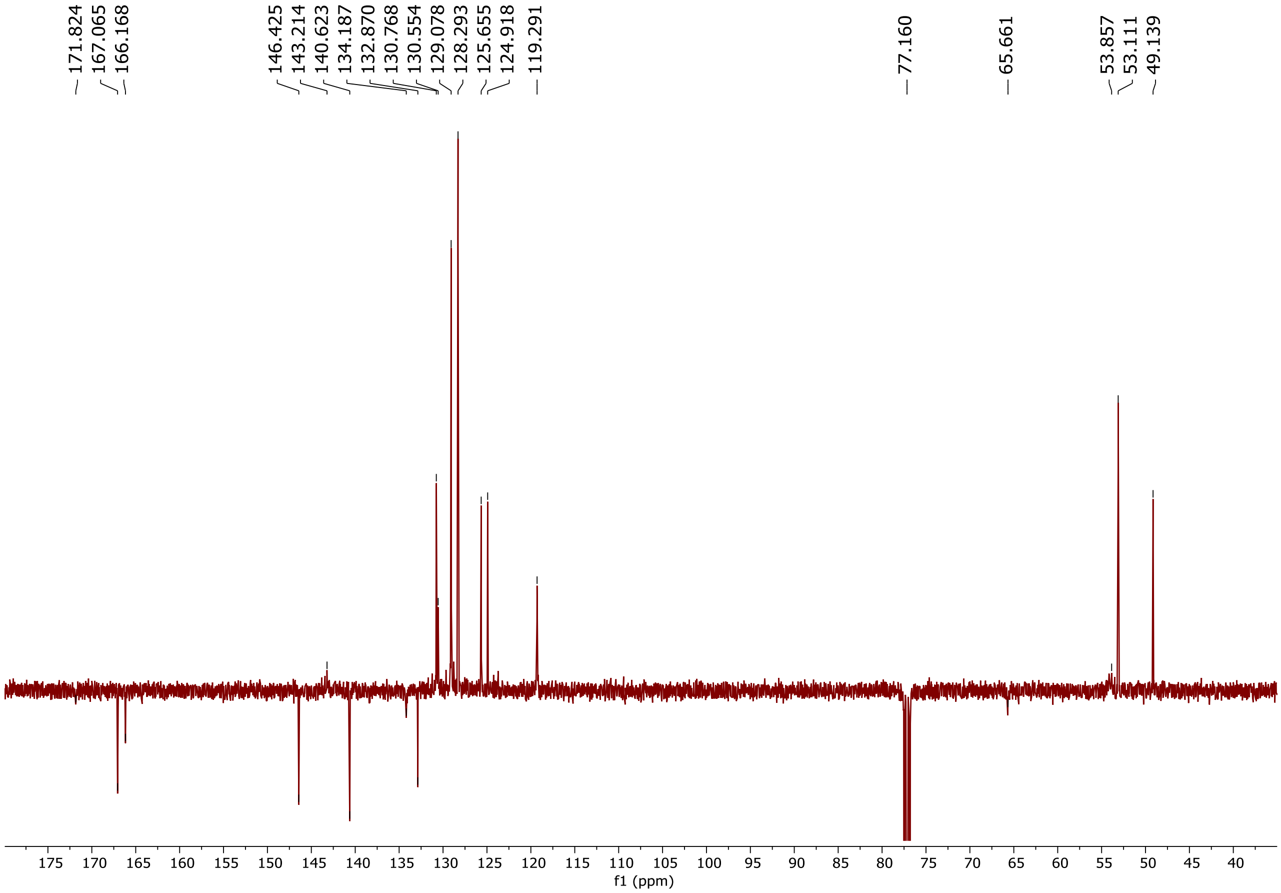

$^{13}C\{^1H\}$-(APT) NMR spectrum ($CDCl_3$, 75.47 MHz) of **5j**

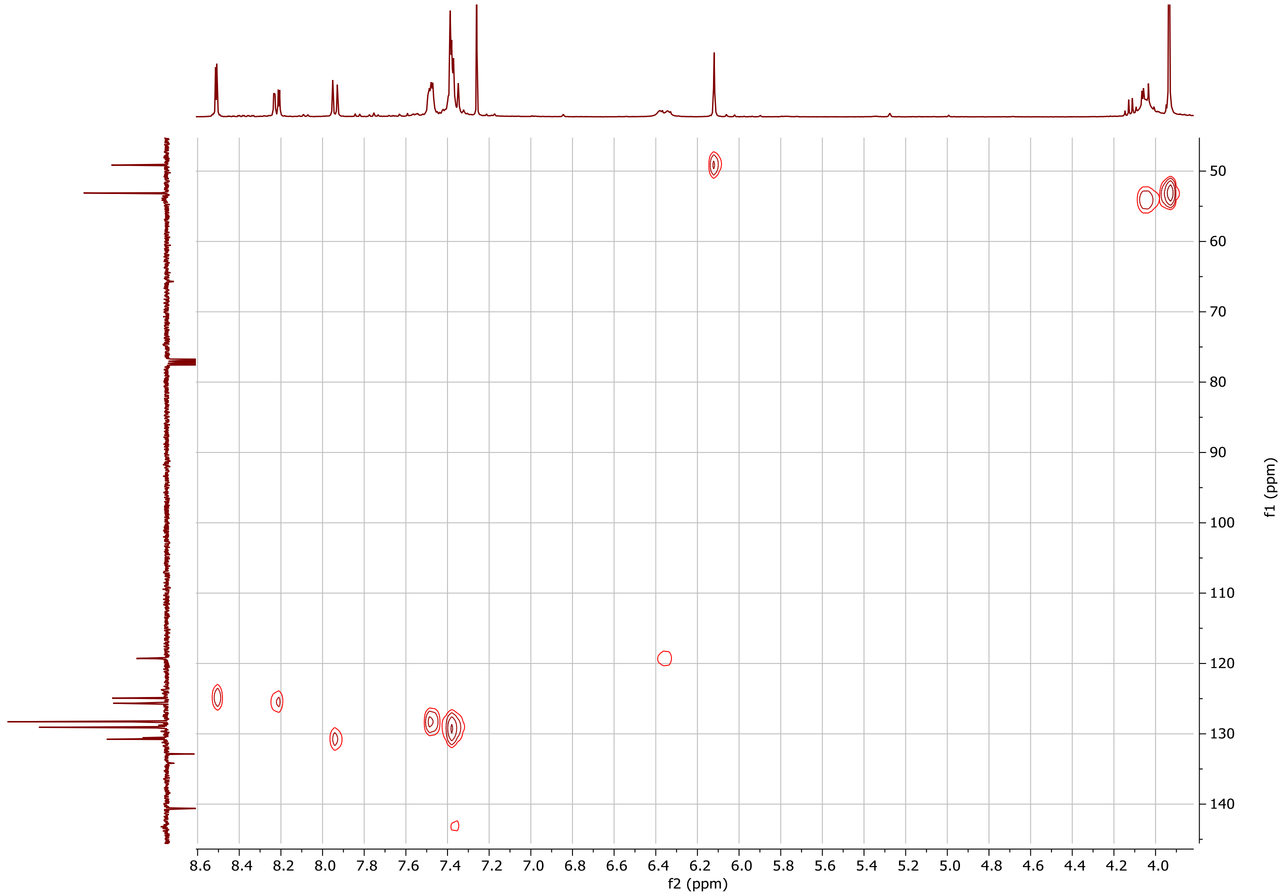

^{1}H-^{13}C HSQC NMR spectrum of **5j**

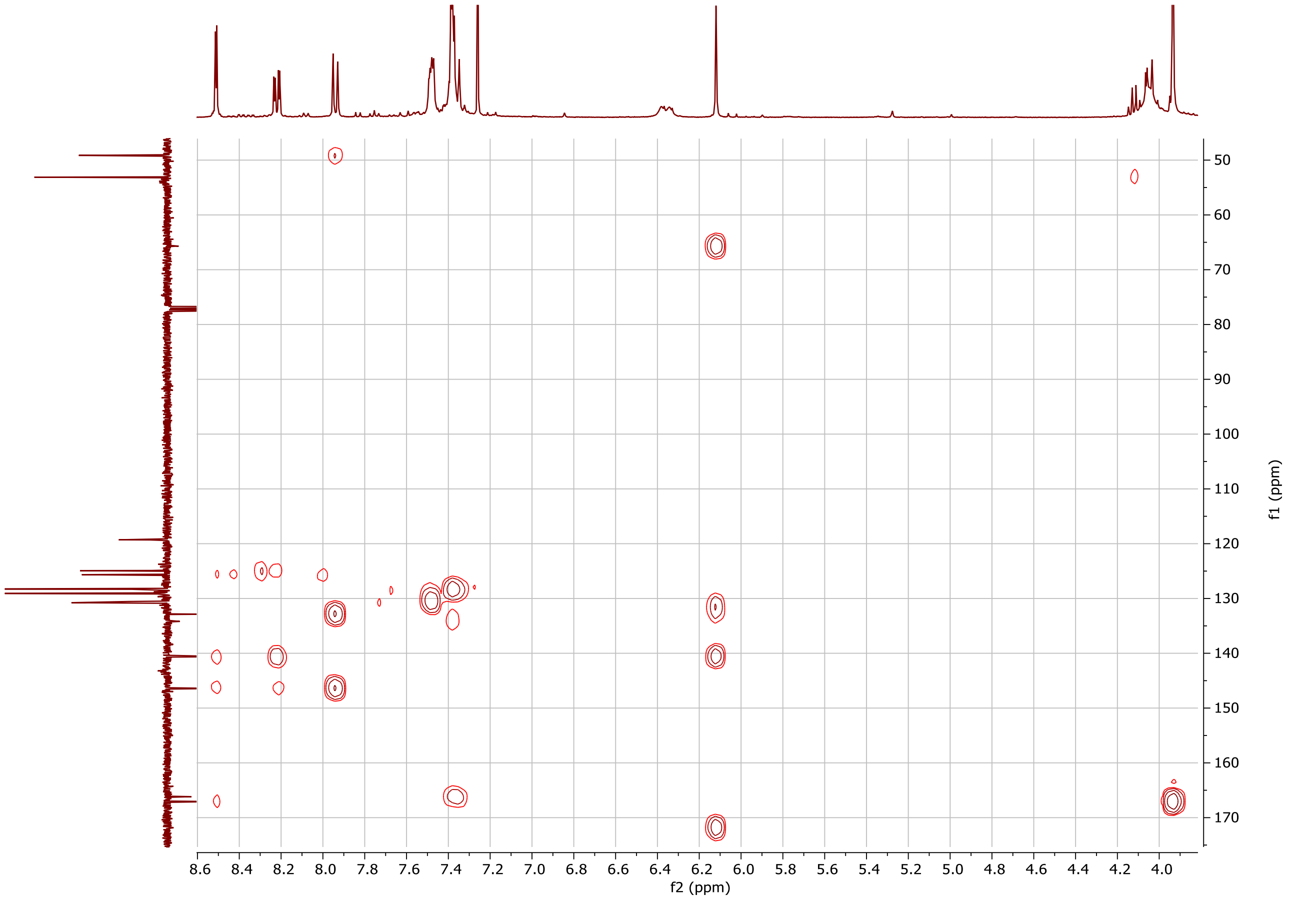

^{1}H-^{13}C HMBC NMR spectrum of **5j**

4.- X-RAY DIFFRACTION METHODS

4.1. Crystallographic data for compound 2c

Table S1. Crystal data and structure refinement for 2c.

Empirical formula	C18 H12 Cl N O2	
Formula weight	309.74	
Temperature	100(2) K	
Wavelength	0.71073 Å	
Crystal system	Monoclinic	
Space group	P2(1)/c	
Unit cell dimensions	a = 10.2286(5) Å	α= 90°.
	b = 16.9895(8) Å	β= 98.0460(10)°.
	c = 8.3844(4) Å	γ = 90°.
Volume	1442.69(12) $Å^3$	
Z	4	
Density (calculated)	1.426 Mg/m^3	
Absorption coefficient	0.271 mm^{-1}	
F(000)	640	
Crystal size	0.28 x 0.18 x 0.16 mm^3	
Theta range for data collection	2.01 to 29.02°.	
Index ranges	-13<=h<=13, -22<=k<=22, -11<=l<=11	
Reflections collected	28273	
Independent reflections	3676 [R(int) = 0.0186]	
Completeness to theta = 29.02°	95.7 %	
Absorption correction	Semi-empirical from equivalents	
Max. and min. transmission	0.958 and 0.909	
Refinement method	Full-matrix least-squares on F^2	
Data / restraints / parameters	3676 / 0 / 199	
Goodness-of-fit on F^2	1.020	
Final R indices [I>2sigma(I)]	R1 = 0.0313, wR2 = 0.0766	
R indices (all data)	R1 = 0.0338, wR2 = 0.0787	
Largest diff. peak and hole	0.400 and -0.218 e.$Å^{-3}$	

Table S2. Atomic coordinates (× 10^4) and equivalent isotropic displacement parameters ($Å^2$ × 10^3) for 2c. U(eq) is defined as one third of the trace of the orthogonalized U^{ij} tensor.

	x	y	z	U(eq)
Cl(1)	6919(1)	845(1)	5288(1)	22(1)
O(1)	11122(1)	2433(1)	5272(1)	22(1)
O(2)	12598(1)	1978(1)	3708(1)	18(1)
N(1)	11543(1)	886(1)	2604(1)	17(1)
C(1)	8748(1)	330(1)	3424(1)	15(1)
C(2)	7519(1)	196(1)	3956(1)	16(1)
C(3)	6741(1)	-454(1)	3462(1)	20(1)
C(4)	7179(1)	-990(1)	2403(1)	21(1)
C(5)	8381(1)	-871(1)	1835(1)	21(1)
C(6)	9151(1)	-223(1)	2339(1)	18(1)
C(7)	9547(1)	1008(1)	3996(1)	16(1)
C(8)	10739(1)	1231(1)	3633(1)	16(1)
C(9)	11414(1)	1946(1)	4349(1)	17(1)
C(10)	12577(1)	1333(1)	2694(1)	17(1)
C(11)	13703(1)	1217(1)	1866(1)	19(1)
C(12)	14744(1)	1708(1)	2036(1)	19(1)
C(13)	15937(1)	1636(1)	1270(1)	18(1)
C(14)	16960(1)	2177(1)	1707(1)	20(1)
C(15)	18141(1)	2122(1)	1071(1)	22(1)
C(16)	18317(1)	1527(1)	-11(2)	23(1)
C(17)	17307(1)	985(1)	-468(1)	22(1)
C(18)	16125(1)	1042(1)	161(1)	20(1)

Table S3. Bond lengths [Å] and angles [°] for 2c.

Cl(1)-C(2)	1.7413(11)
O(1)-C(9)	1.1990(14)
O(2)-C(10)	1.3861(13)
O(2)-C(9)	1.3928(13)
N(1)-C(10)	1.2951(14)
N(1)-C(8)	1.4009(14)
C(1)-C(6)	1.4092(15)
C(1)-C(2)	1.4096(14)
C(1)-C(7)	1.4543(15)
C(2)-C(3)	1.3906(15)
C(3)-C(4)	1.3892(16)
C(4)-C(5)	1.3938(16)
C(5)-C(6)	1.3844(15)
C(7)-C(8)	1.3513(15)
C(8)-C(9)	1.4816(14)
C(10)-C(11)	1.4396(15)
C(11)-C(12)	1.3437(15)
C(12)-C(13)	1.4616(15)
C(13)-C(14)	1.4027(15)
C(13)-C(18)	1.4036(16)
C(14)-C(15)	1.3898(16)
C(15)-C(16)	1.3863(17)
C(16)-C(17)	1.3969(17)
C(17)-C(18)	1.3885(16)
C(10)-O(2)-C(9)	105.39(8)
C(10)-N(1)-C(8)	105.32(9)
C(6)-C(1)-C(2)	116.81(10)
C(6)-C(1)-C(7)	122.60(10)
C(2)-C(1)-C(7)	120.59(10)
C(3)-C(2)-C(1)	122.19(10)
C(3)-C(2)-Cl(1)	117.13(8)
C(1)-C(2)-Cl(1)	120.67(8)
C(4)-C(3)-C(2)	119.22(10)
C(3)-C(4)-C(5)	120.17(10)
C(6)-C(5)-C(4)	120.15(10)
C(5)-C(6)-C(1)	121.45(10)
C(8)-C(7)-C(1)	129.18(10)

C(7)-C(8)-N(1)	130.31(10)
C(7)-C(8)-C(9)	121.53(10)
N(1)-C(8)-C(9)	108.15(9)
O(1)-C(9)-O(2)	122.03(10)
O(1)-C(9)-C(8)	133.05(10)
O(2)-C(9)-C(8)	104.92(9)
N(1)-C(10)-O(2)	116.21(9)
N(1)-C(10)-C(11)	126.45(10)
O(2)-C(10)-C(11)	117.34(9)
C(12)-C(11)-C(10)	122.64(10)
C(11)-C(12)-C(13)	126.80(11)
C(14)-C(13)-C(18)	118.54(10)
C(14)-C(13)-C(12)	117.95(10)
C(18)-C(13)-C(12)	123.48(10)
C(15)-C(14)-C(13)	120.81(11)
C(16)-C(15)-C(14)	119.97(11)
C(15)-C(16)-C(17)	120.11(11)
C(18)-C(17)-C(16)	119.95(11)
C(17)-C(18)-C(13)	120.62(11)

Symmetry transformations used to generate equivalent atoms:

Table S4. Anisotropic displacement parameters ($Å^2$x 10^3)for 2c. The anisotropic displacement factor exponent takes the form: $-2\pi^2$[$h^2a^{*2}U^{11}$ + ... + 2 h k a* b* U^{12}]

	U^{11}	U^{22}	U^{33}	U^{23}	U^{13}	U^{12}
Cl(1)	21(1)	24(1)	23(1)	-4(1)	9(1)	-1(1)
O(1)	20(1)	18(1)	28(1)	-4(1)	6(1)	0(1)
O(2)	16(1)	17(1)	22(1)	-2(1)	4(1)	-2(1)
N(1)	16(1)	18(1)	17(1)	1(1)	4(1)	0(1)
C(1)	16(1)	16(1)	15(1)	2(1)	2(1)	1(1)
C(2)	17(1)	18(1)	15(1)	1(1)	3(1)	1(1)
C(3)	16(1)	22(1)	22(1)	2(1)	4(1)	-3(1)
C(4)	22(1)	18(1)	23(1)	0(1)	0(1)	-4(1)
C(5)	22(1)	18(1)	21(1)	-2(1)	3(1)	1(1)
C(6)	17(1)	19(1)	20(1)	0(1)	4(1)	1(1)
C(7)	17(1)	16(1)	16(1)	0(1)	2(1)	2(1)
C(8)	17(1)	14(1)	16(1)	1(1)	1(1)	1(1)
C(9)	15(1)	16(1)	21(1)	2(1)	2(1)	1(1)
C(10)	18(1)	17(1)	17(1)	1(1)	2(1)	0(1)
C(11)	18(1)	20(1)	19(1)	0(1)	4(1)	0(1)
C(12)	18(1)	19(1)	21(1)	0(1)	4(1)	1(1)
C(13)	16(1)	18(1)	20(1)	3(1)	3(1)	0(1)
C(14)	20(1)	18(1)	23(1)	2(1)	3(1)	-1(1)
C(15)	17(1)	22(1)	27(1)	6(1)	2(1)	-3(1)
C(16)	18(1)	27(1)	25(1)	8(1)	7(1)	2(1)
C(17)	24(1)	23(1)	20(1)	2(1)	6(1)	3(1)
C(18)	19(1)	20(1)	20(1)	1(1)	2(1)	-1(1)

Table S5. Torsion angles [°] for 2c

C(6)-C(1)-C(2)-C(3)	0.92(16)
C(7)-C(1)-C(2)-C(3)	-178.63(10)
C(6)-C(1)-C(2)-Cl(1)	-179.57(8)
C(7)-C(1)-C(2)-Cl(1)	0.88(14)
C(1)-C(2)-C(3)-C(4)	-0.32(17)
Cl(1)-C(2)-C(3)-C(4)	-179.85(9)
C(2)-C(3)-C(4)-C(5)	-0.56(17)
C(3)-C(4)-C(5)-C(6)	0.79(17)
C(4)-C(5)-C(6)-C(1)	-0.16(17)
C(2)-C(1)-C(6)-C(5)	-0.68(16)
C(7)-C(1)-C(6)-C(5)	178.86(10)
C(6)-C(1)-C(7)-C(8)	0.25(18)
C(2)-C(1)-C(7)-C(8)	179.77(11)
C(1)-C(7)-C(8)-N(1)	0.55(19)
C(1)-C(7)-C(8)-C(9)	-179.61(10)
C(10)-N(1)-C(8)-C(7)	-179.87(11)
C(10)-N(1)-C(8)-C(9)	0.28(11)
C(10)-O(2)-C(9)-O(1)	-179.24(10)
C(10)-O(2)-C(9)-C(8)	0.60(10)
C(7)-C(8)-C(9)-O(1)	-0.61(19)
N(1)-C(8)-C(9)-O(1)	179.26(12)
C(7)-C(8)-C(9)-O(2)	179.57(9)
N(1)-C(8)-C(9)-O(2)	-0.56(11)
C(8)-N(1)-C(10)-O(2)	0.13(12)
C(8)-N(1)-C(10)-C(11)	179.13(10)
C(9)-O(2)-C(10)-N(1)	-0.49(12)
C(9)-O(2)-C(10)-C(11)	-179.59(9)
N(1)-C(10)-C(11)-C(12)	-179.44(11)
O(2)-C(10)-C(11)-C(12)	-0.44(16)
C(10)-C(11)-C(12)-C(13)	179.12(10)
C(11)-C(12)-C(13)-C(14)	-174.94(11)
C(11)-C(12)-C(13)-C(18)	3.00(19)
C(18)-C(13)-C(14)-C(15)	-0.61(17)
C(12)-C(13)-C(14)-C(15)	177.43(10)
C(13)-C(14)-C(15)-C(16)	0.02(17)
C(14)-C(15)-C(16)-C(17)	0.29(17)
C(15)-C(16)-C(17)-C(18)	0.01(17)
C(16)-C(17)-C(18)-C(13)	-0.62(17)

C(14)-C(13)-C(18)-C(17)	0.91(16)
C(12)-C(13)-C(18)-C(17)	-177.02(10)

Symmetry transformations used to generate equivalent atoms:

4.2. Crystallographic data for compound 4a

Table S6. Crystal data and structure refinement for 4a.

Empirical formula	C40 H22 F8 N2 O8 Pd2	
Formula weight	1023.39	
Temperature	100(2) K	
Wavelength	0.71073 Å	
Crystal system	Monoclinic	
Space group	C 2/c	
Unit cell dimensions	a = 17.320(4) Å	α= 90º
	b = 17.504(4) Å	β= 103.399(8)º.
	c = 12.717(3) Å	γ = 90º.
Volume	3750.7(14) $Å^3$	
Z	4	
Density (calculated)	1.812 Mg/m^3	
Absorption coefficient	1.056 mm^{-1}	
F(000)	2016	
Crystal size	0.320 x 0.250 x 0.230 mm^3	
Theta range for data collection	2.327 to 28.253º.	
Index ranges	-22<=h<=23, -23<=k<=23, -16<=l<=14	
Reflections collected	24985	
Independent reflections	4648 [R(int) = 0.0191]	
Completeness to theta = 25.242°	99.9 %	
Refinement method	Full-matrix least-squares on F^2	
Data / restraints / parameters	4648 / 0 / 271	
Goodness-of-fit on F^2	1.065	
Final R indices [I>2sigma(I)]	R1 = 0.0238, wR2 = 0.0599	
R indices (all data)	R1 = 0.0245, wR2 = 0.0604	
Largest diff. peak and hole	1.077 and -0.725 e.$Å^{-3}$	

Table S7. Atomic coordinates (× 10^4) and equivalent isotropic displacement parameters (Å^2× 10^3) for 4a. U(eq) is defined as one third of the trace of the orthogonalized U^{ij} tensor.

	x	y	z	U(eq)
F(1)	6733(1)	1180(1)	4662(1)	23(1)
O(1)	3295(1)	1644(1)	3387(1)	15(1)
N(1)	4159(1)	2562(1)	3189(1)	13(1)
C(1)	3482(1)	2409(1)	3411(1)	14(1)
O(2)	3990(1)	576(1)	3182(1)	20(1)
PD2	4777(1)	3541(1)	3485(1)	13(1)
F(2)	7295(1)	5129(1)	3697(2)	87(1)
C(2)	3949(1)	1251(1)	3183(1)	14(1)
O(3)	5466(1)	4514(1)	3769(1)	19(1)
F(3)	6512(1)	5549(2)	4596(2)	87(1)
C(3)	4495(1)	1854(1)	2907(1)	13(1)
O(4)	6153(1)	4239(1)	2498(1)	20(1)
C(4)	5403(1)	1749(1)	3296(1)	13(1)
C(5)	5856(1)	2225(1)	4207(1)	14(1)
C(6)	5644(1)	2970(1)	4437(1)	15(1)
C(7)	6078(1)	3337(1)	5368(2)	19(1)
C(9)	6943(1)	2241(1)	5827(2)	21(1)
C(8)	6715(1)	2971(1)	6053(2)	22(1)
F(00N)	6365(2)	5861(1)	2998(2)	102(1)
C(10)	6512(1)	1893(1)	4905(2)	17(1)
C(11)	2912(1)	2943(1)	3630(1)	15(1)
C(12)	2192(1)	2703(1)	3735(1)	15(1)
C(13)	1523(1)	3177(1)	3840(1)	16(1)
C(14)	801(1)	2815(1)	3835(2)	18(1)
C(15)	134(1)	3239(1)	3892(2)	21(1)
C(16)	185(1)	4027(1)	3984(2)	24(1)
C(17)	902(1)	4393(1)	4008(2)	27(1)
C(18)	1567(1)	3976(1)	3924(2)	22(1)
C(19)	6004(1)	4602(1)	3267(2)	19(1)
C(20)	6563(1)	5285(1)	3662(2)	31(1)

Table S8. Bond lengths [Å] and angles [°] for 4a.

F(1)-C(10)	1.362(2)
O(1)-C(1)	1.376(2)
O(1)-C(2)	1.400(2)
N(1)-C(1)	1.296(2)
N(1)-C(3)	1.450(2)
N(1)-PD2	2.0094(15)
C(1)-C(11)	1.433(2)
O(2)-C(2)	1.183(2)
PD2-C(6)	1.9695(18)
PD2-O(3)	2.0624(13)
PD2-O(4)#1	2.1714(14)
PD2-PD2#1	2.7869(6)
F(2)-C(20)	1.287(3)
C(2)-C(3)	1.513(2)
O(3)-C(19)	1.255(2)
F(3)-C(20)	1.297(3)
C(3)-C(4)	1.547(2)
C(3)-C(4)#1	1.591(2)
O(4)-C(19)	1.242(2)
O(4)-PD2#1	2.1714(14)
C(4)-C(5)	1.493(2)
C(4)-C(3)#1	1.591(2)
C(5)-C(10)	1.396(2)
C(5)-C(6)	1.404(3)
C(6)-C(7)	1.402(2)
C(7)-C(8)	1.394(3)
C(9)-C(10)	1.378(3)
C(9)-C(8)	1.387(3)
F(00N)-C(20)	1.308(3)
C(11)-C(12)	1.351(2)
C(12)-C(13)	1.457(2)
C(13)-C(14)	1.401(2)
C(13)-C(18)	1.402(3)
C(14)-C(15)	1.388(3)
C(15)-C(16)	1.386(3)
C(16)-C(17)	1.391(3)
C(17)-C(18)	1.389(3)
C(19)-C(20)	1.548(3)

C(1)-O(1)-C(2)	106.67(13)
C(1)-N(1)-C(3)	108.26(14)
C(1)-N(1)-PD2	127.21(12)
C(3)-N(1)-PD2	123.26(11)
N(1)-C(1)-O(1)	114.56(15)
N(1)-C(1)-C(11)	127.35(17)
O(1)-C(1)-C(11)	118.04(15)
C(6)-PD2-N(1)	88.81(7)
C(6)-PD2-O(3)	89.04(7)
N(1)-PD2-O(3)	176.72(6)
C(6)-PD2-O(4)#1	176.11(6)
N(1)-PD2-O(4)#1	94.60(6)
O(3)-PD2-O(4)#1	87.63(6)
C(6)-PD2-PD2#1	102.80(5)
N(1)-PD2-PD2#1	94.52(4)
O(3)-PD2-PD2#1	83.54(4)
O(4)#1-PD2-PD2#1	78.83(4)
O(2)-C(2)-O(1)	122.85(16)
O(2)-C(2)-C(3)	130.95(17)
O(1)-C(2)-C(3)	106.05(14)
C(19)-O(3)-PD2	118.67(12)
N(1)-C(3)-C(2)	103.31(13)
N(1)-C(3)-C(4)	117.35(14)
C(2)-C(3)-C(4)	119.19(14)
N(1)-C(3)-C(4)#1	118.49(14)
C(2)-C(3)-C(4)#1	110.93(14)
C(4)-C(3)-C(4)#1	87.92(12)
C(19)-O(4)-PD2#1	118.57(12)
C(5)-C(4)-C(3)	119.63(15)
C(5)-C(4)-C(3)#1	121.76(14)
C(3)-C(4)-C(3)#1	90.51(12)
C(10)-C(5)-C(6)	117.90(16)
C(10)-C(5)-C(4)	117.66(16)
C(6)-C(5)-C(4)	124.36(16)
C(7)-C(6)-C(5)	119.09(17)
C(7)-C(6)-PD2	118.04(14)
C(5)-C(6)-PD2	122.85(13)
C(8)-C(7)-C(6)	120.61(18)
C(10)-C(9)-C(8)	117.30(18)
C(9)-C(8)-C(7)	121.10(18)

F(1)-C(10)-C(9)	118.16(17)
F(1)-C(10)-C(5)	117.89(16)
C(9)-C(10)-C(5)	123.95(18)
C(12)-C(11)-C(1)	120.60(17)
C(11)-C(12)-C(13)	127.10(17)
C(14)-C(13)-C(18)	119.01(17)
C(14)-C(13)-C(12)	118.01(17)
C(18)-C(13)-C(12)	122.97(16)
C(15)-C(14)-C(13)	120.62(18)
C(16)-C(15)-C(14)	119.97(18)
C(15)-C(16)-C(17)	119.95(18)
C(18)-C(17)-C(16)	120.52(19)
C(17)-C(18)-C(13)	119.90(18)
O(4)-C(19)-O(3)	130.73(18)
O(4)-C(19)-C(20)	114.96(17)
O(3)-C(19)-C(20)	114.30(17)
F(2)-C(20)-F(3)	108.6(2)
F(2)-C(20)-F(00N)	107.1(3)
F(3)-C(20)-F(00N)	104.7(3)
F(2)-C(20)-C(19)	112.60(19)
F(3)-C(20)-C(19)	113.75(19)
F(00N)-C(20)-C(19)	109.6(2)

Symmetry transformations used to generate equivalent atoms:

#1 -x+1,y,-z+1/2

Table S9. Anisotropic displacement parameters ($Å^2$x 10^3)for 4a. The anisotropic displacement factor exponent takes the form: $-2\pi^2[h^2a^{*2}U^{11} + ... + 2 h k a^* b^* U^{12}]$

	U^{11}	U^{22}	U^{33}	U^{23}	U^{13}	U^{12}
F(1)	18(1)	24(1)	24(1)	0(1)	0(1)	6(1)
O(1)	13(1)	14(1)	20(1)	0(1)	5(1)	-1(1)
N(1)	12(1)	14(1)	12(1)	0(1)	2(1)	0(1)
C(1)	13(1)	16(1)	12(1)	0(1)	1(1)	-1(1)
O(2)	19(1)	15(1)	27(1)	1(1)	8(1)	0(1)
PD2	13(1)	12(1)	14(1)	-1(1)	3(1)	-1(1)
F(2)	33(1)	73(1)	157(2)	-63(2)	27(1)	-28(1)
C(2)	12(1)	18(1)	14(1)	0(1)	2(1)	0(1)
O(3)	21(1)	15(1)	22(1)	-2(1)	5(1)	-4(1)
F(3)	98(2)	103(2)	73(1)	-64(1)	49(1)	-78(2)
C(3)	12(1)	12(1)	14(1)	0(1)	2(1)	0(1)
O(4)	18(1)	18(1)	26(1)	-2(1)	5(1)	-4(1)
C(4)	11(1)	14(1)	13(1)	2(1)	2(1)	-1(1)
C(5)	12(1)	19(1)	12(1)	1(1)	3(1)	-3(1)
C(6)	14(1)	18(1)	14(1)	1(1)	2(1)	-2(1)
C(7)	20(1)	21(1)	16(1)	-2(1)	3(1)	-5(1)
C(9)	14(1)	31(1)	16(1)	2(1)	0(1)	-2(1)
C(8)	18(1)	31(1)	14(1)	-2(1)	0(1)	-7(1)
F(00N)	146(2)	37(1)	96(2)	21(1)	-27(2)	-51(1)
C(10)	14(1)	21(1)	18(1)	2(1)	4(1)	0(1)
C(11)	15(1)	16(1)	14(1)	-1(1)	3(1)	1(1)
C(12)	15(1)	16(1)	14(1)	0(1)	3(1)	1(1)
C(13)	14(1)	19(1)	14(1)	1(1)	4(1)	1(1)
C(14)	17(1)	20(1)	19(1)	2(1)	6(1)	1(1)
C(15)	16(1)	28(1)	21(1)	4(1)	8(1)	2(1)
C(16)	23(1)	28(1)	25(1)	5(1)	10(1)	11(1)
C(17)	30(1)	17(1)	37(1)	3(1)	12(1)	6(1)
C(18)	19(1)	19(1)	28(1)	1(1)	7(1)	0(1)
C(19)	19(1)	14(1)	23(1)	0(1)	2(1)	-3(1)
C(20)	33(1)	24(1)	37(1)	-7(1)	10(1)	-13(1)

Table S10. Torsion angles [º] for 4a.

C(3)-N(1)-C(1)-O(1)	3.7(2)
PD2-N(1)-C(1)-O(1)	-163.63(11)
C(3)-N(1)-C(1)-C(11)	-173.64(16)
PD2-N(1)-C(1)-C(11)	19.0(3)
C(2)-O(1)-C(1)-N(1)	3.59(19)
C(2)-O(1)-C(1)-C(11)	-178.77(15)
C(1)-O(1)-C(2)-O(2)	175.13(17)
C(1)-O(1)-C(2)-C(3)	-8.90(17)
C(1)-N(1)-C(3)-C(2)	-8.81(17)
PD2-N(1)-C(3)-C(2)	159.17(11)
C(1)-N(1)-C(3)-C(4)	-142.16(15)
PD2-N(1)-C(3)-C(4)	25.8(2)
C(1)-N(1)-C(3)-C(4)#1	114.29(16)
PD2-N(1)-C(3)-C(4)#1	-77.73(17)
O(2)-C(2)-C(3)-N(1)	-173.73(19)
O(1)-C(2)-C(3)-N(1)	10.75(17)
O(2)-C(2)-C(3)-C(4)	-41.4(3)
O(1)-C(2)-C(3)-C(4)	143.04(15)
O(2)-C(2)-C(3)-C(4)#1	58.3(2)
O(1)-C(2)-C(3)-C(4)#1	-117.23(15)
N(1)-C(3)-C(4)-C(5)	19.6(2)
C(2)-C(3)-C(4)-C(5)	-106.30(18)
C(4)#1-C(3)-C(4)-C(5)	140.80(12)
N(1)-C(3)-C(4)-C(3)#1	-108.08(13)
C(2)-C(3)-C(4)-C(3)#1	126.06(14)
C(4)#1-C(3)-C(4)-C(3)#1	13.16(17)
C(3)-C(4)-C(5)-C(10)	146.32(16)
C(3)#1-C(4)-C(5)-C(10)	-102.32(19)
C(3)-C(4)-C(5)-C(6)	-30.5(2)
C(3)#1-C(4)-C(5)-C(6)	80.9(2)
C(10)-C(5)-C(6)-C(7)	-2.1(3)
C(4)-C(5)-C(6)-C(7)	174.71(16)
C(10)-C(5)-C(6)-PD2	176.14(13)
C(4)-C(5)-C(6)-PD2	-7.1(2)
C(5)-C(6)-C(7)-C(8)	0.4(3)
PD2-C(6)-C(7)-C(8)	-177.90(14)
C(10)-C(9)-C(8)-C(7)	-0.5(3)
C(6)-C(7)-C(8)-C(9)	0.9(3)

C(8)-C(9)-C(10)-F(1)	179.21(16)
C(8)-C(9)-C(10)-C(5)	-1.3(3)
C(6)-C(5)-C(10)-F(1)	-177.91(15)
C(4)-C(5)-C(10)-F(1)	5.1(2)
C(6)-C(5)-C(10)-C(9)	2.6(3)
C(4)-C(5)-C(10)-C(9)	-174.38(17)
N(1)-C(1)-C(11)-C(12)	172.82(17)
O(1)-C(1)-C(11)-C(12)	-4.5(2)
C(1)-C(11)-C(12)-C(13)	-172.83(17)
C(11)-C(12)-C(13)-C(14)	173.94(18)
C(11)-C(12)-C(13)-C(18)	-4.8(3)
C(18)-C(13)-C(14)-C(15)	1.2(3)
C(12)-C(13)-C(14)-C(15)	-177.60(17)
C(13)-C(14)-C(15)-C(16)	-1.8(3)
C(14)-C(15)-C(16)-C(17)	0.7(3)
C(15)-C(16)-C(17)-C(18)	0.9(3)
C(16)-C(17)-C(18)-C(13)	-1.5(3)
C(14)-C(13)-C(18)-C(17)	0.5(3)
C(12)-C(13)-C(18)-C(17)	179.15(19)
PD2#1-O(4)-C(19)-O(3)	19.5(3)
PD2#1-O(4)-C(19)-C(20)	-159.25(14)
PD2-O(3)-C(19)-O(4)	9.9(3)
PD2-O(3)-C(19)-C(20)	-171.33(13)
O(4)-C(19)-C(20)-F(2)	-41.6(3)
O(3)-C(19)-C(20)-F(2)	139.5(2)
O(4)-C(19)-C(20)-F(3)	-165.7(2)
O(3)-C(19)-C(20)-F(3)	15.4(3)
O(4)-C(19)-C(20)-F(00N)	77.5(3)
O(3)-C(19)-C(20)-F(00N)	-101.5(3)

Symmetry transformations used to generate equivalent atoms:

#1 -x+1,y,-z+1/2
